# Ligand-specific changes in conformational flexibility mediate long-range allostery in the *lac* repressor

**DOI:** 10.1038/s41467-023-36798-1

**Published:** 2023-03-02

**Authors:** Anum Glasgow, Helen T. Hobbs, Zion R. Perry, Malcolm L. Wells, Susan Marqusee, Tanja Kortemme

**Affiliations:** 1 Department of Bioengineering and Therapeutic Sciences, University of California, San Francisco, CA 94158 USA; 2 Department of Biochemistry and Molecular Biophysics, Columbia University, New York, NY 10032 USA; 3 Department of Chemistry, University of California, Berkeley, Berkeley, CA 94720 USA; 4 Department of Molecular Biophysics and Biochemistry, Yale University, New Haven, CT 06511 USA; 5 Department of Physics, Columbia University, New York, NY 10032 USA; 6 Department of Molecular & Cell Biology, University of California, Berkeley, Berkeley, CA 94720 USA

**Keywords:** Molecular conformation, Biophysical chemistry, Mass spectrometry, DNA-binding proteins

## Abstract

Biological regulation ubiquitously depends on protein allostery, but the regulatory mechanisms are incompletely understood, especially in proteins that undergo ligand-induced allostery with few structural changes. Here we used hydrogen-deuterium exchange with mass spectrometry (HDX/MS) to map allosteric effects in a paradigm ligand-responsive transcription factor, the *lac* repressor (LacI), in different functional states (apo, or bound to inducer, anti-inducer, and/or DNA). Although X-ray crystal structures of the LacI core domain in these states are nearly indistinguishable, HDX/MS experiments reveal widespread differences in flexibility. We integrate these results with modeling of protein-ligand-solvent interactions to propose a revised model for allostery in LacI, where ligand binding allosterically shifts the conformational ensemble as a result of distinct changes in the rigidity of secondary structures in the different states. Our model provides a mechanistic basis for the altered function of distal mutations. More generally, our approach provides a platform for characterizing and engineering protein allostery.

## Abstract (web summary)

Using hydrogen-deuterium exchange, the authors propose a model explaining how a classic transcription factor undergoes changes in its conformational ensemble in response to different ligands.

## Introduction

Biological regulation ubiquitously depends on protein allostery, but few allosteric mechanisms are understood in structural detail. In the absence of high-resolution structural information, the Monod-Wyman-Changeux (MWC)^1^ and Koshland-Nemethy-Filmer (KNF)^2^ models for allostery have been used to explain the functional behavior of diverse allosteric systems. In a few cases, detailed structural information has enabled descriptions of allosteric mechanisms for proteins, both in terms of specific, sequential conformational changes resulting from perturbation^3–5^ and as shifts in the average conformational ensemble^6–8^. However, deducing an atomic-level mechanism for allosteric effects on protein function is challenging in several common scenarios: when the beginning- and end-state average structures are structurally indistinguishable; when the structural change occurs over a large distance with few structural differences in the allosteric site; or when the protein is disordered in one or both states.

**Fig. 1:**
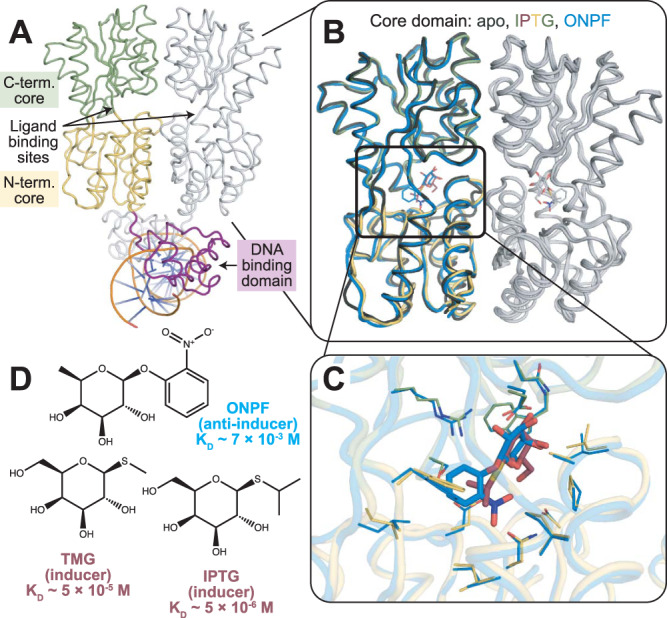
Ligands bind without dramatic structural changes in the core domain to control the structure of the DNA-binding domain allosterically. **A** X-ray crystal structure of dimeric LacI bound to the DNA operator. One subunit is shown in color (green: C-terminal subdomain; yellow: N-terminal subdomain; purple: DNA-binding domain) and the other subunit is shown in gray. PDB ID: 1EFA (ONPF-bound LacI without the tetramerization helix). **B** Overlaid structures of the core domains show similar backbone structures for apo-LacI (dark gray), IPTG-LacI (green, yellow, maroon), and ONPF-LacI (blue). PDB IDs: 1LBI (apo LacI core domain with tetramerization helix), 2P9H (IPTG-bound LacI core domain), 2PAF (ONPF-bound LacI core domain). **C** Overlaid structures of the ligand binding site in IPTG-LacI and ONPF-LacI reveal similar sidechain-atom positions, colored as in **B**. Thin sticks show amino acid sidechains. Thick sticks show ligands. **D** Chemical structures of ONPF, IPTG, and TMG with binding affinities to full-length LacI.

A paradigm example for these challenging scenarios is the *E. coli lac* repressor (LacI), a model allosteric transcription factor that regulates expression of the genes of the *lac* operon. Wild-type (WT) LacI forms a dimer of dimers, and each dimer can bind to one of three DNA operator sequences in the *E. coli* genome. The MWC and KNF models for protein allostery were established in part from studies of LacI after observing bacterial responses to sugar ligands^9^. The ligands bind in a pocket in the core domain of LacI, 40 Å away from the DNA-binding surface of the DNA-binding domain (Fig. 1A), and allosterically modulate the binding affinity of LacI for its DNA operator. Interestingly, X-ray crystal structures of the core domain are highly similar in different ligand-bound states as well as the apo state (<1.5 Å heavy atom RMSD) (Fig. 1B, C), with the observable differences among X-ray crystal structures localized mainly to sidechain positions in the N-terminal core domain monomer-monomer interface^10,11^. The high structural similarity of the core domains, combined with a lack of X-ray crystal structures for a non-operator-bound DNA-binding domain, complicates mechanistic understanding of ligand-regulated allostery in LacI despite more than five decades of study using structural^12–14^, genetic^15–21^, computational^3,22–24^, and biophysical^25–33^ methods.

Adding to this challenge, chemically similar molecules binding in the core domain can have opposite effects on LacI-regulated gene expression (Fig. 1D). When bound to the inducers allolactose or isopropyl-β-D-1-thiogalactopyranoside (IPTG), LacI binds ~1000-fold less efficiently to the DNA operator than apo-LacI^34^, allowing for the transcription of downstream genes. However, when bound to the anti-inducer *ortho*-nitrophenyl-β-D-fucoside (ONPF), LacI is stabilized in the operator-bound state by 2- to 5-fold over apo-LacI. Other ligands such as *ortho*-nitrophenyl-β-galactoside (ONPG) bind LacI but produce no observable functional effects. Inducer potency has been hypothesized to depend on the molecule’s binding affinity for LacI, the presence of an O6 hydroxyl group on the sugar ring, an optimal size for the C1 substituent, and the flexibility of the substituent linkage to the sugar ring^35,36^.

Several models have been proposed to explain the inducer-specific effects on gene expression by LacI. Crystallography and molecular dynamics simulations support a model in which inducer binding leads to new intramolecular interactions in the N-terminal subdomain of the core and partial unfolding of the DNA-binding domain, with the C-terminal subdomain of the core remaining unchanged^13,37,38^. Crystallographic studies of LacI bound to the inducer IPTG or to DNA revealed similar structures of the core domain, and a small shift (all-atom RMSD 1.5 Å) of the N-terminal subdomains of the core closer together in the IPTG-bound structure as compared to DNA-bound LacI (Supplementary Fig. 1)^13^. Because of the structural similarity of the core domain in the apo, DNA-bound, inducer-bound, and anti-inducer-bound states of the protein, a detailed molecular mechanism for allosteric changes in the core domain upon ligand binding remains elusive. For example, effects on inducer binding and gene expression from mutations or insertions in localized regions of LacI have informed hypotheses that are inherently limited to the role of these regions^21,39–41^, but do not explain why mutations outside these regions also affect the functional response of the repressor^42,43^, or why chimeric repressors have attenuated responses^44–46^. Further, no study has systematically tested the effects of mutations on anti-inducer binding or the functional response of anti-induction, so it is difficult to determine whether previously proposed allosteric mechanisms are specific to inducer molecules. Finally, while several models propose that the transition from the DNA-bound state of LacI to the inducer-bound state requires the destabilization of a hinge helix linking the core and DNA binding domains and the participation of binding pocket residues in both subunits^22,47–49^, it has remained unclear how this change is propagated over a distance of 40 Å across the monomer-monomer interface and between both ligand-binding sites and hinge helices.

Here, we map allosteric changes across the entire core domain of wild-type LacI in different functional states via hydrogen-deuterium exchange with mass spectrometry (HDX/MS). HDX/MS has been used previously to monitor multiple liganded states in pyruvate kinase^50–53^, thrombin^54^, and other proteins^55,56^. We define “functional states” by binding partner, where IPTG-LacI denotes the IPTG-bound protein; ONPF-LacI the ONPF-bound protein; ONPF-DNA-LacI the protein bound to ONPF and the *lac* operator at the same time; and apo-LacI the unbound protein. We propose a model for mutually exclusive low-energy conformational ensembles of LacI in inducer- and DNA-bound states. Using a combined computational and experimental approach, we show that protein, ligand, and solvent atoms together play a central role in modulating the rigidity of secondary-structure elements in the ligand-binding core domain of LacI to control its functional state, without dramatic conformational changes. Our model for allosteric regulation in the core domain of LacI can inform future efforts to rationally redesign the protein to control gene expression in response to new ligands, and establishes a method for characterizing how allostery in other proteins is conserved, evolved, or affected in disease.

## Results

We set out to determine the mechanism of allosteric changes in dimeric LacI (truncating the tetramerization domain, see Methods) by comparing structural and functional differences between DNA-bound states and inducer-bound states using HDX/MS. The DNA-bound and inducer-bound states are the key functionally relevant states of LacI. We note that in dimeric LacI, the DNA-bound state comprises two LacI protomers per operator sequence, whereas the non-DNA-bound state is in a monomer-dimer equilibrium (K_D_ ~ 8 × 10^−8^ M for the monomer-dimer equilibrium of apo dimeric LacI)^57,58^. To study inducer-bound states, we complexed LacI with IPTG, a strong inducer molecule (LacI-IPTG K_D_ ~ 5 × 10^−6^ M), and thiomethyl β-D-galactoside (TMG), a weaker inducer molecule (LacI-TMG K_D_ ~ 5 × 10^−5^ M)^35^ (Fig. 1D). To study DNA-bound states, we used the LacI-*lac* operator complex, with and without the anti-inducer molecule ONPF (LacI-operator K_D_ < 10^−12^ M, LacI-ONPF K_D_ ~ 7 × 10^−3^ M)^35^ (Fig. 1D). (All K_D_ values referenced here were determined for full-length LacI, but the IPTG and ONPF K_D_ values were also measured by isothermal calorimetry and are very close for dimeric LacI^32^.) This strategy generated a set of LacI states with a range of binding affinities for the operator DNA (in order from unmeasurably weak to strongest): IPTG-LacI; TMG-LacI; DNA-LacI; and the ternary ONPF-DNA-LacI. We confirmed the effects on gene expression of IPTG, TMG, and ONPF ligands binding to LacI using a plate-based cell culture assay (Methods), in which LacI regulates the expression of a green fluorescent protein (GFP) that is encoded downstream of the LacI DNA operator sequence on a plasmid (Supplementary Fig. 2A). We also confirmed the functionality of our purified dimeric LacI by measuring in vitro binding to the *lac* operator DNA using bio-layer interferometry consistent with previous results^32,57,59^ (Supplementary Fig. 2B, Methods).

For each state, we monitored amide hydrogen-deuterium exchange at the peptide level over a four-hour time period using mass spectrometry (see Methods). From the full set of mass spectra representing more than 300 total peptides, we curated a set of 57 peptides from the core domain of LacI for which we could collect replicate data in six different states (apo, IPTG-LacI, TMG-LacI, ONPF-LacI, DNA-LacI, and ONPF-DNA-LacI) as well as a fully deuterated control HDX/MS experiment (in order to correct for back-exchange; see Methods). The curated dataset contains 97% peptide coverage for the core domain, excluding the extreme C-terminal 22 amino acids (Supplementary Fig 3) for which we could not obtain a complete dataset (data for each time point and each state with back-exchange). In general, all hydrogen exchange data are consistent with EX2 regime behavior, indicating that we are probing the equilibrium state conformational ensembles populated under each condition.

### Comparison of inducer-bound and DNA-bound states

**Fig. 2:**
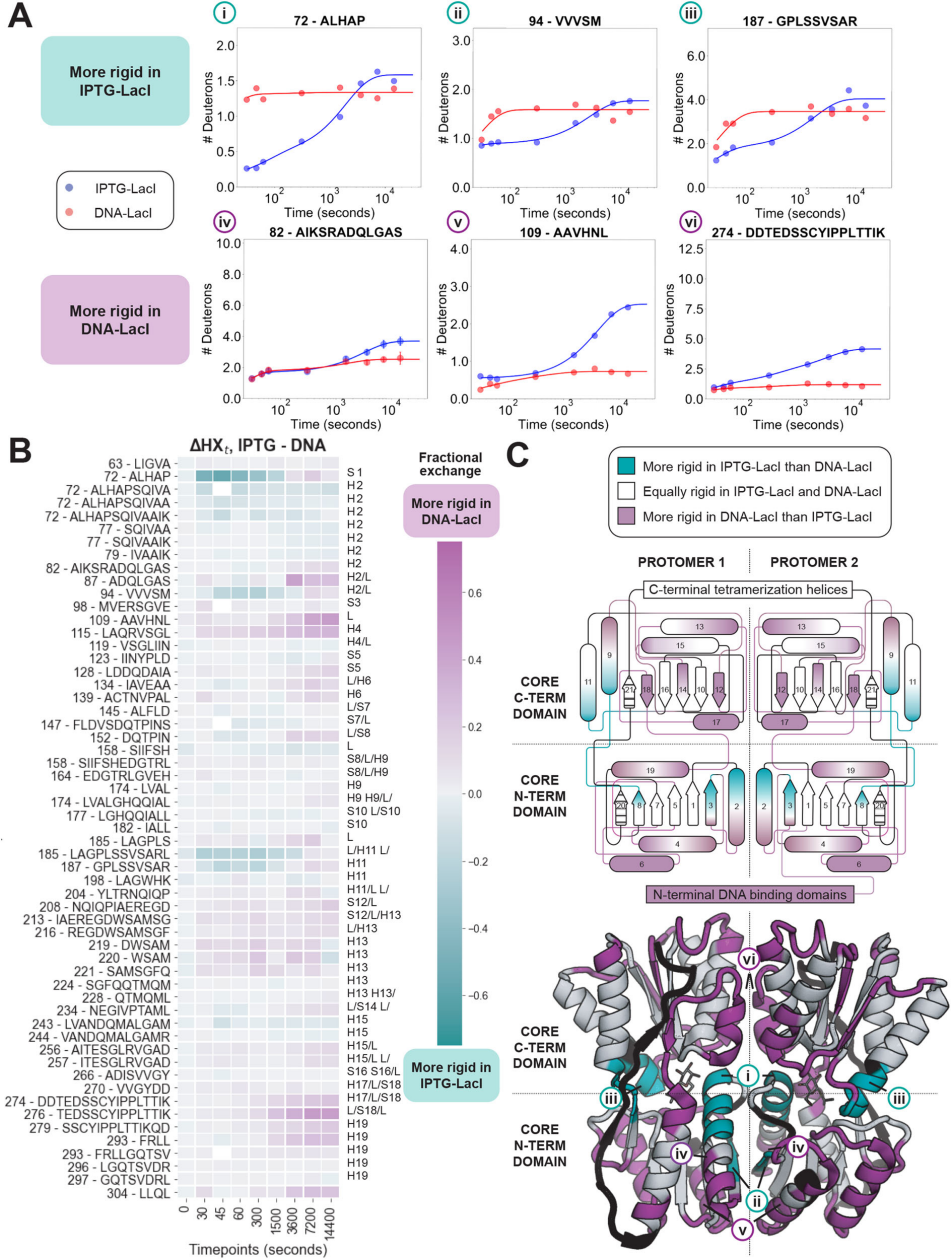
Conformational differences between IPTG-LacI and DNA-LacI. **A** Uptake plots for peptides marked in **C** with data presented as means for *n* = 2 individual measurements on biologically independent samples, except panel iv, for which *n* = 3 for IPTG-LacI a*n*d *n* = 5 for DNA-LacI. Error bars are standard deviations. **B** Comparison of fractional differences. Purple: less exchange in DNA-LacI; teal: less exchange in IPTG-LacI. Right-side labels correspond to the labels in (C, top). **C** Differential exchange, colored as in (B). Striped and dark gray regions indicate no data. PDB ID: 2P9H (ONPF-bound LacI without the tetramerization helix).

We first compared exchange patterns for core domain peptides in the IPTG-LacI and DNA-LacI states (Fig. 2, Supplementary Fig. 4). We quantified the level of exchange in both states by calculating the deuterium uptake as a function of time for each peptide (Supplementary Fig. 5), and fit these data to a nonlinear regression model to estimate the number of slow-, medium-, fast-, and non-exchanging protons in each peptide/state, and the resulting rates associated with each group (slow, medium, and fast) (Methods). These functions are plotted in all uptake plots with the experimental measurements at each timepoint to guide the eye. In the deuterium-uptake plots in Fig. 2A, increased rates of exchange for a peptide indicate increased structural flexibility associated with decreased participation in secondary structure hydrogen bonds by the peptide’s backbone amide groups. Figure 2B shows this behavior for the curated set of 57 peptides in the core domain, and Fig. 2C illustrates these differences in H-D exchange between IPTG-LacI and DNA-LacI mapped onto the structure of the core domain. To analyze differences in H-D exchange between functional states, residues were labeled “more rigid” in either state if a larger than 20% difference in exchange was observed at three or more individual timepoints in HDX/MS peptides that included that residue; our main conclusions hold with increased stringency in this classification (Supplementary Fig. 6A, B).

When comparing IPTG-LacI to DNA-LacI, we observed structural regions of both decreased deuterium uptake (less exchange in IPTG-LacI relative to DNA-LacI, teal, Fig. 2A, C, i-iii, Supplementary Figs. 6A, B, 7A) and regions of increased deuterium uptake (more exchange in IPTG-LacI, purple, Fig. 2A, C, iv-vi, Supplementary Fig. 6A, B). (In this context, “more exchange in IPTG-LacI” is the same as “less exchange in DNA-LacI.”) Regions with *decreased* deuterium uptake in IPTG-LacI relative to DNA-LacI are located near the ligand-binding pocket (Fig. 2A, C, i, N-terminal end of helix 2) and the monomer-monomer interface in the N-terminal subdomain (Fig. 2A, C, ii, beta strand 3). We hypothesize that the decreased deuterium uptake for beta strand 3 reports on the formation of a hydrogen bond between the backbone atoms of position 96 across the beta sheets of both N-terminal subdomains in the LacI dimer (Supplementary Fig. 8). We also observed decreased deuterium uptake in regions distal to the monomer-monomer interface (Fig. 2C, iii, N-terminal portion of helix 11). Other regions in this category include the loop connecting beta strand 8 (N-terminal subdomain) and helix 9 (C-terminal subdomain) and residues 159–162 (Fig. 2C).

Conversely, regions with increased deuterium uptake (purple, Fig. 2, Supplementary Fig. 6) in IPTG-LacI compared to DNA-LacI included alternating beta strands in the C-terminal subdomain beta sheet (strands 12, 14, and 18) and all of the N-terminal subdomain helices (C-terminal end of helix 2, and helices 4, 6, and 19), which provide an interacting surface for the DNA-binding domain. Peptides from N-terminal subdomain helices 2 and 4 are shown in Fig. 2C, panels iv and v, respectively. Other regions in this category included residues in the C-terminal subdomain at the monomer-monomer interface (helix 13, residues 221–235, and helix 17, residues 276–282, Fig. 2C, vi, Supplementary Fig. 7B). Of the nine loops that flank each ligand binding site, five loops exchanged more in IPTG-LacI than DNA-LacI.

**Fig. 3:**
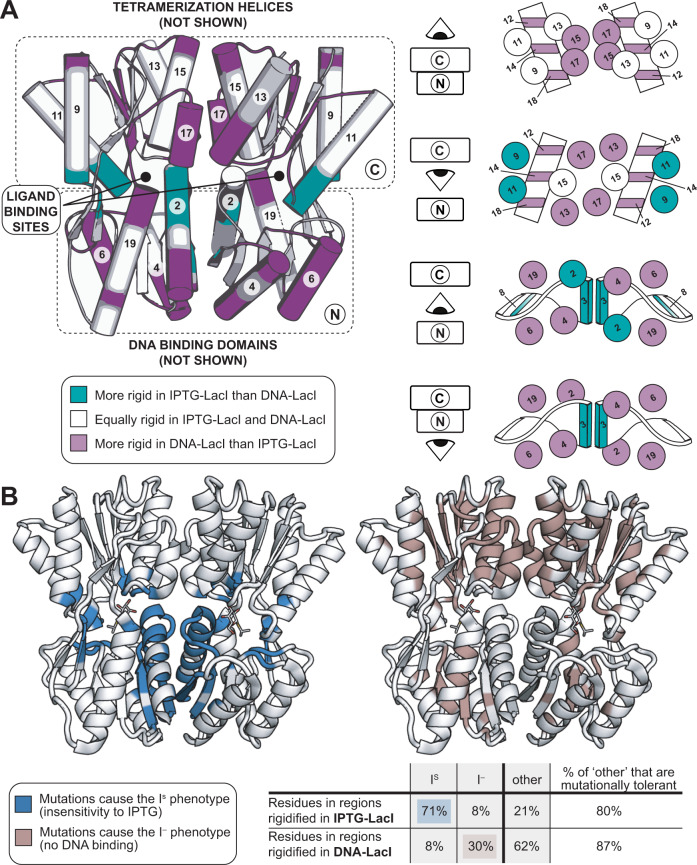
Structural and functional differences between IPTG-LacI and DNA-LacI. **A** Diagrams on the right are cross-sectioned according to the schematic on the left. Secondary structure elements are labeled according to Fig. 2C, top. Purple: less exchange in DNA-LacI; teal: less exchange in IPTG-LacI. **B** LacI models colored by mutational phenotype classes: I^S^ (constitutive repression) and I^−^ (constitutive expression)^19^. The table summarizes mutational phenotype data as percentages of residues in differentially rigidified regions observed in HDX/MS experiments. PDB ID: 2P9H (IPTG-bound LacI core domain).

Taken together, and supported by comparison to mutational data in the next section, these results suggest a model where DNA-bound and inducer-bound states of the *lac* repressor adopt mutually incompatible low-energy conformational ensembles (Fig. 3A). DNA-LacI is characterized by an overall rigid structure of both the N- and C-terminal core domains, where the N-terminal subdomain makes extensive interactions with a well-folded operator-bound DNA-binding domain in the DNA-LacI crystal structure. In the IPTG inducer-bound state, the ligand-binding pocket periphery as well as several secondary structure elements at the N-terminal core monomer-monomer interface are further rigidified. Accordingly, the HDX/MS data suggest increased interactions between the LacI N-terminal core subdomains. Importantly, this rigidification upon inducer binding occurs alongside an increase in flexibility in all of the N-terminal subdomain helices at the interface with the DNA-binding domain, whether or not the operator DNA is bound (Supplementary Fig. 9, see ΔHX_t_ IPTG-DNA plot). In addition, the C-terminal core subdomain helices 13 and 17, which flank the central helix 15 at the monomer-monomer interface, become *more* flexible in the inducer-bound states, in concert with increased flexibility in every other beta strand in the C-terminal subdomain beta sheet. This effect could be explained by LacI being in a monomer-dimer equilibrium in the IPTG state. In contrast, in the DNA-bound state, LacI is necessarily dimeric, likely contributing to a less flexible monomer-monomer interface as compared to non-DNA-bound functional states.

BecauseIPTG-LacI binds DNA with 1000-fold reduced affinity compared to apo-LacI^35,58^, we were curious how IPTG-LacI interacts with DNA. To explore this, we performed an additional set of HDX/MS experiments on the ternary IPTG-DNA-LacI complex under conditions in which we expect that LacI is close to 100% IPTG-bound and 50% DNA-bound. Compared to DNA-LacI, we observed reduced rigidity of three out of four helices in the DNA-binding domain that make direct interactions with DNA in the DNA-LacI crystal structure (Supplementary Figs. 10, 11, representative peptides: helix 1, residues 7–12; helix 2, residues 16–23; helix 3, residues 36–45; and hinge helix, residues 46–62). Similarly, at the C-terminal monomer-monomer interface peptide rigidity is reduced in IPTG-DNA-LacI compared to DNA-LacI (Supplementary Figs. 10, 11, e.g., 212-PIAEREGDWSAMSGFQQTM and 274-DDTEDSSCYIPPLTT). The effect is not as dramatic as in IPTG-LacI, likely because of the weak DNA binding of the IPTG-LacI complex. At the inducer binding pocket periphery, IPTG-LacI and IPTG-DNA-LacI behave similarly, suggesting similar IPTG–LacI interactions regardless of the presence of operator DNA at these concentrations of binding partners (Supplementary Figs. 10, 11, e.g., 158-SIIFSHEDGTRL and 185-LAGPLSSARL). These data demonstrate in structural detail how the monomer-dimer equilibrium and the rigidity of secondary structures in LacI is modulated by both DNA and IPTG binding. We note, however, that higher DNA concentrations in the experiment would impact these data, and that our model does not include information on an order of events for release from the DNA operator upon IPTG binding.

To further characterize differences in the rigidity of secondary structure elements between inducer- and DNA-bound states, we also used an alternate inducer. With few exceptions localized to the ligand binding pocket, we found that H-D exchange in TMG-LacI was similar to IPTG-LacI (Supplementary Fig. 9). Likewise, H-D exchange in ONPF-DNA-LacI was similar to DNA-LacI (Supplementary Fig. 9). At the pocket-peripheral regions (e.g. 158–SIIFSH and 185–LAGPLSSVSARL, Supplementary Fig. 9), the diminished magnitude of differential exchange compared to the DNA-bound state in TMG-LacI vs. IPTG-LacI is also likely due to lower binding affinity of LacI for TMG, and the sub-saturation TMG concentration in the experiment (K_D_ = 50 µM, experiment concentration = 15 µM).

### Comparison to mutational data

We next compared our model for conformational changes in the LacI core domain in the presence of the inducer (Fig. 3A) to the extensive mutational phenotype data for LacI, where “phenotype” is defined by the effect of point mutations on gene expression. In one landmark study, 4000 LacI point mutants were tested for effects on gene expression^19^. The authors binned each WT amino-acid residue position according to its tolerance to mutation, and mapped these classifications onto the crystal structure of LacI^14^ (Supplementary Table 1, Supplementary Fig. 12). The main mutational phenotypes observed in the study were: similar inducer response to WT LacI; unresponsive to inducer binding (I^S^); or constitutively inducing (I^−^).

We find a striking correlation between the phenotypic effects of these point mutants and our H-D exchange data. Regions rigidified in IPTG-LacI (teal in Fig. 2C, see Methods) correlated with positions at which mutations commonly cause an I^s^ phenotype, whereas regions rigidified in DNA-LacI (purple in Fig. 2C) correlated with positions at which mutations cause an I^-^ phenotype. Mutations at 71% of the core domain residues that we observed to be rigidified in IPTG-LacI cause the I^S^ phenotype (Fig. 3B), but mutations to only 8% of these rigidified residues cause the I^-^ phenotype. Conversely, mutations at 30% of the core domain residues in regions that we observed to be rigidified in DNA-LacI cause the I^−^ phenotype, while mutations to only 8% of these residues cause the I^S^ phenotype. There were also mutations in regions with altered rigidity that cause neither phenotype (“other”), but the majority of these positions are tolerant to substitutions (Fig. 3B, last two columns in the table). Interestingly, when we increased the stringency for differential rigidity classification in our analysis, there were no mutationally tolerant positions in the “other” category (Supplementary Fig. 6B). This observation suggests that mutations to the smallest set of positions with the most dramatic differences in HDX/MS between the DNA- and IPTG-LacI states most strongly affect ligand-driven allostery and protein stability. Although the HDX/MS experiment integrates the responses of several amino acids into peptide-level data, overlapping peptides improve the resolution of the data. At the same time, mutagenesis experiments in which a cell-level phenotype is observed do not represent every possible mutation at any position. Even so, it can be instructive to compare these informative data, despite these limitations. Overall, we find considerable agreement between the phenotype assignment groups and differences in exchange between DNA-LacI and IPTG-LacI.

In addition, our data are consistent with focused biochemical studies on the effects of individual mutations in the monomer-monomer interface and the binding-pocket periphery (Supplementary Fig. 13, Supplementary Discussion, mutational analysis). Taken together, the HDX/MS and mutational phenotype data for LacI support a model for allosteric response in which the differential rigidification of secondary structures relative to one another in each state modulates protein function. Mutations in regions that are uniquely rigidified in IPTG-LacI may destabilize the conformational ensemble of that state, leading to constitutive repression. Conversely, mutations in regions that are more rigid in DNA-LacI may destabilize the DNA-LacI conformational ensemble, leading to constitutive transcription. Notably, the regions of the protein that are rigidified in either state are not confined to the ligand-binding pocket or the DNA-binding surface.

### Effect of anti-inducer binding

**Fig. 4:**
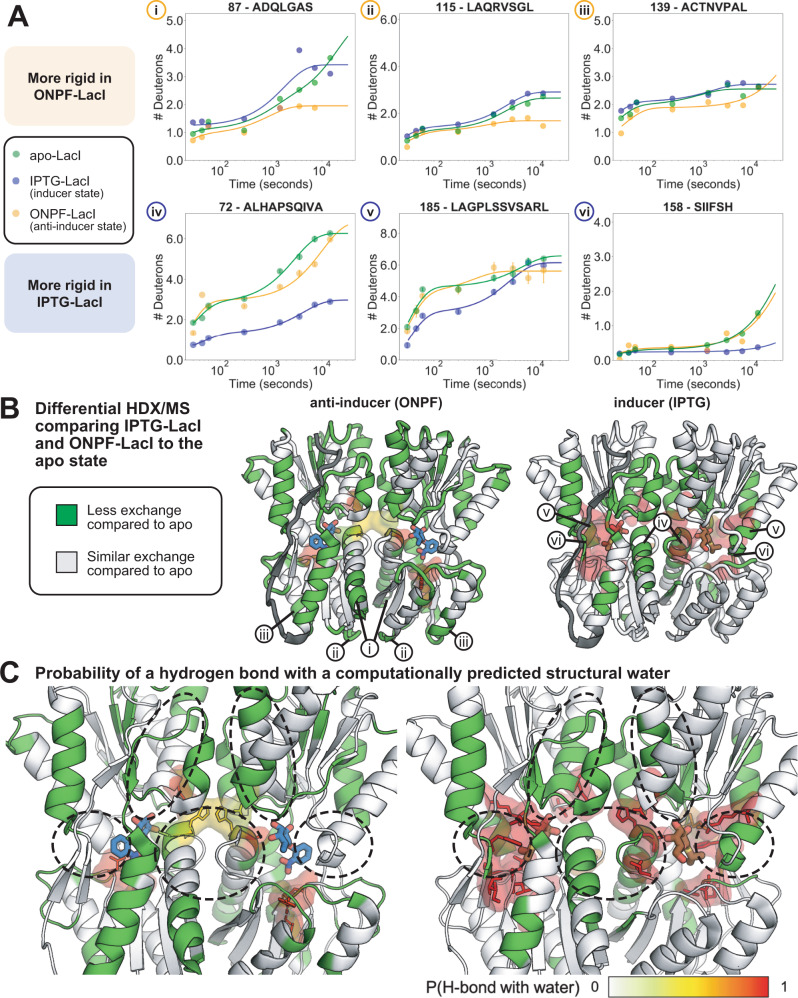
Predicted solvent-mediated hydrogen bonding networks in the ligand-binding pocket. **A** Deuterium uptake plots for peptides *i-vi* marked in **B**. The data represent means for *n* = 2 measurements on biologically independent samples, except panel iv which has *n* = 5 for all states, and panel v which has *n* = 5 for IPTG-LacI and ONPF-LacI, and *n* = 4 for DNA-LacI. Error bars show standard deviations. **B**, **C** ONPF-LacI (left) and IPTG-LacI (right) core domain structures colored by differences in exchange compared to apo-LacI. Green regions are rigidified as compared to apo-LacI. Dark gray indicates no data. Surfaces show predicted probabilities of residue interactions with structural water molecules. **C** Ligand binding sites in **C**. Circles indicate regions that are rigidified in IPTG-LacI but not ONPF-LacI.

We next sought to identify differences in the conformational ensembles between IPTG-LacI and ONPF-LacI. The two ligands IPTG and ONPF have opposite functional effects, where IPTG destabilizes and ONPF stabilizes the DNA-bound state^13,35^. To facilitate the comparison, and because IPTG-LacI has 1000-fold reduced binding to DNA^35,58^, we compared H-D exchange for IPTG-LacI and ONPF-LacI without the DNA operator to the apo state. Because of solubility limitations for ONPF, we carried out two sets of HDX/MS experiments: one at sub-saturating conditions, and one with ONPF added to the D_2_O buffer at the solubility limit. Figure 4 and Supplementary Fig. 4 show HDX/MS data with ONPF at 300 µM under sub-saturating conditions but with an experimental setup otherwise identical to all other states; Supplementary Figs. 14–17 show data with ONPF added at the solubility limit of 14.6 mM, which is above the LacI-ONPF KD, to all HDX/MS buffers, see Methods. We observed similar exchange behaviors for LacI peptides in both sets of ONPF-LacI experiments (Supplementary Figs. 14–17).

Comparing all ligand-bound LacI experiments to the apo state, we found that rigidification (less exchange) of particular secondary structure elements near the ligand binding pocket and in the interface between the N- and C-terminal core subdomains (the N-terminal part of helix 2, strand 3, strand 8 and the loop C-terminal to strand 8, helix 11, helix 15 and the loop N-terminal to helix 15) was unique to IPTG-LacI and not observed in ONPF-LacI (Fig. 4A, B, iv–vi). In contrast, ONPF binding rigidified regions distal to the ligand binding site in the N-terminal subdomain (Fig. 4A, B, i–iii, the C-terminal end of helix 2 and the loop connecting it to strand 3, the loop between strand 3 and helix 4, the loop between helix 4 and strand 5, the loop between strand 5 and helix 6, the loop between helix 6 and strand 7, the loop connecting strands 7 and 8, and the C-terminal ends of helices 6 and 19). Many of these regions interface directly with the DNA-binding domain (although DNA is absent in these HDX/MS experiments). Taken together, these results suggest that IPTG and ONPF selectively stabilize different secondary structure elements in the core domain to shift the conformational ensemble of LacI to different, mutually exclusive low-energy states. Unlike IPTG binding, ONPF binding does not cause changes to the structure of LacI that are incompatible with the observed conformational ensemble of DNA-LacI (Supplementary Fig. 9, compare ΔHX_t_ ONPF-APO, ΔHX_t_ IPTG-APO, and ΔHX_t_ APO-DNA plots).

The rigidification of the N-terminal core subdomain in ONPF-LacI in the absence of DNA suggests that ONPF binding could order this subdomain, and possibly the DNA-binding domain, for DNA binding. To test this idea, we compared HDX/MS data for peptides in the DNA-binding domain in different functional states of LacI. The structure of the DNA-binding domain has only been solved by X-ray crystallography in the DNA-LacI state (Supplementary Fig. 18A). In these crystal structures, the N-terminal subdomain beta strands and helices form electrostatic interactions with a well-folded DNA-binding domain. Interestingly, while HDX/MS confirmed that the DNA-binding domain is well-structured in DNA-LacI and ONPF-DNA-LacI, we observed a range of hydrogen exchange behaviors in the DNA-binding domain for the non-DNA-bound ligand-bound states. The DNA-binding domain underwent the most H-D exchange across timepoints in IPTG-LacI, particularly in the hinge helix (residues 50-57) that connects the DNA-binding domain to the core domain (Supplementary Fig. 18B), and was therefore the least structured among the states (and some of these peptides may pass into the EX1 regime due to cooperative unfolding, unlike the core domain peptides). This observation is consistent with previously reported small-angle X-ray scattering (SAXS) and NMR data for the LacI hinge helix, which became unstructured upon removal of DNA^58,60,61^. However, decreased H-D exchange in the DNA-binding domain for ONPF-LacI when compared to IPTG-LacI even in the absence of DNA suggests the possibility that ONPF binding may structure the DNA-binding domain, thereby improving the binding affinity of LacI for the DNA operator when compared to apo-LacI (Supplementary Figs. 16 and 18C, LacI peptides corresponding to residues 1–60).

### Water-mediated interactions in the ligand-binding site

Several secondary structure elements near the ligand binding pocket that show decreased exchange only in IPTG-LacI and/or TMG-LacI, such as helix 2, strand 8 and helix 11 (Supplementary Figs. 4, 9), do not make direct contacts with ligands in X-ray crystal structures (Supplementary Fig. 19). We hypothesized that decreased exchange in these regions may result from the formation of additional solvent-mediated hydrogen bonds in the ligand binding pocket that form upon binding inducers, but not anti-inducers. Crystal structures of IPTG-LacI show such solvent-mediated interactions^13^. In addition, mutations at binding pocket residues D149 and N125, which form water-mediated hydrogen bonds to IPTG in the crystal structure, decrease the protein’s affinity for IPTG^62^.

To explore the potential role of water-mediated hydrogen bonding networks in the ligand binding pocket, we applied a computational “semi-explicit” solvation protocol in the macromolecular modeling program Rosetta^63^ to predict the positions of structural water molecules in IPTG-LacI, ONPF-LacI, and apo-LacI. We defined “structural waters” as water molecules that make three or four hydrogen bonds with atoms in the protein or ligand. As inputs, we first relaxed the experimentally solved crystal structures of IPTG-LacI and ONPF-LacI (Supplementary computational methods). The computational protocol then places water molecules in hydrogen bonding geometries relative to protein polar groups. Highly coordinated water molecules in the protein structure are kept, with or without “solvated” sidechains allowed to change rotameric conformations in Monte Carlo simulations. Only one high-resolution structure of LacI that includes electron densities for water molecules in the ligand binding sites is available (protein databank (PDB) ID 2P9H [https://www.wwpdb.org/pdb?id=pdb_00002p9h] (IPTG-bound LacI core domain), the core domain of IPTG-LacI). Using our computational strategy without sidechain repacking, we first confirmed that the positions of our computationally predicted structural water molecules in IPTG-LacI are within 0.5 Å of several water molecules observed in this structure (Supplementary Fig. 20). We then placed structural water molecules in the ligand binding pockets of ONPF-LacI and apo-LacI from crystal structures using the same protocol (Fig. 4C, Supplementary Fig. 21). Based on 100 models for each LacI state, we calculated the probability that an atom in the protein or ligand makes a hydrogen bond with a computationally placed structural water in the ligand binding pocket (Supplementary Fig. 21).

We observed a distinctive correspondence between regions with ligand-induced rigidification of secondary structures as measured by HDX/MS (Fig. 4A, B) and the calculated probability for water-mediated hydrogen bonding among residues in the ligand binding pocket (Fig. 4C, circled regions correspond to regions iv, v, and vi highlighted in Fig. 4A, bottom row, and 4B, right panel, as well as helix 15). Most strikingly, we predict an extensive water-mediated hydrogen bonding network for IPTG-LacI (Fig. 4C, right). In contrast, this network is not observed in the ONPF-LacI simulations (Fig. 4C, left). This result supports a model in which structural water molecules play a role in differentially rigidifying LacI in different functional states, where water-mediated hydrogen bonding networks contribute to increased interactions in the ligand binding pocket between the N- and C-terminal core subdomains in IPTG-LacI, but not ONPF-LacI.

## Discussion

We used a computational and experimental approach to map the allosteric response of the core domain of LacI to different binding partners in six different functional states, at secondary structure resolution. Because the X-ray crystal structures of the core domains of DNA-LacI and IPTG-LacI are almost identical, our approach addressed the long-standing question of how allosteric effects in LacI are transmitted across more than 40 Å in the absence of sizeable conformational changes between functional states. Our model for how inducer binding decreases the affinity of LacI for the DNA operator centers on selective rigidification of particular secondary structure elements relative to each other, shifting the conformational ensemble of the protein between mutually incompatible DNA-bound and inducer-bound states. This model assumes that the H-D exchange observed in the experiment primarily occurs as a result of changes in the equilibrium ensemble that may be relevant for function (although HDX/MS provides an incomplete picture of protein dynamics^64^). Nevertheless, our results describing detailed changes in the LacI core domain are consistent with data from structural and biochemical studies. Moreover, the differences in conformational ensembles between the inducer-bound states and DNA-bound states that we observed by HDX/MS and predicted using computational methods are remarkably consistent with the altered functions of thousands of documented LacI mutants, and hence provide mechanistic insights expanding on the original MWC and KWF phenomenological models.

**Fig. 5:**
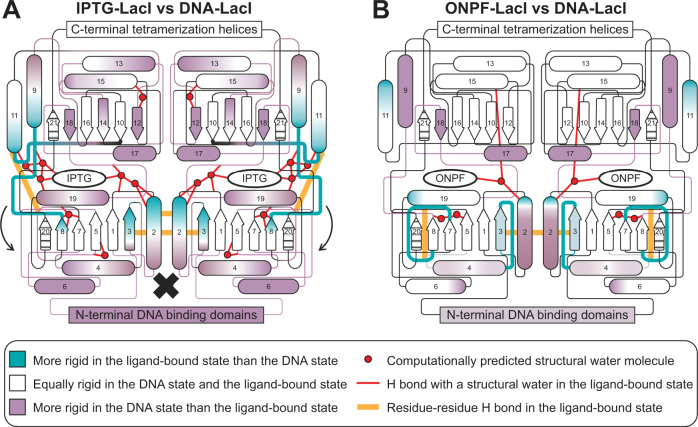
Consolidated models for ligand-specific changes in the conformational ensemble in IPTG-LacI and ONPF-LacI, as compared to DNA-LacI. Schematics summarizing the rigidity of LacI core domain secondary structure elements in IPTG-LacI and ONPF-LacI as compared to DNA-LacI, with rigidified regions in each ligand-bound state shown in teal, de-rigidified regions shown in purple, and regions for which there is no data shown with stripes. Red circles show the positions of predicted structural water molecules, and red lines show the interactions of the predicted structural water molecules with atoms in ligands or amino acid residues. Orange lines represent hydrogen bonds uniquely formed among protein atoms in each ligand-bound state as compared to DNA-LacI. Rigidified loops in each schematic are thick for emphasis. **A** In IPTG-LacI, hydrogen bond formation at the ligand-binding pocket periphery promotes interactions between the N- and C-terminal subdomains of the core, which causes small structural rearrangements in the N-terminal subdomains (arrows) to increase flexibility in the hinge helix (black X). **B** In ONPF-LacI, the C-terminal core subdomain is not extensively perturbed as compared to DNA-LacI. However, N-terminal core subdomain loops become more structured, which may stabilize the interface of the core domain with the DNA-binding domain to bind the operator.

Figure 5A illustrates the key differences in the mutually incompatible conformational ensembles of the core domains in the IPTG-LacI and DNA-LacI states. The main features of IPTG-LacI, compared to DNA-LacI, are: rigidification of specific secondary structure elements near the ligand binding pocket (teal); increased specific inter-subdomain and inter-subunit interactions in the periphery of the ligand binding site (orange lines), some of which are supported by hydrogen bonding of protein and ligand atoms with structural water molecules (red circles and lines); and increased flexibility of other secondary structures throughout the C-terminal subdomain and in the N-terminal subdomain adjacent to the DNA-binding domain (purple). Differences in the inter-subunit interface likely reflect, at least partly, changes in the oligomeric state (our DNA-bound LacI states are dimeric, whereas the IPTG-LacI state is in a monomer-dimer equilibrium). Nevertheless, in contrast to IPTG-LacI, DNA-LacI is characterized by increased rigidification in both core subdomains away from the inter-subunit interface and ligand-binding site and at the interface with the DNA binding domain. We propose that IPTG binding rigidifies the N-terminal end of helix 2 and the inter-subdomain contacts at the expense of the rigidity of neighboring helices. These shifts in the conformational ensemble in the core domain are concurrent with changes in the DNA-binding domain: increased flexibility in the minor-groove binding hinge helices and major-groove binding two N-terminal helices. Together, these structural effects limit the binding affinity of LacI for the DNA operator.

Figure 5B shows the key differences in the conformational ensembles of the core domains of ONPF-LacI and DNA-LacI. The main feature of ONPF-LacI is increased rigidity (teal) in loops in the N-terminal subdomain of the core distal from the ligand binding site. We propose that because ONPF binding does not increase the flexibility of key secondary structure elements in the core domain to the same extent as inducer binding, ONPF binding is not incompatible with the DNA-bound conformational ensemble. The main functional consequence of ONPF binding may be that the binding interface of the core domain with the DNA-binding domain is preorganized in ONPF-LacI as compared to apo-LacI, leading to an increase in the binding affinity of LacI for the DNA operator.

In contrast to other methods for discovering allosteric mechanisms underlying protein behavior, our approach allows for the direct measurement of changes in the flexibility of secondary structure elements in response to perturbations. In general, observing the effects of mutations on the function of a protein is a powerful strategy for understanding protein allostery. However, the effects of distal mutations are difficult to predict for proteins like LacI, which function via long-range allostery across domains. Our approach offers a generalizable method to map sets of localized structural changes from perturbations to different functional states for a detailed description of allostery in a protein, without targeted mutations that require prior hypotheses on allosteric sites. These data present HDX/MS of a functional LacI; previously, HDX/nuclear magnetic resonance (NMR) spectroscopy studies have been carried out on the isolated DNA-binding domain^27,47,65^.

Engineering allosteric regulation in proteins is challenging if allosteric mechanisms are incompletely understood. Our study provides mechanistic understanding of differences in conformational ensembles that can inform efforts to reengineer natural allosteric proteins to respond to user-defined perturbations. Transcription factors regulate gene expression through two types of binding interactions: binding to a specific DNA sequence, and binding to a set of small molecule or protein partners that control DNA binding. Domain-swapping^44^, chimeric fusions^66^, directed evolution^37^, saturation mutagenesis^19^, and deep mutational scanning^43^ have resulted in LacI variants that control gene transcription in unexpected and interesting ways that depart from WT behavior, or in response to other sugars. However, none of these approaches are predictive with respect to tuning ligand response or allostery. Our strategy for computationally predicting interactions between protein, ligand, and water molecules and observing changes in conformational flexibility can help pinpoint structural changes that govern allosteric behavior in transcription factors and other proteins towards predictively and precisely designing the genotype-phenotype landscape.

## Methods

### Cell culture fluorescence assay

The *E. coli* DH10B Δ*lacI*:220 strain was chemically transformed with a plasmid encoding superfolder GFP (sfGFP) under the control of the pLlacO promoter and a plasmid encoding the WT LacI (pEZ22G-GFP-kan and pSc101-lacI-specR, gifts from the Raman lab at U. Wisconsin)^67^ and plated on Lysogeny broth (LB) agar with 50 µl/ml kanamycin and 50 µl/ml spectinomycin. A culture was grown overnight in LB medium with antibiotics at the same concentrations from a single colony. New 5 ml cultures were grown at 37 °C and shaking at 225 rpm for 16 h in blocks by subculturing the overnight culture using a 1:100 dilution in fresh LB medium. IPTG, TMG, and ONPF (Sigma-Aldrich, St. Louis, MO) were added to separate cultures to final concentrations of 100 µM, 100 µM, and 3 mM, respectively, at the two hour mark. Fully grown cultures were spun down at 3000 rpm for ten minutes and the supernatant was decanted. Pellets were washed two times in 5 ml 1× phosphate-buffered saline (PBS), resuspended in 5 ml 1× PBS, and diluted 1:20 in 200 µl PBS in a clear-bottom black 96 well microtiter plate (Costar). Fluorescence from sfGFP expression was read from 7 mm above the plate from all sample wells using an excitation wavelength of 485 nm and an emission wavelength of 528 nm. All fluorescence readings were normalized by culture growth, measured by absorbance at 600 nm.

### Protein expression and purification

A BL21(AI) Δ*lacI*-ZYA strain was generated by the method of Datsenko and Wanner^68^ and BL21(AI) Δ*lacI*-ZYA cells were transformed with the pET9a-6×His-LacI(1-331) vector. This construct encodes for dimeric LacI, without the tetramerization domain. All HDX/MS experiments were conducted with dimeric LacI. Cells were grown at 37 °C while shaking at 225 rpm for 12-16 h overnight in LB medium with 50 µg/ml kanamycin (LB-kan). The overnight culture was subcultured at a 1:50 dilution in LB-kan with 0.2% glucose and grown at 37 °C while shaking at 225 rpm for two hours, until the OD600 value of the culture was between 0.4 and 0.5. The temperature was then decreased to 16 °C, protein expression was induced by the addition of 0.2% arabinose, and cells were grown for an additional 16 hours. The culture was centrifuged at 6000 × *g* for 20 min at 4 °C and the pellet was frozen at −80 °C for at least two hours. The pellet was then thawed on ice for 15 min and resuspended in 40 ml lysis buffer (50 mM Tris, 150 mM NaCl, pH 8.0, with 5 mM MgCl_2_, 1 mM MnCl_2_, 100 µM CaCl_2_, a dissolved Pierce™ protease inhibitor cocktail tablet (Thermo Fisher Scientific, Waltham, MA), and 40 µg DNaseI (Thermo Fisher Scientific)) by gentle vortexing for 20 min. The resuspended cells were lysed by two passes of microfluidization. The lysed cells solution was centrifuged at 27,000 × *g* for 30 min at 4 °C. The soluble cell fraction was decanted, supplemented with 20 mM imidazole, stored on ice for one hour, centrifuged at 3270 × *g* for 10 minutes at 4 °C, and decanted again. We added 2 ml Ni^2+^-NTA slurry (HisPur Ni-NTA resin, Thermo Fisher Scientific) in binding buffer (50 mM Tris, 150 mM NaCl, 20 mM imidazole, pH 8.0) to the soluble cell fraction and nutated the protein-resin solution at 4 °C for one hour. The protein-resin solution was then centrifuged at 500 × *g* for five minutes at 4 °C and the supernatant was removed. The pelleted resin was resuspended in 2 ml binding buffer and applied to a 20 ml chromatography column. The resin was washed in the column with 50 ml binding buffer, then the protein was eluted in 14 ml elution buffer (50 mM Tris, 150 mM NaCl, 250 mM imidazole, pH 8.0). The eluted protein solution was spin-concentrated to a volume of 1.5 ml using Amicon™ Ultra Centrifugal Filter Units (MilliporeSigma, Burlington, MA) with a 10,000 Da molecular weight cutoff (MWCO). A volume of 3 µl from a 2 mg/ml stock solution of tobacco etch virus (TEV) protease with an N-terminal 6× histidine tag and 0.5 mM tris(2-carboxyethyl)phosphine (TCEP) was added to the concentrated protein solution. The reaction was incubated at 30 °C for two hours. Then, we diluted the reaction to 3 ml in LacI in dialysis buffer (50 mM MOPS, 200 mM NaCl, 0.5 mM TCEP, pH 7.0) and dialyzed the reaction in dialysis buffer at 4 °C while stirring, using a dialysis cassette with a 3000 Da MWCO (Slide-A-Lyzer, Thermo Fisher Scientific). The dialysis buffer was changed in 1 L increments of ~2, 16, and 2 h. The dialyzed protein solution was diluted to 17 ml in dialysis buffer with 20 mM imidazole and supplemented with 2 ml Ni^2+^-NTA slurry that had been pre-equilibrated in dialysis buffer, nutated at 4 °C for one hour, and then applied to a 20 ml chromatography column. The flowthrough containing purified LacI was collected. An additional 5 ml dialysis buffer was applied to the column and the flowthrough was also collected. The flowthrough fractions were combined and spin-concentrated to a final concentration of 10 µM. A small volume of the final LacI sample was run on a denaturing SDS-PAGE gel to confirm the protein size and purity (>95%). The oligomerization state was confirmed by size-exclusion chromatography and protein secondary structure assessed by circular dichroism.

### DNA operator annealing

Biotinylated operator DNA sequences were ordered as complementary single-stranded primers from IDT DNA, diluted to equimolar concentrations, and annealed at 95 °C for two minutes. Annealed primers were allowed to come room temperature. Only the 5’ forward sequences were biotinylated. The forward sequences of the operators are:

Synthetic perfectly symmetric operator (O_syn_ used in BLI experiment)^59^

AGTAGTCTAGAATTGTGAGCGCTCACAATTCTAGAGTAGT

LacO1 operator (O_lac_, which was also used in the HDX/MS experiments)

AGTAGTggAATTGTGAGCGGATAACAATTGACATTGTGAGCGGATAACAAGATACTGAGCACATCAGC

### Determination of operator binding by LacI using bio-layer interferometry (BLI)

Binding measurements were carried out at room temperature using an Octet RED96 system and streptavidin-coated biosensor tips (Pall ForteBio). The biotinylated operator DNAs were diluted to 200 nM in phosphate-buffered saline (PBS) with 0.2% bovine serum albumin (BSA) and 0.05% Tween-20 (PBS-T), pH 8.0, to be used as the antigen. Antigen-bound streptavidin tips were washed in PBS-T, pH 8.0 and dipped in LacI solutions in microplate wells with concentrations ranging from 0 to 40 nM in the same buffer during an association period. Tips were then returned to the washing well during a dissociation period. Experiments were carried out in technical triplicate, duplicate, and singlicate for −IPTG, lacO1 (natural operator), −IPTG, synthetic operator, and +IPTG, lacO1 conditions, respectively, at 10 LacI concentrations. Raw data were fit to 1:1 binding curves in Octet Data Analysis HT software version 10.0 using curve-fitting kinetic analysis with global fitting.

### MS protocol and data acquisition

Acetonitrile, formic acid (1 mL ampules, Fisher Optima grade, 99.9%), and MS-grade water (Thermo Fisher Scientific) were used to prepare mobile phase solvents. Solvent A was 99.9% water/0.1% formic acid and solvent B was 90% acetonitrile/10% water with 0.1% formic acid added (v/v). The elution program consisted of isocratic flow at 10% B for 4 min, a linear gradient to 45% B over 9 min, isocratic flow at 100% B for one min, isocratic flow at 10% B for one min, a linear gradient to 45% B over one min, isocratic flow at 100% B for one min, and then isocratic flow at 10% B for 3 min, at a flow rate of 20 mL/min. Full-scan mass spectra were acquired in the positive ion mode over the range *m/z* = 400–2000 using the Orbitrap mass analyzer, with a mass resolution setting of 70,000, AGC target of 1.00e6, and maximum IT set to 50 s. We also ran three tandem mass spectrometry (MS/MS) experiments for each sample with the same full MS settings as described above, and dd-MS2 settings as follows: resolution 17,500, AGC target 1e5, maximum IT 100 ms, loop count 6, isolation window 2.0 *m/z*, NCE 28, charge state 1 and ≥7 excluded, dynamic exclusion of 20 s. Data acquisition was controlled using Xcalibur software (version 4.1, Thermo Fisher Scientific).

### Sample preparation for HDX/MS

Exchange buffer (50 mM MOPS, 200 mM NaCl, 0.5 mM TCEP, pH 7.0) was prepared in deionized water. To make a D_2_O exchange buffer, the H_2_O exchange buffer was lyophilized and resuspended in the same volume of D_2_O three times. Dimeric LacI (residues 1–331) was expressed and purified as described. LacI samples were prepared at 10 µM in 100 µl H_2_O exchange buffer, with a small molecule ligand, DNA operator, or both added to separate protein samples at the following concentrations: IPTG, 10 mM; ONPF, 3 mM or 14.6 mM; TMG, 150 µM; DNA operator, 25 µM. For the 14.6 mM ONPF-LacI condition, we added 14.6 mM ONPF in the exchange buffer. (The ternary IPTG-DNA-LacI complex data were collected similarly, but with small differences in sample preparation and using different HDX/MS equipment. See Methods section “HDX/MS sample preparation, experimental protocol, and data analysis for the DNA, IPTG, and LacI ternary complex experiments shown in Supplementary Figs. 10, 11.”) The DNA was a 68 base pair long sequence with two LacO1 sites separated by a 6 base pair linker, ordered from IDT DNA (Coralville, IA) as complementary primers and annealed as described in the Methods section “DNA operator annealing.” Samples were equilibrated at 4 °C with binding partners for at least 30 minutes before the HDX/MS experiment. Fully deuterated samples were prepared at the same concentrations in D_2_O buffer with 8 M urea, diluted in D_2_O exchange buffer 1:10, incubated at room temperature for 16 h, and lyophilized two times for at least 16 h, adding back an equal volume of D_2_O each time. Then the fully deuterated samples were incubated at room temperature for an additional 24 h. Fully deuterated experiments were conducted for all samples except for those with 14.6 mM ONPF in the sample buffer.

### HDX/MS experimental protocol

For all experiments except the IPTG-DNA-LacI ternary complex experiments (see next section, “HDX/MS sample preparation, experimental protocol, and data analysis for the DNA, IPTG, and LacI ternary complex experiments shown in Supplementary Figs. 10 and 11.”), HDX labeling was performed in solution using a LEAP technologies HDX PAL robot (Carrboro, NC), with a temperature-controlled sample vials and buffer holder held at 4 °C and refrigerated compartments held at 1 °C. Samples were injected on a Thermo UltiMate 3000 LC connected in-line with an Q Exactive mass spectrometer equipped with an electrospray ionization source (Thermo Fisher Scientific). The samples were diluted 1:10 in the D_2_O exchange buffer (50 mM MOPS, 200 mM NaCl, 0.5 mM TCEP, pH 7.0) to allow for hydrogen atoms in the protein backbone amide groups to exchange for deuterons in the buffer. Aliquots of the diluted samples were collected at nine timepoints (0 s, 30 s, 45 s, 1 min, 5 min, 25 min, 1 h, 2 h, 4 h) and quenched 1:1 in an acidic quench buffer, cooled to 1 °C to slow the HDX reaction (6 M urea, 200 mM arginine, 100 mM TCEP, pH 2.0). This range of timepoints was chosen to allow for the measurement of a large range of exchange rates events in all functional states of LacI. Each sample was then enzymatically proteolyzed on a Waters Enzymate™ BEH Pepsin Column (30 mm length × 2.1 mm diameter, 5 μm particle size, Waters Corporation, Milford, MA), or a combination of pepsin and fungal protease type XIII (manually conjugated to POROS™ 20 AL Aldehyde Activated Resin, Thermo Fisher Scientific, and packed in a 2 mm ID × 2 cm IDEX C-130B column, Fisher Scientific, Hampton, NH), loaded on a C18 analytical column (Hypersil Gold, 10 mm length × 2.1 mm diameter, 3 µm particle size, Thermo Fisher Scientific) with a guard column attached to the inlet (Thermo Fisher Scientific), and injected in a mass spectrometer using a 100 µL sample loop (Thermo Q Exactive) in solvent A (defined below). Desalting in sample A occurred over 2 min at 300 µl/min. Five independently generated exchange reactions (technical replicates) for all states were performed from four separately expressed and purified LacI samples (biological replicates) to ensure adequate technical and biological replicates, except for the TMG state, for which two biological replicates were performed. Back exchange was calculated on a peptide basis by conducting an additional HDX/MS experiment on an apo LacI “maximally labeled” or “fully deuterated” control sample. The average deuteration for all fully deuterated peptides over the whole protein was 70% with a standard deviation of 15.7%, and 11.4% of fully deuterated peptides had 50% deuteration or less (44 out of 386 total peptides). In the curated set of 57 core domain peptides included in this study, the average deuteration for fully deuterated peptides was 70.5% with a standard deviation of 14.6% (Supplementary Table 2). Further mass spectrometry details and full data analysis methods are available in the Methods section, “HDX/MS data analysis.” Calculated fit parameters, uncertainties of the parameters, and raw centroid data for all replicates are available in Supplementary Datasets 1–3, respectively.

*HDX/MS sample preparation, experimental protocol, and data analysis for the DNA, IPTG, and LacI ternary complex experiments shown in* Supplementary Figs. 10 and 11

Sample preparation: exchange buffer (50 mM Tris, 150 mM NaCl, pH 8.0) was prepared in deionized water. To make a D_2_O exchange buffer, the H_2_O exchange buffer was lyophilized and resuspended twice in the same volume of D_2_O. LacI samples were prepared at 10 μM in 200 μl H_2_O exchange buffer, with IPTG, DNA operator, or both added to separate protein samples at the following concentrations: IPTG, 200 mM; DNA operator, 25 μM. These concentrations were chosen to be saturating while maintaining solubility. Samples were equilibrated at 4 °C with binding partners for at least 30 min before the HDX/MS experiment.

HDX/MS protocol: all HDX labeling was performed in solution using a LEAP technologies HDX PAL robot (Carrboro, NC), with a temperature-controlled sample vials and buffer holder held at 2.5 °C, a labeling reaction sample tray held at 15 °C, and protease digestion compartment held at 7 °C. Samples were loaded and digested using a MX class reciprocating pump (Teledyne SSI) and eluted using an Agilent 1290 Infinity II binary pump in line with a Bruker maXis II mass spectrometer with ESI-qTOF. The samples were diluted 1:10 in the D_2_O exchange buffer (50 mM Tris, 150 mM NaCl, pH 8.0) to allow for hydrogen atoms in the protein backbone amide groups to exchange for deuterons in the buffer. Aliquots of the diluted samples were collected at timepoints (0 s, 30 s, 1000 s, and 14,400 s for the DNA-LacI state, with an additional 300 s timepoint for the IPTG-LacI and IPTG-DNA-LacI states), quenched 1:1 in an acidic quench buffer (3 M guanidine hydrochloride, 1.5% (v/v) formic acid, 3% (v/v) acetonitrile, pH 2.0), and cooled to 4 °C to slow the HDX reaction. This range of timepoints was chosen to allow for the measurement of fast and slow exchange events in all functional states of LacI. Each sample was then enzymatically proteolyzed on a combination of pepsin and fungal protease type XIII (Immobilized protease type XIII/pepsin column (w/w, 1:1) 30 mm length × 2.1 mm diameter, NovaBioAssays), loaded on a trap column (EXP, 5 mm length × 1 mm diameter, 3.0 μm packing material, C8 phase, Optimize Technologies), loaded on a C18 analytical column (Hypersil Gold, 50 mm length × 1 mm diameter, 1.9 μm particle size, Thermo Fisher Scientific) with a guard column attached to the inlet (Thermo Fisher Scientific), and injected in the mass spectrometer using a 250 μL sample loop in solvent A (defined below). Desalting in solvent A occurred over 3 min at 150 μl/min. Acetonitrile, formic acid and MS-grade water (Fisher Optima LC/MS) were used to prepare mobile phase solvents. Solvent A was 3% acetonitrile/97% water with 0.15% formic acid added (v/v) and solvent B was 97% acetonitrile/3% water with 0.15% formic acid. The elution program consisted of a linear gradient from 2 to 10% solvent B over 1 min, a linear gradient from 10 to 45% B over 11 min, a linear gradient from 45 to 95% B over 2 min, isocratic flow at 95% B for 4 min, a linear gradient from 95 to 2% B over 1 min, isocratic flow at 2% B for 4 min, a linear gradient from 2 to 95% B over 1 min, isocratic flow at 95% B for 4 min, a linear gradient from 95 to 2% B over 1 min, and isocratic flow at 2% B for 13 min. This was conducted at a flow rate of 40 µl/min. Full-scan mass spectra were acquired in the positive ion mode over the range *m/z* = 100–2250 using the TOF mass analyzer, with a spectra rate of 1.00 Hz. We also ran one tandem mass spectrometry (MS/MS) experiment for each sample with the same full MS settings as described above except with spectra rate 1.2 Hz, and dd-MS2 settings as follows: 12 precursor ions, active exclusion after 3 spectra with release after 0.2 min, charge states >7 excluded. Data acquisition was controlled using Bruker Compass HyStar software.

HDX/MS data analysis: for each sample, peptides were identified using combined MS/MS raw data by searching against the LacI amino acid sequence (UniProtKB, www.uniprot.org, accessed 6/18/2019) using BioTools software (version 3.2, build 9.10, Bruker Daltonics). The digest parameters used in the search were: pepsin cut sites, 15 partials, mass range 400–6000, precursor mass tolerance, 7 ppm; and fragment mass tolerance, 0.02 Da. In a typical sample MS/MS, 245 unique forward peptides were found; 1413 spectra were matched to forward peptides; coverage was 93.9% of the 330-amino acid LacI sequence, intensity coverage was 17.3%, and stringent sequence coverage was 72.5%. The exported peptide lists (sequence, charge state, and retention time) from separate samples were imported into HD Examiner 3.0 (Sierra Analytics, Modesto, CA) and combined. All peptide isotope distributions at each exchange timepoint were fit in HD Examiner and manually checked for correct assignments. For each peptide in each functional state, deuteration levels at each timepoint were calculated by subtracting mass centroids of undeuterated peptides (*m*_0_) from mass centroids of deuterated peptides (*m*_t_), and multiplying by the theoretical maximum number of exchangeable protons (max_protons). For each peptide, all charge states with high quality data as determined by HD Examiner were averaged, and the average used. Uptake plots were constructed by graphing the calculated deuteration levels against time for each peptide, with the maximum number of exchangeable protons as the limit on the y axis and the error bars showing the standard deviation of *n* = 2–5 replicates. All calculations for fractional exchange were performed using averaged centroid data at each timepoint. The uptake data were fit to a nonlinear regression model and the fractional differences in H-D exchange were calculated as described in the previous section (“HDX/MS data analysis”).

### HDX/MS data analysis

For each sample, peptides were identified using combined MS/MS raw data by searching against the LacI amino acid sequence (UniProtKB, www.uniprot.org, accessed 6/18/2019) and the pepsin amino acid sequence using Proteome Discoverer software (version 1.3, SEQUEST algorithm, Thermo Fisher Scientific) or Byonic software (Protein Metrics, Cupertino, CA). The protein search configuration used in the search were: cleavage residues, FWYLI;FWYLI; digest cutter, N-terminal and C-terminal cutter; and spectrum-level FDR, autocut. In a typical sample MS/MS, 476 unique forward peptides were found; 1027 spectra were matched to forward peptides; coverage was 98.18% of the 330-amino acid dimeric LacI sequence; and estimated spectrum-level false-discovery rate (FDR) on true proteins was 0.0%. The following filters were applied when identifying peptides: minimum precursor mass, 350 Da; maximum precursor mass, 5000 Da; minimum peak count, 1; maximum collision energy, 1000; S/N threshold, 1.5; minimum peptide length, 4; maximum peptide length, 144; maximum number of peptides reported, 10; precursor mass tolerance, 10 ppm; and fragment mass tolerance, 0.02 Da. The exported peptide lists (sequence, charge state, and retention time) from separate samples were imported into HD Examiner 2.0 (Sierra Analytics, Modesto, CA) and combined. All peptide isotope distributions at each exchange timepoint were fit in HD Examiner and manually checked for correct assignments. For each peptide in each functional state, deuteration levels at each timepoint were calculated by subtracting mass centroids of undeuterated peptides (*m*_*0*_) from mass centroids of deuterated peptides (*m*_*t*_), dividing by the difference between the mass centroid of the fully deuterated peptide (*m*_*∞*_) and the undeuterated peptide, and multiplying by the theoretical maximum number of exchangeable protons (*max_protons*):

1 $$D(t)={{{{{\rm{max }}}}}}\_{{{{{\rm{protons}}}}}}\times \frac{{m}_{t}-{m}_{0}}{{m}_{{{\infty }}}-{m}_{0}}$$

For each peptide, the charge state with the highest quality data as determined by HD Examiner was used. We note that the fully deuterated sample was in 100% D exchange buffer, while the experimental samples at each timepoint were in 90% D exchange buffer after 1:10 dilution, which would result in an increase in the NE and C parameters (described below).

Uptake plots were constructed by graphing the calculated deuteration levels against time for each peptide, with the maximum number of exchangeable protons as the limit on the *y* axis and the error bars showing the standard deviation of *n* = 2–5 replicates. The uptake data were fit to a nonlinear regression model using the SciPy optimize.curve_fit function. All calculations for fractional exchange as shown in Fig. 2B and Fig. S7 were performed using averaged centroid data at each timepoint, not the fitting functions; the nonlinear regression function fits are shown as lines in uptake plots to guide the eye. The nonlinear regression function was:

2 $$D(t)={{{{{\rm{max }}}}}}\_{{{{{\rm{protons}}}}}}-A\times {e}^{-{k}_{1}x}-B\times {e}^{-{k}_{2}x}-C\times {e}^{-{k}_{3}x}-{NE}$$

where

$${{{{{\rm{max }}}}}}\_{{{{{\rm{protons}}}}}}=A+B+C+{NE}-2-{{{{{\rm{num}}}}}}\_{{{{{\rm{prolines}}}}}}$$

$${k}_{1} \, > \, {k}_{2}$$

$${k}_{2} \, > \, {k}_{3}$$

$${k}_{1} \, > \, {k}_{3}$$

*D(t)*: number of deuterons exchanged for protons as a function of time

max_protons: theoretical maximum number of exchangeable protons for the peptide

*A, B, C*: number of fast-, medium-, and slow-exchanging protons, respectively

*k*_*1*_*, k*_*2*_*, k*_*3*_: rates of exchange for fast-, medium-, and slow- exchanging protons, respectively

*NE*: number of non-exchanging protons

num_prolines: number of prolines in the peptide

For slow-exchanging peptides in which the last timepoint had an experimentally observed value of <0.5 D, data were fit to a modified regression function with A = 0 and k_1_ = 0.

The fractional difference in H-D exchange at all timepoints and the associated uncertainty were calculated as described previously using averaged centroid data^69^. Supplementary Dataset 1 contains the calculated parameters for H-D exchange fitting to experimental data points for all peptides, corresponding to each line for the uptake plots in Supplementary Fig. 4. Supplementary Dataset 2 contains the calculated uncertainties for the differential exchange plots shown in Supplementary Fig. 9. Supplementary Dataset 3 contains raw MS centroids for replicates at each timepoint for each peptide and functional state. Supplementary Table 3 contains summary information for all HDX/MS experiments performed in this study.

### Computational prediction of structural water molecules

To predict the positions of structural water molecules, we applied a semi-explicit solvation method implemented in the macromolecular modeling program Rosetta^63^ on each pre-relaxed LacI crystal structure (PDB 2PAF (ONPF-bound LacI core domain), 2P9H (IPTG-bound LacI core domain), 1LBI (apo LacI core domain with tetramerization helix) using the beta_nov16 energy function. Predicted structural water molecules within a 6 Å shell of any atom of the ligand were retained. All hydrogen bonds within a heavy-atom distance of 3.2 Å were calculated between water oxygen atoms and protein or ligand residues. The probability of hydrogen bonding was calculated as the number of times that a residue could form a hydrogen bond with a predicted structural water molecule out of 100 total output structures. See the Supplementary computational methods for the detailed protocol, scripts, and command lines.

### Reporting summary

Further information on research design is available in the Nature Portfolio Reporting Summary linked to this article.

## Supplementary information

Supplementary Information

Description of Additional Supplementary Files

Supplementary Data 1

Supplementary Data 2

Supplementary Data 3

Reporting Summary

## Data Availability

All data generated in this study are provided in the Supplementary information, Supplementary files, and Source File. The raw MS data have been deposited in the Zenodo database under accession code 10.5281/zenodo.7585854 (raw MS files on Zenodo)^70^. PDB codes used in this study include 1LBI (apo LacI core domain with tetramerization helix), 2P9H (IPTG-bound LacI core domain), 2PAF (ONPF-bound LacI core domain), and 1EFA (ONPF-bound LacI without the tetramerization helix). Source data are provided with this paper.

## Source data

Source data

## Code availability

All code is available on https://github.com/anumazam/watermaps_analysis_scripts. 10.5281/zenodo.7577312^71^. The Rosetta software suite can be downloaded from the Rosetta Commons website [https://www.rosettacommons.org/software/license-and-download] (Rosetta Commons website).
