# Author Correction: Ligand-specific changes in conformational flexibility mediate long-range allostery in the *lac* repressor

**DOI:** 10.1038/s41467-023-39918-z

**Published:** 2023-07-14

**Authors:** Anum Glasgow, Helen T. Hobbs, Zion R. Perry, Malcolm L. Wells, Susan Marqusee, Tanja Kortemme

**Affiliations:** 1 Department of Bioengineering and Therapeutic Sciences, University of California, San Francisco, CA 94158 USA; 2 Department of Biochemistry and Molecular Biophysics, Columbia University, New York, NY 10032 USA; 3 Department of Chemistry, University of California, Berkeley, Berkeley, CA 94720 USA; 4 Department of Molecular Biophysics and Biochemistry, Yale University, New Haven, CT 06511 USA; 5 Department of Physics, Columbia University, New York, NY 10032 USA; 6 Department of Molecular & Cell Biology, University of California, Berkeley, Berkeley, CA 94720 USA

**Keywords:** Molecular conformation, Biophysical chemistry, Mass spectrometry, DNA-binding proteins

Correction to: *Nature Communications* 10.1038/s41467-023-36798-1, published online 02 March 2023

The original version of this Article contained an error in Fig. 2a, in which the graph in panel i (for peptide ALHAP) was duplicated in panel v. This has been corrected in both the PDF and HTML versions of the Article.

