# Supplementary material for: Ligand-specific changes in conformational flexibility mediate long-range allostery in the lac repressor
Source: Nat Commun. 2023 Mar 2;14:1179. doi: 10.1038/s41467-023-36798-1 (PMC9977783; doi:10.1038/s41467-023-36798-1)
Supplement: Supplementary file 1 — Supplementary Information [file 41467_2023_36798_MOESM1_ESM.pdf]

Supplementary information for

Ligand-specific changes in conformational flexibility mediate long-range allostery in the *lac* repressor

Supplementary Discussion: mutational analysis

Comparing the effects of specific mutations from previous biochemistry studies with our HDX/MS dataset puts the functional effects of these mutations in a structural context. We focused on mutations in the monomer-monomer interface and in the ligand binding pocket.

At the monomer-monomer interface in helix 2, mutations at A81 and K84 (Supplementary Figure 9A) changed the IPTG-LacI and DNA-LacI binding affinities, as well as the protein stability[1–5]. One hypothesis informed by crystal structures is that an inter-subunit, inter-subdomain salt bridge between H74 in helix 2 and D278 in helix 17 of the other subunit (Supplementary Figure 9B) stabilizes the conformation of IPTG-LacI[6] although mutations to these residues do not necessarily disrupt IPTG binding[4]. Our H-D exchange data alongside these mutational data suggest that several quaternary contacts at the monomer-monomer interface in helices 2, 13, and 17, and beta strand 3, facilitate the IPTG-specific shifts in the conformational ensemble (Figures 2B, 2C, Supplementary Figure 5).

At the N-terminal subdomain monomer-monomer interface, mutations to residues 94-98 in beta strand 3 (Supplementary Figure 9C) have also previously been shown to disrupt LacI allostery[7]. This observation is consistent with our hypothesis that hydrogen bonds across the beta sheets in both subunits rigidify the inducer-bound states, which arises from the observation of decreased H-D exchange in beta strand 3 in IPTG-LacI as compared to DNA-LacI (Figure 2A, ii, and see also peptide 94 - VVVSM in Fig. S7, ΔHX_t DNA-TMG and ΔHX_t IPTG-DNA plots). Crystal structures additionally show a decrease in the distance between the beta strands, which corresponds to the formation of direct hydrogen bonds between V96 backbone atoms in the IPTG state, rather than water-mediated hydrogen bonds in the DNA state in which the sidechain of K84 interacts with the carbonyl oxygens of V94 and V96 (Supplementary Figure 6).

Finally, we considered residues in the binding pocket-peripheral loops that interact directly with IPTG, TMG, and ONPF. Mutagenesis to residues L148 and S151 (Supplementary Figure 9D) resulted in shifts in the binding affinities of LacI for both IPTG and the DNA operator[8]. In line with the shift in binding affinity for IPTG noted in these mutational studies, we observed attenuated H-D exchange in the pocket-peripheral loops for IPTG as compared to the weaker-affinity ligand TMG (Supplementary Figure 7, ΔHX_t IPTG-TMG plot, peptides 98 - MVERSGVE, 158 - SIIFSH, 147 - FLDVSDQTPINS and 185 - LAGPLSSVSARL)[9].

Supplementary computational methods: command lines, input files and scripts

We used Rosetta 3.9 (version 2019.19) to predict structural water molecule placement, as described in the Methods.

(1) Preparation of LacI structures for solvation

Relax command line:

```
~/Path/to/rosetta/source/bin/relax.linuxgccrelease -database ~/Path/to/rosetta//database
-s INPUT.pdb -nstruct 2 -constrain_relax_to_start_coords -coord_constrain_sidechains -
beta -ex1 -ex2 -use_input_sc -flip_HNQ -no_optH false -
optimization::default_max_cycles 200
```

(2) Semi-explicit solvation

Command line to solvate the relaxed LacI structure 2P9H using the beta_nov16 energy function and output 100 solvated structures:

```
~/Desktop/Rosetta/rosetta/main/source/bin/rosetta_scripts.macosclangrelease
@flags_NOWATERS.txt -parser:protocol solvate_NOpack.xml -database
~/Desktop/Rosetta/rosetta/main/database -in:file:s 2p9h_R_align_relax.pdb -out:suffix
_solvate
```

Example XML file implementing semi-explicit solvation[10] without packing:

```
<ROSETTASCRIPTS>
  <SCOREFXNS>
            <ScoreFunction name="beta" weights="beta_nov16"/>
      </SCOREFXNS>
      <TASKOPERATIONS>
            <PreventRepacking name="prevent_repacking" resnum="62A-330A,62B-
330B"/>
      </TASKOPERATIONS>
      <MOVERS>
            <PackRotamersMover
                  name="pack"
                  scorefxn="beta"
                  task_operations="prevent_repacking"/>
            <WaterBoxMover
                  name="solvate"
                  mode="replace"
                  gen_fixed="1"
                  scorefxn="beta"
                  task_operations="prevent_repacking"/>
      </MOVERS>
      <PROTOCOLS>
```

```
            <Add mover_name="solvate"/>
            <Add mover_name="pack"/>
      </PROTOCOLS>
</ROSETTASCRIPTS>
```

Example flags file to specify input file, packing parameters, energy function, and number of output structures:

```
-in
  -file
    -s 2p9h_R_r.pdb
-packing
  -ex1
  -ex1aro
  -extrachi_cutoff 0
  -ex2
-nstruct 100
-beta_nov16
-parser
  -view
-mute core.util.prof
-mute core.io.database
```

(3) Downstream analysis and WaterMap generation performed in Python and PyMOL.

All Python scripts are available on Github (https://github.com/anumazam/watermaps_analysis_scripts) with DOI: 10.5281/zenodo.7577312:

delete_waters.py
bin_waters.py
hbnets.py
run_hbnets.py
data2bfactor.py
color_b.py

Supplementary Figure 1. X-ray crystal structures of LacI. ONPF-DNA-LacI (PDB 1EFA, gray, resolution 2.6 Å)[11] and IPTG-LacI (PDB ID 2P9H, red, resolution 2.0 Å)[12] states are aligned on the C-terminal subdomain to show a small shift towards a more “pinched” conformation of the N-terminal subdomain of the core.

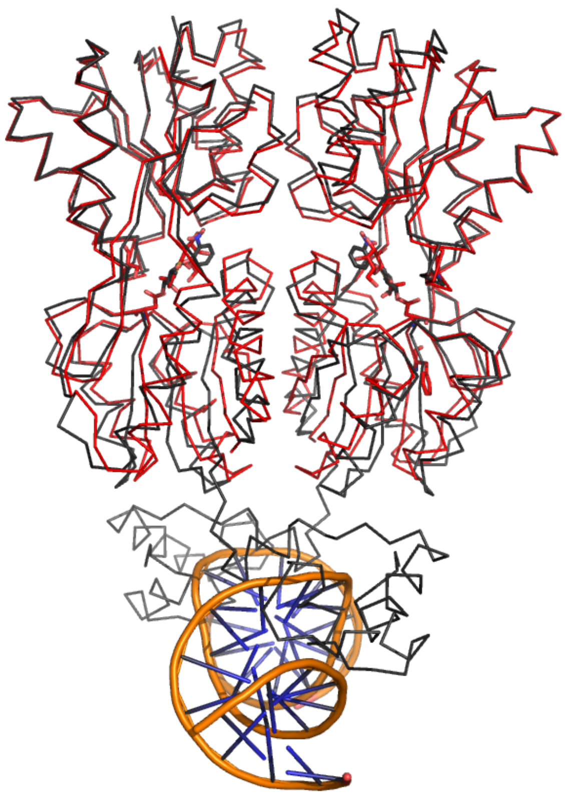

Supplementary Figure 2. LacI characterization. (A) Cell culture assay to measure effect of ligands on LacI regulation gene expression. In a two-plasmid system in ΔlacI *E. coli* (the same strain as described in "Cell culture fluorescence assay" in the Methods), LacI is expressed constitutively from one plasmid alongside a green fluorescent protein (GFP) that is encoded under the control of pLlacO1 on a pZE22G plasmid, in the presence of different ligands. GFP fluorescence is then plate-read. Individual points show *n = 3* biologically independent experiments. Samples are colored to match structures in Figures 1C, 1D. **(B)** Biolayer interferometry (BLI) assay to measure binding of operators to LacI in the presence and absence of 100 μM IPTG. Biotinylated operator sequences were associated to strep-tagged tips in microplate wells in the BLI experiment, and LacI was introduced as the analyte in solution in separate wells at concentrations of 0-40 nM. No LacI binding to the operator was detected upon IPTG addition. The perfectly symmetric LacO1 binds more tightly than the native operator to LacI, as was previously observed (1.1 and 9.5 nM), which correspond with published values (between 1 nM and 1-10 nM, respectively)[13–17]. O_{lac}: LacO1 operator. O_{syn}: synthetic symmetric operator. Experiments were carried out in technical triplicate, duplicate and singlicate on the same LacI sample for -IPTG, lacO1 (natural operator), -IPTG, synthetic operator, and +IPTG, lacO1 conditions, respectively, at 10 LacI concentrations. Data are presented as mean values +/- standard deviations. See Methods for sequences and full methods details.

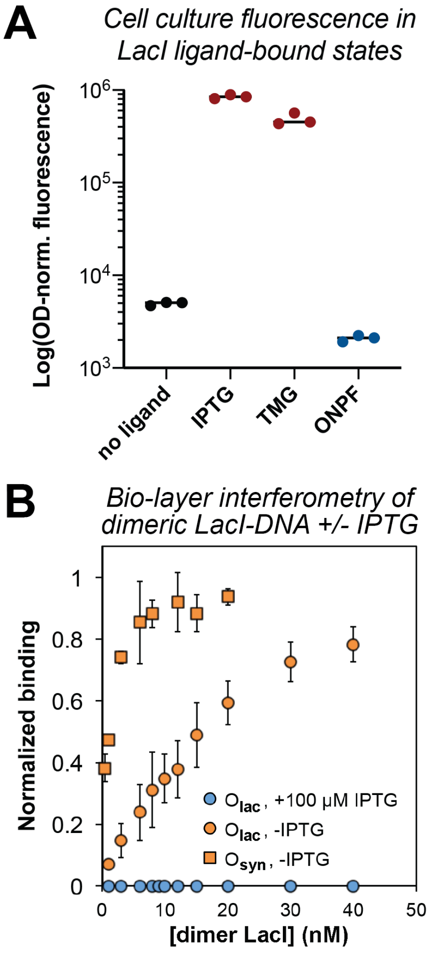

Supplementary Figure 3. Summary of HDX/MS peptide coverage for the curated set of peptides for the core domain of LacI. (A) Coverage map showing individual peptides (gray bars) by amino acid position (small squares) in the core domain (residues 63-330). Blue boxes mark every tenth residue in each row. Brown boxes mark residues that were not present in any of the peptides. The extreme C-terminal sequence of the core domain pertaining to beta strands 20-21 (as labeled in Figure 2) and the loop connecting them were not included in the dataset because of incomplete or noisy data for at least one of the functional states in this region of the protein. Excluding this sequence, HDX/MS coverage of the core domain was 97% with several overlapping peptides for some regions. **(B)** Structure of the core domain (PDB ID 2P9H) with brown residues corresponding to brown amino acid positions in (A). **(C)** Peptide statistics. The majority of peptides (65%) were 4 to 9 residues in length. Peptides were evenly distributed between the N- and C-terminal subdomains of the core.

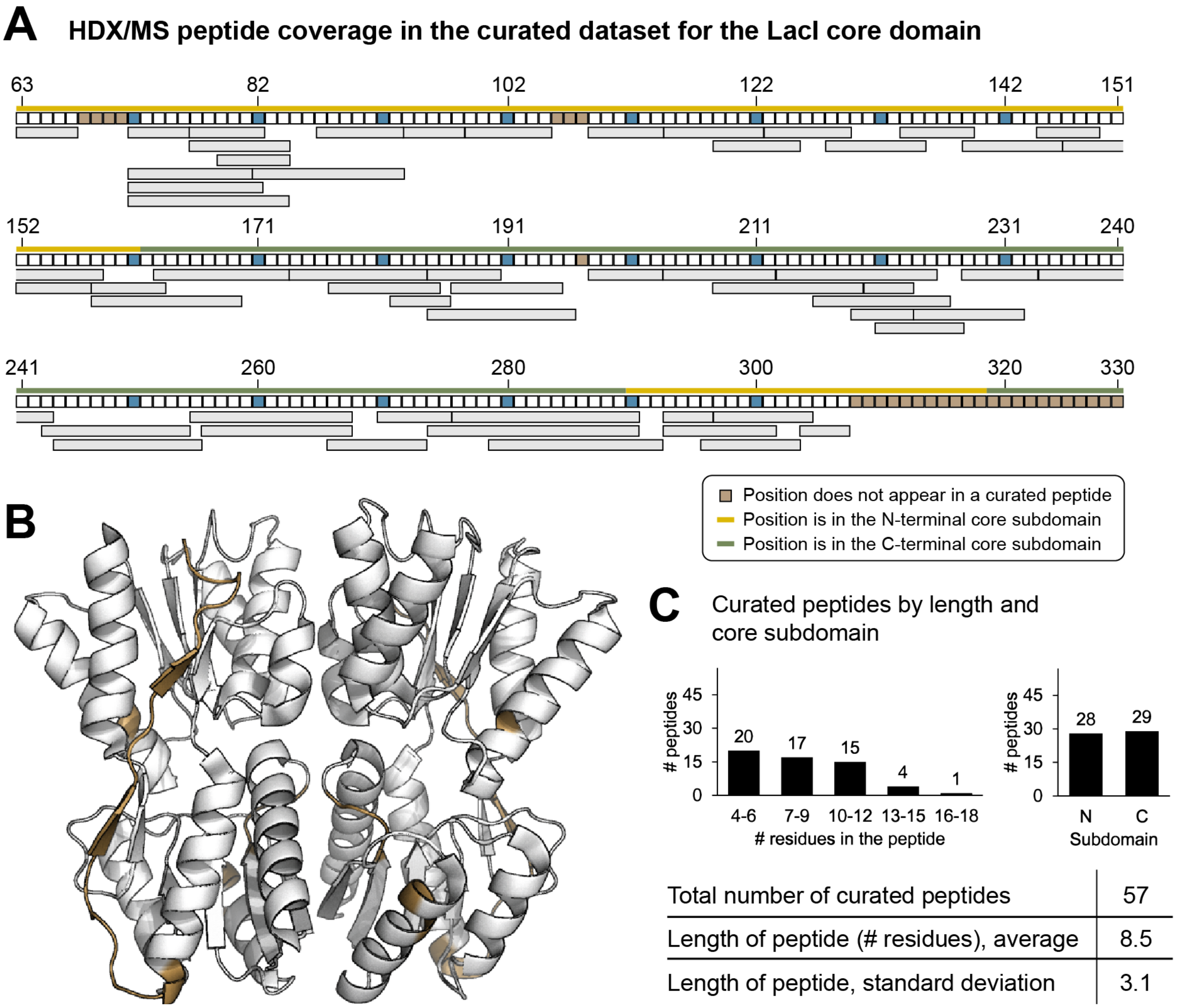

Total number of curated peptides	57
Length of peptide (# residues), average	8.5
Length of peptide, standard deviation	3.1

Supplementary Figure 4. Uptake plots for 57 LacI peptides in six functional states under identical conditions. All experiments shown here were performed with identical buffers and dilution protocols. TMG and ONPF were at sub-saturating concentrations due to solubility limitations (15 μM and 300 μM, respectively); data for the maximum ONPF concentration (14.6 mM), using altered buffers and dilution protocols, are shown in Supplementary Figures 14-17. Plots are organized from N- to C-terminus of LacI. The gray labels in the plot corners indicate the LacI secondary structure element(s) represented in the peptide, as in Figure 2.

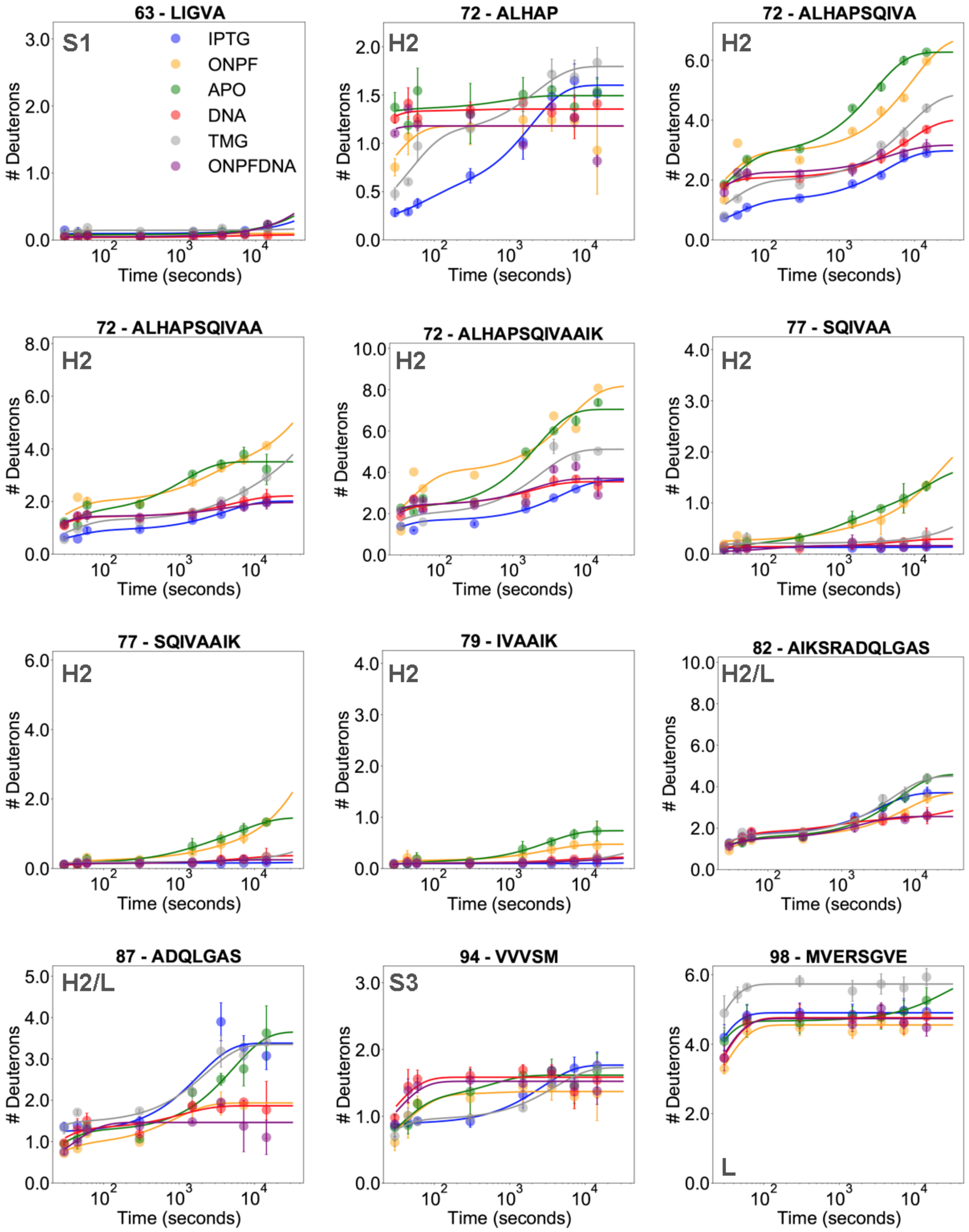
63 - LIGVA
S1
IPTG
ONPF
APO
DNA
TMG
ONPFDNA
72 - ALHAP
H2
72 - ALHAPSQIVA
H2
72 - ALHAPSQIVAA
H2
72 - ALHAPSQIVAAIK
H2
77 - SQIVAA
H2
77 - SQIVAAIK
H2
79 - IVAAIK
H2
82 - AIKSRADQLGAS
H2/L
87 - ADQLGAS
H2/L
94 - VVVSM
S3
98 - MVERSGVE
L
Deuterons
Time (seconds)

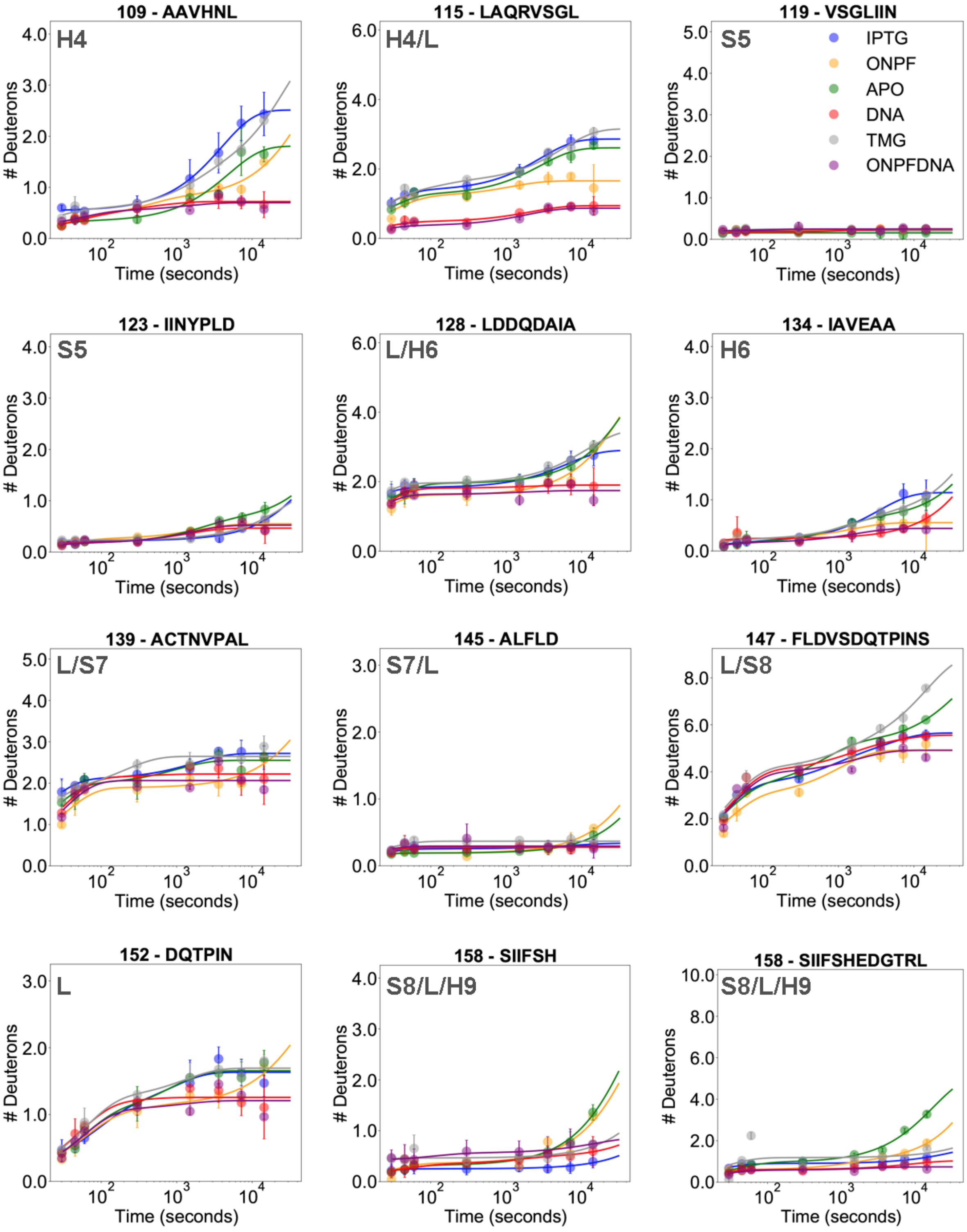

109 - AAVHNL
H4
115 - LAQRVSGL
H4/L
119 - VSGLIIN
S5
IPTG
ONPF
APO
DNA
TMG
ONPFDNA
123 - IINYPLD
S5
128 - LDDQDAIA
L/H6
134 - IAVEAA
H6
139 - ACTNVPAL
L/S7
145 - ALFLD
S7/L
147 - FLDVSDQTPINS
L/S8
152 - DQTPIN
L
158 - SIIFSH
S8/L/H9
158 - SIIFSHEDGTRL
S8/L/H9
Deuterons
Time (seconds)

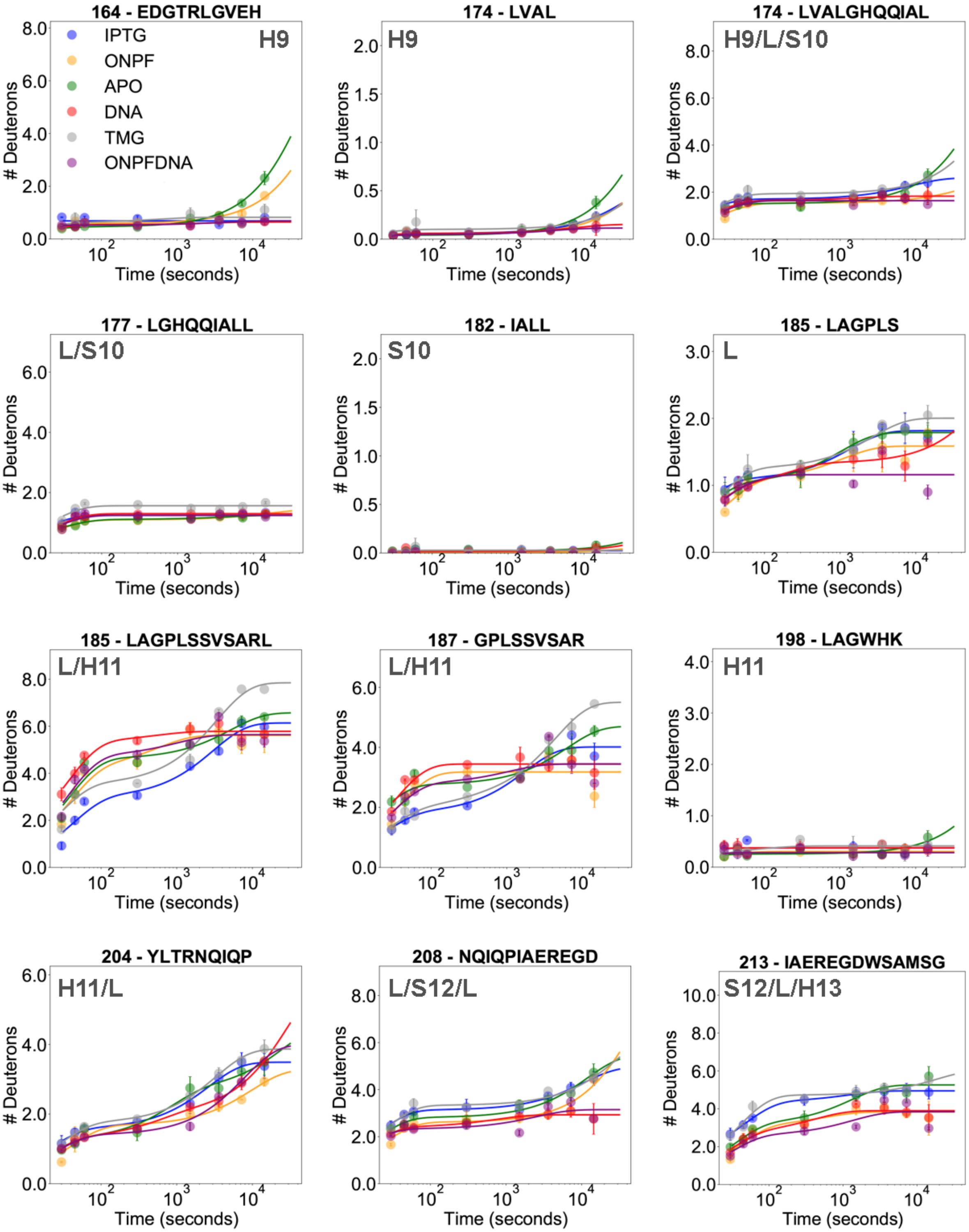
164 - EDGTRLGVEH
H9
IPTG
ONPF
APO
DNA
TMG
ONPFDNA
174 - LVAL
H9
174 - LVALGHQQIAL
H9/L/S10
177 - LGHQQIALL
L/S10
182 - IALL
S10
185 - LAGPLS
L
185 - LAGPLSSVSARL
L/H11
187 - GPLSSVSAR
L/H11
198 - LAGWHK
H11
204 - YLTRNQIQP
H11/L
208 - NQIQPIAEREGD
L/S12/L
213 - IAEREGDWSAMSG
S12/L/H13
Deuterons
Time (seconds)

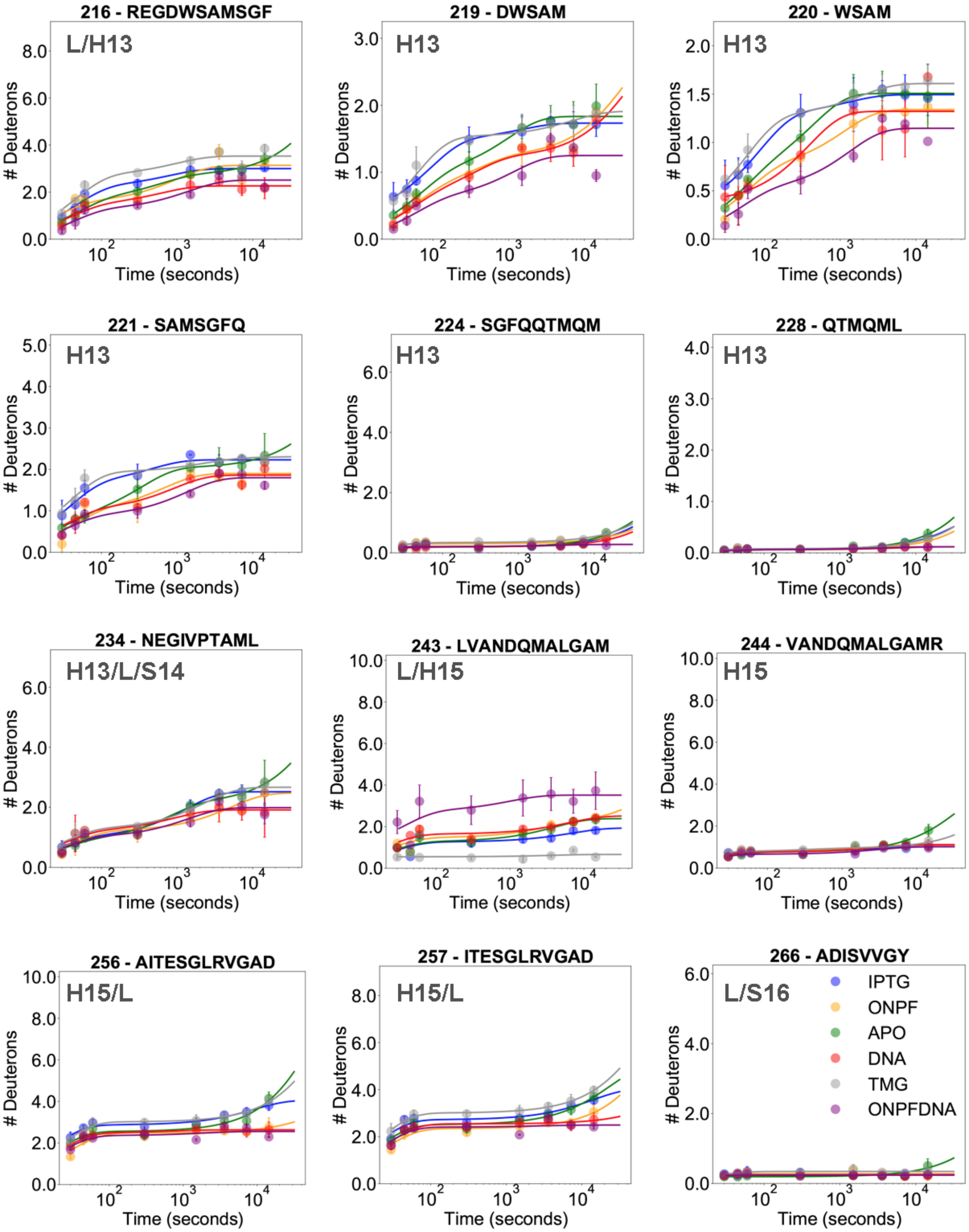
216 - REGDWSAMSGF
L/H13
219 - DWSAM
H13
220 - WSAM
H13
221 - SAMSGFQ
H13
224 - SGFQQTMQM
H13
228 - QTMQML
H13
234 - NEGIVPTAML
H13/L/S14
243 - LVANDQMALGAM
L/H15
244 - VANDQMALGAMR
H15
256 - AITESGLRVGAD
H15/L
257 - ITESGLRVGAD
H15/L
266 - ADISVVGY
L/S16
Deuterons
Time (seconds)
IPTG
ONPF
APO
DNA
TMG
ONPFDNA

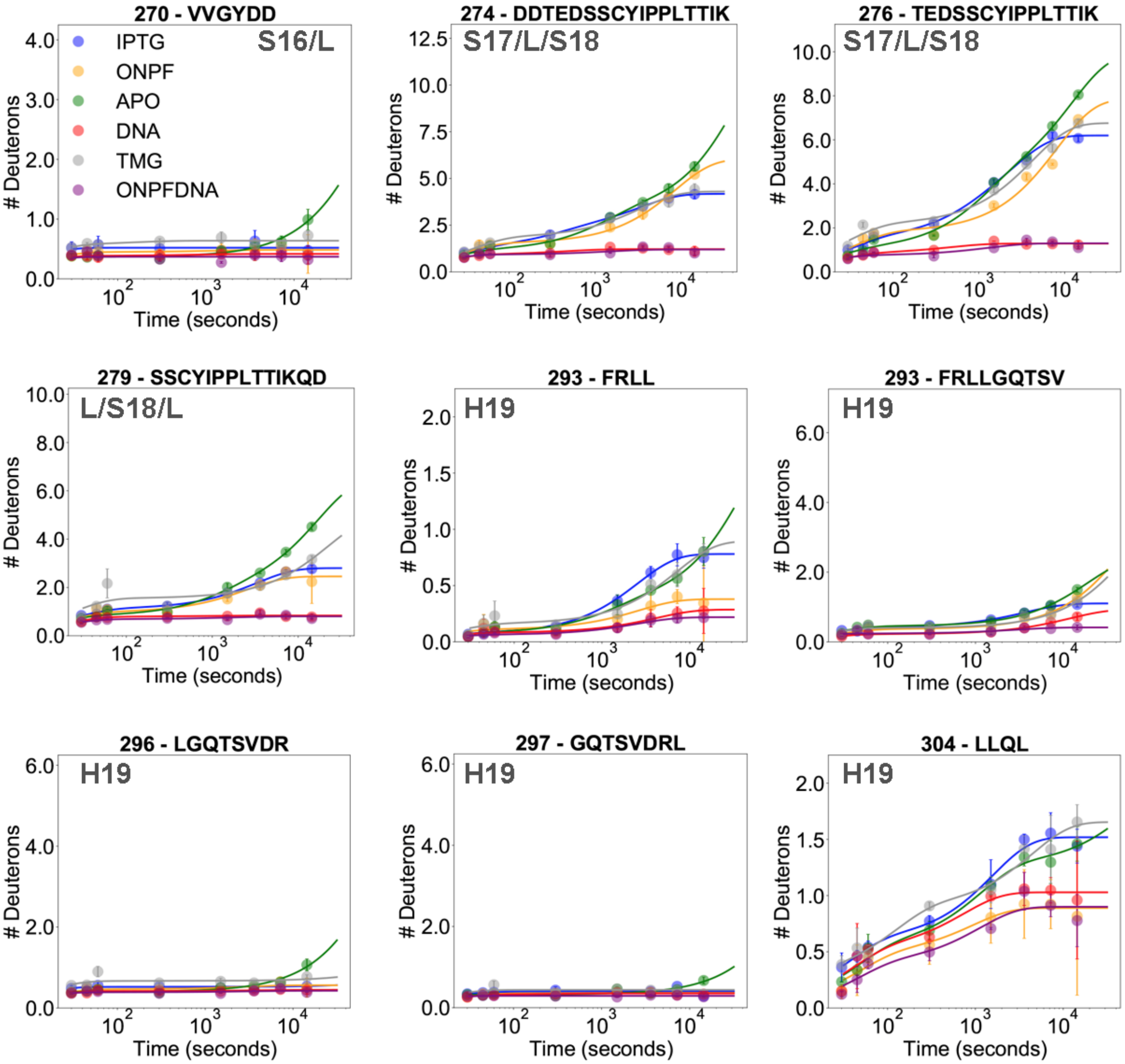

270 - VVGYDD
S16/L
IPTG
ONPF
APO
DNA
TMG
ONPFDNA
274 - DDTEDSSCYIPPLTTIK
S17/L/S18
276 - TEDSSCYIPPLTTIK
S17/L/S18
279 - SSCYIPPLTTIKQD
L/S18/L
293 - FRLL
H19
293 - FRLLGQTSV
H19
296 - LGQTSVDR
H19
297 - GQTSVDRL
H19
304 - LLQL
H19
Deuterons
Time (seconds)

Supplementary Figure 5. Example spectra for peptide 115 - LAQRVSGL.

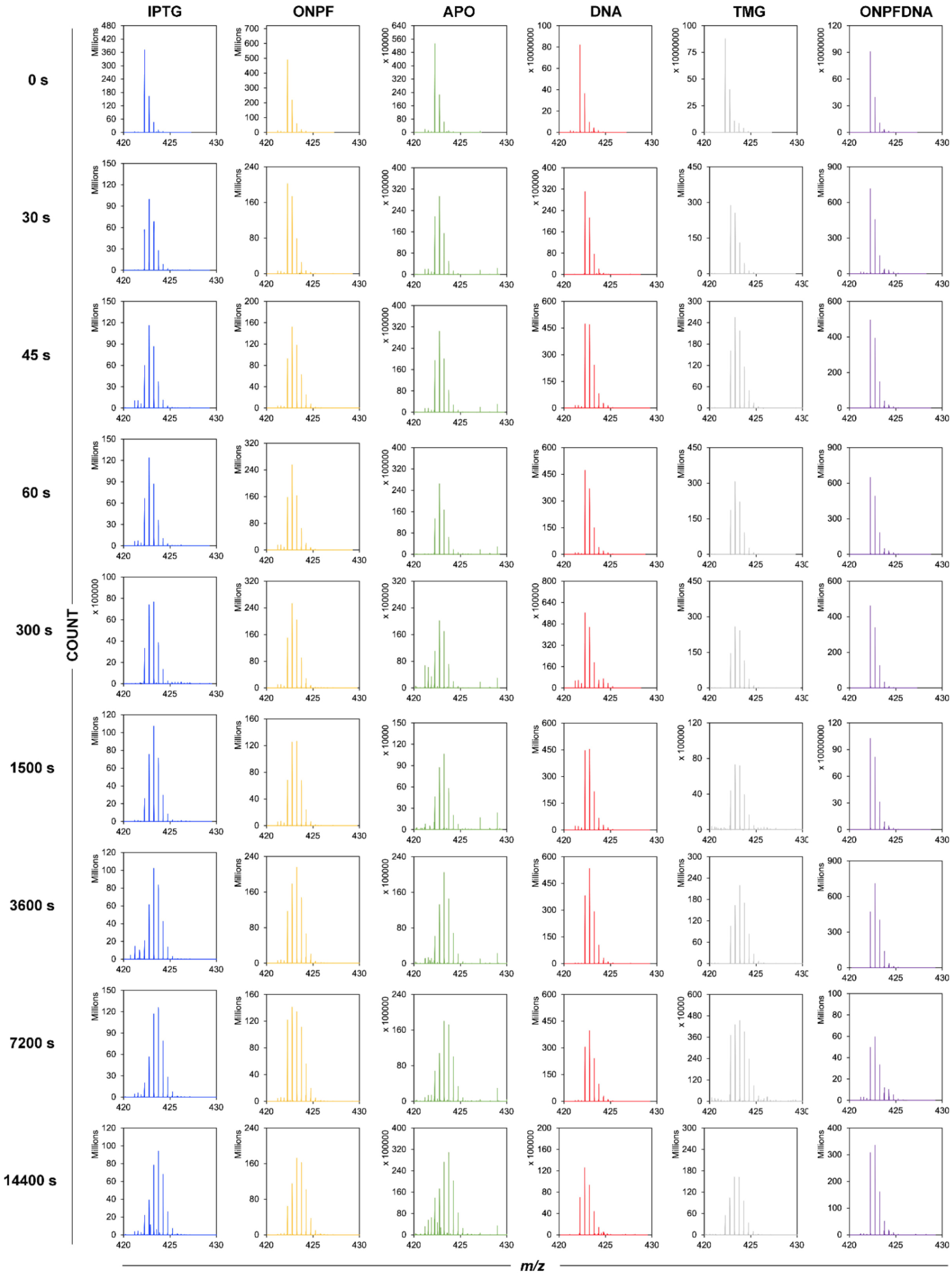

Supplementary Figure 6. Increasing the stringency criteria to determine differences in H-D exchange between IPTG-LacI and DNA-LacI. (A, B) Two alternative thresholds, indicated in Figure panel title, to highlight key secondary structures that undergo rigidification or de-rigidification upon the addition of IPTG to LacI-DNA, colored as in the main text figures (purple: more rigid in DNA-LacI compared to IPTG-LacI; team: more rigid in IPTG-LacI than DNA-LacI). **(C)** With the increased stringency criteria in (B), no residues in the "other" category are mutationally tolerant.

A 30% difference at 3+ timepoints

B 30% difference at 4+ timepoints

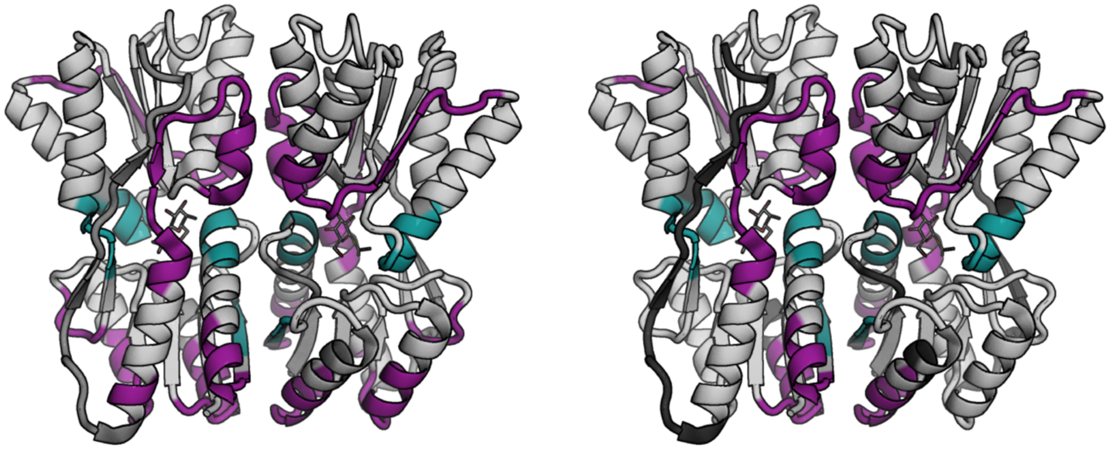

C

	I^S	I^-	other	% of 'other' that are mutationally tolerant
Residues in regions rigidified in **IPTG-LacI**	68%	11%	21%	0%
Residues in regions rigidified in **DNA-LacI**	13%	40%	47%	0%

Supplementary Figure 7. H-D exchange at interface helices. Peptides in the structural models for LacI on the left are colored green, magenta and navy blue to match the heading (peptide starting position and sequence) of the respective deuterium uptake plots on the right. **(A)** H-D exchange in helix 2 at the N-terminal subdomain monomer-monomer interface of LacI (PDB ID 2P9H). The N-terminal region of the helix (green) shows decreased exchange in IPTG-LacI (blue line), whereas the C-terminal region of the helix (navy blue) shows decreased exchange in DNA-LacI (red line). The central region of the helix (magenta) is rigid in IPTG-LacI and DNA-LacI, but shows increased exchange in ONPF-LacI (orange line). **(B)** The outward-facing alpha helices 13 (green) and 17 (navy blue) at the C-terminal subdomain monomer-monomer interface of LacI show decreased exchange in DNA-LacI as compared to IPTG-LacI. In these uptake plots, *n* = 2 biologically independent samples for peptides starting at residues 72, 77, 219, 244, and 276. For the peptide starting at residue 82, *n* = 5 biologically independent samples for IPTG-LacI and ONPF-LacI, and *n* = 3 biologically independent samples for DNA-LacI. Data are presented as mean values +/- standard deviations.

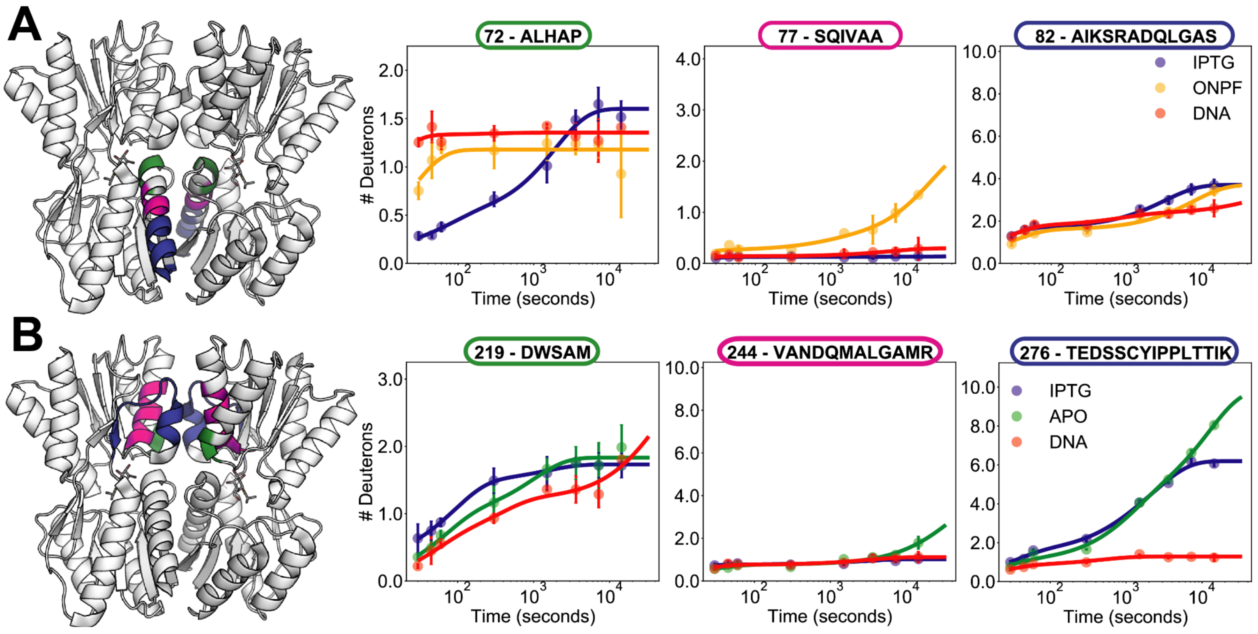

Supplementary Figure 8. Reduced inter-beta-sheet distance in the N-terminal subdomain monomer-monomer interface in IPTG-LacI compared to DNA-LacI. (A) A water molecule bridges the distance between beta strand residues in each subunit in the structure of DNA-LacI (PDB ID 1EFA). **(B)** In IPTG-LacI, valines in the interface beta strands on each subunit form direct backbone-backbone hydrogen bonds (PDB ID 2P9H).

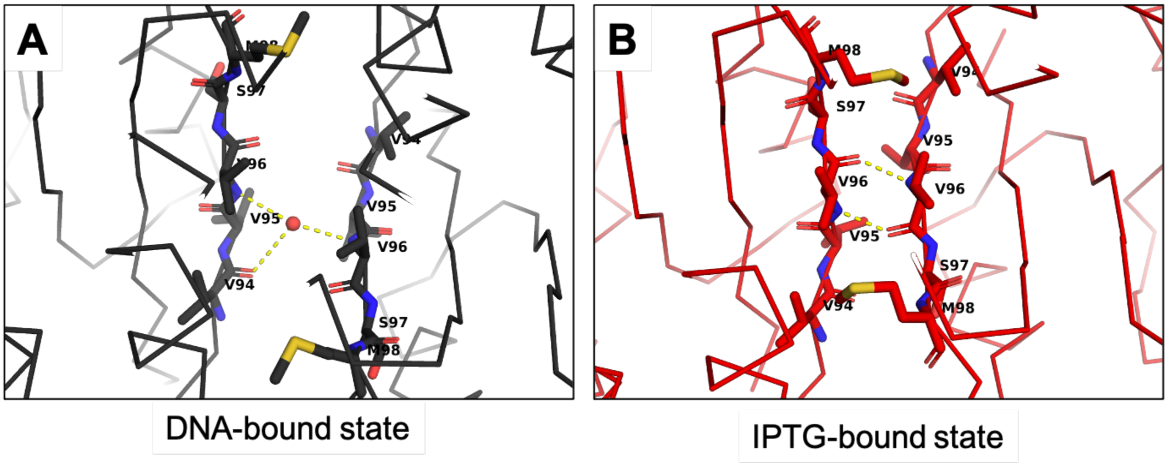

Supplementary Figure 9. Fractional differences in H-D exchange between LacI states at single HX timepoints based on maximum theoretical exchange and corrected for back-exchange. The fractional difference was calculated as $\Delta HX_t = \Delta D_t / (N_H m_\delta)$, where $\Delta D_t = m_{A,t} - m_{B,t}$ (the difference between averaged centroids at time t for states A and B); N_H is the number of exchangeable amide hydrogens in the peptide (total length - 2 - number of prolines); and $m_\delta \approx 1$ Da, the difference between the mass of deuterium and protium. The heading of each heatmap shows the states compared in each heatmap and the direction of the comparison. For example, in the plot with the heading "APO-DNA," the blue boxes denote less exchange in apo-LacI, and the red boxes denote less exchange in DNA-LacI.

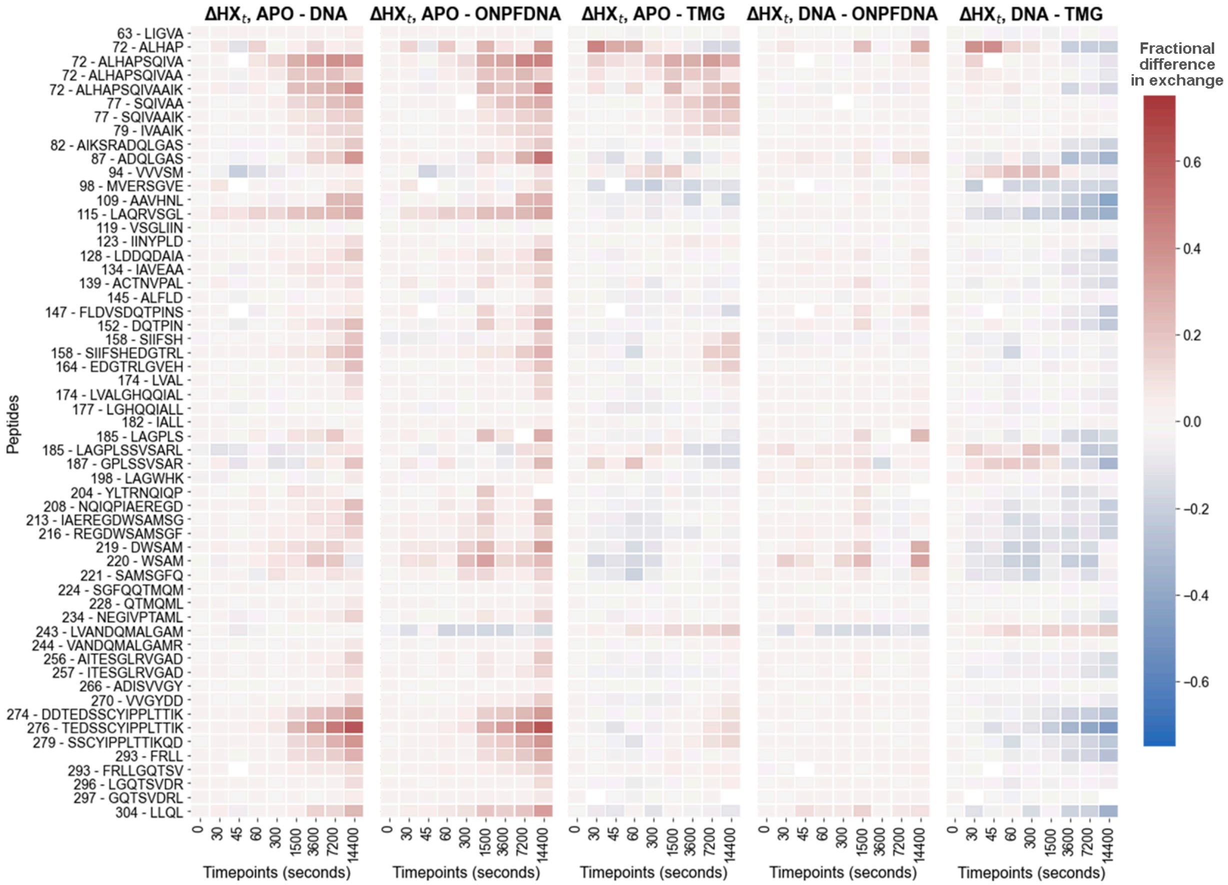

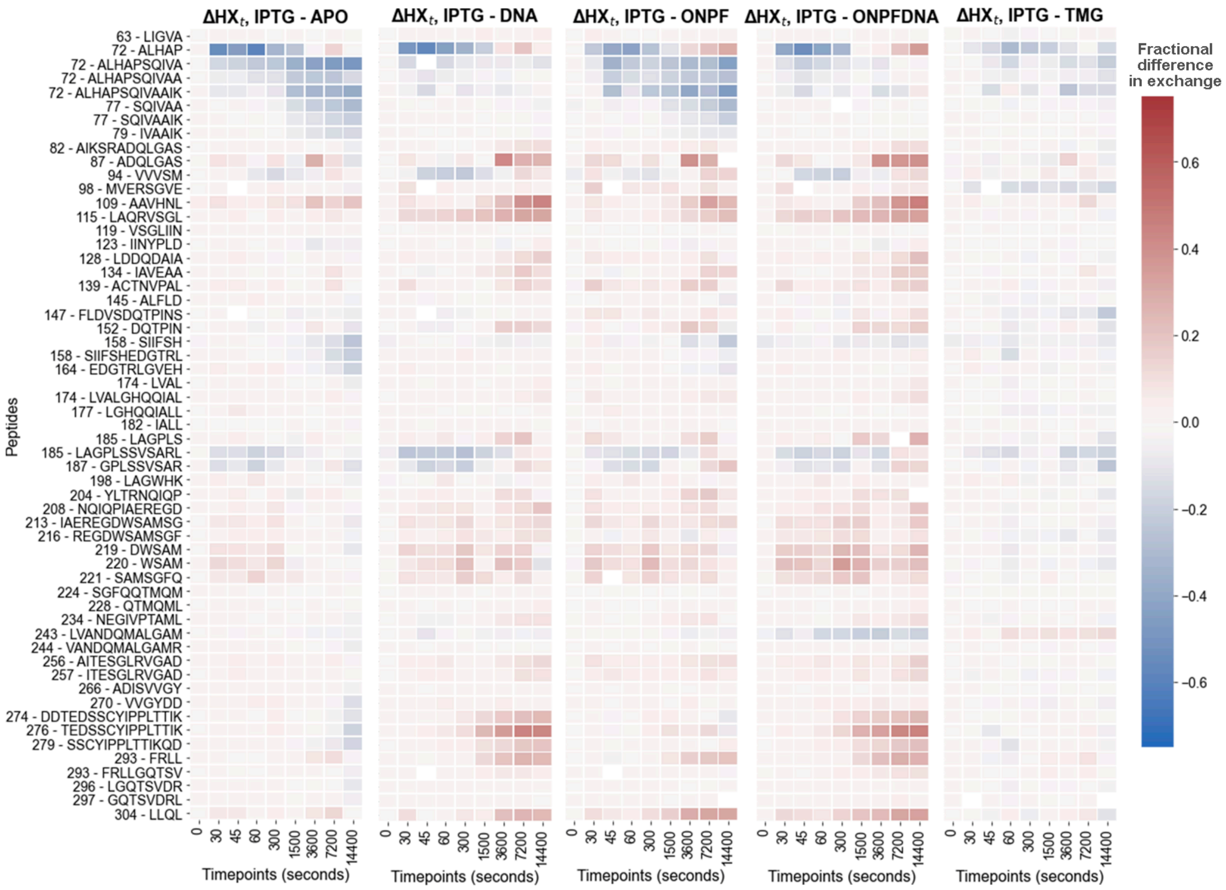

ΔHX$_t$, IPTG - APO
ΔHX$_t$, IPTG - DNA
ΔHX$_t$, IPTG - ONPF
ΔHX$_t$, IPTG - ONPFDNA
ΔHX$_t$, IPTG - TMG
Peptides
63 - LIGVA
72 - ALHAP
72 - ALHAPSQIVA
72 - ALHAPSQIVAA
72 - ALHAPSQIVAAIK
77 - SQIVAA
77 - SQIVAAIK
79 - IVAAIK
82 - AIKSRADQLGAS
87 - ADQLGAS
94 - VVVSM
98 - MVERSGVE
109 - AAVHNL
115 - LAQRVSGL
119 - VSGLIIN
123 - IINYPLD
128 - LDDQDAIA
134 - IAVEAA
139 - ACTNVPAL
145 - ALFLD
147 - FLDVSDQTPINS
152 - DQTPIN
158 - SIIFSH
158 - SIIFSHEDGTRL
164 - EDGTRLGVEH
174 - LVAL
174 - LVALGHQQIAL
177 - LGHQQIALL
182 - IALL
185 - LAGPLS
185 - LAGPLSSVSARL
187 - GPLSSVSAR
198 - LAGWHK
204 - YLTRNQIQP
208 - NQIQPIAEREGD
213 - IAEREGDWSAMSG
216 - REGDWSAMSGF
219 - DWSAM
220 - WSAM
221 - SAMSGFQ
224 - SGFQQTMQM
228 - QTMQML
234 - NEGIVPTAML
243 - LVANDQMALGAM
244 - VANDQMALGAMR
256 - AITESGLRVGAD
257 - ITESGLRVGAD
266 - ADISVVGY
270 - VVGYDD
274 - DDTEDSSCYIPPLTTIK
276 - TEDSSCYIPPLTTIK
279 - SSCYIPPLTTIKQD
293 - FRLL
293 - FRLLGQTSV
296 - LGQTSVDR
297 - GQTSVDRL
304 - LLQL
0 30 45 60 300 1500 3600 7200 14400
Timepoints (seconds)
Fractional difference in exchange
0.6
0.4
0.2
0.0
−0.2
−0.4
−0.6

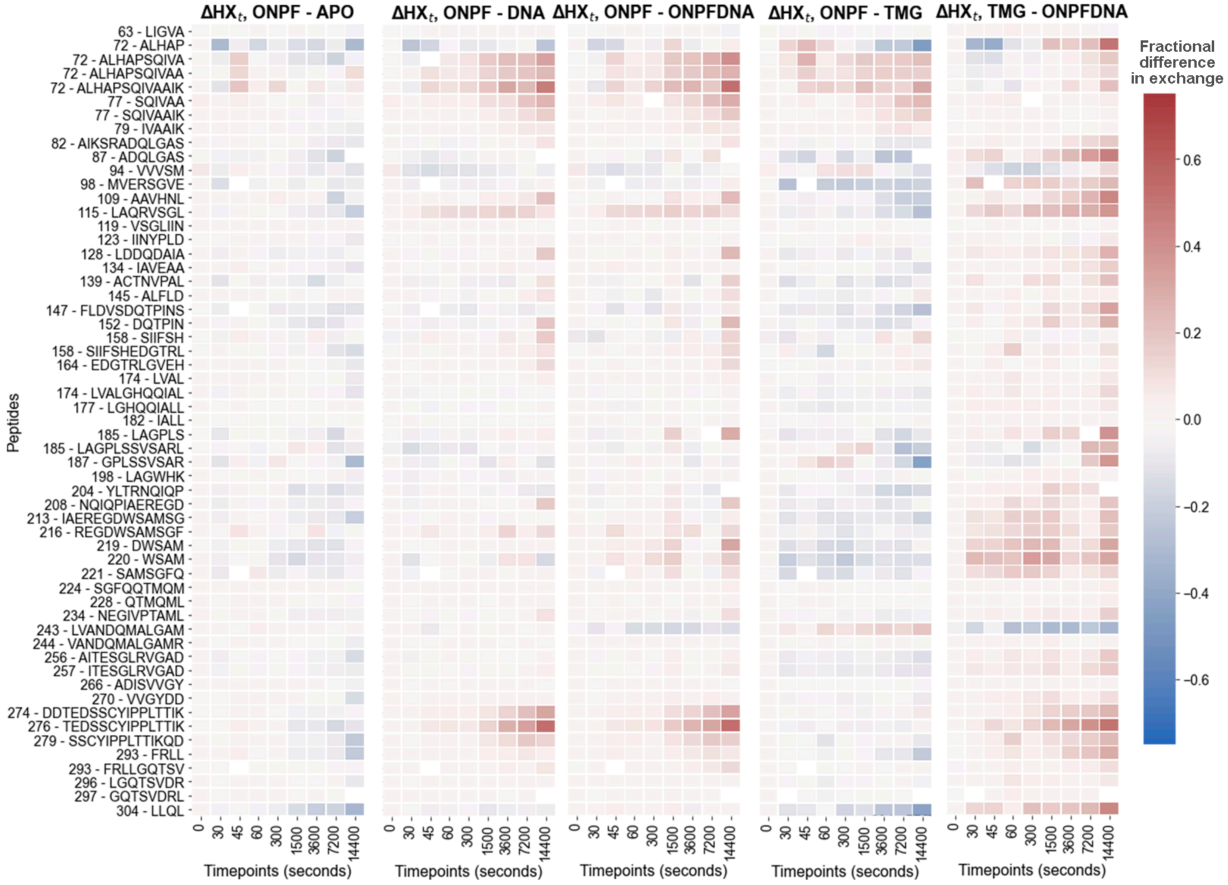
ΔHX$_t$, ONPF - APO
ΔHX$_t$, ONPF - DNA
ΔHX$_t$, ONPF - ONPFDNA
ΔHX$_t$, ONPF - TMG
ΔHX$_t$, TMG - ONPFDNA
Fractional difference in exchange
0.6
0.4
0.2
0.0
−0.2
−0.4
−0.6
Peptides
63 - LIGVA
72 - ALHAP
72 - ALHAPSQIVA
72 - ALHAPSQIVAA
72 - ALHAPSQIVAAIK
77 - SQIVAA
77 - SQIVAAIK
79 - IVAAIK
82 - AIKSRADQLGAS
87 - ADQLGAS
94 - VVVSM
98 - MVERSGVE
109 - AAVHNL
115 - LAQRVSGL
119 - VSGLIIN
123 - IINYPLD
128 - LDDQDAIA
134 - IAVEAA
139 - ACTNVPAL
145 - ALFLD
147 - FLDVSDQTPINS
152 - DQTPIN
158 - SIIFSH
158 - SIIFSHEDGTRL
164 - EDGTRLGVEH
174 - LVAL
174 - LVALGHQQIAL
177 - LGHQQIALL
182 - IALL
185 - LAGPLS
185 - LAGPLSSVSARL
187 - GPLSSVSAR
198 - LAGWHK
204 - YLTRNQIQP
208 - NQIQPIAEREGD
213 - IAEREGDWSAMSG
216 - REGDWSAMSGF
219 - DWSAM
220 - WSAM
221 - SAMSGFQ
224 - SGFQQTMQM
228 - QTMQML
234 - NEGIVPTAML
243 - LVANDQMALGAM
244 - VANDQMALGAMR
256 - AITESGLRVGAD
257 - ITESGLRVGAD
266 - ADISVVGY
270 - VVGYDD
274 - DDTEDSSCYIPPLTTIK
276 - TEDSSCYIPPLTTIK
279 - SSCYIPPLTTIKQD
293 - FRLL
293 - FRLLGQTSV
296 - LGQTSVDR
297 - GQTSVDRL
304 - LLQL
0
30
45
60
300
1500
3600
7200
14400
Timepoints (seconds)

Supplementary Figure 10. Comparison of DNA-LacI, IPTG-LacI, and IPTG-DNA-LacI by fractional differences in H-D exchange at single HX timepoints for 199 peptides. The fractional difference was calculated by the same method as for plots in Supplementary Figure 9.

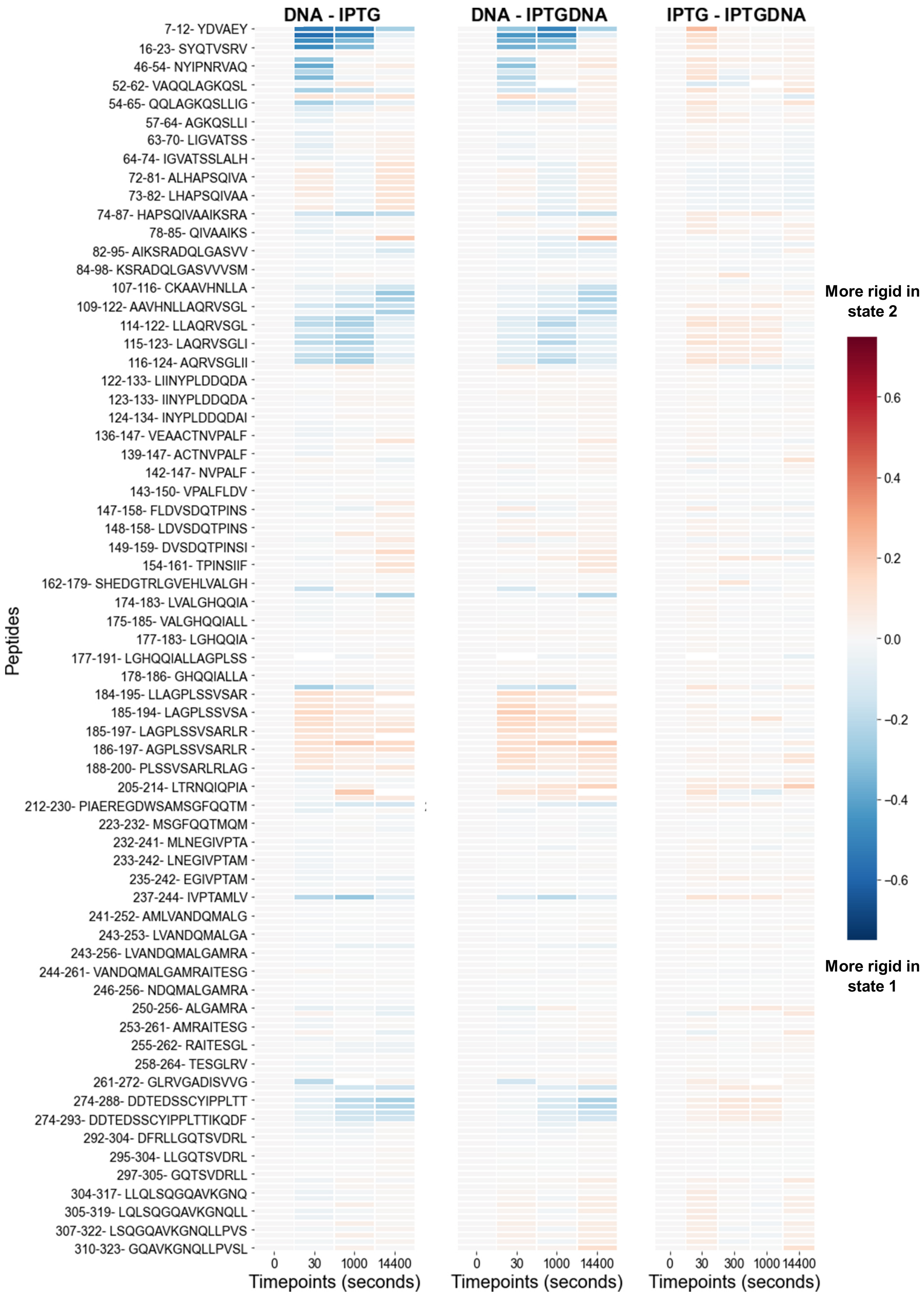

DNA - IPTG
DNA - IPTGDNA
IPTG - IPTGDNA
Peptides
7-12- YDVAEY
16-23- SYQTVSRV
46-54- NYIPNRVAQ
52-62- VAQQLAGKQSL
54-65- QQLAGKQSLLIG
57-64- AGKQSLLI
63-70- LIGVATSS
64-74- IGVATSSLALH
72-81- ALHAPSQIVA
73-82- LHAPSQIVAA
74-87- HAPSQIVAAIKSRA
78-85- QIVAAIKS
82-95- AIKSRADQLGASVV
84-98- KSRADQLGASVVVSM
107-116- CKAAVHNLLA
109-122- AAVHNLLAQRVSGL
114-122- LLAQRVSGL
115-123- LAQRVSGLI
116-124- AQRVSGLII
122-133- LIINYPLDDQDA
123-133- IINYPLDDQDA
124-134- INYPLDDQDAI
136-147- VEAACTNVPALF
139-147- ACTNVPALF
142-147- NVPALF
143-150- VPALFLDV
147-158- FLDVSDQTPINS
148-158- LDVSDQTPINS
149-159- DVSDQTPINSI
154-161- TPINSIIF
162-179- SHEDGTRLGVEHLVALGH
174-183- LVALGHQQIA
175-185- VALGHQQIALL
177-183- LGHQQIA
177-191- LGHQQIALLAGPLSS
178-186- GHQQIALLA
184-195- LLAGPLSSVSAR
185-194- LAGPLSSVSA
185-197- LAGPLSSVSARLR
186-197- AGPLSSVSARLR
188-200- PLSSVSARLRLAG
205-214- LTRNQIQPIA
212-230- PIAEREGDWSAMSGFQQTM
223-232- MSGFQQTMQM
232-241- MLNEGIVPTA
233-242- LNEGIVPTAM
235-242- EGIVPTAM
237-244- IVPTAMLV
241-252- AMLVANDQMALG
243-253- LVANDQMALGA
243-256- LVANDQMALGAMRA
244-261- VANDQMALGAMRAITESG
246-256- NDQMALGAMRA
250-256- ALGAMRA
253-261- AMRAITESG
255-262- RAITESGL
258-264- TESGLRV
261-272- GLRVGADISVVG
274-288- DDTEDSSCYIPPLTT
274-293- DDTEDSSCYIPPLTTIKQDF
292-304- DFRLLGQTSVDRL
295-304- LLGQTSVDRL
297-305- GQTSVDRLL
304-317- LLQLSQGQAVKGNQ
305-319- LQLSQGQAVKGNQLL
307-322- LSQGQAVKGNQLLPVS
310-323- GQAVKGNQLLPVSL
0
30
1000
14400
Timepoints (seconds)
0
30
1000
14400
Timepoints (seconds)
0
30
300
1000
14400
Timepoints (seconds)
More rigid in state 2
0.6
0.4
0.2
0.0
−0.2
−0.4
−0.6
More rigid in state 1

Supplementary Figure 11. Uptake plots for 70 peptides in IPTG-DNA-LacI.

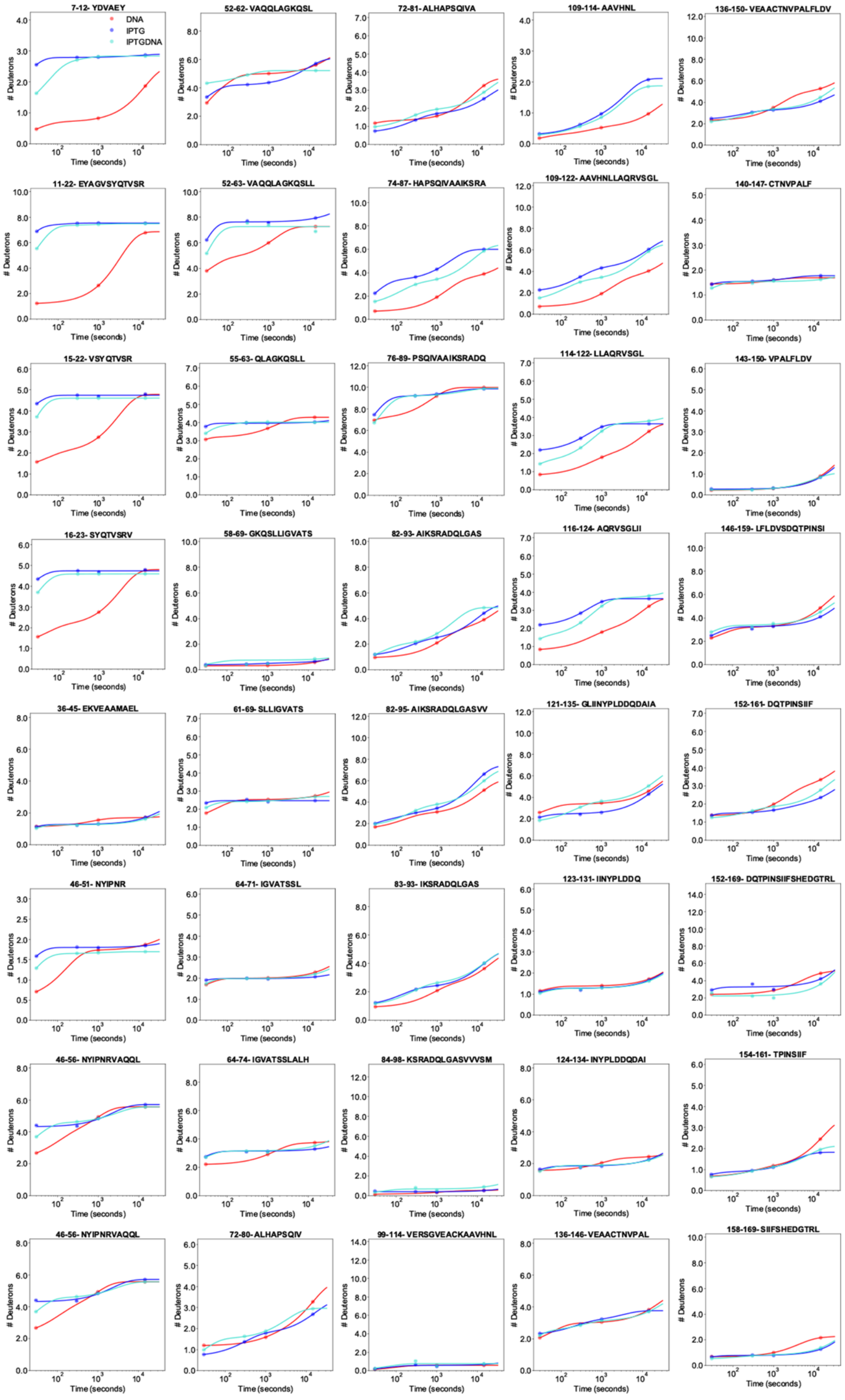

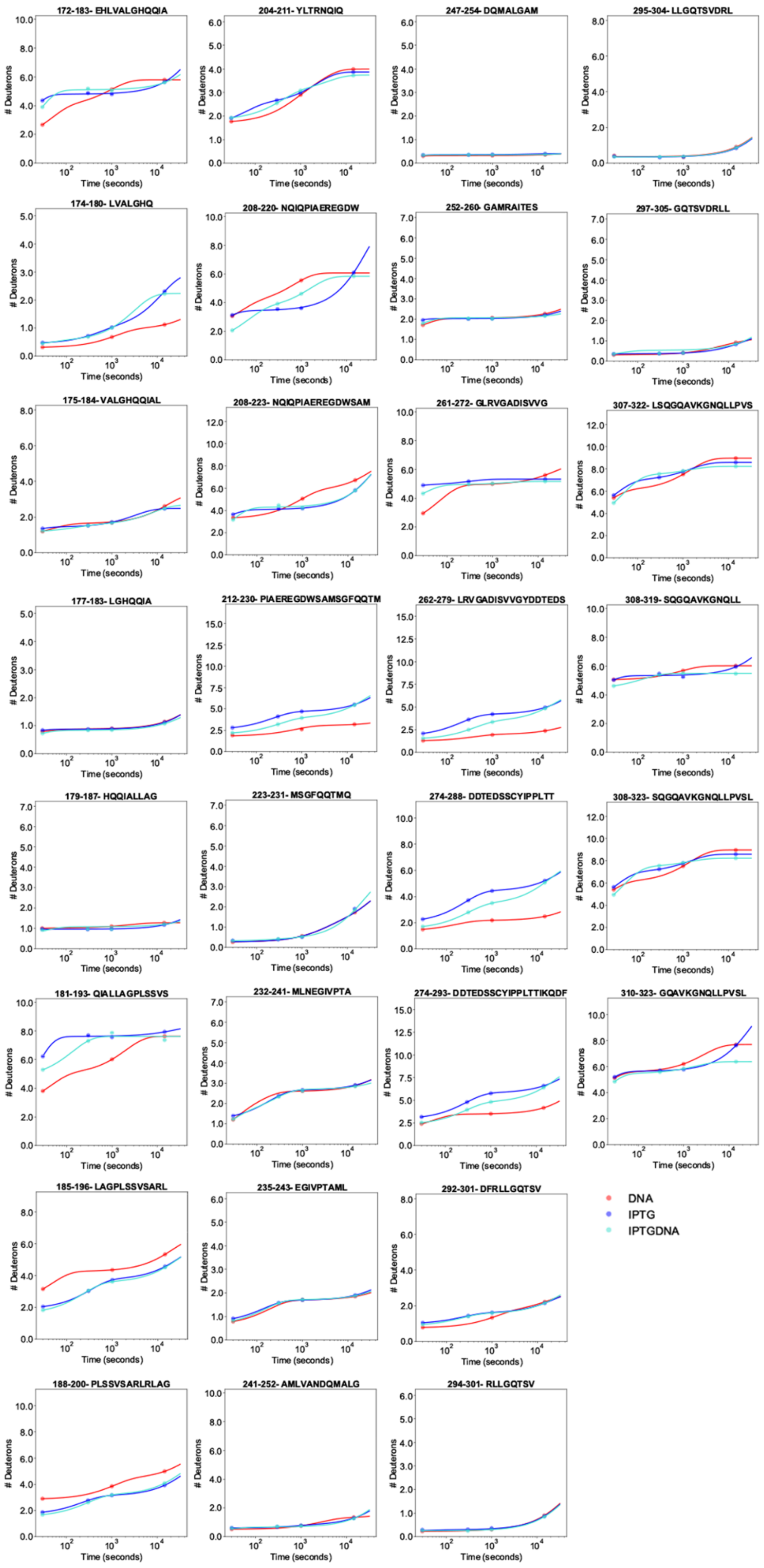

172-183- EHLVALGHQQIA
204-211- YLTRNQIQ
247-254- DQMALGAM
295-304- LLGQTSVDRL
174-180- LVALGHQ
208-220- NQIQPIAEREGDW
252-260- GAMRAITES
297-305- GQTSVDRLL
175-184- VALGHQQIAL
208-223- NQIQPIAEREGDWSAM
261-272- GLRVGADISVVG
307-322- LSQGQAVKGNQLLPVS
177-183- LGHQQIA
212-230- PIAEREGDWSAMSGFQQTM
262-279- LRVGADISVVGYDDTEDS
308-319- SQGQAVKGNQLL
179-187- HQQIALLAG
223-231- MSGFQQTMQ
274-288- DDTEDSSCYIPPLTT
308-323- SQGQAVKGNQLLPVSL
181-193- QIALLAGPLSSVS
232-241- MLNEGIVPTA
274-293- DDTEDSSCYIPPLTTIKQDF
310-323- GQAVKGNQLLPVSL
185-196- LAGPLSSVSARL
235-243- EGIVPTAML
292-301- DFRLLGQTSV
188-200- PLSSVSARLRLAG
241-252- AMLVANDQMALG
294-301- RLLGQTSV
Deuterons
Time (seconds)
DNA
IPTG
IPTGDNA

Supplementary Figure 12. LacI core domain colored according to effects of point mutations on gene expression. Each residue position was classified in one of fifteen categories according to its position in the protein structure as well as its most common mutational phenotype. The structure is colored according to these classifications as shown in Supplementary Table 1. Data from Suckow *et al.*[18] (PDB ID: 2P9H.)

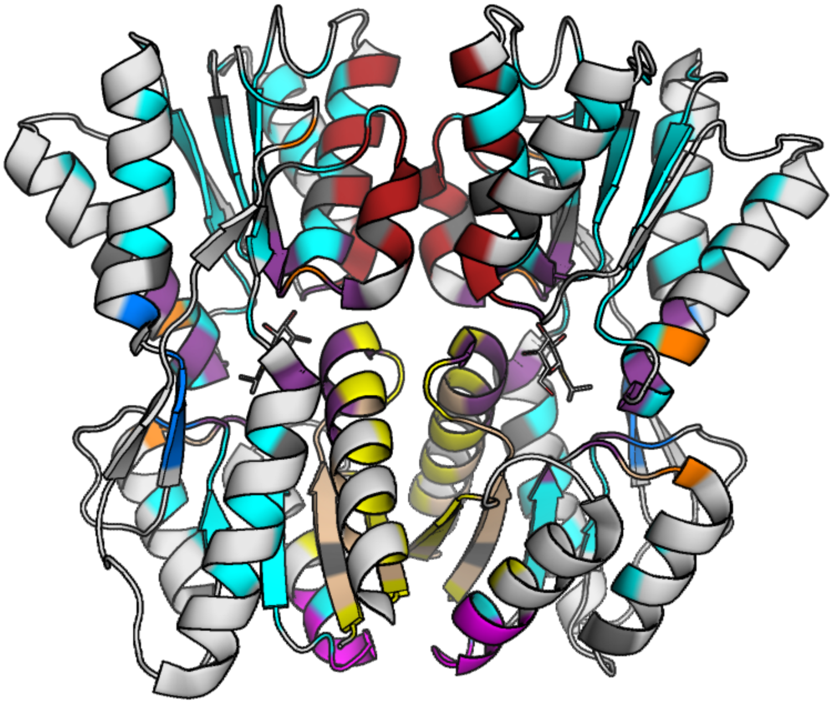

Supplementary Figure 13. Comparison of HDX/MS data to functional data from individual LacI mutants. H-D exchange patterns can be compared to functional data for residues at the monomer-monomer interface **(A-C)**, which play a role in switching the conformational ensemble of LacI from the IPTG-bound state to the DNA-bound state, and in a pocket peripheral loop **(D)**, which modulates the inducer affinity. Structures are colored according to the color scheme in Figures 2, 3.

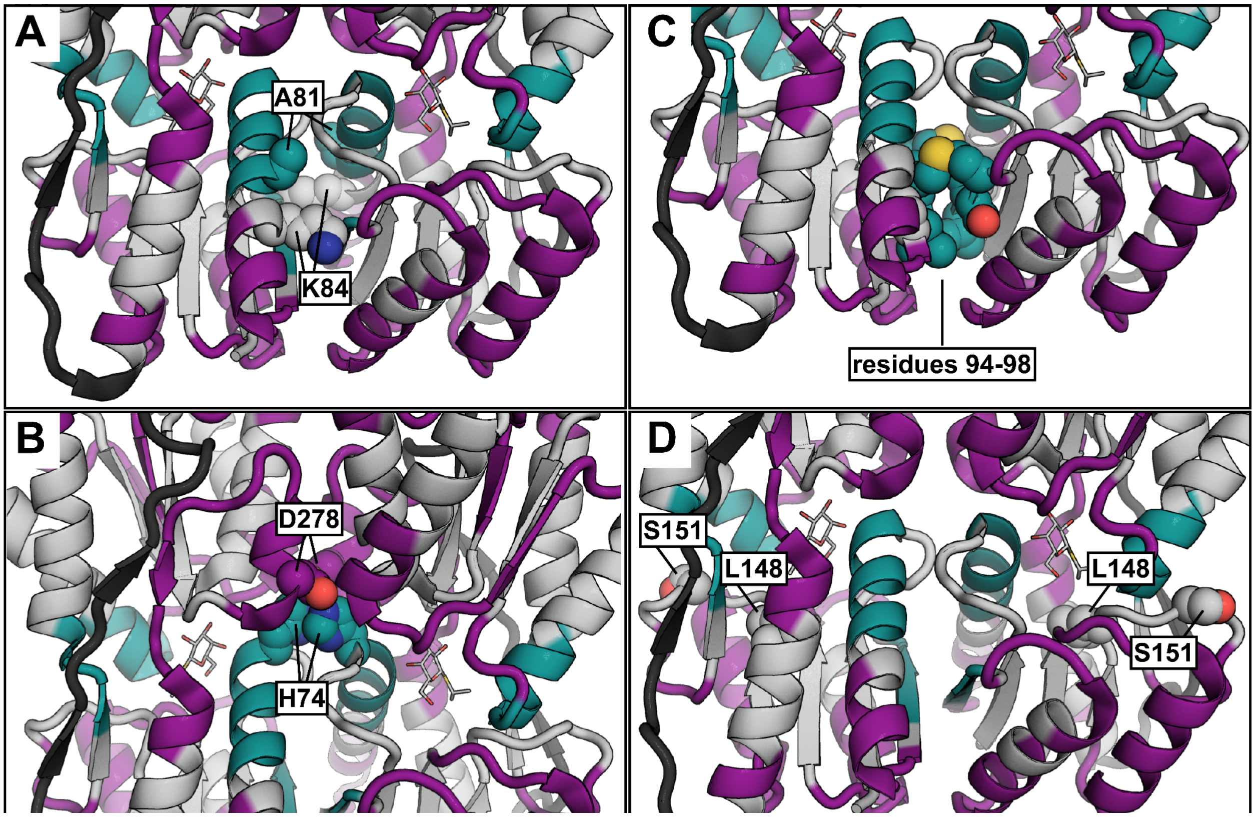

Supplementary Figure 14. Comparison of ONPF-LacI and ONPF-DNA-LacI with 14.6 mM ONPF by fractional differences in exchange at single timepoints for 192 peptides. The fractional difference was calculated as for Supplementary Figure 9.

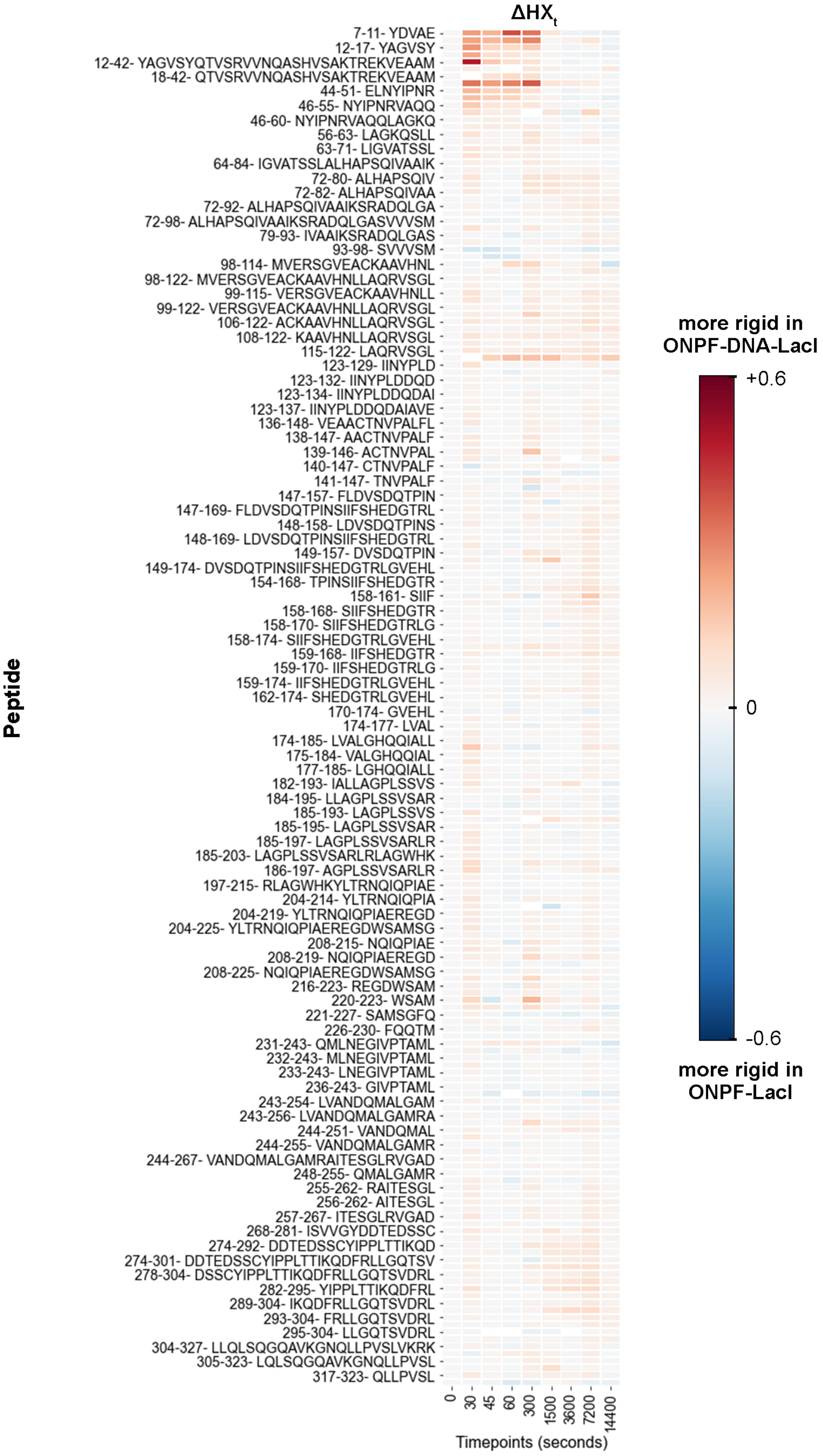

Supplementary Figure 15. Peptide coverage for 14.6 mM ONPF concentration HDX/MS experiments on ONPF-LacI and ONPF-DNA-LacI states. Each peptide is represented as a bar colored according to the arithmetic mean deuteration difference between the ONPF-LacI and ONPF-DNA-LacI states across all HDX times (ΔD_{dif})[19].

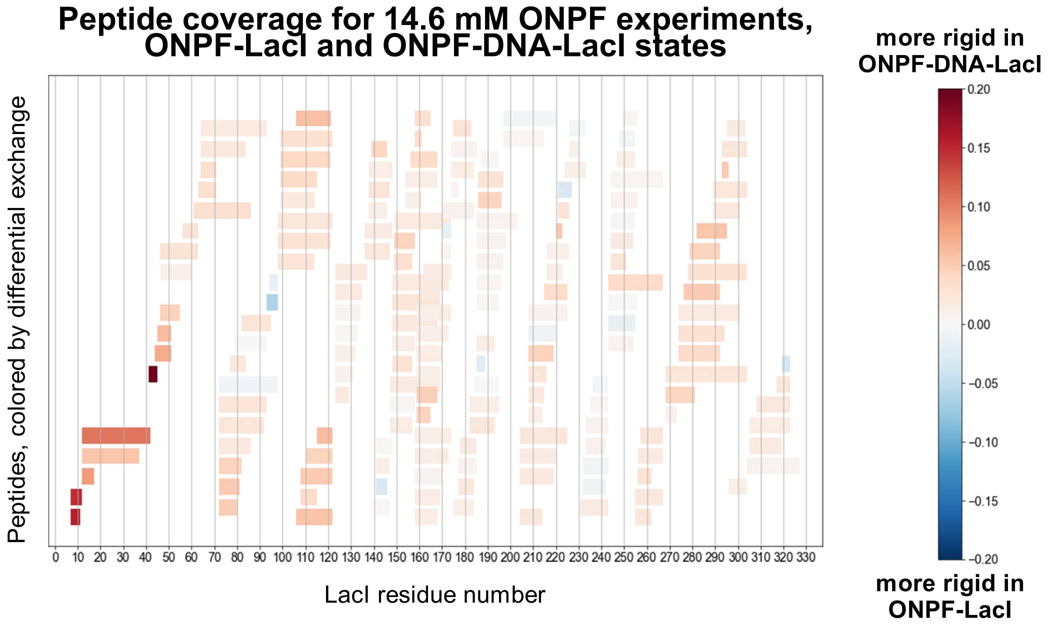

Supplementary Figure 16. Uptake plots for 50 LacI peptides in ONPF-LacI and ONPF-DNA-LacI states for HDX/MS experiments conducted with 14.6 mM ONPF, from 192 peptides in Supplementary Figures 14, 15. Plots are organized from N- to C-terminus of LacI. These data are presented as the mean of technical replicates from *n = 3* independent measurements for one LacI sample for each state with error bars showing standard deviations.

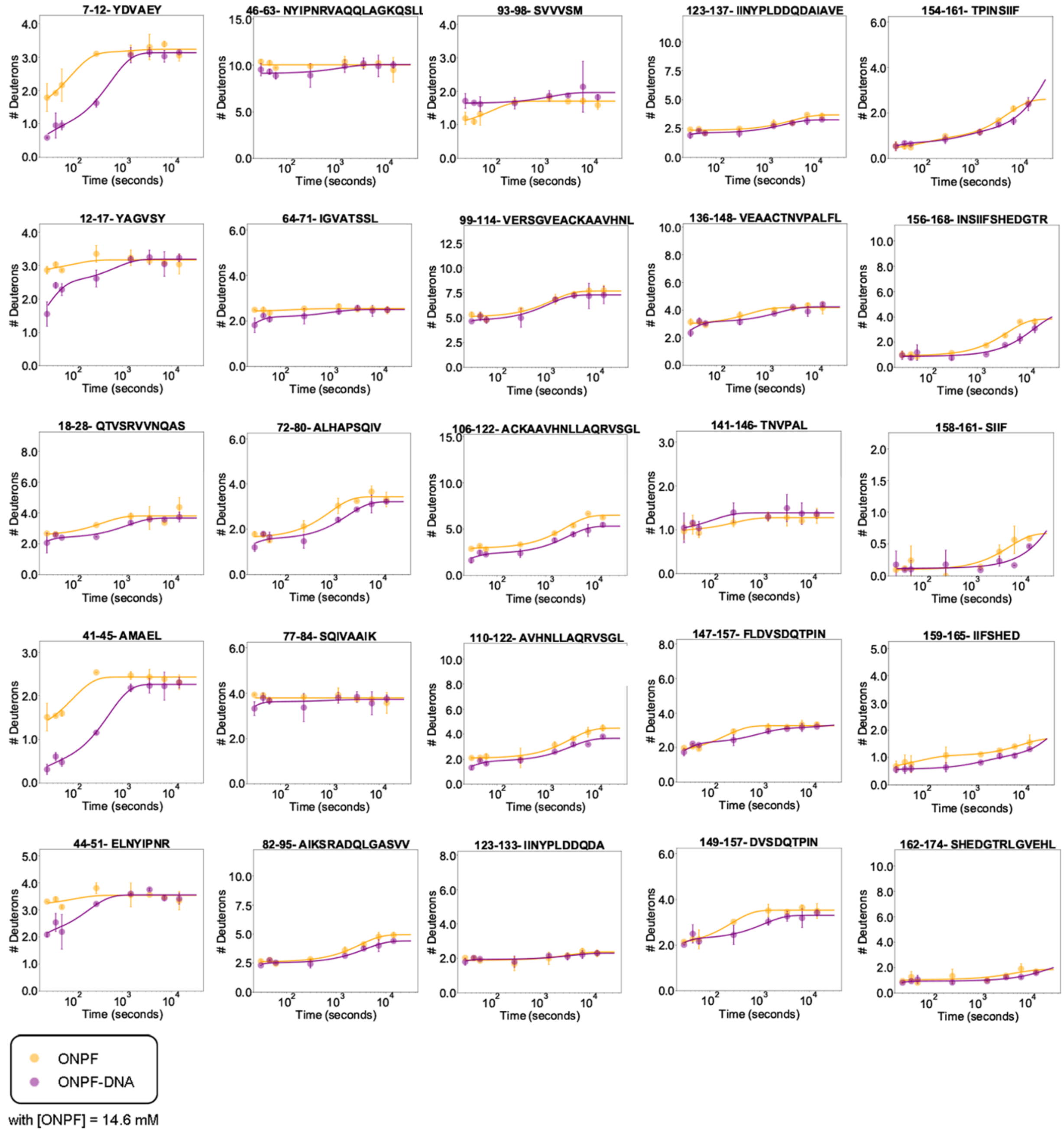

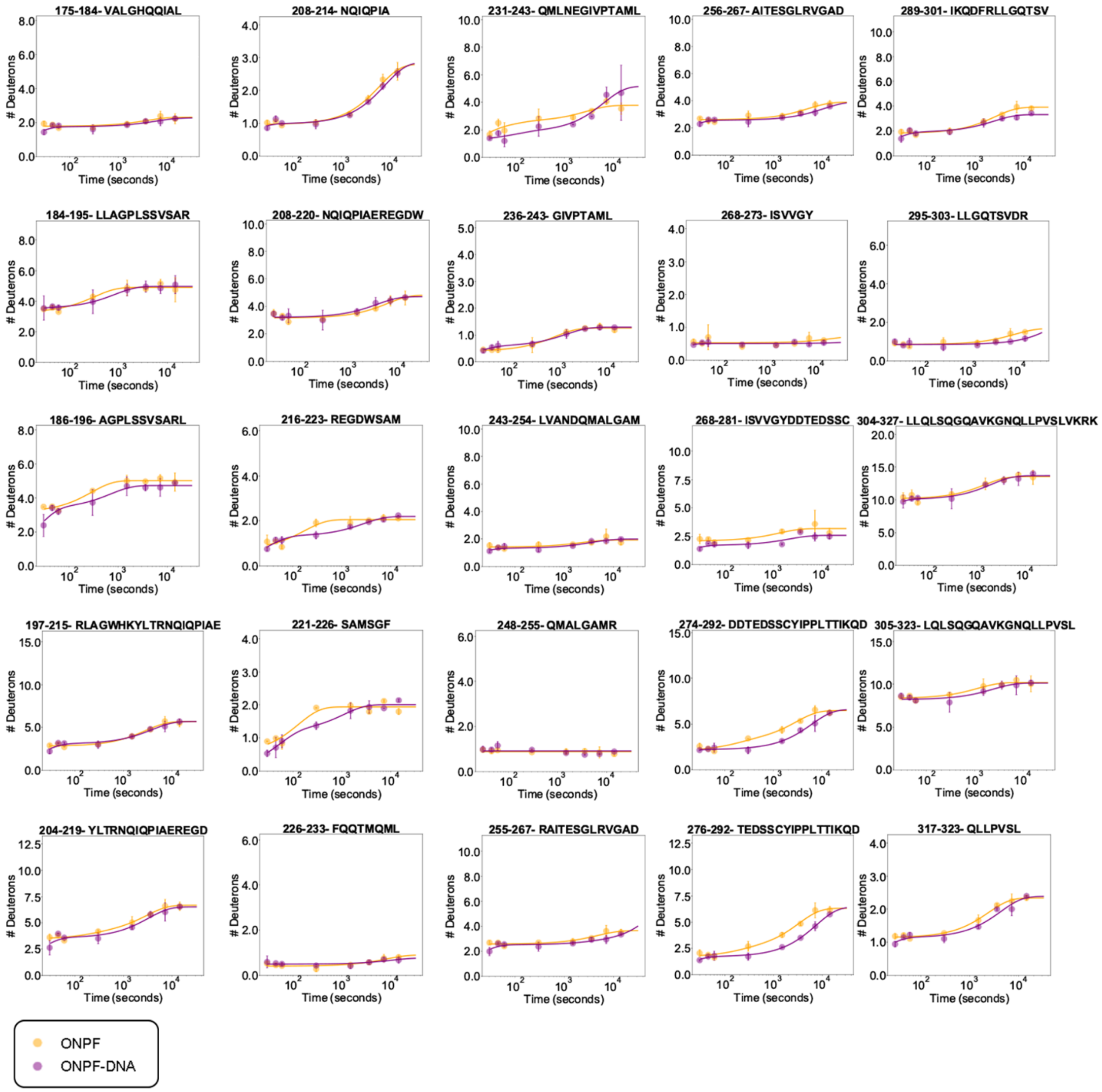

with [ONPF] = 14.6 mM

Supplementary Figure 17. Comparison of uptake plots for 24 peptides in ONPF-LacI and ONPF-DNA-LacI states for HDX/MS experiments with 300 µM and 14.6 mM ONPF. Plots are organized from N- to C-terminus of LacI. Plots with red asterisks mark similar but not identical peptides. Only the 300 µM ONPF samples are corrected for back-exchange. The data in the 14.6 mM column are presented as means of technical replicates from *n = 3* independent measurements for one LacI sample for each state. The data in the 300 µM column are presented as means from *n* = 5 experiments with biologically independent samples. All error bars are standard deviations.

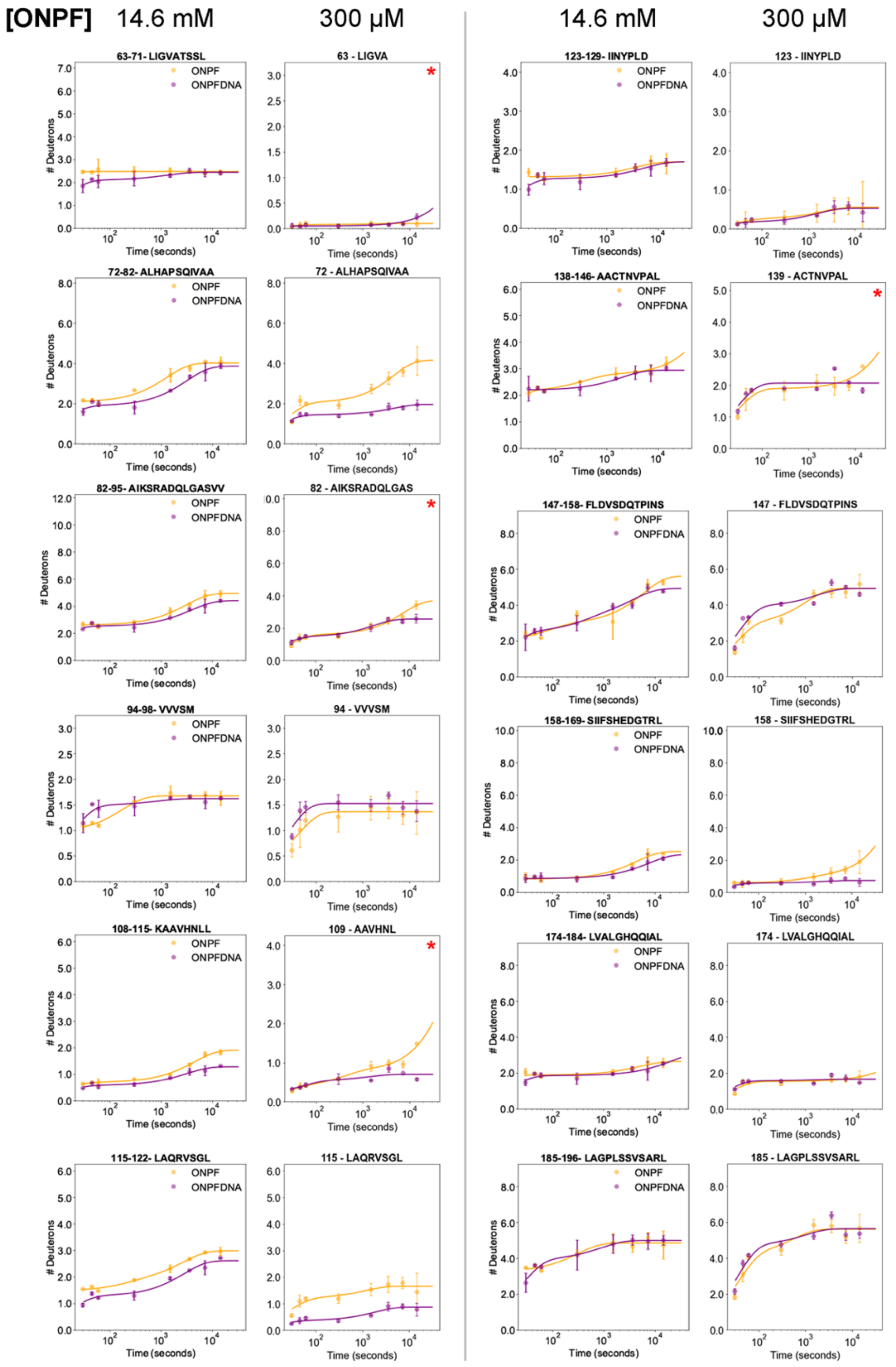
[ONPF]
14.6 mM
300 µM
14.6 mM
300 µM
63-71- LIGVATSSL
63 - LIGVA
123-129- IINYPLD
123 - IINYPLD
72-82- ALHAPSQIVAA
72 - ALHAPSQIVAA
138-146- AACTNVPAL
139 - ACTNVPAL
82-95- AIKSRADQLGASVV
82 - AIKSRADQLGAS
147-158- FLDVSDQTPINS
147 - FLDVSDQTPINS
94-98- VVVSM
94 - VVVSM
158-169- SIIFSHEDGTRL
158 - SIIFSHEDGTRL
108-115- KAAVHNLL
109 - AAVHNL
174-184- LVALGHQQIAL
174 - LVALGHQQIAL
115-122- LAQRVSGL
115 - LAQRVSGL
185-196- LAGPLSSVSARL
185 - LAGPLSSVSARL
ONPF
ONPFDNA
Deuterons
Time (seconds)

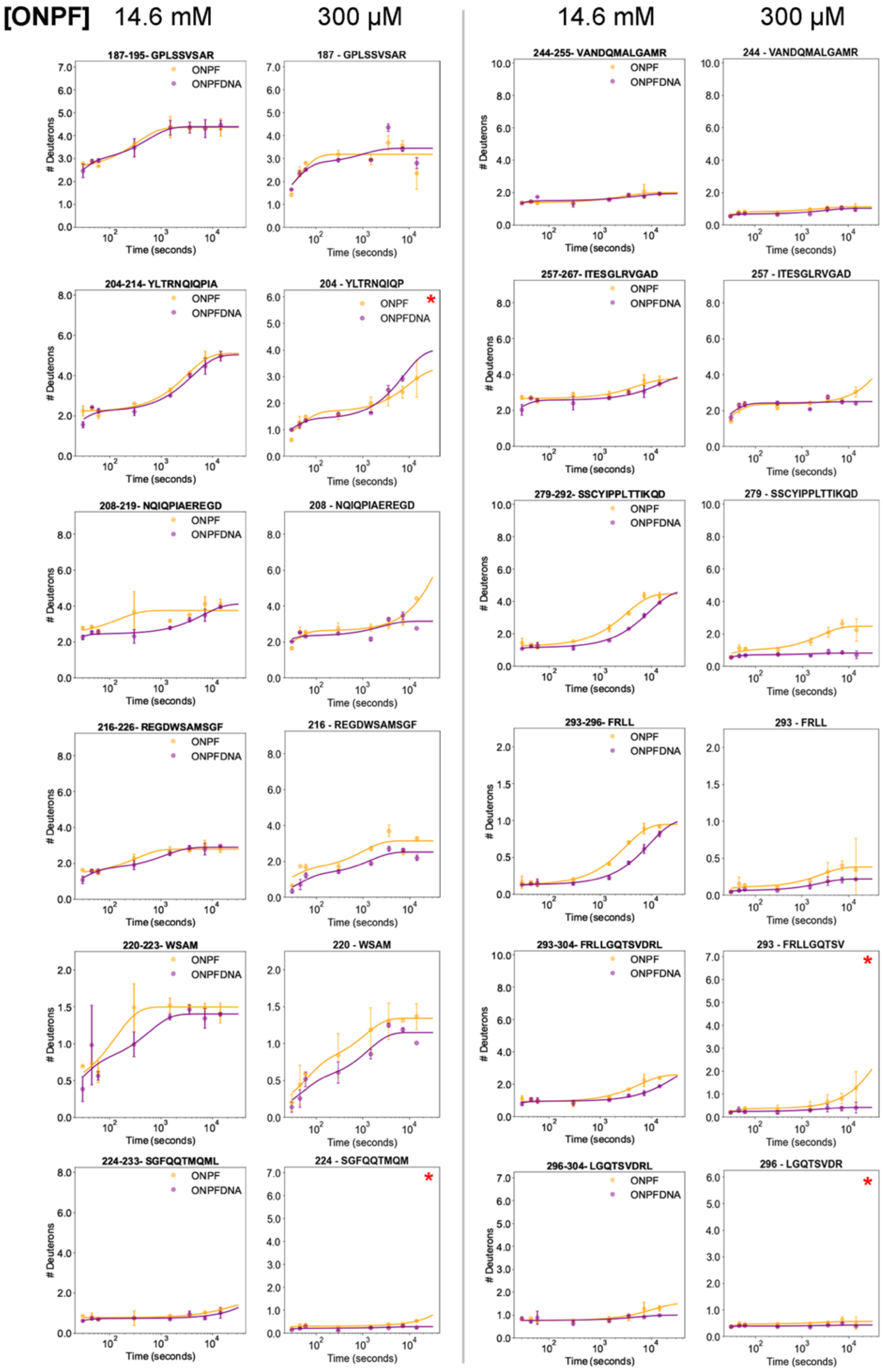

[ONPF]
14.6 mM
300 μM
14.6 mM
300 μM
187-195- GPLSSVSAR
187 - GPLSSVSAR
244-255- VANDQMALGAMR
244 - VANDQMALGAMR
204-214- YLTRNQIQPIA
204 - YLTRNQIQP
257-267- ITESGLRVGAD
257 - ITESGLRVGAD
208-219- NQIQPIAEREGD
208 - NQIQPIAEREGD
279-292- SSCYIPPLTTIKQD
279 - SSCYIPPLTTIKQD
216-226- REGDWSAMSGF
216 - REGDWSAMSGF
293-296- FRLL
293 - FRLL
220-223- WSAM
220 - WSAM
293-304- FRLLGQTSVDRL
293 - FRLLGQTSV
224-233- SGFQQTMQML
224 - SGFQQTMQM
296-304- LGQTSVDRL
296 - LGQTSVDR
ONPF
ONPFDNA
Deuterons
Time (seconds)

Supplementary Figure 18. Compiled HDX/MS data for the DNA-binding domain of LacI (residues 1-61). (A) The DNA-binding domain has a helix-turn-helix structure when bound to DNA. One subunit is shown in color and the other is shown in gray. In DNA-LacI, the hinge regions (darkest blue) have a helical structure and interact with each other (PDB ID: 1EFA). **(B-C)** Woods plots show that DNA-LacI (red) and ONPF-LacI (orange) exchange less than IPTG-LacI (blue) in the DNA-binding domain over the time course.

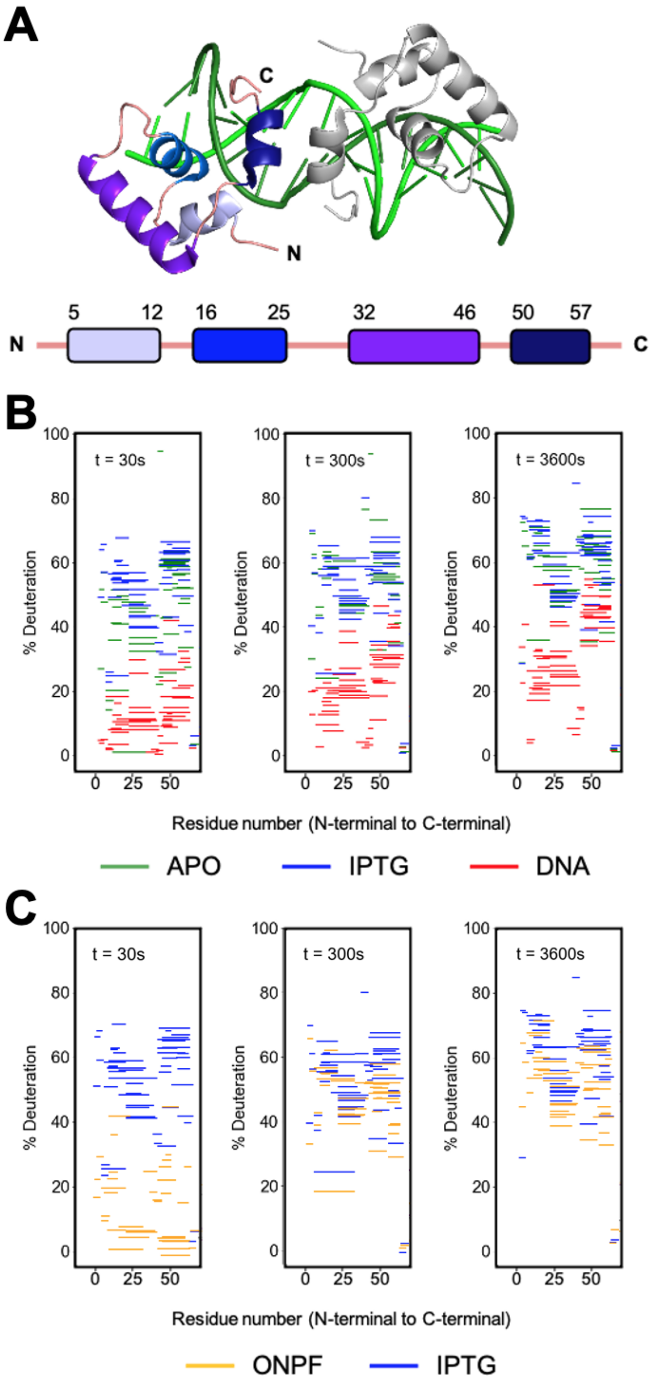

Supplementary Figure 19. Direct interactions of ligands with LacI residues in crystal structures. Residues within 6 Å of any atom in the ligand are shown as sticks, with atoms that are within 3.5 Å of the ligand colored black, for the **(A)** ONPF-bound and **(B)** IPTG-bound states of LacI. Structures are colored as in the color scheme in Fig. 4. PDB 2PAF for (A) and 2P9H for (B).

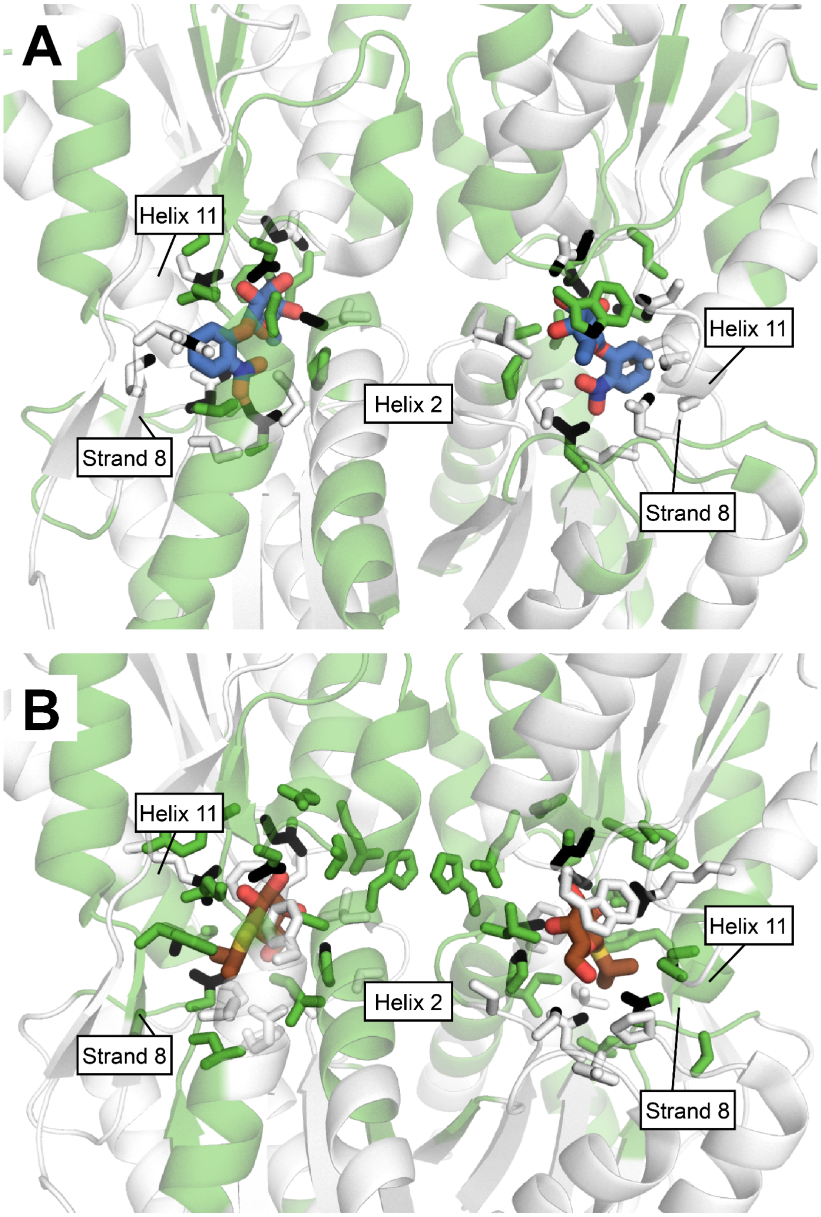

Supplementary Figure 20. Computationally predicted structural waters. (A) Representative models showing the placement of structural water molecules, which make 3-4 hydrogen bonds with ligand or protein atoms, in the ligand binding pocket of LacI. A semi-explicit water molecule placement protocol implemented in Rosetta (14) was used to solvate apo-LacI, DNA-LacI, and IPTG-LacI (Methods). LacI models were based on X-ray crystal structures (PDB IDs: 1LBI, 2PAF, 2P9H)[11,12]. **(B)** Several predicted structural water molecules are within 0.5 Å of assigned water molecules (structural and transient) in the X-ray crystal structure for a high-resolution (2.0 Å) structure of IPTG-LacI.

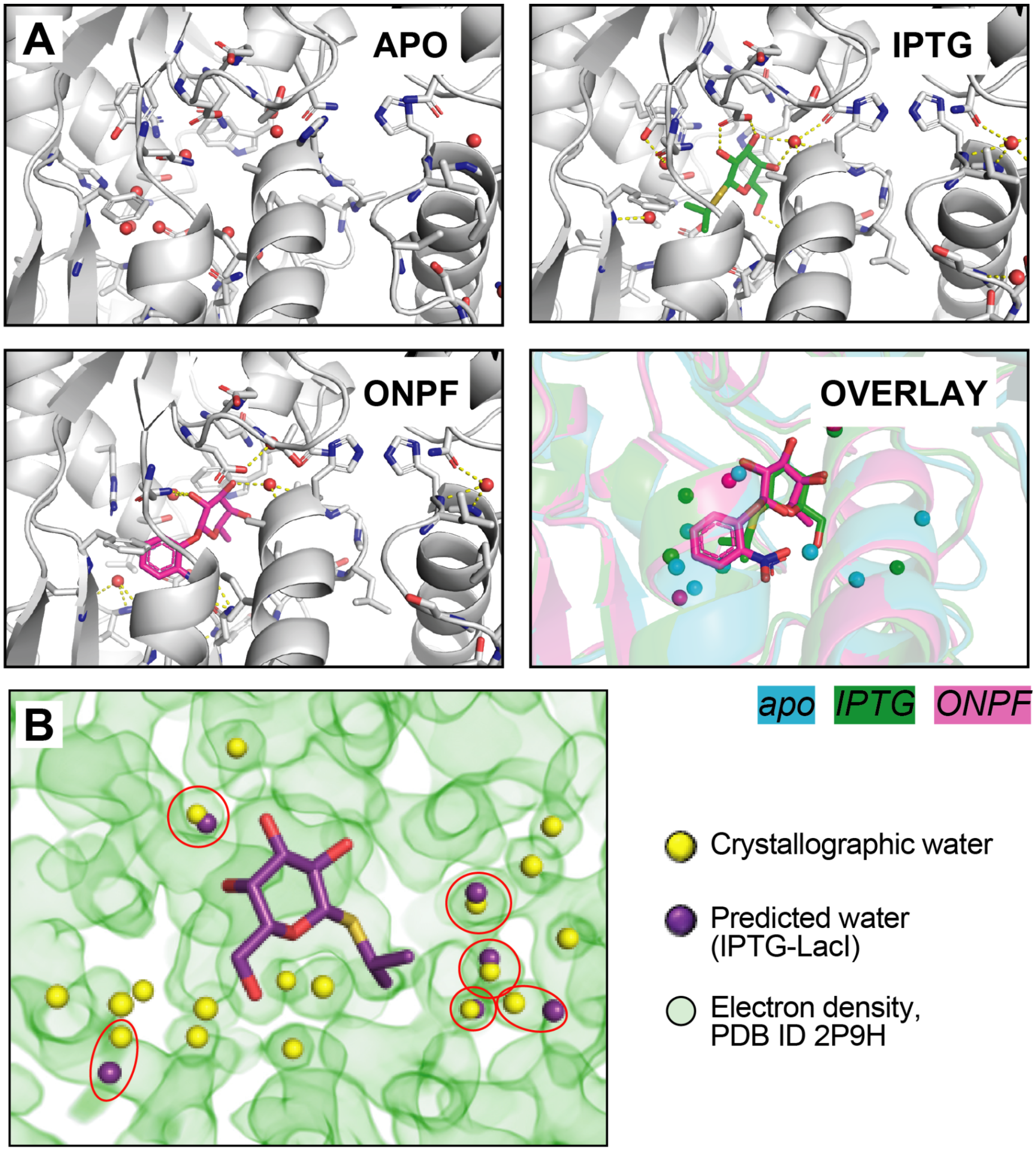

Supplementary Figure 21. Frequency of residue interactions with predicted structural water molecules in different ligand-bound states of LacI. (A) Percentage of simulations in which a predicted structural water molecule is observed within 6 Å of any ligand or protein atom in the binding pocket. Data is shown for both LacI subunits, with and without allowing sidechain repacking (change in rotamer conformation). **(B)** Structural models of apo-LacI, IPTG-LacI, and ONPF-LacI from structural water placement simulations without sidechain repacking (top) and with sidechain repacking (bottom). Sidechains in the ligand binding pocket are colored according to the probability that they make a hydrogen bond with a predicted structural water molecule.

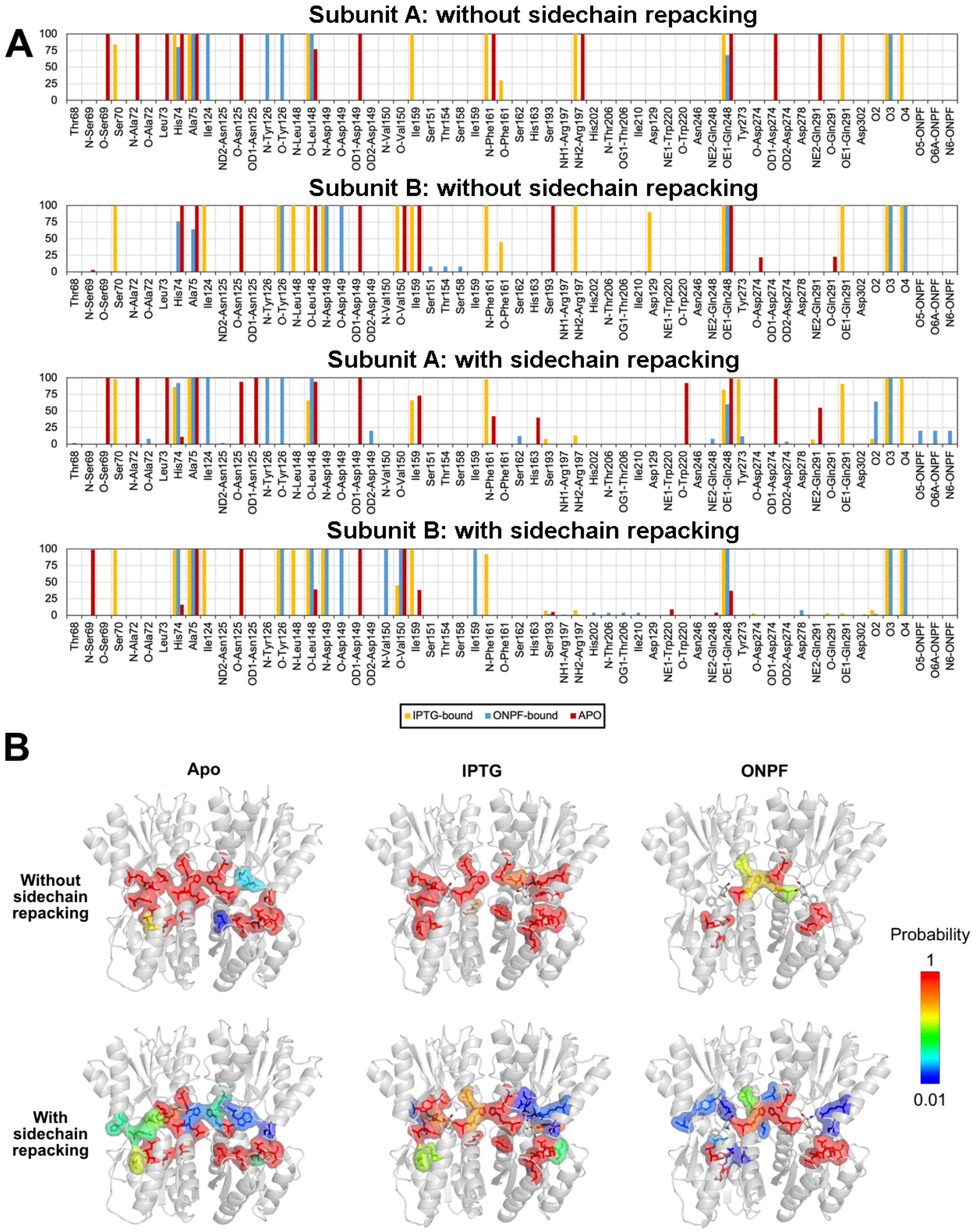

Supplementary Table 1. Mutational phenotypes in LacI. Classification scheme for effects on gene expression of point mutations in the core domain of LacI from Suckow *et al.*[18]

Group	Location, type of amino acid	Remarks
**1**	Solvent exposed	Mostly tolerant to substitutions
**2**	Spacer regions	Tolerant to substitutions; Ala stretch tolerated
**3**	Buried	Tolerant to substitutions
**4**	Solvent exposed	Intolerant to substitutions: involved in salt bridges
**5**	Buried and small	Only small amino acids are tolerated (I^-)
**6**	Solvent exposed and small	Preferably small amino acids are tolerated
**7**	Buried	Intolerant to substitutions (I^-)
**8**	C-terminal subdomain and monomer-monomer interface	Intolerant to substitutions; substitutions result in I^- phenotype
**9**	DNA contacts	Intolerant to substitutions I^-
**10**	Stabilization of the helix-turn-helix motif in the DNA binding domain	Conserved amino acids I^-
**11**	IPTG contacts	Substitutions result in I^s phenotype
**12**	N-terminal subdomain and monomer-monomer interface	Substitutions result in I^s phenotype
**13**	N-terminal subdomain of the core, near the hinge connecting both core domains	Substitutions result in I^s phenotype
**14**	N-terminal subdomain of the core, near the center of the domain	Substitutions result in weak I^s phenotype
**15**	Interface between DNA binding domain A and core B, or the interface between DNA binding domains A and B	Intolerant to substitutions

Supplementary Table 2. Fully deuterated LacI peptides. Percent deuteration observed in maximally labeled HDX/MS controls for the 57 peptides in the curated peptide dataset.

First residue	Last residue	Peptide	%D
63	67	LIGVA	62.375
72	76	ALHAP	53.456
72	81	ALHAPSQIVA	69.843
72	82	ALHAPSQIVAA	66.865
72	84	ALHAPSQIVAAIK	71.838
77	82	SQIVAA	82.949
77	84	SQIVAAIK	88.284
79	84	IVAAIK	91.593
82	93	AIKSRADQLGAS	62.077
87	93	ADQLGAS	57.699
94	98	VVVSM	82.723
98	105	MVERSGVE	52.327
109	114	AAVHNL	54.412
115	122	LAQRVSGL	74.479
119	125	VSGLIIN	84.658
123	129	IINYPLD	75.582
128	135	LDDQDAIA	54.55
134	139	IAVEAA	104.619
139	146	ACTNVPAL	71.379
145	149	ALFLD	64.994
147	158	FLDVSDQTPINS	79.973
152	157	DQTPIN	79.801
158	163	SIIFSH	62.602
158	169	SIIFSHEDGTRL	32.071
164	173	EDGTRLGVEH	54.708
174	177	LVAL	82.243
174	184	LVALGHQQIAL	69.995
177	185	LGHQQIALL	60.627
182	185	IALL	92.705

185	190	LAGPLS	71.843
185	196	LAGPLSSVSARL	46.157
187	195	GPLSSVSAR	77.371
198	203	LAGWHK	50.69
204	212	YLTRNQIQP	85.554
208	219	NQIQPIAEREGD	71.614
213	225	IAEREGDWSAMSG	59.832
216	226	REGDWSAMSGF	60.662
219	223	DWSAM	71.97
220	223	WSAM	81.576
221	227	SAMSGFQ	78.404
224	232	SGFQQTMQM	67.03
228	233	QTMQML	82.819
234	243	NEGIVPTAML	83.32
243	254	LVANDQMALGAM	43.976
244	255	VANDQMALGAMR	73.363
256	267	AITESGLRVGAD	68.841
257	267	ITESGLRVGAD	66.687
266	273	ADISVVGY	86.737
270	275	VVGYDD	48.3
274	290	DDTEDSSCYIPPLTTIK	97.302
276	290	TEDSSCYIPPLTTIK	43.726
279	292	SSCYIPPLTTIKQD	79.388
293	296	FRLL	82.69
293	301	FRLLGQTSV	79.012
296	303	LGQTSVDR	68.554
297	304	GQTSVDRL	70.732
304	307	LLQL	80.627
		Average	70.52989
		Standard deviation	14.61508

Supplementary Table 3. HDX summary table. Information about all HDX/MS data collected in this study, reported as recommended by Masson *et al.*[20]

Data Set	APO, IPTG, DNA, ONPF-DNA	TMG	IPTG-DNA
HDX reaction details	50 mM MOPS, 200 mM NaCl, 0.5 mM TCEP, pH 7.0, 25 °C		50 mM Tris, 150 mM NaCl, pH 8.0, 25 °C
HDX time course	0 sec, 30 sec, 45 sec, 1 min, 5 min, 25 min, 1 hr, 2 hr, 4 hr		0 sec, 30 sec, 16.6 min, and 4 hr
HDX control samples	Maximally-labeled control (WT protein)		none
Back-exchange (mean / IQR)	29.5% / 20.9%		not calculated
# of Peptides	57		199
Sequence coverage	94%		99%
Average peptide length	8.5		11
Replicates (biological or technical)	3+ (biological)	2 (biological)	1
Repeatability	See Supplementary Dataset 3 for all standard deviations for all peptides		n/a
Significant differences in HDX (delta HDX > X D)	0.20 * # exchangeable protons in the peptide D (99% CI)		n/a

Supplementary references

1. Chakerian, A. E., Pfahl, M., Olson, J. S. & Matthews, K. S. A mutant lactose repressor with altered inducer and operator binding parameters. *J Mol Biol* **183**, 43–51 (1985).
2. Chang, W. I., Olson, J. S. & Matthews, K. S. Lysine 84 is at the subunit interface of lac repressor protein. *J Biol Chem* **268**, 17613–17622 (1993).
3. Markiewicz, P., Kleina, L. G., Cruz, C., Ehret, S. & Miller, J. H. Genetic studies of the lac repressor. XIV. Analysis of 4000 altered Escherichia coli lac repressors reveals essential and non-essential residues, as well as 'spacers' which do not require a specific sequence. *J. Mol. Biol.* **240**, 421–433 (1994).
4. Nichols, J. C. & Matthews, K. S. Combinatorial mutations of lac repressor. Stability of monomer-monomer interface is increased by apolar substitution at position 84. *J Biol Chem* **272**, 18550–18557 (1997).
5. Swint-Kruse, L., Zhan, H. & Matthews, K. S. Integrated insights from simulation, experiment, and mutational analysis yield new details of LacI function. *Biochemistry* **44**, 11201–11213 (2005).
6. Lewis, M. The lac repressor. *Comptes Rendus Biologies* **328**, 521–548 (2005).
7. Zhan, H., Camargo, M. & Matthews, K. S. Positions 94-98 of the lactose repressor N-subdomain monomer-monomer interface are critical for allosteric communication. *Biochemistry* **49**, 8636–8645 (2010).
8. Swint-Kruse, L., Zhan, H., Fairbanks, B. M., Maheshwari, A. & Matthews, K. S. Perturbation from a Distance: Mutations that Alter LacI Function through Long-Range Effects. *Biochemistry* **42**, 14004–14016 (2003).
9. Barkley, M. D., Riggs, A. D., Jobe, A. & Bourgeois, S. Interaction of effecting ligands with lac repressor and repressor-operator complex. *Biochemistry* **14**, 1700–1712 (1975).

10. Pavlovicz, R. E., Park, H. & DiMaio, F. Efficient consideration of coordinated water molecules improves computational protein-protein and protein-ligand docking discrimination. *PLOS Computational Biology* **16**, e1008103 (2020).

11. Bell, C. E. & Lewis, M. A closer view of the conformation of the Lac repressor bound to operator. *Nat Struct Biol* **7**, 209–214 (2000).

12. Daber, R., Stayrook, S., Rosenberg, A. & Lewis, M. Structural Analysis of Lac Repressor Bound to Allosteric Effectors. *Journal of Molecular Biology* **370**, 609–619 (2007).

13. Frank, D. E. *et al.* Thermodynamics of the interactions of Lac repressor with variants of the symmetric Lac operator: effects of converting a consensus site to a non-specific site11Edited by P. E. Wright. *Journal of Molecular Biology* **267**, 1186–1206 (1997).

14. Sadler, J. R., Sasmor, H. & Betz, J. L. A perfectly symmetric lac operator binds the lac repressor very tightly. *Proc Natl Acad Sci U S A* **80**, 6785–6789 (1983).

15. Wilson, C. J., Zhan, H., Swint-Kruse, L. & Matthews, K. S. Ligand interactions with lactose repressor protein and the repressor-operator complex: The effects of ionization and oligomerization on binding. *Biophysical Chemistry* **126**, 94–105 (2007).

16. Chen, J. & Matthews, K. S. Subunit dissociation affects DNA binding in a dimeric lac repressor produced by C-terminal deletion. *Biochemistry* **33**, 8728–8735 (1994).

17. Riggs, A. D., Bourgeois, S. & Cohn, M. The lac represser-operator interaction: III. Kinetic studies. *Journal of Molecular Biology* **53**, 401–417 (1970).

18. Suckow, J. *et al.* Genetic Studies of the Lac Repressor XV: 4000 Single Amino Acid Substitutions and Analysis of the Resulting Phenotypes on the Basis of the Protein Structure. *Journal of Molecular Biology* **261**, 509–523 (1996).

19. Weis, D. D. Recommendations for the Propagation of Uncertainty in Hydrogen Exchange-Mass Spectrometric Measurements. *J. Am. Soc. Mass Spectrom.* **32**, 1610–1617 (2021).

20. Masson, G. R. *et al.* Recommendations for performing, interpreting and reporting hydrogen deuterium exchange mass spectrometry (HDX-MS) experiments. *Nat Methods* **16**, 595–602 (2019).
