# Supplementary material for: Ligand-specific changes in conformational flexibility mediate long-range allostery in the lac repressor
Source: Nat Commun. 2023 Mar 2;14:1179. doi: 10.1038/s41467-023-36798-1 (PMC9977783; doi:10.1038/s41467-023-36798-1)
Supplement: Supplementary file 2 — Description of Additional Supplementary Files [file 41467_2023_36798_MOESM2_ESM.pdf]

Description of Additional Supplementary Files

File name: Supplementary Dataset 1.

Description: Fitted HDX parameters for 57 LacI peptides in 6 functional states. Nonlinear regression parameters and errors were calculated for deuterium incorporation in each peptide over 9 timepoints (0 s, 30 s, 45 s, 60 s, 300 s, 1500 s, 3600 s, 7200 s, 14400 s) with backexchange correction, as described in the Methods.

File name: Supplementary Dataset 2.

Description: Calculated uncertainties for the fractional differences at single HX timepoints based on maximum theoretical exchange and corrected for back-exchange.

File name: Supplementary Dataset 3.

Description: Compiled raw HDX/MS data. Averages and standard deviations for MS centroids from HDX/MS technical replicates were calculated for all states at each timepoint. Averaged centroid values were converted to the number of deuterons exchanged given the experimentally observed centroid of the maximally labeled control sample, as described in the Methods.
