# Supplementary material for: Ligand-specific changes in conformational flexibility mediate long-range allostery in the lac repressor
Source: Nat Commun. 2023 Mar 2;14:1179. doi: 10.1038/s41467-023-36798-1 (PMC9977783; doi:10.1038/s41467-023-36798-1)
Supplement: Supplementary file 3 — Supplementary Data 1 [file 41467_2023_36798_MOESM3_ESM.pdf]

LIGVA

	IPTG	ONPF	APO	DNA	TMG	ONPFDNA
**A**	0	0	0	0	0	0
**B**	0.115463185	0.10024544	0.084606856	0.057214699	0.126436209	0.060692427
**C**	2.903053752	0.021290284	2.919684509	0.036125307	2.853918913	2.951084938
**k1**	0	0	0	0	0	0
**k2**	0.1	0.044754511	0.1	0.1	0.060004711	0.1
**k3**	2.06E-06	0.000630601	3.16E-06	0.000178678	2.15E-07	3.90E-06
**NE**	1.07E-09	2.901638814	1.92E-15	2.923818648	1.85E-09	2.54E-13

ALHAP

	IPTG	ONPF	APO	DNA	TMG	ONPFDNA
**A**	0.123193928	0.006262936	1.238802193	1.131581426	0.006546529	1.181419608
**B**	0.351875901	1.172436108	0.11524941	0.183472391	1.094517786	7.25E-07
**C**	1.126203602	3.36E-10	0.139649633	0.025229198	0.700671853	9.97E-34
**k1**	0.524464625	0.04363626	0.533346564	0.106267903	0.000600259	0.121281617
**k2**	0.014963257	0.043605679	0.054518936	0.1	0.022049753	0.1
**k3**	0.000519497	0.009982929	0.001141383	0.003490958	0.000444242	0.009999927
**NE**	0.398726564	0.821997051	0.506305495	0.645219376	0.204051819	0.819977149

ALHAPSQIVA

	IPTG	ONPF	APO	DNA	TMG	ONPFDNA
**A**	0.608610214	1.456936105	1.135677283	1.040043545	1.002925004	0.009847622
**B**	0.689746335	1.577539849	1.647584895	0.997625866	1.010614997	2.290805372
**C**	1.67564032	3.847833538	3.443422379	1.97016621	2.927034271	0.9269073
**k1**	0.025531109	0.039488113	0.033201389	0.070622063	0.031176988	0.052819182
**k2**	0.025534119	0.039488938	0.033209768	0.070622662	0.031180283	0.05275461
**k3**	0.000235924	0.000105089	0.000340287	0.000124227	0.00012261	0.00020674
**NE**	4.026432816	0.243396721	0.731273023	2.974413159	2.127368912	3.838758762

ALHAPSQIVAA

	IPTG	ONPF	APO	DNA	TMG	ONPFDNA
**A**	0.115927425	1.235830587	1.03E-14	0.731672612	5.806072679	2.14E-20

**B**	0.785434444	2.018917173	1.544045727	0.68026695	1.354085478	1.435818163
**C**	1.117347634	4.781128946	1.958549635	0.796386112	0.907194978	0.528713045
**k1**	0.581306417	0.000461237	1.96E-08	0.058609689	1.00E-05	0.068994538
**k2**	0.031246548	0.045103819	0.042840175	0.058604254	0.033399343	0.063122291
**k3**	0.000250091	1.44E-05	0.000886733	0.000194576	0.00020307	0.00022259
**NE**	5.985075983	2.36E-06	4.490601757	5.787843993	5.84E-05	6.038582298

ALHAPSQIVAAIK

	**IPTG**	**ONPF**	**APO**	**DNA**	**TMG**	**ONPFDNA**
**A**	5.70E-08	1.211780028	1.489704313	0.037474194	0.97793931	1.172953579
**B**	1.667429187	2.87128947	0.771586853	2.461995998	1.029258773	1.275651649
**C**	1.97475263	4.20117811	4.801947359	1.12421584	3.173918309	1.297695788
**k1**	3.07E-13	0.028196061	0.695009352	0.062672971	0.0638817	0.090041492
**k2**	0.056266605	0.028184805	0.075704363	0.062676722	0.06386423	0.087466182
**k3**	0.000217541	0.000169244	0.000462763	0.000545103	0.000412208	0.000530332
**NE**	6.371634737	1.820169237	2.954439825	6.456075672	4.886353701	6.301700233

SQIVAA

	**IPTG**	**ONPF**	**APO**	**DNA**	**TMG**	**ONPFDNA**
**A**	0	1.903763247	0.18313929	0	0	0
**B**	0.115957623	0.099598123	0.502763023	0.175895504	0.166778701	0.083152763
**C**	2.654925357	0.172133353	1.009579505	0.155604193	3.788948354	0.052574738
**k1**	0	4.55E-05	0.028969449	0	0	0
**k2**	0.061657834	0.05819577	0.000929625	0.1	0.062244996	0.007090137
**k3**	9.54E-08	0.001306893	7.04E-05	0.000163912	2.72E-06	0.007090759
**NE**	1.2156987	1.665641177	2.304169448	3.704015553	7.46E-05	3.836956277

SQIVAAIK

	**IPTG**	**ONPF**	**APO**	**DNA**	**TMG**	**ONPFDNA**
**A**	0	5.537306063	0.351152842	0	0	0
**B**	0.143640165	0.225000924	0.163409705	0.16920706	0.195456788	0.140838974
**C**	5.066022292	0.230048155	0.952347961	0.221048546	5.815410109	0.109109728
**k1**	0	1.17E-05	0.000931962	0	0	0
**k2**	0.051301531	0.032640504	0.040861788	0.039116795	0.058349578	0.070265966
**k3**	1.13E-07	0.000626699	0.000143032	0.000144413	1.59E-06	0.000364743
**NE**	0.778685584	1.59E-07	4.540086417	5.630376788	1.94E-05	5.750595319

IVAAIK

	**IPTG**	**ONPF**	**APO**	**DNA**	**TMG**	**ONPFDNA**
**A**	0	0	0.052942939	0	0	0

**B**	0.085508162	0.1328334	0.069818316	0.115968651	0.102891276	0.085070831
**C**	2.060572909	0.31938008	0.616697563	0.108795413	3.88914118	0.105973012
**k1**	0	0	0.041627817	0	0	0
**k2**	0.1	0.058227506	0.041623648	0.068043244	0.063996414	0.056323651
**k3**	1.31E-07	0.000304008	0.000309073	0.000138481	1.50E-06	7.33E-05
**NE**	1.841098281	3.52605758	3.262407428	3.780085069	2.23E-11	3.792308186

AIKSRADQLGAS

	**IPTG**	**ONPF**	**APO**	**DNA**	**TMG**	**ONPFDNA**
**A**	0.796523583	0.764854755	0.410636859	0.573195865	0.878778056	0.092981211
**B**	0.909725471	0.81332074	1.148168485	1.803028645	0.801106255	1.392101106
**C**	2.015315075	2.119531443	3.040286173	1.070211474	2.837112475	1.107085914
**k1**	0.052151284	0.034877505	0.923020065	0.00117518	0.056479014	0.053037015
**k2**	0.052152345	0.034878026	0.038469576	0.045574613	0.056464872	0.05303494
**k3**	0.00031234	0.000131416	0.000165173	1.96E-05	0.00020577	0.000571319
**NE**	6.292882309	6.290874352	5.400908483	6.58451683	5.490919886	7.440012269

ADQLGAS

	**IPTG**	**ONPF**	**APO**	**DNA**	**TMG**	**ONPFDNA**
**A**	1.277310383	0.476842341	0.400900755	0.744298362	0.696158716	0.094652199
**B**	6.72E-21	0.508540949	0.850047764	0.614103824	0.686466283	1.492752577
**C**	2.165714948	1.01827824	2.402999313	0.565743345	1.896636338	2.16E-05
**k1**	0.613894278	0.056524963	0.048922639	0.058147887	0.094863476	0.029203938
**k2**	0.099999996	0.05651833	0.048921939	0.058153603	0.094861878	0.029212727
**k3**	0.000552794	0.001108472	0.0001914	0.000914279	0.000438481	0.009984332
**NE**	1.61901846	3.065369674	1.347819149	3.133102958	1.649722921	3.535664345

VVVSM

	**IPTG**	**ONPF**	**APO**	**DNA**	**TMG**	**ONPFDNA**
**A**	0.889374411	0.053846039	0.055554334	7.51E-17	0.439497915	0.750022816
**B**	3.24E-10	1.147233562	1.173432691	1.597353486	0.444208983	0.790756436
**C**	0.879287144	0.000178266	0.386837832	4.98E-14	0.782149771	5.61E-07
**k1**	0.109287069	0.001762475	0.705808477	0.420444554	0.055715628	0.038865706
**k2**	3.02E-05	0.02542359	0.031876867	0.041649717	0.055716125	0.038858113
**k3**	0.000370352	0.009999014	0.001823584	0.01	0.000250272	0.01
**NE**	1.233946022	1.628900383	1.384175143	1.41511241	1.270642131	1.476138362

MVERSGVE

	**IPTG**	**ONPF**	**APO**	**DNA**	**TMG**	**ONPFDNA**
**A**	9.54E-18	3.94E-15	2.02E-24	2.05E-14	3.866699762	3.94E-17

**B**	4.905780467	4.503340327	4.647058998	4.771765542	1.805249269	4.764014171
**C**	7.78E-09	1.47E-12	1.336214495	4.48E-12	1.90E-07	7.71E-21
**k1**	0.354801242	0.076670814	0.005239193	2.96E-07	0.064181297	0.519134169
**k2**	0.064752254	0.044340463	0.070397855	0.048695019	0.064175535	0.051104873
**k3**	0.01	0.01	3.74E-05	0.01	0.009999985	0.01
**NE**	1.09744742	1.446622774	6.23E-17	1.239604972	0.268546386	1.264383838

AAVHNL

	**IPTG**	**ONPF**	**APO**	**DNA**	**TMG**	**ONPFDNA**
**A**	0.373970342	0.30056858	0.627652463	0.294829105	0.510661242	0.04566784
**B**	3.76E-17	0.408254562	0.333422263	0.191088711	0.616100213	0.365927749
**C**	1.972108503	3.214145864	0.847341923	0.196558799	2.832108186	0.170024572
**k1**	0.838801221	0.037395362	0.00020367	0.03323381	0.051234275	0.528135611
**k2**	0.097025636	0.002465809	0.059949222	0.007780216	0.00063238	0.019592558
**k3**	0.00025262	1.54E-05	0.000203578	0.001660453	3.52E-05	0.000760475
**NE**	1.487373452	1.64E-05	2.194568839	3.27775267	1.30E-19	3.300575611

LAQRVSGL

	**IPTG**	**ONPF**	**APO**	**DNA**	**TMG**	**ONPFDNA**
**A**	0.598162865	0.539963789	0.618603179	0.203425174	0.444046563	0.17551411
**B**	0.746344426	0.642884777	0.629120888	0.470578527	1.159581308	0.173256373
**C**	1.494121085	0.457168101	1.358474769	0.265758191	1.53810156	0.51212521
**k1**	0.046263771	0.03431201	0.040964084	0.000461825	0.009576998	0.056828975
**k2**	0.046259652	0.034309149	0.040967333	0.049553999	0.053722125	0.05682989
**k3**	0.000350193	0.000873771	0.000344046	0.000461875	0.00017795	0.000533649
**NE**	3.139432539	4.343436037	3.393865419	5.058401656	2.846823552	5.129212223

VSGLIIN

	**IPTG**	**ONPF**	**APO**	**DNA**	**TMG**	**ONPFDNA**
**A**	0	0	0	0	0	0
**B**	0.189154947	0.135414781	0.156185885	0.112710688	0.223787881	0.079469029
**C**	2.47E-08	3.64E-11	1.49E-12	0.073868366	4.67E-12	0.052615274
**k1**	0	0	0	0	0	0
**k2**	0.061834741	0.056566463	0.1	0.1	0.045156806	0.1
**k3**	5.02E-26	1.35E-25	0.009999994	0.000507169	0.01	0.01
**NE**	4.783193112	4.771411086	4.843578023	4.749795297	4.756545192	4.752238941

IINYPLD

	**IPTG**	**ONPF**	**APO**	**DNA**	**TMG**	**ONPFDNA**
**A**	0.193663035	0.122805744	0.423201595	0	0.19536132	0

**B**	0.028125507	0.13149759	0.165586405	0.164718793	0.005033882	0.185218311
**C**	2.340592524	0.300047402	3.411208632	0.286916018	1.144755849	0.358274546
**k1**	0.114391229	0.030009903	0.000513014	0	0.558051646	0
**k2**	0.012664453	0.030008838	0.080150217	0.081421847	0.017517334	0.058101251
**k3**	1.31E-05	0.000497073	5.00E-06	0.000706389	3.26E-05	0.000630873
**NE**	1.440635444	3.44970887	6.69E-13	3.534418856	2.624016089	3.475327386

LDDQDAIA

	**IPTG**	**ONPF**	**APO**	**DNA**	**TMG**	**ONPFDNA**
**A**	0.020020434	1.97E-24	0.689361077	0.093503677	0.87913265	0.00561492
**B**	1.765892688	1.525721405	1.25627043	1.829493441	1.043446901	1.631870354
**C**	1.075590959	4.391920576	4.054368493	9.19E-08	1.544313663	0.111809676
**k1**	0.08890734	5.73E-05	0.703146201	0.000455895	0.076936021	0.248689726
**k2**	0.088939956	0.057135557	0.036070089	0.054952683	0.076926233	0.067771498
**k3**	0.000156782	2.26E-05	1.97E-05	0.009975747	8.58E-05	0.000549956
**NE**	3.099244354	7.00E-08	2.49E-13	4.097455398	2.505831699	4.255907383

IAVEAA

	**IPTG**	**ONPF**	**APO**	**DNA**	**TMG**	**ONPFDNA**
**A**	0.0465787	0.104715233	0.463397018	0.126898329	0.426937401	0
**B**	0.124269749	0.105425446	0.150015984	0.132783267	0.207174188	0.165867326
**C**	0.964030348	0.332441228	3.37812064	3.67004259	3.35460235	0.280304219
**k1**	0.580332526	0.057364284	0.000937436	0.069623679	0.000619248	0
**k2**	0.029422832	0.057358542	0.041924392	0.06963508	0.050714409	0.042852772
**k3**	0.000298106	0.000639335	7.21E-06	7.83E-06	9.23E-06	0.000595788
**NE**	2.858573734	3.445672008	0.012487874	0.085139981	2.56E-05	3.559497934

ACTNVPAL

	**IPTG**	**ONPF**	**APO**	**DNA**	**TMG**	**ONPFDNA**
**A**	0.364699779	2.04E-39	0.978231958	3.91E-06	0.069231417	8.97E-15
**B**	1.710973271	1.783178422	0.995264638	2.13069922	1.654506492	2.074150143
**C**	0.626247839	3.113098653	0.581525118	0.095817843	0.902524104	2.69E-09
**k1**	0.501317405	0.382436701	0.04916163	0.200339911	0.07035891	0.202837257
**k2**	0.055732053	0.032511023	0.049164264	0.035419348	0.070414716	0.034319749
**k3**	0.000573025	1.45E-05	0.000980126	0.002364933	0.004900921	6.82E-15
**NE**	2.281983477	8.43E-10	2.448489263	2.779518407	2.353178755	2.936764625

ALFLD

	**IPTG**	**ONPF**	**APO**	**DNA**	**TMG**	**ONPFDNA**
**A**	0	0.15087794	0	0	0	0

**B**	0.23389919	4.67E-31	0.196807542	0.300152764	0.339067605	0.285164983
**C**	0.083939947	2.810116311	2.811401434	4.94E-15	1.43E-16	1.07E-11
**k1**	0	0.751636433	0	0	0	0
**k2**	0.058837133	0.009303356	0.1	0.060058588	0.043920054	0.064318355
**k3**	0.000138692	9.17E-06	6.43E-06	0.01	0.00992111	0.01
**NE**	2.66413954	2.50E-06	2.08E-16	2.722627655	2.632379968	2.706146186

FLDVSDQTPINS

	**IPTG**	**ONPF**	**APO**	**DNA**	**TMG**	**ONPFDNA**
**A**	1.017564257	0.089592264	3.725418739	0.626409312	0.792880346	3.89E-05
**B**	3.445625195	2.969562498	3.265938104	4.107879909	4.162204668	4.062761346
**C**	1.236769179	1.986953976	1.92966237	0.938512451	4.134993293	0.993621634
**k1**	0.001384256	0.031538745	2.28E-05	0.000185538	0.001012129	0.053713378
**k2**	0.036866439	0.031526374	0.034438	0.028720234	0.02991451	0.028179203
**k3**	0.000220854	0.000932483	0.001601261	0.000811593	6.87E-05	0.000550167
**NE**	3.349989822	4.079665333	0.0960493	3.441957445	5.63E-06	4.088093832

DQTPIN

	**IPTG**	**ONPF**	**APO**	**DNA**	**TMG**	**ONPFDNA**
**A**	0.187363319	0.92655919	0.430712196	0.107571881	0.568657969	0.513088677
**B**	0.842426595	0.175475644	0.566393125	1.171942155	0.655757543	0.564219611
**C**	0.612223672	1.858670632	0.670790508	0.0001038	0.476657422	0.145236757
**k1**	0.391737753	0.016936288	0.018411769	0.003388128	0.016997567	0.017641928
**k2**	0.011699243	0.002944995	0.018410945	0.019394403	0.016997229	0.017641197
**k3**	0.001152022	2.06E-05	0.001165372	0.01	0.000782871	0.000838086
**NE**	1.36720502	2.42E-05	1.345496304	1.743419736	1.306461096	1.791716565

SIIFSH

	**IPTG**	**ONPF**	**APO**	**DNA**	**TMG**	**ONPFDNA**
**A**	0	0.230293942	2.46E-33	0.13316651	4.95E-31	0.126194363
**B**	0.347829674	0.195958286	0.321957275	0.225035825	0.267592601	0.160917931
**C**	3.748467516	3.641543661	3.682492777	2.594855891	3.536519202	0.304749037
**k1**	0	0.038821274	0.844333445	0.000708488	0.999978135	0.911151028
**k2**	0.078960117	0.038797191	0.034323239	0.020460123	0.1	0.009064348
**k3**	2.23E-06	1.78E-05	2.20E-05	3.11E-06	4.64E-06	6.40E-05
**NE**	8.46E-13	0.000222259	1.91E-13	0.932168122	2.07E-06	3.136016429

SIIFSHEDGTRL

	**IPTG**	**ONPF**	**APO**	**DNA**	**TMG**	**ONPFDNA**
**A**	7.87E-21	0.597997908	0.473058106	0.320252847	0.612440301	0.291815217

**B**	0.644375436	0.383039418	0.463240647	0.308504066	0.629329747	0.284011368
**C**	0.935099002	9.03731504	4.228375675	0.426640156	2.927329924	0.164005941
**k1**	0.003798515	0.999973223	0.029320224	0.05810532	0.041928044	0.046237008
**k2**	0.043295386	0.000876652	0.029326626	0.058112895	0.041920509	0.046241334
**k3**	2.69E-05	7.41E-06	5.66E-05	0.00011134	5.36E-06	0.000381893
**NE**	8.165991582	8.42E-14	4.824183864	8.965576376	5.891689088	9.262060228

EDGTRLGVEH

	**IPTG**	**ONPF**	**APO**	**DNA**	**TMG**	**ONPFDNA**
**A**	0.648486304	6.29E-37	0.245191416	0.118025008	0.282850366	0.406508145
**B**	5.38E-16	0.569295351	0.225543262	0.454355339	0.26195077	0.129099048
**C**	1.83E-15	7.409614422	7.543303528	0.104830718	0.195781134	0.158678107
**k1**	0.857651676	0.999995578	0.070126411	0.060911268	0.060538502	0.73197984
**k2**	0.1	0.064054484	0.070096235	0.060903984	0.060564154	0.1
**k3**	0.01	9.89E-06	1.90E-05	0.000378485	0.001764975	0.000273685
**NE**	7.314992793	1.16E-09	1.66E-06	7.356001128	7.178356778	7.317247617

LVAL

	**IPTG**	**ONPF**	**APO**	**DNA**	**TMG**	**ONPFDNA**
**A**	0	0	0	0	0	0
**B**	0.059621227	0.055672589	0.037509119	0.058649798	0.102930432	0.050676079
**C**	0.580934104	1.94291822	1.960561225	0.094293327	1.899922927	0.066991936
**k1**	0	0	0	0	0	0
**k2**	0.050860871	0.073395644	0.1	0.064494529	0.054794493	0.060336866
**k3**	2.43E-05	5.55E-06	1.20E-05	0.000132985	4.62E-06	0.000247882
**NE**	1.359652403	7.12E-08	7.65E-16	1.848147295	2.71E-06	1.885867196

LVALGHQQIAL

	**IPTG**	**ONPF**	**APO**	**DNA**	**TMG**	**ONPFDNA**
**A**	1.45E-13	0.758287515	4.06E-39	0.646405145	4.14E-37	0.811850664
**B**	1.684423368	0.683407196	1.511241013	0.981890447	1.904395205	0.829936302
**C**	0.908838213	1.170578031	7.490699234	0.178735409	7.074580815	7.10E-07
**k1**	0.174794019	0.039174421	0.828670689	0.050265355	0.153879979	0.045657633
**k2**	0.069153592	0.039167336	0.070233447	0.050266884	0.054065883	0.045644847
**k3**	0.00011544	1.73E-05	1.16E-05	0.000497698	6.83E-06	0.009999913
**NE**	6.395382322	6.293111623	2.41E-14	7.171818104	2.76E-09	7.365406305

LGHQQIALL

	**IPTG**	**ONPF**	**APO**	**DNA**	**TMG**	**ONPFDNA**
**A**	0.555016987	1.92E-13	0.239806071	0.001716008	0.000498243	1.37E-21

**B**	0.649382542	0.932510005	0.773292328	1.262086989	1.515846641	1.242013591
**C**	1.10E-08	0.345555112	0.138966262	1.08E-14	1.11E-11	8.01E-19
**k1**	0.061276226	0.233259794	0.638144953	1.50E-11	0.047856806	0.999994513
**k2**	0.061275571	0.040137927	0.035491504	0.045154644	0.047810461	0.046422921
**k3**	0.00999939	6.30E-05	0.000221177	0.01	0.009999865	2.32E-05
**NE**	5.753649447	5.560332755	5.754185337	5.698565175	5.436398477	5.7565278

IALL

	**IPTG**	**ONPF**	**APO**	**DNA**	**TMG**	**ONPFDNA**
**A**	0	0	0	0	0	0
**B**	0.044403303	0.03475756	0.025537734	0.037737075	0.057343316	0.042997412
**C**	3.68E-07	1.996181297	1.979511973	1.983777643	1.69E-06	0.009789165
**k1**	0	0	0	0	0	0
**k2**	0.058207591	0.1	0.1	0.1	0.062770274	0.1
**k3**	1.74E-17	5.92E-07	1.37E-06	9.98E-07	3.99E-19	2.79E-17
**NE**	1.976181682	7.18E-09	1.65E-08	1.65E-10	1.971981519	1.998815444

LAGPLS

	**IPTG**	**ONPF**	**APO**	**DNA**	**TMG**	**ONPFDNA**
**A**	0.030410272	2.45E-14	0.00069302	0.952854304	5.20E-19	0.319363189
**B**	1.045284228	1.119738663	1.027895334	0.393654032	1.279377435	0.87358147
**C**	0.73890566	0.511206276	0.762190077	1.669809223	0.75783735	6.15E-07
**k1**	0.069819103	0.140057622	0.000980188	0.05571887	0.051528942	0.018148255
**k2**	0.069777956	0.034217355	0.060700663	0.005011054	0.044256391	0.053344885
**k3**	0.000735776	0.000916143	0.000978015	1.06E-05	0.000370646	0.01
**NE**	1.184566691	1.414230523	1.211568475	1.64E-10	0.997448336	1.841506943

LAGPLSSVSARL

	**IPTG**	**ONPF**	**APO**	**DNA**	**TMG**	**ONPFDNA**
**A**	1.54050119	0.853503947	0.541270942	0.019027757	0.890859498	3.37E-06
**B**	1.670762808	3.443687937	4.182111694	4.922694511	2.791709296	4.970964098
**C**	3.191916954	1.460527929	1.969824993	0.441242614	4.34616904	0.937156962
**k1**	0.024857872	0.02779967	0.027926206	0.001837496	0.038149495	0.171877808
**k2**	0.024859913	0.027791112	0.027925773	0.028971166	0.038139096	0.029453678
**k3**	0.000328499	0.001769204	0.000192352	0.001832589	0.000294522	0.000968049
**NE**	2.861074957	3.383659887	2.429024358	3.217628024	1.152934638	3.358790693

GPLSSVSAR

	**IPTG**	**ONPF**	**APO**	**DNA**	**TMG**	**ONPFDNA**
**A**	0.859443238	1.440520906	0.256128644	1.759420358	1.850543745	1.402688979

**B**	0.916876224	1.698264101	2.49157497	1.692970158	0.334364827	1.370503254
**C**	2.216572758	3.23E-05	1.949815168	1.24E-05	3.283085193	0.673918648
**k1**	0.04144469	0.027389273	0.051798035	0.031866851	0.038870426	0.03486246
**k2**	0.041444283	0.02737765	0.051799576	0.031860452	0.004578258	0.034872652
**k3**	0.00057292	0.01	0.000159213	0.01	0.000228332	0.000929605
**NE**	2.988429908	3.822166655	2.302200136	3.557201013	1.497944506	3.554651635

LAGWHK

	**IPTG**	**ONPF**	**APO**	**DNA**	**TMG**	**ONPFDNA**
**A**	0	0	2.23E-39	0	0	0
**B**	0.332800966	0.264157666	0.246481681	0.221216944	0.282667992	0.35791492
**C**	5.45E-14	1.18E-12	3.745898793	0.000739596	0.138784878	6.53E-09
**k1**	0	0	0.999932028	0	0	0
**k2**	0.1	0.041406634	0.091281845	0.1	0.06006399	0.1
**k3**	0.01	2.24E-05	4.86E-06	0.003903854	0.01	0.01
**NE**	3.62705792	3.695817726	9.61E-07	3.623947163	3.58759062	3.717669977

YLTRNQIQP

	**IPTG**	**ONPF**	**APO**	**DNA**	**TMG**	**ONPFDNA**
**A**	0.772915696	0.823031392	1.413802731	1.320235883	0.832798477	0.481425364
**B**	0.78865275	0.865668883	1.263338	0.692660105	0.896194205	0.933613179
**C**	1.923320569	1.611585743	1.875704669	4.002754741	2.143303399	2.605306363
**k1**	0.048197007	0.026081991	0.001214568	0.048602319	0.037344915	0.042890946
**k2**	0.048196523	0.026081929	0.05178588	0.001778038	0.03734505	0.042892918
**k3**	0.000379582	0.000102417	4.07E-05	3.35E-05	0.000299082	0.000128098
**NE**	2.510158832	2.723215774	1.454130862	4.75E-10	2.131013725	2.003221669

NQIQPIAEREGD

	**IPTG**	**ONPF**	**APO**	**DNA**	**TMG**	**ONPFDNA**
**A**	0.669673008	1.032057982	0.047063393	1.181268698	3.85E-22	0.000365378
**B**	2.425017651	1.541846783	2.749185435	1.214702902	3.317408862	2.306204608
**C**	1.835814737	6.392284058	2.676683655	0.537114542	3.12762041	0.823992712
**k1**	0.054819362	0.04423923	0.590934514	0.072178845	0.998936591	0.048981502
**k2**	0.054816078	0.044235375	0.056398782	0.072165825	0.046119752	0.081628067
**k3**	9.52E-05	1.98E-05	8.37E-05	0.001360318	3.37E-05	0.000400764
**NE**	4.039241446	0.000284915	3.518275592	6.070342562	2.546907332	5.852013244

IAEREGDWSAMSG

	**IPTG**	**ONPF**	**APO**	**DNA**	**TMG**	**ONPFDNA**
**A**	1.09278301	1.21324451	1.525813483	1.389419915	2.283963192	1.280033604

**B**	3.289359918	1.821159768	1.658426594	1.310298408	2.435755513	1.333949032
**C**	0.603330535	0.77327294	2.08918821	1.222545508	1.343981752	1.272523727
**k1**	0.853007346	0.027327018	0.031787583	0.036627746	0.02688829	0.035344709
**k2**	0.022173356	0.027320879	0.03178888	0.036628342	0.026889124	0.03535311
**k3**	0.000853081	0.001773977	0.000750966	0.002429563	5.42E-05	0.00075032
**NE**	6.05311072	7.168555489	5.738771143	7.100340932	4.942591622	7.155596381

REGDWSAMSGF

	**IPTG**	**ONPF**	**APO**	**DNA**	**TMG**	**ONPFDNA**
**A**	6.28E-31	0.732037422	1.05908464	0.631748442	3.44E-33	0.789180229
**B**	2.283411719	0.806877634	1.674944863	0.864890449	2.880992748	0.771320489
**C**	0.810340114	1.589343925	6.2915073	0.858255045	0.866981987	1.277754857
**k1**	0.314372789	0.038187747	0.001704235	0.027450482	0.816457675	0.026007743
**k2**	0.022862292	0.038187018	0.021599762	0.027446838	0.023334847	0.026004962
**k3**	0.001277868	0.001001285	7.64E-06	0.001666991	0.001046827	0.00069767
**NE**	6.002661283	5.863697457	0.002224032	6.735176851	5.468083675	6.489419656

DWSAM

	**IPTG**	**ONPF**	**APO**	**DNA**	**TMG**	**ONPFDNA**
**A**	0.306059729	0.522145805	0.497212042	0.644552371	0.506678855	0.297833783
**B**	1.188960277	0.753403511	0.467130178	0.624932431	1.068448002	0.349213547
**C**	0.265847906	1.776753031	0.879089002	1.789904127	0.386543081	0.668592321
**k1**	0.861845153	0.002628106	0.015718459	0.021599515	0.017249188	0.018046987
**k2**	0.011230655	0.018799913	0.015716134	0.002883016	0.017243682	0.018048103
**k3**	0.000694447	2.76E-05	0.001011156	2.27E-05	0.000143797	0.000868555
**NE**	1.266828029	2.07E-06	1.167028032	3.41E-06	1.088867192	1.750638766

WSAM

	**IPTG**	**ONPF**	**APO**	**DNA**	**TMG**	**ONPFDNA**
**A**	0.229657155	0.362994763	0.647811775	0.328764125	0.096097743	0.25035886
**B**	1.051219904	0.341060491	0.349477362	0.408365515	1.140120974	0.283869584
**C**	0.212629311	0.618517686	0.520309636	0.545114422	0.342006154	0.654359563
**k1**	0.922106483	0.017537918	0.021435056	0.768782289	0.837623399	0.021718917
**k2**	0.011810276	0.017535662	0.002245761	0.002574208	0.017308744	0.021712774
**k3**	0.000786665	0.00089846	0.002245773	0.002574298	0.000528667	0.000753177
**NE**	0.505837593	0.660404445	0.492763925	0.678365804	0.390884561	0.853832355

SAMSGFQ

	**IPTG**	**ONPF**	**APO**	**DNA**	**TMG**	**ONPFDNA**
**A**	0.817895584	0.488928341	0.772376281	0.489605476	0.049530513	9.33E-08

**B**	0.892663227	0.516437532	1.26275698	0.545512549	1.878784545	0.882408839
**C**	0.599651819	0.922003875	2.971475734	0.861108989	0.330285175	0.943807137
**k1**	0.028718308	0.023907666	0.035168795	0.032169218	8.61E-05	0.057005631
**k2**	0.028718959	0.023903645	0.002971568	0.032163493	0.025942288	0.030835896
**k3**	0.002226156	0.001451563	6.82E-06	0.001097514	0.000451116	0.000740391
**NE**	2.769831695	3.100458129	1.14E-17	3.139506399	2.692927345	3.200659784

SGFQQTMQM

	**IPTG**	**ONPF**	**APO**	**DNA**	**TMG**	**ONPFDNA**
**A**	0.201458592	0.149073884	0.197696442	0	0.154255961	0
**B**	1.69E-22	0.086564563	0.009492574	0.156635046	0.146005365	0.184369685
**C**	3.903102556	5.988255194	3.682442956	6.801094187	6.657740446	0.073029108
**k1**	0.126441893	0.069615403	0.067822636	0	0.052017936	0
**k2**	0.092596978	0.069603303	8.23E-06	0.044591317	0.051997947	0.066931362
**k3**	5.79E-06	2.65E-06	8.20E-06	2.37E-06	3.07E-06	0.000296931
**NE**	2.893916274	0.713449751	3.11908714	1.14E-09	0.002448401	6.721291935

QTMQML

	**IPTG**	**ONPF**	**APO**	**DNA**	**TMG**	**ONPFDNA**
**A**	0	0	0	0	0	0
**B**	0.067733126	0.068668748	0.065706128	0.096485739	0.095350899	0.089465891
**C**	1.103119401	3.933559802	3.938716518	0.058510662	3.922683359	0.055010039
**k1**	0	0	0	0	0	0
**k2**	0.074468762	0.051269459	0.037935455	0.062834292	0.06442549	0.041532834
**k3**	1.65E-05	2.94E-06	5.45E-06	0.000173948	3.65E-06	0.000262549
**NE**	2.836894144	1.01E-08	2.71E-16	3.88200463	4.72E-10	3.882709071

NEGIVPTAML

	**IPTG**	**ONPF**	**APO**	**DNA**	**TMG**	**ONPFDNA**
**A**	0.499353769	0.558750093	0.8760085	0.503253667	0.591599884	0.44438584
**B**	0.442723526	0.544540385	1.263623642	0.651568355	0.651315857	0.554419566
**C**	1.549133499	1.267787051	4.879299909	0.734591292	1.406102797	1.008811422
**k1**	0.037484018	0.023562684	0.036860544	0.033360153	0.033155472	0.034245481
**k2**	0.037484006	0.023560477	0.001292489	0.033361642	0.033158397	0.034243508
**k3**	0.000761687	0.000192215	1.02E-05	0.001076975	0.000426153	0.00070928
**NE**	4.482152523	4.524667478	0.000775835	5.083958962	4.330783267	5.009898286

LVANDQMALGAM

	**IPTG**	**ONPF**	**APO**	**DNA**	**TMG**	**ONPFDNA**
**A**	9.84E-07	0.664527344	7.39E-15	0.770978811	0.61895426	0.221318456

**B**	1.271630028	1.34218574	1.25644212	0.829609451	0.008959238	2.05691936
**C**	0.673619656	7.872920739	1.11009661	0.880002688	0.113892347	0.793765169
**k1**	0.000131001	0.000353361	0.246300028	0.046421671	0.995395282	0.945482152
**k2**	0.039658422	0.041544042	0.041377504	0.04642527	0.099999999	0.028469744
**k3**	0.000146389	2.87E-06	0.000249286	0.000155719	0.000263815	0.000999426
**NE**	8.066368391	0.003726608	7.615214706	7.484030505	9.337099454	6.483744517

VANDQMALGAMR

	**IPTG**	**ONPF**	**APO**	**DNA**	**TMG**	**ONPFDNA**
**A**	0.777093139	0.412767204	0.680011682	1.95E-06	0.771783762	2.59E-09
**B**	1.14E-10	0.323850227	0.094776717	0.772702924	0.152320104	0.663216695
**C**	0.249747264	0.310418385	3.247746174	0.357993604	7.880379133	0.363972754
**k1**	0.101751627	0.04647227	0.040219879	7.57E-20	0.091744149	0.284557692
**k2**	0.058665503	0.046471162	0.1	0.062602804	0.00298608	0.072484508
**k3**	0.000382351	0.000419209	2.59E-05	0.000331344	2.69E-06	0.000325297
**NE**	8.980481576	8.893479145	5.990157253	8.882640228	1.185968765	8.982081063

AITESGLRVGAD

	**IPTG**	**ONPF**	**APO**	**DNA**	**TMG**	**ONPFDNA**
**A**	8.67E-13	0.068686367	1.401215382	8.65E-05	2.769032201	1.163308342
**B**	2.863265144	2.29091339	1.152813709	2.449359492	0.203557686	1.196269467
**C**	1.195812313	2.98844917	7.450265431	0.154920531	6.99133431	0.199728929
**k1**	0.094512125	0.034553433	0.053231167	0.056599668	0.044938924	0.049701018
**k2**	0.058299347	0.034531923	0.053231582	0.048733824	0.044946327	0.049634128
**k3**	0.000109479	6.18E-06	1.55E-05	0.001405322	1.03E-05	0.000501596
**NE**	5.942626373	4.528080441	0.001255561	7.368005745	0.000424431	7.445686452

ITESGLRVGAD

	**IPTG**	**ONPF**	**APO**	**DNA**	**TMG**	**ONPFDNA**
**A**	5.74E-05	2.17E-38	0.02377937	1.45E-34	7.45E-36	0.087911409
**B**	2.777345383	2.306987164	2.502435343	2.592285727	2.968377184	2.506166677
**C**	1.333638442	6.66342357	2.613240002	6.454632899	5.349668855	0.005965124
**k1**	5.80E-19	0.999976535	0.049993062	0.8563855	0.90711752	0.000330476
**k2**	0.053076179	0.041279201	0.049985322	0.041331451	0.047531842	0.048498131
**k3**	7.22E-05	7.54E-06	4.22E-05	1.59E-06	1.39E-05	0.009999464
**NE**	4.951148638	1.17E-07	3.885743066	0.002963174	0.649661005	6.505721618

ADISVVGY

	**IPTG**	**ONPF**	**APO**	**DNA**	**TMG**	**ONPFDNA**
**A**	0	0	0.188003895	0	0	0

**B**	0.250685123	0.250124534	1.44E-23	0.236359397	0.325807978	0.241159565
**C**	1.27E-17	1.35E-15	3.74887402	1.24E-10	0.001984495	8.40E-10
**k1**	0	0	0.998187931	0	0	0
**k2**	0.1	0.060997781	0.070085065	0.099999998	0.048994472	0.1
**k3**	0.01	0.01	4.86E-06	0.01	0.00252465	1.55E-15
**NE**	5.728404055	5.725429641	2.058267716	5.752750557	5.656644204	5.761757141

VVGYDD

	**IPTG**	**ONPF**	**APO**	**DNA**	**TMG**	**ONPFDNA**
**A**	0	0	0.361217868	0	8.80E-13	0
**B**	0.509111788	0.444089118	8.83E-24	0.396418528	0.566667113	0.378578257
**C**	4.12E-17	0.036964802	3.633308416	0.033412104	0.072768251	2.07E-17
**k1**	0	0	0.76764394	0	0.910958338	0
**k2**	0.1	0.075193887	0.099999313	0.1	0.082468379	0.1
**k3**	0.01	0.000778152	1.25E-05	0.001257325	0.006595416	0.01
**NE**	3.479103865	3.51536697	2.63E-07	3.582918685	3.361801284	3.630268415

DDTEDSSCYIPPLTTIK

	**IPTG**	**ONPF**	**APO**	**DNA**	**TMG**	**ONPFDNA**
**A**	1.254318015	0.711136491	1.137629332	0.000777005	0.994361263	0.003499609
**B**	0.971889241	0.858260932	2.018220641	0.9627777	0.870949204	0.976371651
**C**	1.976806505	4.498190745	9.849176788	0.301747965	2.470465535	0.276954691
**k1**	0.041613426	0.03661087	0.050381563	0.216598986	0.029115321	0.60581454
**k2**	0.003108388	0.03661014	0.000826123	0.062922765	0.029112518	0.070278791
**k3**	0.000299264	0.000117216	2.02E-05	0.002178815	0.000293302	0.000672477
**NE**	8.822910939	6.973983343	2.25E-18	11.78344966	8.697763017	11.80225004

TEDSSCYIPPLTTIK

	**IPTG**	**ONPF**	**APO**	**DNA**	**TMG**	**ONPFDNA**
**A**	1.556158942	0.905079637	1.030343206	0.441543018	1.196164657	0.379321104
**B**	0.809604118	0.974444615	2.896849146	0.489022162	0.981607126	0.389762086
**C**	3.910578968	6.024632724	5.979150867	0.462655758	4.620472821	0.554258046
**k1**	0.034007018	0.029547049	0.045930275	0.048652283	0.034876185	0.067820847
**k2**	0.001817886	0.029541352	0.00095218	0.048647935	0.034876224	0.067864963
**k3**	0.000393407	0.000121969	8.19E-05	0.002200478	0.000237001	0.000775912
**NE**	4.802841215	3.164265721	1.104426569	9.7124752	4.240824452	9.701689373

SSCYIPPLTTIKQD

	**IPTG**	**ONPF**	**APO**	**DNA**	**TMG**	**ONPFDNA**
**A**	0.130810187	0.642413033	0.823054684	0.00021676	0.761662413	0.375536537

**B**	0.967598641	0.299906808	0.987173756	0.76784736	0.829350946	0.296699986
**C**	1.691973435	1.489285742	4.722252288	0.044575752	3.394668487	0.115675819
**k1**	0.74434456	0.051072324	0.066054389	0.09767127	0.043331748	0.056387979
**k2**	0.038955714	0.0510693	0.000824975	0.050729061	0.043326971	0.056504494
**k3**	0.000307053	0.000393708	5.90E-05	0.001113702	4.54E-05	0.000574887
**NE**	7.199014689	7.542752085	3.469629204	9.163341998	5.058566125	9.197139363

FRLL

	**IPTG**	**ONPF**	**APO**	**DNA**	**TMG**	**ONPFDNA**
**A**	0.110105893	0	0.076546111	0	0.108237439	0
**B**	0.066996739	0.100867429	0.256463103	0.08420405	0.06257733	0.063455588
**C**	0.59712447	0.272010478	1.668627967	0.205558266	0.736102452	0.157206027
**k1**	0.00038976	0	0.056945022	0	0.046334155	0
**k2**	0.065844227	0.059710012	0.000820678	0.064540749	0.046327873	0.064289434
**k3**	0.000389705	0.000377778	2.26E-05	0.000242122	0.000139956	0.000391565
**NE**	1.21977678	1.621227098	9.86E-06	1.714069599	1.105613665	1.781732619

FRLLGQTSV

	**IPTG**	**ONPF**	**APO**	**DNA**	**TMG**	**ONPFDNA**
**A**	0.139736718	0.192301081	0.217320196	0.18431024	0.238234793	0
**B**	0.304726555	0.212774957	0.239855393	0.122859513	0.230572895	0.121385781
**C**	0.689332146	3.760681575	1.978761923	0.711898237	3.38538576	0.176937078
**k1**	0.896304155	0.0392138	0.040312634	0.057785273	0.054147405	0
**k2**	0.034869593	0.039202939	0.040312145	0.057788315	0.054141733	0.094787671
**k3**	0.000268502	1.91E-05	5.23E-05	8.50E-05	1.78E-05	0.000347577
**NE**	5.8966946	2.874319436	4.572492082	6.066823556	3.215708387	6.585360841

LGQTSVDR

	**IPTG**	**ONPF**	**APO**	**DNA**	**TMG**	**ONPFDNA**
**A**	5.00E-39	3.03E-08	0.401804817	0	0.072945415	0
**B**	0.499979276	0.437077541	4.68E-15	0.426872423	0.589450811	0.394185904
**C**	4.652834157	0.097970795	5.598232396	0.031760722	2.280335178	0.038096873
**k1**	0.855896632	0.977514284	0.129523616	0	0.068171797	0
**k2**	0.090333269	0.062588725	9.52E-06	0.075104944	0.068162898	0.1
**k3**	2.96E-07	0.000343452	8.15E-06	0.0002399	1.34E-06	0.000312438
**NE**	0.82514454	5.438054166	1.73E-06	5.552239898	3.051786285	5.573989807

GQTSVDRL

	**IPTG**	**ONPF**	**APO**	**DNA**	**TMG**	**ONPFDNA**
**A**	0	0	0.292953513	0	0	0

**B**	0.33451389	0.320702877	0.022253958	0.32717771	0.452396532	0.285105524
**C**	0.027192927	0.010336733	5.67734544	0.030290974	5.82E-09	6.81E-08
**k1**	0	0	0.845399583	0	0	0
**k2**	0.066486252	0.048971692	0.045401786	0.058643081	0.063971803	0.099999999
**k3**	0.01	0.001793366	4.05E-06	0.003851939	4.15E-13	1.02E-09
**NE**	5.596515114	5.634517757	2.92E-06	5.64880139	5.560479158	5.70858039

LLQL

	**IPTG**	**ONPF**	**APO**	**DNA**	**TMG**	**ONPFDNA**
**A**	0.424998015	0.223092342	0.557166438	0.255350703	0.261636585	0.191567915
**B**	0.179738592	0.246744715	0.73088921	0.282527942	0.623866593	0.237726609
**C**	0.920235119	0.417486013	0.731379744	0.520407526	0.765584232	0.509704477
**k1**	0.02958332	0.021901569	0.023639393	0.027449606	0.109529557	0.023587116
**k2**	0.029586161	0.021901531	0.000893312	0.027447244	0.008533518	0.023584066
**k3**	0.000632798	0.001075579	1.85E-05	0.001369187	0.000223998	0.000887792
**NE**	0.48099988	1.110742548	3.14E-13	0.971072018	0.345807287	1.09987796
