# Supplementary material for: Ligand-specific changes in conformational flexibility mediate long-range allostery in the lac repressor
Source: Nat Commun. 2023 Mar 2;14:1179. doi: 10.1038/s41467-023-36798-1 (PMC9977783; doi:10.1038/s41467-023-36798-1)
Supplement: Supplementary file 4 — Supplementary Data 2 [file 41467_2023_36798_MOESM4_ESM.pdf]

APO vs DNA

Peptide	0 s	30 s	45 s	60 s	300 s	1500 s	3600 s	7200 s	14400 s
LIGVA	3.32E-06	3.62E-05	1.75E-05	3.79E-05	5.77E-05	3.22E-06	3.48E-06	8.91E-06	4.94E-05
ALHAP	1.51E-06	2.41E-04	3.08E-04	5.53E-04	9.37E-04	2.91E-04	4.98E-04	8.55E-04	nan
ALHAPSQIVA	2.50E-06	3.31E-05	nan	1.00E-04	4.85E-05	3.20E-05	1.73E-04	1.06E-04	5.26E-05
ALHAPSQIVAA	7.63E-06	7.02E-05	2.24E-04	1.27E-04	1.39E-04	6.40E-05	2.49E-04	4.38E-04	1.65E-03
ALHAPSQIVAAIK	5.04E-06	nan	2.63E-04	1.61E-04	3.36E-05	6.40E-05	6.36E-04	7.15E-04	2.24E-03
SQIVAA	2.42E-06	1.76E-05	1.38E-05	8.87E-05	7.88E-05	3.28E-04	1.14E-06	8.25E-04	5.31E-04
SQIVAAIK	4.22E-06	1.91E-05	nan	1.78E-04	2.32E-05	4.54E-04	9.74E-05	3.09E-04	5.38E-04
IVAAIK	5.93E-06	4.25E-06	5.01E-05	1.41E-04	5.73E-06	2.18E-04	5.05E-05	8.61E-05	5.14E-04
AIKSRADQLGAS	2.30E-06	1.36E-04	3.38E-04	1.67E-04	2.29E-04	1.50E-04	3.06E-04	3.83E-04	1.19E-03
ADQLGAS	1.28E-05	3.90E-05	1.17E-03	3.14E-04	2.14E-05	1.78E-04	3.00E-04	nan	8.32E-03
VVVSM	8.41E-07	3.62E-04	7.40E-04	2.09E-04	1.17E-03	2.93E-04	4.68E-04	1.26E-03	1.24E-03
MVERSGVE	6.49E-07	9.14E-04	nan	1.35E-03	1.16E-03	7.61E-04	1.19E-03	1.11E-03	9.17E-04
AAVHNL	2.54E-05	1.95E-05	2.20E-04	6.18E-07	2.52E-04	3.60E-06	4.50E-04	2.08E-03	7.94E-04
LAQRVSGL	1.82E-07	4.53E-05	2.78E-04	2.23E-06	1.15E-04	2.95E-06	1.82E-04	3.58E-04	7.66E-04
VSGLIIN	5.91E-05	1.79E-05	6.37E-05	9.76E-05	2.61E-05	9.35E-06	1.38E-04	1.57E-04	1.28E-05
IINYPLD	5.30E-06	6.46E-06	1.24E-05	2.40E-06	2.10E-05	1.49E-05	4.05E-05	1.18E-04	7.46E-04
LDDQDAIA	5.91E-08	5.49E-04	1.07E-03	8.62E-04	nan	2.27E-04	1.05E-03	1.50E-03	3.16E-03
IAVEAA	5.91E-07	nan	8.96E-04	2.01E-04	5.28E-05	1.45E-04	1.72E-04	4.42E-04	3.04E-04
ACTNVPAL	2.98E-05	6.74E-04	1.17E-03	2.16E-04	1.55E-03	1.87E-04	6.14E-04	2.47E-03	4.09E-03
ALFLD	3.64E-08	3.75E-05	1.61E-04	1.70E-05	4.56E-05	3.14E-05	5.91E-05	1.79E-05	1.21E-04
FLDVSDQTPINS	8.28E-06	9.66E-05	nan	2.85E-04	1.35E-04	3.71E-04	3.07E-04	5.20E-04	1.94E-04
DQTPIN	5.40E-06	7.40E-06	4.66E-04	4.79E-05	6.41E-04	1.69E-05	4.96E-04	8.56E-04	2.33E-03
SIIFSH	3.77E-05	1.54E-04	3.28E-04	1.85E-04	nan	2.20E-04	3.48E-04	3.79E-04	9.78E-04
SIIFSHEDGTRL	1.96E-06	2.06E-05	1.09E-05	1.82E-05	3.60E-05	3.93E-05	5.78E-05	7.56E-05	1.66E-04
EDGTRLGVEH	5.50E-06	2.75E-05	1.25E-05	3.61E-05	6.02E-06	1.17E-05	1.32E-04	1.05E-04	7.07E-04
LVAL	1.18E-07	1.84E-06	1.54E-05	4.16E-06	1.62E-06	1.43E-05	9.49E-06	3.09E-06	1.29E-04
LVALGHQQIAL	4.45E-06	2.64E-04	3.39E-04	9.73E-05	2.16E-04	4.99E-05	3.51E-04	7.92E-04	1.63E-03
LGHQQIALL	4.01E-06	1.48E-05	nan	1.30E-05	5.02E-05	3.11E-05	4.39E-05	7.31E-05	4.38E-05
IALL	6.39E-06	8.75E-06	nan	6.59E-06	1.17E-05	1.42E-05	2.24E-06	6.26E-06	1.33E-05

**LAGPLS**	5.32E-06	2.20E-04	3.37E-04	6.90E-05	3.84E-04	nan	4.80E-04	nan	nan
**LAGPLSSVSARL**	3.17E-04	4.29E-04	4.43E-04	8.11E-05	2.04E-04	4.12E-04	9.66E-04	4.86E-04	7.77E-04
**GPLSSVSAR**	4.34E-06	3.61E-04	6.58E-05	1.16E-04	nan	1.03E-03	2.65E-03	4.03E-03	3.06E-03
**LAGWHK**	8.92E-05	6.26E-05	7.76E-05	7.54E-06	5.05E-05	1.71E-05	1.57E-05	5.00E-05	6.09E-05
**YLTRNQIQP**	1.93E-06	6.68E-05	2.82E-04	6.55E-05	5.77E-04	9.71E-04	4.94E-04	1.32E-03	nan
**NQIQPIAEREGD**	3.73E-06	3.91E-04	5.22E-04	1.77E-04	7.29E-04	3.21E-04	6.84E-04	1.35E-03	5.10E-03
**IAEREGDWSAMSG**	2.85E-06	2.78E-04	1.13E-03	1.08E-05	1.36E-03	8.53E-04	1.77E-03	2.47E-03	6.54E-03
**REGDWSAMSGF**	5.96E-06	2.64E-05	2.72E-04	8.96E-05	1.12E-04	4.89E-04	3.02E-04	4.49E-04	1.10E-03
**DWSAM**	5.84E-06	2.82E-05	4.68E-04	9.85E-05	5.97E-04	2.37E-04	9.10E-04	1.41E-03	nan
**WSAM**	9.28E-05	1.04E-03	1.12E-03	1.59E-05	9.26E-04	nan	1.31E-03	nan	nan
**SAMSGFQ**	2.21E-05	1.06E-04	1.10E-03	nan	4.83E-04	4.04E-05	1.41E-03	6.97E-04	nan
**SGFQQTMQM**	4.22E-06	8.75E-06	1.02E-05	4.46E-05	1.26E-05	8.95E-06	7.64E-06	1.01E-05	8.76E-05
**QTMQML**	1.23E-05	1.20E-06	nan	1.48E-05	2.84E-06	2.71E-06	3.17E-06	1.75E-05	9.64E-05
**NEGIVPTAML**	1.15E-05	3.41E-05	3.58E-03	3.15E-04	4.98E-04	2.11E-06	2.79E-03	2.06E-03	1.13E-02
**LVANDQMALGAM**	2.32E-05	1.88E-04	nan	4.01E-04	2.93E-04	3.64E-04	2.73E-04	2.69E-04	2.79E-04
**VANDQMALGAMR**	4.09E-07	6.72E-05	2.08E-04	7.03E-06	8.02E-05	1.65E-05	2.81E-04	2.23E-04	1.42E-03
**AITESGLRVGAD**	7.54E-06	3.66E-04	3.03E-04	1.80E-04	4.68E-04	8.34E-05	3.98E-04	1.46E-03	2.23E-03
**ITESGLRVGAD**	3.83E-06	2.38E-04	2.69E-04	2.59E-04	3.34E-04	2.53E-04	3.50E-04	4.28E-04	4.41E-04
**ADISVVGY**	5.55E-06	8.57E-05	1.10E-04	1.59E-04	7.55E-05	3.96E-05	5.71E-05	1.72E-05	4.31E-04
**VVGYDD**	4.41E-06	8.46E-05	4.92E-05	6.23E-05	9.11E-05	4.41E-05	1.49E-04	1.58E-04	4.76E-04
**DDTEDSSCYIPPLTTIK**	4.55E-07	8.57E-05	1.62E-04	2.79E-05	1.87E-04	1.77E-04	2.20E-04	6.32E-04	1.17E-03
**TEDSSCYIPPLTTIK**	1.39E-06	2.08E-05	2.51E-05	1.38E-04	3.75E-05	2.82E-04	7.70E-05	2.95E-04	4.38E-04
**SSCYIPPLTTIKQD**	1.68E-06	4.75E-05	1.22E-04	3.60E-05	4.78E-05	2.26E-04	1.53E-04	1.77E-04	3.34E-04
**FRLL**	2.64E-07	1.82E-06	6.37E-05	2.22E-05	3.43E-06	7.12E-05	5.61E-05	6.13E-05	5.06E-04
**FRLLGQTSV**	2.13E-05	1.47E-05	nan	3.21E-05	2.39E-05	3.43E-05	5.08E-05	2.86E-05	1.17E-04
**LGQTSVDR**	1.32E-07	3.27E-05	2.89E-05	5.07E-05	3.72E-05	1.49E-05	4.17E-05	6.96E-05	2.73E-04
**GQTSVDRL**	1.14E-07	1.60E-05	2.30E-05	1.43E-05	3.01E-05	3.46E-05	3.31E-05	7.30E-05	1.05E-04
**LLQL**	4.02E-06	2.05E-07	7.87E-04	1.54E-04	6.61E-05	5.53E-05	2.52E-04	4.46E-04	2.71E-03

APO vs ONPFDNA

Peptide	0 s	30 s	45 s	60 s	300 s	1500 s	3600 s	7200 s	14400 s
LIGVA	4.48E-06	4.58E-05	4.82E-06	3.52E-05	4.71E-05	6.85E-06	3.77E-06	9.18E-06	6.73E-05
ALHAP	3.32E-07	2.25E-04	6.85E-05	5.34E-04	9.45E-04	2.88E-04	3.31E-04	4.60E-04	1.84E-04
ALHAPSQIVA	4.74E-06	1.32E-04	nan	1.48E-04	5.32E-05	2.29E-04	1.99E-04	1.49E-04	3.40E-04
ALHAPSQIVAA	4.93E-06	1.26E-04	nan	1.37E-04	1.21E-04	1.64E-04	2.80E-04	3.91E-04	1.47E-03
ALHAPSQIVAAIK	4.59E-06	nan	4.71E-05	1.71E-04	4.85E-05	4.35E-05	2.46E-04	8.57E-04	2.29E-04
SQIVAA	2.44E-05	1.06E-05	2.50E-06	7.42E-05	nan	4.48E-04	8.52E-06	7.66E-04	8.94E-05
SQIVAAIK	7.27E-07	9.62E-06	1.84E-05	1.79E-04	1.55E-06	4.58E-04	1.76E-04	3.72E-04	5.07E-05
IVAAIK	3.78E-06	9.32E-07	5.26E-06	1.42E-04	1.86E-06	2.19E-04	4.07E-05	1.19E-04	3.41E-04
AIKSRADQLGAS	2.22E-06	1.65E-04	1.26E-04	2.35E-05	1.48E-04	2.91E-04	2.15E-04	2.61E-04	5.64E-04
ADQLGAS	3.06E-05	4.36E-05	5.14E-04	5.73E-05	4.21E-04	6.29E-05	2.30E-04	5.05E-03	5.55E-03
VVVSM	7.45E-07	3.87E-04	4.36E-04	1.47E-04	1.22E-03	3.60E-04	3.22E-04	8.31E-04	9.58E-04
MVERSGVE	1.07E-06	1.46E-03	nan	1.21E-03	6.51E-04	6.32E-04	7.00E-04	6.01E-04	1.00E-03
AAVHNL	5.17E-05	8.95E-07	7.14E-05	2.24E-05	2.17E-04	5.20E-06	1.16E-04	nan	2.09E-04
LAQRVSGL	3.27E-07	3.49E-05	1.56E-04	3.86E-07	1.11E-04	1.35E-05	1.41E-04	3.06E-04	1.24E-04
VSGLIIN	6.77E-05	2.33E-05	6.01E-05	9.08E-05	1.31E-04	1.01E-05	1.09E-04	1.24E-04	1.15E-05
IINYPLD	3.11E-06	1.05E-06	6.12E-06	1.43E-05	4.66E-06	3.45E-05	3.49E-05	7.22E-05	1.86E-04
LDDQDAIA	6.73E-07	5.02E-04	7.38E-04	4.93E-04	nan	2.43E-04	4.69E-04	7.88E-04	6.80E-04
IAVEAA	5.32E-07	4.62E-06	6.11E-05	2.12E-04	2.12E-05	2.04E-04	1.56E-04	4.32E-04	2.20E-04
ACTNVPAL	1.29E-05	7.20E-04	8.37E-04	2.32E-04	1.52E-03	1.93E-04	1.33E-04	1.37E-03	6.77E-04
ALFLD	1.49E-06	2.47E-05	nan	6.23E-05	4.34E-04	2.23E-05	1.05E-04	1.09E-04	2.85E-04
FLDVSDQTPINS	8.64E-06	2.07E-04	nan	1.16E-04	1.22E-04	2.33E-04	3.41E-04	1.91E-04	1.44E-04
DQTPIN	7.02E-06	2.64E-05	6.36E-05	5.20E-05	7.41E-04	2.75E-05	2.15E-04	5.37E-04	3.25E-04
SIIFSH	2.74E-04	2.52E-04	8.23E-04	4.48E-04	nan	4.23E-04	1.12E-03	1.20E-03	nan
SIIFSHEDGTRL	1.86E-06	1.94E-05	nan	1.03E-05	5.02E-05	3.46E-05	6.18E-05	9.02E-05	9.48E-05
EDGTRLGVEH	1.44E-06	3.61E-05	1.03E-05	7.03E-06	3.51E-06	2.46E-05	1.69E-04	1.00E-04	5.75E-04
LVAL	1.55E-07	3.09E-07	6.23E-07	4.20E-06	5.27E-07	1.76E-05	4.55E-06	3.86E-07	4.15E-05
LVALGHQQIAL	9.32E-07	2.60E-04	2.84E-04	8.75E-05	2.27E-04	3.76E-05	2.11E-04	7.08E-04	6.74E-04
LGHQQIALL	4.79E-06	3.59E-05	nan	5.55E-05	4.62E-05	1.46E-05	3.33E-05	5.48E-05	1.55E-05
IALL	4.79E-06	7.56E-06	9.06E-06	6.93E-06	8.30E-06	6.30E-06	8.82E-07	6.59E-06	3.54E-05

**LAGPLS**	2.19E-06	2.66E-04	1.68E-04	7.39E-05	3.77E-04	1.55E-04	1.46E-04	nan	3.64E-04
**LAGPLSSVSARL**	1.54E-05	1.96E-04	2.39E-04	5.21E-05	2.99E-04	2.66E-04	8.31E-04	6.46E-04	3.25E-04
**GPLSSVSAR**	3.39E-06	4.04E-04	2.81E-04	1.13E-04	nan	1.53E-05	2.50E-03	3.50E-03	2.91E-04
**LAGWHK**	3.70E-06	4.58E-05	1.86E-06	1.18E-05	2.75E-05	5.96E-06	nan	3.35E-05	1.48E-04
**YLTRNQIQP**	1.49E-06	1.12E-04	6.34E-05	1.14E-04	5.71E-04	1.09E-03	2.35E-04	9.75E-04	nan
**NQIQPIAEREGD**	7.27E-07	3.80E-04	4.44E-04	1.69E-04	7.48E-04	4.15E-04	3.38E-04	1.11E-03	nan
**IAEREGDWSAMSG**	2.97E-06	3.28E-04	7.38E-04	1.18E-05	1.53E-03	9.23E-04	9.61E-04	1.76E-03	2.38E-03
**REGDWSAMSGF**	2.64E-05	9.14E-05	3.94E-04	1.26E-04	9.83E-05	1.07E-04	2.10E-04	1.69E-04	3.03E-04
**DWSAM**	1.35E-06	2.16E-05	1.30E-04	5.30E-05	6.79E-04	4.25E-04	6.03E-04	1.04E-03	1.07E-03
**WSAM**	2.66E-05	1.16E-04	4.32E-04	9.45E-05	8.51E-04	3.87E-04	4.70E-04	nan	nan
**SAMSGFQ**	7.55E-05	7.57E-05	2.20E-04	3.22E-04	7.51E-04	6.31E-05	1.22E-03	6.16E-04	2.66E-03
**SGFQQTMQM**	6.77E-06	1.48E-06	2.53E-05	nan	2.97E-06	1.14E-05	1.68E-06	1.31E-05	3.90E-05
**QTMQML**	8.53E-06	1.11E-06	3.65E-06	1.29E-05	2.99E-06	1.14E-05	3.06E-06	1.78E-05	1.07E-04
**NEGIVPTAML**	3.46E-05	1.37E-04	8.22E-04	3.10E-04	4.39E-04	2.28E-04	1.07E-03	1.21E-03	5.80E-03
**LVANDQMALGAM**	1.42E-04	2.00E-03	nan	3.99E-03	3.09E-03	4.85E-03	4.05E-03	2.23E-03	5.18E-03
**VANDQMALGAMR**	4.82E-07	5.30E-05	5.01E-05	1.44E-05	4.32E-05	1.68E-06	1.88E-04	2.43E-04	8.48E-04
**AITESGLRVGAD**	1.33E-06	3.43E-04	7.56E-05	1.40E-04	4.75E-04	5.50E-05	1.41E-04	1.01E-03	8.72E-04
**ITESGLRVGAD**	7.39E-06	2.87E-04	1.95E-04	2.07E-04	1.91E-04	9.31E-05	1.99E-04	2.39E-04	1.13E-04
**ADISVVGY**	9.45E-07	4.21E-05	6.75E-05	4.70E-06	1.33E-05	2.12E-05	2.78E-05	3.86E-05	3.90E-04
**VVGYDD**	2.63E-06	5.18E-05	6.68E-05	2.16E-05	2.75E-05	2.14E-05	6.73E-05	1.66E-04	2.78E-04
**DDTEDSSCYIPPLTTIK**	5.14E-07	7.83E-05	2.43E-04	3.77E-05	2.44E-04	1.75E-04	1.92E-04	5.21E-04	5.94E-04
**TEDSSCYIPPLTTIK**	1.39E-06	1.10E-05	1.47E-05	1.37E-04	6.50E-05	2.83E-04	7.19E-05	2.56E-04	1.73E-04
**SSCYIPPLTTIKQD**	2.64E-07	6.50E-05	8.49E-05	3.76E-05	3.83E-05	2.29E-04	1.35E-05	1.39E-04	1.83E-04
**FRLL**	6.77E-07	1.03E-06	7.27E-06	2.05E-05	1.32E-07	8.36E-05	3.69E-05	4.74E-05	1.43E-04
**FRLLGQTSV**	7.57E-05	2.22E-05	1.50E-05	3.22E-05	1.59E-05	7.79E-05	1.46E-05	2.21E-05	4.60E-05
**LGQTSVDR**	1.82E-08	2.10E-05	2.50E-05	2.36E-05	2.12E-05	5.30E-06	2.03E-05	7.22E-05	1.97E-04
**GQTSVDRL**	2.77E-07	1.43E-05	1.46E-05	1.22E-05	1.26E-05	2.91E-05	3.85E-06	6.65E-05	6.68E-05
**LLQL**	1.93E-06	4.70E-06	1.42E-04	1.58E-04	1.12E-04	5.58E-05	3.10E-04	4.23E-04	7.08E-04

APO vs TMG

Peptide	0 s	30 s	45 s	60 s	300 s	1500 s	3600 s	7200 s	14400 s
LIGVA	3.77E-06	2.31E-05	5.27E-06	2.70E-05	3.35E-05	2.22E-06	1.71E-05	9.33E-06	4.63E-05
ALHAP	4.09E-07	2.56E-04	7.29E-05	7.12E-04	8.83E-04	4.26E-04	5.55E-04	6.77E-04	3.76E-04
ALHAPSQIVA	2.99E-06	4.66E-05	nan	7.08E-04	1.35E-05	2.26E-04	1.07E-04	4.27E-04	1.76E-04
ALHAPSQIVAA	5.76E-06	6.69E-05	6.97E-05	6.62E-04	8.38E-05	2.00E-04	9.69E-05	6.17E-04	1.39E-03
ALHAPSQIVAAIK	7.93E-06	nan	2.10E-04	nan	1.57E-04	3.75E-05	1.19E-03	6.63E-04	2.04E-04
SQIVAA	1.35E-05	1.21E-05	3.31E-05	1.50E-04	nan	2.80E-04	9.05E-05	7.82E-04	8.57E-05
SQIVAAIK	1.82E-06	9.94E-06	1.68E-05	2.65E-04	1.18E-06	4.82E-04	2.16E-05	2.52E-04	5.76E-05
IVAAIK	4.44E-06	1.64E-06	4.37E-06	1.84E-04	2.53E-06	2.33E-04	3.49E-05	8.84E-05	3.41E-04
AIKSRADQLGAS	2.92E-06	2.92E-04	1.11E-04	3.13E-05	1.28E-04	2.32E-04	4.03E-04	1.15E-03	5.73E-04
ADQLGAS	1.38E-05	1.97E-04	5.40E-04	3.00E-04	2.34E-05	6.66E-04	1.52E-03	2.67E-03	4.10E-03
VVVSM	6.15E-05	5.87E-04	2.72E-04	6.92E-05	1.02E-03	2.50E-04	2.96E-04	1.12E-03	6.89E-04
MVERSGVE	1.11E-05	2.99E-03	nan	1.05E-03	6.57E-04	1.23E-03	1.32E-03	1.35E-03	1.19E-03
AAVHNL	2.40E-05	1.18E-06	3.76E-04	3.54E-05	8.28E-05	4.46E-05	2.20E-04	2.10E-03	2.85E-04
LAQRVSGL	3.86E-07	1.98E-04	2.25E-04	4.39E-05	1.31E-04	5.18E-05	1.76E-04	6.32E-04	2.81E-04
VSGLIIN	5.11E-05	1.63E-05	8.09E-05	9.32E-05	2.70E-05	2.16E-05	1.11E-04	1.00E-04	1.60E-05
IINYPLD	1.87E-05	1.18E-05	2.02E-06	nan	1.82E-06	3.53E-05	2.36E-05	7.94E-05	1.76E-04
LDDQDAIA	6.24E-06	1.18E-03	6.42E-04	4.88E-04	nan	3.43E-04	4.67E-04	1.01E-03	5.12E-04
IAVEAA	3.73E-07	9.89E-06	1.09E-04	2.00E-04	2.48E-05	4.48E-05	1.45E-04	5.35E-04	2.06E-04
ACTNVPAL	3.65E-05	2.28E-03	8.38E-04	3.18E-04	1.66E-03	8.93E-04	5.11E-04	3.07E-03	1.22E-03
ALFLD	1.01E-05	3.53E-06	1.18E-04	1.81E-05	9.32E-06	7.27E-07	2.29E-05	6.85E-05	1.95E-04
FLDVSDQTPINS	1.35E-05	2.74E-04	nan	7.54E-04	3.57E-05	5.43E-04	3.36E-04	6.38E-04	1.01E-04
DQTPIN	2.65E-06	4.77E-05	1.75E-04	4.63E-04	6.15E-04	4.51E-04	3.94E-04	1.13E-03	3.95E-04
SIIFSH	3.42E-04	4.16E-05	9.17E-05	6.83E-04	nan	1.18E-04	1.17E-04	3.67E-04	2.45E-04
SIIFSHEDGTRL	2.01E-06	2.03E-04	5.52E-06	2.45E-04	5.34E-05	6.71E-05	2.90E-05	8.01E-05	1.11E-04
EDGTRLGVEH	1.75E-05	8.18E-05	3.22E-04	nan	1.02E-04	4.70E-04	1.87E-05	1.28E-04	9.96E-04
LVAL	1.86E-07	1.32E-07	6.64E-06	1.48E-04	4.59E-07	3.17E-05	6.42E-06	2.41E-07	3.79E-05
LVALGHQQIAL	4.98E-06	4.98E-04	2.35E-04	4.14E-04	1.84E-04	2.52E-04	1.56E-04	5.71E-04	7.47E-04
LGHQQIALL	1.08E-05	3.33E-05	nan	1.03E-05	2.68E-05	6.07E-05	3.46E-05	6.37E-05	2.82E-05
IALL	4.95E-06	1.35E-05	9.43E-06	6.80E-05	7.86E-06	2.20E-05	1.54E-06	9.54E-06	1.25E-05

**LAGPLS**	2.26E-05	6.59E-04	1.72E-04	4.88E-04	3.96E-04	3.13E-04	1.41E-04	nan	4.59E-04
**LAGPLSSVSARL**	3.93E-05	1.56E-04	9.43E-05	2.71E-04	1.32E-04	6.82E-04	7.83E-04	nan	nan
**GPLSSVSAR**	6.88E-06	7.47E-04	1.42E-04	nan	nan	4.13E-04	2.79E-03	3.40E-03	nan
**LAGWHK**	1.70E-05	1.87E-04	2.43E-04	nan	1.30E-07	5.08E-04	1.61E-05	3.01E-04	4.25E-04
**YLTRNQIQP**	2.79E-06	4.81E-04	nan	1.57E-04	5.26E-04	nan	4.85E-04	nan	1.87E-03
**NQIQPIAEREGD**	1.20E-06	1.28E-03	4.43E-04	5.23E-04	nan	7.84E-04	nan	1.29E-03	1.40E-03
**IAEREGDWSAMSG**	2.97E-06	8.18E-04	5.79E-04	7.04E-04	1.43E-03	1.78E-03	1.34E-03	2.70E-03	2.87E-03
**REGDWSAMSGF**	6.29E-06	3.79E-04	2.98E-04	3.11E-04	nan	2.75E-04	nan	8.80E-04	4.85E-04
**DWSAM**	1.41E-05	1.60E-04	2.05E-04	7.44E-04	6.03E-04	6.33E-04	8.29E-04	1.82E-03	1.28E-03
**WSAM**	1.48E-05	7.12E-05	4.77E-04	2.59E-04	6.81E-04	5.27E-04	7.05E-04	nan	1.26E-03
**SAMSGFQ**	2.91E-05	4.48E-04	8.73E-04	5.86E-04	4.99E-04	1.74E-04	1.29E-03	nan	2.68E-03
**SGFQQTMQM**	6.47E-06	2.64E-06	3.04E-05	nan	3.84E-06	1.36E-05	3.21E-06	3.51E-06	3.06E-05
**QTMQML**	2.71E-06	1.20E-05	1.59E-05	nan	3.07E-06	7.39E-06	4.22E-06	2.30E-05	8.29E-05
**NEGIVPTAML**	2.20E-05	2.78E-04	1.50E-03	3.18E-04	2.45E-04	2.36E-04	6.30E-04	2.22E-03	5.37E-03
**LVANDQMALGAM**	2.29E-05	2.45E-04	6.22E-06	nan	2.68E-04	4.68E-04	2.51E-04	2.16E-04	1.99E-04
**VANDQMALGAMR**	1.14E-06	1.20E-04	8.91E-07	nan	3.38E-05	1.97E-04	1.54E-05	1.22E-04	8.60E-04
**AITESGLRVGAD**	9.72E-06	1.09E-03	4.65E-06	6.40E-04	6.34E-04	7.19E-04	3.08E-04	1.49E-03	1.20E-03
**ITESGLRVGAD**	9.36E-06	1.17E-03	1.93E-04	4.12E-04	3.40E-04	6.96E-04	3.76E-04	9.44E-04	3.68E-04
**ADISVVGY**	1.31E-06	2.39E-05	6.10E-06	nan	1.31E-06	1.77E-04	5.06E-06	6.59E-06	4.24E-04
**VVGYDD**	2.77E-06	2.86E-05	2.53E-05	nan	2.95E-05	9.84E-05	3.62E-05	9.07E-05	3.25E-04
**DDTEDSSCYIPPLTTIK**	4.55E-07	2.17E-04	1.75E-04	nan	1.74E-04	4.35E-04	1.74E-04	1.13E-03	1.34E-03
**TEDSSCYIPPLTTIK**	3.78E-06	5.63E-05	1.25E-04	nan	7.80E-06	3.76E-04	2.53E-05	5.09E-04	4.61E-04
**SSCYIPPLTTIKQD**	1.55E-07	9.28E-05	1.96E-04	3.33E-03	3.28E-05	4.58E-04	2.02E-05	1.82E-04	3.73E-04
**FRLL**	4.45E-07	2.01E-06	5.38E-05	1.78E-04	3.19E-06	8.22E-05	1.33E-05	1.41E-04	1.87E-04
**FRLLGQTSV**	2.54E-05	6.55E-05	9.84E-05	1.32E-04	4.28E-05	8.54E-05	nan	3.90E-05	3.39E-05
**LGQTSVDR**	2.41E-07	2.48E-05	2.75E-05	1.71E-04	2.22E-05	5.73E-05	1.76E-05	5.46E-05	2.17E-04
**GQTSVDRL**	2.77E-07	nan	nan	8.64E-05	nan	8.47E-05	1.45E-06	6.06E-05	nan
**LLQL**	2.46E-06	5.60E-05	3.05E-04	2.13E-04	6.81E-05	1.79E-04	1.76E-04	1.17E-03	4.32E-04

DNA vs ONPFDNA

Peptide	0 s	30 s	45 s	60 s	300 s	1500 s	3600 s	7200 s	14400 s
LIGVA	1.17E-06	3.62E-05	1.79E-05	2.19E-05	3.91E-05	5.68E-06	6.18E-07	2.05E-06	3.47E-05
ALHAP	1.76E-06	2.57E-05	2.41E-04	9.10E-05	1.22E-04	1.09E-05	1.72E-04	5.24E-04	nan
ALHAPSQIVA	2.57E-06	1.18E-04	nan	1.93E-04	7.47E-05	2.25E-04	2.19E-04	1.02E-04	3.76E-04
ALHAPSQIVAA	2.82E-06	1.36E-04	nan	2.05E-04	1.15E-04	1.78E-04	3.42E-04	2.86E-04	6.67E-04
ALHAPSQIVAAIK	2.50E-06	4.84E-05	2.24E-04	3.09E-05	7.60E-05	7.59E-05	7.43E-04	5.70E-04	2.06E-03
SQIVAA	2.64E-05	2.15E-05	1.42E-05	2.76E-05	nan	2.73E-04	9.43E-06	6.34E-05	4.55E-04
SQIVAAIK	3.64E-06	2.00E-05	nan	4.20E-06	2.29E-05	4.20E-06	2.70E-04	1.79E-04	5.45E-04
IVAAIK	4.48E-06	4.86E-06	5.54E-05	1.11E-06	4.31E-06	1.31E-06	4.14E-05	4.71E-05	1.83E-04
AIKSRADQLGAS	2.16E-07	1.45E-04	2.46E-04	1.68E-04	1.95E-04	2.84E-04	2.33E-04	1.70E-04	1.31E-03
ADQLGAS	4.33E-05	6.42E-05	6.58E-04	3.48E-04	4.24E-04	1.41E-04	1.40E-04	nan	5.90E-03
VVVSM	1.00E-06	3.10E-05	8.68E-04	2.69E-04	3.46E-04	1.81E-04	2.18E-04	7.27E-04	1.02E-03
MVERSGVE	4.34E-07	1.00E-03	nan	5.43E-04	9.65E-04	6.13E-04	8.08E-04	7.90E-04	6.80E-04
AAVHNL	3.63E-05	1.86E-05	1.49E-04	2.27E-05	3.70E-04	1.68E-06	5.00E-04	nan	6.07E-04
LAQRVSGL	1.82E-07	1.91E-05	1.63E-04	2.17E-06	1.31E-05	1.31E-05	1.40E-04	6.36E-05	6.43E-04
VSGLIIN	3.22E-05	1.06E-05	2.91E-05	7.73E-06	1.06E-04	1.86E-06	2.86E-05	9.49E-05	7.44E-06
IINYPLD	8.33E-06	5.73E-06	1.74E-05	1.67E-05	2.20E-05	4.92E-05	2.83E-05	4.85E-05	5.79E-04
LDDQDAIA	6.95E-07	7.34E-05	5.52E-04	4.82E-04	5.19E-04	3.44E-04	7.69E-04	8.95E-04	2.94E-03
IAVEAA	3.86E-07	nan	9.49E-04	1.27E-05	3.56E-05	2.69E-04	4.31E-05	7.16E-05	1.38E-04
ACTNVPAL	1.81E-05	4.64E-05	3.34E-04	1.63E-05	2.69E-05	2.05E-05	4.91E-04	1.12E-03	3.53E-03
ALFLD	1.49E-06	5.60E-05	nan	7.70E-05	4.79E-04	5.36E-05	1.64E-04	9.21E-05	1.93E-04
FLDVSDQTPINS	3.82E-07	1.61E-04	nan	1.78E-04	1.92E-04	2.71E-04	4.90E-04	4.20E-04	3.35E-04
DQTPIN	1.15E-05	2.56E-05	5.11E-04	8.97E-06	1.57E-04	3.91E-05	2.83E-04	3.73E-04	2.03E-03
SIIFSH	2.57E-04	3.39E-04	9.67E-04	5.39E-04	4.40E-04	6.43E-04	1.26E-03	8.52E-04	nan
SIIFSHEDGTRL	2.44E-07	1.97E-05	nan	2.81E-05	4.05E-05	3.24E-05	6.18E-05	9.11E-05	9.72E-05
EDGTRLGVEH	5.84E-06	2.87E-05	8.33E-06	4.29E-05	5.14E-06	2.10E-05	2.67E-04	4.15E-05	1.36E-04
LVAL	4.55E-08	2.11E-06	1.58E-05	1.86E-07	1.24E-06	3.32E-06	1.11E-05	3.44E-06	9.55E-05
LVALGHQQIAL	3.60E-06	5.97E-05	1.63E-04	3.35E-05	8.68E-05	1.23E-05	2.96E-04	6.65E-04	9.54E-04
LGHQQIALL	1.05E-06	3.95E-05	1.01E-04	6.80E-05	5.15E-05	2.27E-05	3.34E-05	5.71E-05	3.04E-05
IALL	4.55E-06	1.76E-06	nan	1.28E-05	4.75E-06	8.09E-06	1.59E-06	4.09E-07	2.50E-05

**LAGPLS**	6.77E-06	8.08E-05	1.73E-04	1.20E-05	7.14E-06	nan	3.54E-04	nan	nan
**LAGPLSSVSARL**	3.04E-04	4.64E-04	4.95E-04	5.93E-05	2.39E-04	4.01E-04	4.26E-04	7.80E-04	1.04E-03
**GPLSSVSAR**	1.10E-06	5.70E-05	2.33E-04	2.20E-05	1.84E-06	1.04E-03	1.58E-04	2.12E-03	2.77E-03
**LAGWHK**	9.27E-05	6.98E-05	7.81E-05	7.10E-06	7.80E-05	2.17E-05	nan	2.20E-05	8.73E-05
**YLTRNQIQP**	4.73E-07	4.57E-05	2.84E-04	4.83E-05	1.82E-04	1.23E-04	2.61E-04	3.51E-04	nan
**NQIQPIAEREGD**	3.15E-06	1.64E-05	8.12E-05	7.68E-06	8.74E-05	1.21E-04	4.68E-04	8.87E-04	nan
**IAEREGDWSAMSG**	1.42E-06	1.62E-04	9.05E-04	1.15E-05	3.72E-04	7.13E-05	9.97E-04	7.06E-04	4.23E-03
**REGDWSAMSGF**	2.74E-05	7.26E-05	6.20E-04	1.75E-04	1.01E-04	4.83E-04	2.85E-04	3.13E-04	1.04E-03
**DWSAM**	5.40E-06	1.71E-05	4.27E-04	4.55E-05	1.81E-04	1.98E-04	4.37E-04	3.67E-04	nan
**WSAM**	8.98E-05	1.02E-03	9.32E-04	1.02E-04	4.57E-04	nan	8.60E-04	7.71E-04	nan
**SAMSGFQ**	7.10E-05	4.46E-05	1.09E-03	nan	2.69E-04	2.98E-05	2.07E-04	8.18E-05	nan
**SGFQQTMQM**	7.35E-06	7.28E-06	1.54E-05	nan	1.40E-05	1.77E-05	8.59E-06	2.06E-05	6.54E-05
**QTMQML**	1.90E-05	1.00E-06	nan	6.77E-06	5.91E-07	1.05E-05	1.42E-06	3.86E-07	3.97E-05
**NEGIVPTAML**	3.36E-05	1.27E-04	4.08E-03	5.55E-06	4.48E-04	2.26E-04	2.76E-03	8.80E-04	7.22E-03
**LVANDQMALGAM**	1.19E-04	1.94E-03	nan	3.96E-03	3.00E-03	4.74E-03	3.93E-03	2.13E-03	5.07E-03
**VANDQMALGAMR**	1.55E-07	5.15E-05	2.58E-04	2.12E-05	8.95E-05	1.68E-05	4.39E-04	2.65E-04	6.58E-04
**AITESGLRVGAD**	6.24E-06	3.67E-05	3.78E-04	4.15E-05	9.11E-05	6.80E-05	2.97E-04	9.40E-04	1.36E-03
**ITESGLRVGAD**	4.94E-06	2.55E-04	1.54E-04	2.24E-04	2.45E-04	1.66E-04	2.94E-04	2.92E-04	4.04E-04
**ADISVVGY**	5.91E-06	1.17E-04	1.73E-04	1.64E-04	8.68E-05	5.68E-05	8.47E-05	4.59E-05	4.59E-05
**VVGYDD**	2.23E-06	1.05E-04	7.15E-05	7.34E-05	9.01E-05	2.71E-05	1.45E-04	1.50E-04	1.99E-04
**DDTEDSSCYIPPLTTIK**	3.86E-07	2.42E-05	1.70E-04	1.97E-05	1.42E-04	6.57E-06	1.21E-04	1.87E-04	7.53E-04
**TEDSSCYIPPLTTIK**	4.45E-07	2.51E-05	3.41E-05	8.75E-06	8.86E-05	1.06E-06	1.15E-04	5.74E-05	3.13E-04
**SSCYIPPLTTIKQD**	1.86E-06	5.13E-05	1.12E-04	1.68E-05	3.90E-05	7.35E-06	1.42E-04	9.49E-05	1.54E-04
**FRLL**	4.95E-07	2.84E-06	6.17E-05	1.89E-06	3.33E-06	1.51E-05	8.49E-05	4.14E-05	3.64E-04
**FRLLGQTSV**	5.55E-05	1.19E-05	nan	5.75E-06	9.58E-06	4.35E-05	4.25E-05	6.59E-06	1.28E-04
**LGQTSVDR**	1.14E-07	2.10E-05	1.06E-05	3.70E-05	2.12E-05	9.66E-06	2.71E-05	4.72E-05	7.69E-05
**GQTSVDRL**	1.64E-07	9.39E-06	1.01E-05	9.71E-06	2.08E-05	8.39E-06	3.64E-05	3.93E-05	4.06E-05
**LLQL**	5.04E-06	4.82E-06	8.83E-04	3.40E-05	6.20E-05	1.06E-06	4.52E-04	2.07E-04	2.99E-03

DNA vs TMG

Peptide	0 s	30 s	45 s	60 s	300 s	1500 s	3600 s	7200 s	14400 s
LIGVA	4.59E-07	1.34E-05	1.84E-05	1.38E-05	2.55E-05	1.04E-06	1.39E-05	2.20E-06	1.37E-05
ALHAP	1.84E-06	5.70E-05	2.46E-04	2.69E-04	5.99E-05	1.49E-04	3.95E-04	7.41E-04	nan
ALHAPSQIVA	8.15E-07	3.23E-05	nan	7.53E-04	3.50E-05	2.22E-04	1.27E-04	3.81E-04	2.12E-04
ALHAPSQIVAA	3.65E-06	7.69E-05	1.80E-04	7.30E-04	7.78E-05	2.14E-04	1.58E-04	5.13E-04	5.89E-04
ALHAPSQIVAAIK	5.84E-06	1.43E-04	3.88E-04	nan	1.84E-04	6.99E-05	1.68E-03	3.76E-04	2.04E-03
SQIVAA	1.55E-05	2.31E-05	4.48E-05	1.04E-04	nan	1.06E-04	9.14E-05	7.92E-05	4.51E-04
SQIVAAIK	4.73E-06	2.03E-05	nan	9.11E-05	2.26E-05	2.78E-05	1.15E-04	5.92E-05	5.52E-04
IVAAIK	5.13E-06	5.56E-06	5.45E-05	4.30E-05	4.98E-06	1.51E-05	3.56E-05	1.61E-05	1.83E-04
AIKSRADQLGAS	9.16E-07	2.72E-04	2.31E-04	1.76E-04	1.75E-04	2.26E-04	4.22E-04	1.06E-03	1.32E-03
ADQLGAS	2.65E-05	2.17E-04	6.83E-04	5.91E-04	2.56E-05	7.44E-04	1.43E-03	nan	4.46E-03
VVVSM	6.17E-05	2.31E-04	7.04E-04	1.91E-04	1.44E-04	7.08E-05	1.91E-04	1.02E-03	7.49E-04
MVERSGVE	1.05E-05	2.53E-03	nan	3.82E-04	9.70E-04	1.21E-03	1.43E-03	1.54E-03	8.67E-04
AAVHNL	8.51E-06	1.89E-05	4.54E-04	3.57E-05	2.36E-04	4.11E-05	6.05E-04	3.30E-04	6.82E-04
LAQRVSGL	2.41E-07	1.82E-04	2.31E-04	4.57E-05	3.26E-05	5.14E-05	1.74E-04	3.89E-04	8.00E-04
VSGLIIN	1.56E-05	3.64E-06	5.00E-05	1.01E-05	2.02E-06	1.33E-05	3.02E-05	7.16E-05	1.20E-05
IINYPLD	2.39E-05	1.65E-05	1.33E-05	nan	1.92E-05	5.00E-05	1.70E-05	5.57E-05	5.70E-04
LDDQDAIA	6.26E-06	7.48E-04	4.55E-04	4.77E-04	3.78E-04	4.44E-04	7.68E-04	1.12E-03	2.77E-03
IAVEAA	2.27E-07	nan	9.97E-04	9.32E-07	3.92E-05	1.10E-04	3.29E-05	1.75E-04	1.23E-04
ACTNVPAL	4.17E-05	1.60E-03	3.35E-04	1.03E-04	1.65E-04	7.20E-04	8.68E-04	2.83E-03	4.07E-03
ALFLD	1.01E-05	3.49E-05	2.68E-04	3.27E-05	5.47E-05	3.20E-05	8.20E-05	5.15E-05	1.04E-04
FLDVSDQTPINS	5.27E-06	2.28E-04	nan	8.16E-04	1.06E-04	5.82E-04	4.86E-04	8.67E-04	2.92E-04
DQTPIN	7.15E-06	4.70E-05	6.23E-04	4.20E-04	3.10E-05	4.62E-04	4.62E-04	9.69E-04	2.10E-03
SIIFSH	3.24E-04	1.29E-04	2.36E-04	7.73E-04	4.55E-05	3.38E-04	2.63E-04	1.81E-05	8.02E-04
SIIFSHEDGTRL	3.92E-07	2.03E-04	5.83E-06	2.63E-04	4.37E-05	6.49E-05	2.89E-05	8.10E-05	1.13E-04
EDGTRLGVEH	2.19E-05	7.44E-05	3.20E-04	nan	1.03E-04	4.67E-04	1.17E-04	6.93E-05	5.57E-04
LVAL	7.73E-08	1.93E-06	2.19E-05	1.44E-04	1.17E-06	1.75E-05	1.30E-05	3.30E-06	9.19E-05
LVALGHQQIAL	7.65E-06	2.98E-04	1.14E-04	3.60E-04	4.39E-05	2.26E-04	2.42E-04	5.28E-04	1.03E-03
LGHQQIALL	7.05E-06	3.68E-05	2.51E-05	2.28E-05	3.22E-05	6.87E-05	3.48E-05	6.61E-05	4.31E-05
IALL	4.71E-06	7.70E-06	nan	7.39E-05	4.31E-06	2.38E-05	2.24E-06	3.35E-06	2.13E-06

**LAGPLS**	2.72E-05	4.74E-04	1.77E-04	4.26E-04	2.61E-05	nan	3.49E-04	9.37E-04	nan
**LAGPLSSVSARL**	3.28E-04	4.24E-04	3.50E-04	2.78E-04	7.20E-05	8.17E-04	3.78E-04	nan	nan
**GPLSSVSAR**	4.59E-06	4.00E-04	9.41E-05	nan	1.06E-05	1.44E-03	4.49E-04	2.03E-03	nan
**LAGWHK**	1.06E-04	2.11E-04	3.19E-04	nan	5.06E-05	5.23E-04	2.81E-05	2.90E-04	3.64E-04
**YLTRNQIQP**	1.77E-06	4.15E-04	nan	9.17E-05	1.37E-04	nan	5.11E-04	nan	nan
**NQIQPIAEREGD**	3.62E-06	9.14E-04	7.94E-05	3.61E-04	nan	4.90E-04	nan	1.07E-03	4.05E-03
**IAEREGDWSAMSG**	1.42E-06	6.52E-04	7.45E-04	7.04E-04	2.73E-04	9.29E-04	1.38E-03	1.64E-03	4.72E-03
**REGDWSAMSGF**	7.32E-06	3.60E-04	5.24E-04	3.60E-04	nan	6.50E-04	nan	1.02E-03	1.22E-03
**DWSAM**	1.82E-05	1.56E-04	5.02E-04	7.37E-04	1.06E-04	4.06E-04	6.63E-04	1.15E-03	nan
**WSAM**	7.80E-05	9.70E-04	9.77E-04	2.67E-04	2.87E-04	nan	1.09E-03	nan	nan
**SAMSGFQ**	2.46E-05	4.17E-04	1.74E-03	nan	1.65E-05	1.41E-04	2.78E-04	nan	nan
**SGFQQTMQM**	7.06E-06	8.44E-06	2.05E-05	nan	1.49E-05	1.99E-05	1.01E-05	1.10E-05	5.70E-05
**QTMQML**	1.32E-05	1.19E-05	nan	nan	6.77E-07	6.46E-06	2.59E-06	5.59E-06	1.61E-05
**NEGIVPTAML**	2.11E-05	2.67E-04	4.76E-03	1.33E-05	2.53E-04	2.35E-04	2.32E-03	1.89E-03	6.78E-03
**LVANDQMALGAM**	3.41E-07	1.81E-04	nan	nan	1.72E-04	3.52E-04	1.32E-04	1.14E-04	8.72E-05
**VANDQMALGAMR**	8.09E-07	1.18E-04	2.09E-04	nan	8.02E-05	2.12E-04	2.67E-04	1.43E-04	6.70E-04
**AITESGLRVGAD**	1.46E-05	7.86E-04	3.07E-04	5.42E-04	2.50E-04	7.33E-04	4.65E-04	1.42E-03	1.68E-03
**ITESGLRVGAD**	6.92E-06	1.14E-03	1.52E-04	4.29E-04	3.95E-04	7.68E-04	4.71E-04	9.98E-04	6.59E-04
**ADISVVGY**	6.28E-06	9.90E-05	1.12E-04	nan	7.48E-05	2.12E-04	6.20E-05	1.39E-05	8.05E-05
**VVGYDD**	2.37E-06	8.15E-05	3.00E-05	nan	9.21E-05	1.04E-04	1.14E-04	7.51E-05	2.46E-04
**DDTEDSSCYIPPLTTIK**	3.27E-07	1.63E-04	1.02E-04	nan	7.26E-05	2.66E-04	1.03E-04	7.92E-04	1.50E-03
**TEDSSCYIPPLTTIK**	2.84E-06	7.05E-05	1.45E-04	nan	3.15E-05	9.43E-05	6.85E-05	3.10E-04	6.01E-04
**SSCYIPPLTTIKQD**	1.75E-06	7.91E-05	2.23E-04	3.31E-03	3.34E-05	2.37E-04	1.49E-04	1.38E-04	3.43E-04
**FRLL**	2.64E-07	3.82E-06	1.08E-04	1.59E-04	6.39E-06	1.36E-05	6.12E-05	1.35E-04	4.09E-04
**FRLLGQTSV**	5.20E-06	5.52E-05	nan	1.05E-04	3.65E-05	5.11E-05	nan	2.35E-05	1.16E-04
**LGQTSVDR**	3.36E-07	2.48E-05	1.32E-05	1.84E-04	2.22E-05	6.17E-05	2.44E-05	2.95E-05	9.72E-05
**GQTSVDRL**	1.64E-07	nan	nan	8.39E-05	nan	6.39E-05	3.40E-05	3.34E-05	nan
**LLQL**	5.57E-06	5.62E-05	1.05E-03	8.81E-05	1.81E-05	1.24E-04	3.17E-04	9.51E-04	2.71E-03

IPTG vs APO

Peptide	0 s	30 s	45 s	60 s	300 s	1500 s	3600 s	7200 s	14400 s
LIGVA	8.88E-06	2.55E-05	6.88E-05	5.18E-05	3.29E-05	5.51E-06	3.77E-06	1.04E-05	5.68E-05
ALHAP	2.64E-07	2.36E-04	7.46E-05	5.21E-04	9.31E-04	5.64E-04	4.26E-04	6.68E-04	4.05E-04
ALHAPSQIVA	3.18E-06	2.41E-05	nan	2.94E-05	2.42E-05	2.99E-05	8.52E-05	8.56E-05	2.23E-05
ALHAPSQIVAA	5.12E-06	3.60E-05	nan	7.00E-05	9.12E-05	4.32E-05	1.02E-04	2.89E-04	1.24E-03
ALHAPSQIVAAIK	1.05E-05	nan	5.28E-05	1.97E-04	3.29E-05	2.86E-05	7.15E-05	5.01E-04	2.06E-04
SQIVAA	3.79E-06	1.13E-05	1.79E-06	8.51E-05	1.12E-04	2.52E-04	4.77E-06	7.64E-04	9.16E-05
SQIVAAIK	2.85E-06	1.76E-05	1.54E-05	1.83E-04	5.20E-05	4.55E-04	1.99E-05	2.51E-04	4.08E-05
IVAAIK	2.62E-06	1.06E-05	6.59E-07	1.46E-04	7.53E-06	2.18E-04	2.62E-05	7.97E-05	3.39E-04
AIKSRADQLGAS	2.41E-06	1.06E-04	1.50E-04	9.79E-05	1.36E-04	1.51E-04	2.83E-04	4.79E-04	5.11E-04
ADQLGAS	1.43E-05	nan	5.74E-04	nan	1.36E-04	nan	2.12E-03	2.30E-03	4.99E-03
VVVSM	3.64E-07	5.95E-04	1.88E-04	5.33E-05	1.09E-03	5.58E-04	2.98E-04	9.34E-04	9.58E-04
MVERSGVE	8.78E-07	1.30E-03	nan	1.42E-03	7.71E-04	8.01E-04	8.76E-04	1.01E-03	8.92E-04
AAVHNL	5.89E-05	2.65E-05	7.28E-05	2.57E-05	2.55E-04	1.29E-03	1.44E-03	2.96E-03	1.85E-03
LAQRVSGL	1.98E-06	2.57E-04	2.05E-04	2.04E-06	2.41E-04	3.21E-04	2.28E-04	6.19E-04	6.01E-04
VSGLIIN	5.11E-05	1.61E-05	5.25E-05	9.84E-05	4.98E-05	9.35E-06	1.33E-04	9.76E-05	1.28E-05
IINYPLD	3.35E-06	8.91E-06	5.50E-06	1.18E-05	3.24E-05	6.34E-06	7.56E-05	1.02E-04	2.32E-04
LDDQDAIA	2.23E-05	1.14E-03	8.53E-04	7.05E-04	nan	7.06E-04	6.07E-04	1.35E-03	1.30E-03
IAVEAA	9.18E-07	1.84E-05	1.14E-05	2.04E-04	2.51E-05	9.92E-05	1.97E-04	7.35E-04	1.03E-03
ACTNVPAL	1.96E-05	1.59E-03	8.39E-04	2.16E-04	1.82E-03	7.56E-04	2.02E-04	2.06E-03	1.32E-03
ALFLD	4.67E-06	1.31E-05	3.11E-05	2.95E-05	2.64E-05	1.10E-05	8.04E-05	6.48E-05	2.06E-04
FLDVSDQTPINS	8.83E-06	1.55E-04	nan	1.82E-04	1.04E-04	3.57E-04	3.08E-04	4.42E-04	2.14E-04
DQTPIN	7.73E-06	2.49E-04	1.08E-04	1.10E-04	6.77E-04	1.08E-03	5.05E-04	8.56E-04	6.81E-04
SIIFSH	2.77E-05	1.93E-04	1.73E-04	2.36E-04	nan	6.04E-05	1.56E-04	4.85E-04	3.48E-04
SIIFSHEDGTRL	5.70E-06	1.72E-05	7.16E-06	4.84E-06	4.44E-05	3.55E-05	4.31E-05	5.83E-05	1.05E-04
EDGTRLGVEH	2.19E-06	2.08E-05	2.31E-05	3.07E-05	1.32E-04	1.29E-05	5.21E-05	9.12E-05	6.11E-04
LVAL	1.14E-07	1.18E-06	2.27E-06	8.75E-06	9.66E-06	1.50E-05	4.79E-06	1.66E-06	6.39E-05
LVALGHQQIAL	1.35E-06	6.22E-04	3.66E-04	2.38E-04	4.60E-04	4.15E-04	3.61E-04	7.66E-04	1.12E-03
LGHQQIALL	4.20E-06	3.72E-05	nan	2.64E-05	6.38E-05	3.19E-05	3.92E-05	6.97E-05	3.56E-05
IALL	3.77E-06	7.50E-06	8.45E-06	9.18E-07	1.23E-05	6.68E-06	4.33E-06	6.23E-06	1.51E-05

**LAGPLS**	5.91E-07	5.10E-04	1.91E-04	1.46E-04	4.19E-04	8.12E-04	1.44E-04	nan	3.32E-04
**LAGPLSSVSARL**	1.55E-05	1.53E-04	1.96E-04	8.03E-05	1.82E-04	2.25E-04	7.59E-04	2.74E-04	6.19E-05
**GPLSSVSAR**	3.35E-06	5.70E-04	1.62E-04	1.99E-04	nan	1.36E-04	2.99E-03	2.88E-03	2.02E-03
**LAGWHK**	2.69E-06	2.03E-05	4.02E-06	6.92E-06	2.84E-05	3.49E-05	1.93E-05	4.21E-05	6.34E-05
**YLTRNQIQP**	7.04E-06	5.53E-04	1.31E-04	1.07E-04	6.95E-04	1.72E-03	4.60E-04	1.62E-03	2.45E-03
**NQIQPIAEREGD**	1.55E-06	7.31E-04	5.33E-04	3.01E-04	1.73E-03	8.23E-04	4.35E-04	1.25E-03	2.01E-03
**IAEREGDWSAMSG**	2.27E-06	1.12E-03	8.71E-04	9.98E-05	2.04E-03	1.93E-03	1.80E-03	2.66E-03	3.52E-03
**REGDWSAMSGF**	7.21E-06	6.85E-05	1.36E-04	6.83E-05	1.71E-04	1.73E-04	1.93E-04	2.39E-04	2.49E-04
**DWSAM**	1.18E-06	4.31E-04	2.67E-04	8.90E-05	8.64E-04	7.73E-04	5.74E-04	1.35E-03	1.29E-03
**WSAM**	3.11E-05	6.93E-04	5.91E-04	6.53E-05	9.89E-04	1.05E-03	5.34E-04	nan	1.30E-03
**SAMSGFQ**	1.49E-05	1.33E-03	2.05E-04	5.19E-04	1.17E-03	3.70E-05	1.52E-03	nan	2.59E-03
**SGFQQTMQM**	3.13E-06	1.78E-05	3.04E-05	4.95E-05	2.24E-05	2.20E-06	5.62E-06	1.14E-05	5.22E-05
**QTMQML**	8.91E-07	3.73E-06	3.28E-06	1.43E-05	4.26E-06	2.47E-06	2.52E-06	2.21E-05	1.18E-04
**NEGIVPTAML**	4.72E-05	4.45E-04	3.81E-04	3.10E-04	4.67E-04	5.15E-04	7.91E-04	1.69E-03	6.05E-03
**LVANDQMALGAM**	2.47E-05	2.17E-04	nan	3.52E-04	3.60E-04	3.62E-04	2.77E-04	2.98E-04	2.86E-04
**VANDQMALGAMR**	4.82E-07	1.31E-04	7.56E-05	1.25E-04	1.59E-04	5.17E-05	2.24E-05	1.43E-04	9.48E-04
**AITESGLRVGAD**	1.97E-06	9.69E-04	1.51E-04	3.84E-04	8.03E-04	6.88E-04	2.95E-04	1.18E-03	1.44E-03
**ITESGLRVGAD**	3.26E-06	2.51E-04	2.24E-04	2.01E-04	2.34E-04	1.93E-04	2.01E-04	2.78E-04	1.75E-04
**ADISVVGY**	8.31E-06	8.45E-05	2.56E-05	2.10E-04	1.17E-04	4.30E-05	4.65E-05	2.66E-05	4.32E-04
**VVGYDD**	2.86E-06	1.10E-04	1.20E-04	1.83E-04	1.58E-04	8.58E-05	3.27E-04	1.29E-04	3.38E-04
**DDTEDSSCYIPPLTTIK**	8.41E-07	2.70E-04	2.51E-04	2.08E-04	5.81E-04	5.93E-04	1.89E-04	8.19E-04	1.04E-03
**TEDSSCYIPPLTTIK**	3.36E-06	6.03E-05	6.72E-05	1.92E-04	2.17E-04	3.95E-04	3.33E-05	4.18E-04	3.57E-04
**SSCYIPPLTTIKQD**	1.06E-06	8.07E-05	8.33E-05	1.49E-04	2.42E-04	2.87E-04	3.54E-05	1.82E-04	3.62E-04
**FRLL**	3.36E-07	5.90E-06	9.02E-06	2.09E-05	2.73E-06	9.68E-05	3.17E-05	1.21E-04	2.23E-04
**FRLLGQTSV**	2.41E-05	3.33E-05	nan	2.93E-05	3.79E-05	3.54E-05	4.94E-05	3.65E-05	4.99E-05
**LGQTSVDR**	4.73E-07	5.74E-05	4.59E-05	7.67E-05	6.59E-05	2.51E-05	3.73E-05	7.02E-05	2.20E-04
**GQTSVDRL**	3.19E-06	2.79E-05	3.42E-05	3.82E-05	7.97E-05	5.12E-05	2.72E-05	nan	nan
**LLQL**	6.77E-07	1.49E-04	4.78E-05	1.65E-04	8.10E-05	4.83E-04	7.54E-05	6.34E-04	4.22E-04

IPTG vs DNA

Peptide	0 s	30 s	45 s	60 s	300 s	1500 s	3600 s	7200 s	14400 s
LIGVA	5.57E-06	1.59E-05	8.18E-05	3.85E-05	2.50E-05	4.34E-06	6.18E-07	3.30E-06	2.42E-05
ALHAP	1.70E-06	3.74E-05	2.47E-04	7.79E-05	1.08E-04	2.86E-04	2.66E-04	7.32E-04	nan
ALHAPSQIVA	1.00E-06	9.70E-06	nan	7.45E-05	4.57E-05	2.63E-05	1.05E-04	3.88E-05	5.84E-05
ALHAPSQIVAA	3.01E-06	4.60E-05	nan	1.38E-04	8.52E-05	5.69E-05	1.64E-04	1.85E-04	4.43E-04
ALHAPSQIVAAIK	8.39E-06	nan	2.30E-04	5.68E-05	6.04E-05	6.10E-05	5.68E-04	2.14E-04	2.04E-03
SQIVAA	5.76E-06	2.23E-05	1.35E-05	3.85E-05	3.72E-05	7.75E-05	5.68E-06	6.12E-05	4.57E-04
SQIVAAIK	5.76E-06	2.80E-05	nan	8.23E-06	7.33E-05	8.82E-07	1.14E-04	5.80E-05	5.35E-04
IVAAIK	3.31E-06	1.46E-05	5.08E-05	5.17E-06	9.98E-06	5.14E-07	2.69E-05	7.46E-06	1.81E-04
AIKSRADQLGAS	4.01E-07	8.56E-05	2.70E-04	2.43E-04	1.83E-04	1.45E-04	3.02E-04	3.88E-04	1.26E-03
ADQLGAS	2.70E-05	nan	7.18E-04	nan	1.39E-04	nan	2.03E-03	nan	5.35E-03
VVVSM	6.23E-07	2.40E-04	6.20E-04	1.75E-04	2.13E-04	3.79E-04	1.93E-04	8.29E-04	1.02E-03
MVERSGVE	2.46E-07	8.40E-04	nan	7.52E-04	1.08E-03	7.82E-04	9.84E-04	1.20E-03	5.70E-04
AAVHNL	4.34E-05	4.42E-05	1.51E-04	2.60E-05	4.09E-04	1.29E-03	1.83E-03	1.19E-03	2.24E-03
LAQRVSGL	1.84E-06	2.41E-04	2.11E-04	3.82E-06	1.43E-04	3.21E-04	2.26E-04	3.77E-04	1.12E-03
VSGLIIN	1.56E-05	3.39E-06	2.16E-05	1.53E-05	2.48E-05	1.10E-06	5.26E-05	6.90E-05	8.74E-06
IINYPLD	8.57E-06	1.36E-05	1.68E-05	1.42E-05	4.98E-05	2.10E-05	6.90E-05	7.86E-05	6.26E-04
LDDQDAIA	2.23E-05	7.13E-04	6.66E-04	6.94E-04	7.29E-04	8.07E-04	9.07E-04	1.46E-03	3.55E-03
IAVEAA	7.73E-07	nan	8.99E-04	4.45E-06	3.95E-05	1.64E-04	8.48E-05	3.75E-04	9.43E-04
ACTNVPAL	2.47E-05	9.16E-04	3.36E-04	9.09E-07	3.23E-04	5.83E-04	5.59E-04	1.82E-03	4.17E-03
ALFLD	4.67E-06	4.44E-05	1.81E-04	4.42E-05	7.17E-05	4.22E-05	1.39E-04	4.77E-05	1.14E-04
FLDVSDQTPINS	5.76E-07	1.09E-04	nan	2.44E-04	1.74E-04	3.96E-04	4.58E-04	6.71E-04	4.05E-04
DQTPIN	1.22E-05	2.48E-04	5.55E-04	6.68E-05	9.27E-05	1.09E-03	5.73E-04	6.93E-04	2.39E-03
SIIFSH	1.01E-05	2.80E-04	3.18E-04	3.27E-04	1.16E-04	2.80E-04	3.02E-04	1.36E-04	9.05E-04
SIIFSHEDGTRL	4.08E-06	1.75E-05	7.46E-06	2.26E-05	3.47E-05	3.34E-05	4.31E-05	5.93E-05	1.08E-04
EDGTRLGVEH	6.59E-06	1.34E-05	2.11E-05	6.66E-05	1.34E-04	9.35E-06	1.50E-04	3.25E-05	1.73E-04
LVAL	4.55E-09	2.98E-06	1.75E-05	4.73E-06	1.04E-05	7.73E-07	1.13E-05	4.71E-06	1.18E-04
LVALGHQQIAL	4.02E-06	4.22E-04	2.45E-04	1.84E-04	3.19E-04	3.89E-04	4.46E-04	7.23E-04	1.40E-03
LGHQQIALL	4.51E-07	4.08E-05	9.62E-05	3.89E-05	6.92E-05	3.99E-05	3.94E-05	7.20E-05	5.05E-05
IALL	3.53E-06	1.70E-06	nan	6.77E-06	8.75E-06	8.47E-06	5.04E-06	4.55E-08	4.79E-06

**LAGPLS**	5.17E-06	3.25E-04	1.97E-04	8.40E-05	4.88E-05	nan	3.52E-04	9.28E-04	nan
**LAGPLSSVSARL**	3.04E-04	4.21E-04	4.51E-04	8.76E-05	1.22E-04	3.60E-04	3.54E-04	4.08E-04	7.73E-04
**GPLSSVSAR**	1.06E-06	2.23E-04	1.14E-04	1.08E-04	1.51E-05	1.16E-03	6.48E-04	1.51E-03	4.50E-03
**LAGWHK**	9.17E-05	4.43E-05	8.03E-05	2.22E-06	7.89E-05	5.05E-05	3.13E-05	3.06E-05	2.49E-06
**YLTRNQIQP**	6.02E-06	4.86E-04	3.52E-04	4.20E-05	3.06E-04	7.50E-04	4.86E-04	9.99E-04	nan
**NQIQPIAEREGD**	3.96E-06	3.68E-04	1.70E-04	1.39E-04	1.07E-03	5.29E-04	5.64E-04	1.03E-03	4.66E-03
**IAEREGDWSAMSG**	7.27E-07	9.56E-04	1.04E-03	9.95E-05	8.83E-04	1.08E-03	1.84E-03	1.60E-03	5.37E-03
**REGDWSAMSGF**	8.23E-06	4.98E-05	3.62E-04	1.17E-04	1.73E-04	5.49E-04	2.68E-04	3.83E-04	9.82E-04
**DWSAM**	5.24E-06	4.26E-04	5.64E-04	8.15E-05	3.67E-04	5.46E-04	4.08E-04	6.74E-04	nan
**WSAM**	9.44E-05	1.59E-03	1.09E-03	7.30E-05	5.95E-04	nan	9.23E-04	1.17E-03	nan
**SAMSGFQ**	1.04E-05	1.30E-03	1.07E-03	nan	6.92E-04	3.73E-06	5.11E-04	nan	nan
**SGFQQTMQM**	3.72E-06	2.36E-05	2.05E-05	4.95E-06	3.35E-05	8.53E-06	1.25E-05	1.88E-05	7.87E-05
**QTMQML**	1.14E-05	3.62E-06	nan	8.19E-06	1.86E-06	1.55E-06	8.82E-07	4.67E-06	5.08E-05
**NEGIVPTAML**	4.63E-05	4.35E-04	3.64E-03	5.91E-06	4.75E-04	5.13E-04	2.48E-03	1.36E-03	7.47E-03
**LVANDQMALGAM**	2.17E-06	1.53E-04	nan	3.15E-04	2.64E-04	2.46E-04	1.58E-04	1.95E-04	1.74E-04
**VANDQMALGAMR**	1.55E-07	1.30E-04	2.84E-04	1.32E-04	2.06E-04	6.69E-05	2.74E-04	1.64E-04	7.58E-04
**AITESGLRVGAD**	6.88E-06	6.62E-04	4.53E-04	2.86E-04	4.19E-04	7.01E-04	4.52E-04	1.10E-03	1.92E-03
**ITESGLRVGAD**	8.15E-07	2.19E-04	1.83E-04	2.18E-04	2.88E-04	2.66E-04	2.96E-04	3.32E-04	4.67E-04
**ADISVVGY**	1.33E-05	1.60E-04	1.31E-04	3.69E-04	1.91E-04	7.87E-05	1.03E-04	3.39E-05	8.82E-05
**VVGYDD**	2.46E-06	1.63E-04	1.25E-04	2.35E-04	2.21E-04	9.14E-05	4.04E-04	1.14E-04	2.60E-04
**DDTEDSSCYIPPLTTIK**	7.14E-07	2.16E-04	1.77E-04	1.90E-04	4.80E-04	4.24E-04	1.18E-04	4.85E-04	1.20E-03
**TEDSSCYIPPLTTIK**	2.42E-06	7.45E-05	8.66E-05	6.37E-05	2.41E-04	1.14E-04	7.65E-05	2.19E-04	4.97E-04
**SSCYIPPLTTIKQD**	2.66E-06	6.70E-05	1.10E-04	1.28E-04	2.42E-04	6.57E-05	1.64E-04	1.38E-04	3.32E-04
**FRLL**	1.55E-07	7.71E-06	6.34E-05	2.27E-06	5.93E-06	2.83E-05	7.97E-05	1.15E-04	4.44E-04
**FRLLGQTSV**	3.84E-06	2.30E-05	nan	2.87E-06	3.16E-05	1.03E-06	7.72E-05	2.10E-05	1.32E-04
**LGQTSVDR**	5.68E-07	5.74E-05	3.15E-05	9.01E-05	6.59E-05	2.94E-05	4.40E-05	4.52E-05	1.00E-04
**GQTSVDRL**	3.07E-06	2.30E-05	2.96E-05	3.57E-05	8.80E-05	3.05E-05	5.98E-05	nan	nan
**LLQL**	3.79E-06	1.49E-04	7.89E-04	4.10E-05	3.09E-05	4.29E-04	2.17E-04	4.17E-04	2.70E-03

IPTG vs ONPF

Peptide	0 s	30 s	45 s	60 s	300 s	1500 s	3600 s	7200 s	14400 s
LIGVA	7.04E-06	5.69E-06	9.01E-05	3.76E-05	1.70E-05	1.76E-05	4.73E-07	7.06E-06	5.02E-05
ALHAP	7.73E-07	9.08E-05	3.24E-04	6.75E-05	3.65E-04	5.66E-04	2.16E-04	4.42E-04	2.12E-03
ALHAPSQIVA	7.30E-06	2.02E-05	nan	3.56E-05	2.51E-05	2.98E-05	1.37E-04	4.69E-05	2.78E-05
ALHAPSQIVAA	7.35E-07	2.76E-05	nan	1.64E-04	3.34E-05	9.40E-05	1.04E-04	1.61E-04	2.45E-05
ALHAPSQIVAAIK	nan	nan	nan	nan	nan	nan	nan	nan	nan
SQIVAA	5.86E-05	nan	nan	5.59E-05	nan	nan	7.21E-04	2.98E-04	nan
SQIVAAIK	2.31E-06	1.63E-05	nan	6.30E-06	1.02E-04	4.35E-05	1.11E-03	2.18E-04	nan
IVAAIK	4.09E-08	1.19E-05	1.79E-04	5.60E-06	2.07E-05	1.55E-05	2.29E-04	4.69E-05	1.63E-03
AIKSRADQLGAS	1.36E-06	6.26E-05	2.82E-04	8.93E-05	6.63E-05	1.89E-04	2.67E-04	4.16E-04	5.73E-04
ADQLGAS	2.57E-04	nan	6.40E-05	nan	nan	nan	2.62E-03	nan	nan
VVVSM	1.05E-05	3.79E-04	1.07E-03	1.11E-04	8.55E-04	7.90E-04	3.19E-04	5.33E-04	1.90E-03
MVERSGVE	5.16E-06	7.20E-04	nan	5.63E-04	5.84E-04	5.74E-04	4.81E-04	6.68E-04	3.57E-04
AAVHNL	4.44E-05	4.03E-05	5.99E-05	3.18E-05	2.09E-04	1.44E-03	1.46E-03	1.10E-03	nan
LAQRVSGL	2.19E-06	2.33E-04	5.82E-04	7.07E-06	2.78E-04	4.34E-04	4.09E-04	3.90E-04	4.59E-03
VSGLIIN	2.48E-05	4.33E-06	5.30E-06	1.68E-05	2.44E-05	2.02E-06	2.50E-05	9.60E-06	8.74E-06
IINYPLD	6.63E-06	9.84E-06	7.15E-06	1.49E-05	4.53E-05	6.34E-06	5.51E-05	4.09E-05	3.63E-04
LDDQDAIA	2.24E-05	9.58E-04	1.41E-03	3.69E-04	9.87E-04	1.01E-03	8.93E-04	1.01E-03	nan
IAVEAA	1.71E-06	5.60E-05	5.64E-05	4.45E-06	4.95E-05	1.14E-04	3.81E-04	3.61E-04	4.80E-03
ACTNVPAL	7.50E-06	9.59E-04	1.09E-03	1.85E-05	1.09E-03	1.14E-03	8.15E-04	8.98E-04	nan
ALFLD	5.82E-06	3.63E-05	4.30E-05	3.19E-05	2.93E-05	1.68E-05	8.35E-05	2.52E-04	nan
FLDVSDQTPINS	7.57E-06	1.57E-04	nan	2.26E-04	1.96E-04	4.15E-04	5.25E-04	8.03E-04	1.49E-03
DQTPIN	2.77E-05	2.62E-04	3.78E-04	7.16E-05	6.18E-04	1.68E-03	6.05E-04	5.56E-04	nan
SIIFSH	nan	nan	nan	nan	nan	nan	nan	nan	nan
SIIFSHEDGTRL	4.78E-06	1.45E-05	nan	1.14E-05	2.77E-05	1.74E-05	3.45E-05	4.67E-05	1.32E-04
EDGTRLGVEH	4.48E-06	1.47E-05	6.40E-05	3.06E-05	1.57E-04	3.36E-05	1.62E-04	7.40E-05	nan
LVAL	5.50E-07	1.16E-06	7.15E-06	4.82E-06	9.43E-06	7.86E-07	3.97E-06	2.41E-06	2.63E-05
LVALGHQQIAL	9.09E-07	4.40E-04	4.18E-04	1.77E-04	4.00E-04	4.54E-04	3.64E-04	5.34E-04	1.46E-03
LGHQQIALL	1.05E-05	4.14E-05	7.35E-05	2.66E-05	5.32E-05	4.39E-05	5.19E-05	6.20E-05	3.56E-05
IALL	1.62E-06	3.79E-06	1.06E-06	2.55E-06	5.02E-06	2.86E-06	7.13E-06	5.26E-06	nan

**LAGPLS**	2.06E-05	3.08E-04	1.32E-04	8.04E-05	2.17E-04	1.42E-03	1.28E-03	9.37E-04	nan
**LAGPLSSVSARL**	1.61E-05	1.48E-04	7.38E-04	1.09E-04	4.08E-04	5.54E-04	6.56E-04	6.22E-04	2.88E-03
**GPLSSVSAR**	1.86E-06	3.62E-04	1.77E-03	1.24E-04	2.16E-04	1.35E-04	8.90E-04	6.68E-04	3.01E-03
**LAGWHK**	2.40E-05	1.37E-06	8.82E-06	8.11E-07	2.86E-05	3.72E-05	1.90E-05	2.45E-05	2.49E-06
**YLTRNQIQP**	6.73E-06	4.89E-04	1.31E-03	4.34E-05	5.19E-04	7.94E-04	4.10E-04	6.54E-04	1.33E-03
**NQIQPIAEREGD**	1.11E-06	3.86E-04	8.25E-04	1.33E-04	2.05E-03	8.60E-04	5.09E-04	6.94E-04	nan
**IAEREGDWSAMSG**	3.39E-06	1.08E-03	1.41E-03	1.17E-04	1.62E-03	1.94E-03	1.60E-03	1.23E-03	8.56E-03
**REGDWSAMSGF**	6.55E-06	5.87E-05	nan	1.61E-04	2.23E-04	1.88E-04	5.46E-04	1.74E-04	1.40E-04
**DWSAM**	7.20E-06	4.36E-04	3.86E-04	7.28E-05	1.00E-03	1.16E-03	5.65E-04	5.15E-04	nan
**WSAM**	8.85E-05	7.36E-04	9.14E-04	6.12E-05	1.10E-03	1.50E-03	6.28E-04	nan	5.55E-04
**SAMSGFQ**	5.20E-06	1.53E-03	nan	4.78E-04	2.55E-03	2.30E-04	3.28E-04	nan	nan
**SGFQQTMQM**	3.26E-06	1.90E-05	3.53E-05	2.08E-05	2.25E-05	3.38E-06	1.09E-05	2.41E-05	2.98E-05
**QTMQML**	4.09E-06	1.11E-05	5.73E-06	7.14E-06	1.75E-06	1.55E-06	1.43E-06	6.66E-06	3.60E-05
**NEGIVPTAML**	4.57E-05	5.59E-04	2.50E-03	3.97E-06	7.63E-04	1.01E-03	1.76E-03	7.43E-04	1.31E-03
**LVANDQMALGAM**	3.25E-06	1.14E-04	nan	3.07E-04	2.29E-04	4.06E-04	2.06E-04	2.32E-04	1.86E-04
**VANDQMALGAMR**	4.82E-07	1.11E-04	1.81E-04	1.36E-04	2.04E-04	1.07E-04	1.65E-04	1.31E-04	6.99E-04
**AITESGLRVGAD**	6.59E-07	7.33E-04	7.36E-04	2.68E-04	6.96E-04	8.20E-04	4.34E-04	7.00E-04	2.41E-03
**ITESGLRVGAD**	5.33E-06	1.72E-04	3.35E-04	8.53E-05	1.88E-04	1.40E-04	1.62E-04	1.46E-04	4.83E-04
**ADISVVGY**	8.91E-06	9.84E-05	2.95E-05	2.15E-04	1.20E-04	2.07E-04	6.56E-05	2.86E-05	2.10E-04
**VVGYDD**	9.09E-07	1.16E-04	1.54E-04	2.13E-04	2.05E-04	1.74E-04	5.96E-04	1.79E-04	1.01E-03
**DDTEDSSCYIPPLTTIK**	2.19E-06	2.44E-04	7.32E-04	2.33E-04	6.61E-04	7.39E-04	8.23E-04	8.13E-04	nan
**TEDSSCYIPPLTTIK**	2.24E-06	5.75E-05	4.79E-04	1.05E-04	2.38E-04	1.93E-04	1.84E-04	1.89E-04	nan
**SSCYIPPLTTIKQD**	1.39E-06	8.45E-05	4.40E-04	1.57E-04	2.66E-04	1.58E-04	4.52E-04	2.58E-04	7.57E-03
**FRLL**	5.68E-07	7.36E-06	7.20E-05	3.77E-06	5.24E-06	3.19E-05	1.72E-04	1.24E-04	1.70E-03
**FRLLGQTSV**	4.48E-06	2.42E-05	nan	4.59E-05	2.72E-05	9.80E-06	5.91E-05	3.44E-05	4.75E-05
**LGQTSVDR**	4.73E-07	5.28E-05	5.55E-05	6.36E-05	6.15E-05	3.16E-05	6.26E-05	1.31E-04	5.31E-04
**GQTSVDRL**	3.53E-06	2.18E-05	2.96E-05	3.34E-05	7.68E-05	3.12E-05	4.12E-05	nan	nan
**LLQL**	6.77E-07	1.84E-04	4.85E-04	4.61E-05	2.98E-04	9.03E-04	8.73E-04	7.37E-04	4.70E-03

IPTG vs ONPFDNA

Peptide	0 s	30 s	45 s	60 s	300 s	1500 s	3600 s	7200 s	14400 s
LIGVA	6.73E-06	2.55E-05	6.92E-05	3.59E-05	1.43E-05	7.97E-06	9.09E-07	3.57E-06	4.21E-05
ALHAP	5.14E-07	2.10E-05	7.80E-06	5.89E-05	1.17E-04	2.84E-04	9.93E-05	3.37E-04	2.85E-04
ALHAPSQIVA	3.25E-06	1.09E-04	nan	1.22E-04	5.04E-05	2.23E-04	1.31E-04	8.17E-05	3.46E-04
ALHAPSQIVAA	3.14E-07	1.01E-04	nan	1.48E-04	6.74E-05	1.57E-04	1.95E-04	1.37E-04	2.59E-04
ALHAPSQIVAAIK	7.94E-06	nan	1.44E-05	6.68E-05	7.53E-05	4.04E-05	1.78E-04	3.56E-04	2.62E-05
SQIVAA	2.78E-05	1.53E-05	2.24E-06	2.40E-05	nan	1.97E-04	1.31E-05	2.27E-06	1.54E-05
SQIVAAIK	2.27E-06	1.85E-05	2.76E-05	8.42E-06	5.17E-05	4.86E-06	1.93E-04	1.21E-04	4.83E-05
IVAAIK	1.16E-06	1.12E-05	5.91E-06	5.84E-06	6.11E-06	1.25E-06	1.71E-05	4.07E-05	7.57E-06
AIKSRADQLGAS	3.22E-07	1.15E-04	5.77E-05	9.96E-05	1.02E-04	2.85E-04	2.11E-04	2.66E-04	6.35E-04
ADQLGAS	4.48E-05	nan	6.25E-05	nan	5.39E-04	nan	1.96E-03	4.25E-03	2.58E-03
VVVSM	5.27E-07	2.64E-04	3.16E-04	1.13E-04	2.71E-04	4.45E-04	4.74E-05	4.04E-04	7.38E-04
MVERSGVE	6.64E-07	1.39E-03	nan	6.11E-04	5.73E-04	6.53E-04	4.99E-04	6.94E-04	6.55E-04
AAVHNL	6.98E-05	2.56E-05	2.19E-06	4.78E-05	3.74E-04	1.29E-03	1.49E-03	nan	1.66E-03
LAQRVSGL	1.98E-06	2.30E-04	8.92E-05	1.98E-06	1.39E-04	3.31E-04	1.85E-04	3.25E-04	4.78E-04
VSGLIIN	2.42E-05	8.79E-06	1.80E-05	8.47E-06	1.29E-04	1.86E-06	2.44E-05	3.52E-05	7.44E-06
IINYPLD	6.39E-06	8.18E-06	1.05E-05	2.61E-05	3.34E-05	4.06E-05	6.34E-05	3.25E-05	6.52E-05
LDDQDAIA	2.29E-05	6.66E-04	3.33E-04	3.24E-04	6.75E-04	8.23E-04	3.22E-04	7.45E-04	1.07E-03
IAVEAA	7.14E-07	1.78E-05	6.43E-05	1.54E-05	7.90E-06	2.23E-04	6.83E-05	3.64E-04	8.60E-04
ACTNVPAL	7.93E-06	9.63E-04	2.73E-06	1.63E-05	2.96E-04	5.89E-04	7.80E-05	7.19E-04	7.57E-04
ALFLD	6.13E-06	3.17E-05	nan	8.95E-05	4.60E-04	3.32E-05	1.85E-04	1.39E-04	2.78E-04
FLDVSDQTPINS	9.30E-07	2.20E-04	nan	7.46E-05	1.60E-04	2.58E-04	4.91E-04	3.42E-04	3.55E-04
DQTPIN	1.38E-05	2.67E-04	1.52E-04	7.09E-05	1.93E-04	1.10E-03	2.92E-04	3.73E-04	3.82E-04
SIIFSH	2.47E-04	3.77E-04	8.13E-04	5.90E-04	5.56E-04	4.83E-04	1.07E-03	9.57E-04	nan
SIIFSHEDGTRL	3.99E-06	1.63E-05	nan	1.48E-05	4.88E-05	2.87E-05	4.72E-05	7.39E-05	3.68E-05
EDGTRLGVEH	2.53E-06	2.19E-05	1.89E-05	3.75E-05	1.31E-04	2.22E-05	1.87E-04	2.82E-05	4.03E-05
LVAL	4.09E-08	1.45E-06	2.75E-06	4.77E-06	9.28E-06	4.08E-06	6.39E-06	2.01E-06	3.01E-05
LVALGHQQIAL	4.95E-07	4.18E-04	1.90E-04	1.74E-04	3.31E-04	3.77E-04	3.06E-04	6.40E-04	4.43E-04
LGHQQIALL	1.23E-06	6.19E-05	1.52E-04	8.14E-05	6.51E-05	2.35E-05	2.88E-05	5.37E-05	2.23E-05
IALL	1.93E-06	5.14E-07	6.95E-07	7.11E-06	5.31E-06	5.27E-07	3.68E-06	3.73E-07	2.69E-05

**LAGPLS**	2.04E-06	3.71E-04	2.74E-05	8.88E-05	4.21E-05	6.98E-04	1.81E-05	nan	1.67E-04
**LAGPLSSVSARL**	2.12E-06	1.88E-04	2.48E-04	5.86E-05	2.17E-04	2.14E-04	2.19E-04	5.68E-04	3.21E-04
**GPLSSVSAR**	1.14E-07	2.66E-04	3.29E-04	1.05E-04	1.33E-05	1.42E-04	4.94E-04	9.71E-04	1.73E-03
**LAGWHK**	6.26E-06	2.76E-05	4.56E-06	6.48E-06	5.59E-05	3.94E-05	nan	1.41E-05	8.98E-05
**YLTRNQIQP**	5.59E-06	5.31E-04	1.34E-04	9.01E-05	3.00E-04	8.72E-04	2.27E-04	6.57E-04	nan
**NQIQPIAEREGD**	9.64E-07	3.56E-04	9.24E-05	1.31E-04	1.09E-03	6.24E-04	2.18E-04	7.92E-04	nan
**IAEREGDWSAMSG**	8.41E-07	1.01E-03	6.43E-04	1.00E-04	1.05E-03	1.15E-03	1.03E-03	8.92E-04	1.21E-03
**REGDWSAMSGF**	2.86E-05	1.15E-04	4.84E-04	1.54E-04	1.60E-04	1.67E-04	1.76E-04	1.03E-04	1.83E-04
**DWSAM**	7.45E-07	4.20E-04	2.26E-04	3.60E-05	4.48E-04	7.34E-04	1.01E-04	3.08E-04	3.71E-04
**WSAM**	2.82E-05	6.68E-04	4.04E-04	1.52E-04	5.20E-04	7.31E-04	7.87E-05	4.13E-04	nan
**SAMSGFQ**	6.39E-05	1.27E-03	1.93E-04	4.13E-04	9.60E-04	2.64E-05	3.21E-04	nan	7.93E-05
**SGFQQTMQM**	6.26E-06	1.64E-05	3.57E-05	nan	2.38E-05	1.09E-05	6.57E-06	2.19E-05	3.00E-05
**QTMQML**	7.64E-06	3.53E-06	3.65E-06	6.23E-06	2.01E-06	1.03E-05	7.68E-07	5.02E-06	6.09E-05
**NEGIVPTAML**	6.94E-05	5.38E-04	8.85E-04	9.45E-07	4.17E-04	7.39E-04	7.57E-04	5.08E-04	1.96E-03
**LVANDQMALGAM**	1.21E-04	1.97E-03	nan	3.91E-03	3.06E-03	4.73E-03	3.93E-03	2.16E-03	5.08E-03
**VANDQMALGAMR**	2.27E-07	1.16E-04	1.26E-04	1.40E-04	1.69E-04	5.21E-05	1.80E-04	1.84E-04	1.85E-04
**AITESGLRVGAD**	6.73E-07	6.39E-04	2.26E-04	2.45E-04	4.26E-04	6.73E-04	1.94E-04	6.61E-04	5.64E-04
**ITESGLRVGAD**	4.37E-06	2.68E-04	1.09E-04	1.65E-04	1.45E-04	1.06E-04	1.46E-04	1.42E-04	1.39E-04
**ADISVVGY**	8.67E-06	1.16E-04	8.90E-05	2.15E-04	1.29E-04	6.02E-05	7.40E-05	5.53E-05	4.68E-05
**VVGYDD**	6.77E-07	1.30E-04	1.43E-04	1.95E-04	1.57E-04	6.87E-05	3.22E-04	1.22E-04	6.21E-05
**DDTEDSSCYIPPLTTIK**	7.73E-07	2.09E-04	2.58E-04	2.00E-04	5.36E-04	4.22E-04	9.00E-05	3.74E-04	6.15E-04
**TEDSSCYIPPLTTIK**	2.42E-06	6.47E-05	7.62E-05	6.32E-05	2.68E-04	1.14E-04	7.14E-05	1.80E-04	2.32E-04
**SSCYIPPLTTIKQD**	1.25E-06	8.45E-05	7.36E-05	1.30E-04	2.33E-04	6.82E-05	2.52E-05	9.94E-05	1.81E-04
**FRLL**	5.68E-07	6.91E-06	6.99E-06	5.27E-07	2.64E-06	4.07E-05	6.05E-05	1.02E-04	8.08E-05
**FRLLGQTSV**	5.82E-05	3.06E-05	nan	2.94E-06	2.35E-05	4.45E-05	4.11E-05	1.45E-05	6.09E-05
**LGQTSVDR**	4.55E-07	4.57E-05	2.75E-05	6.30E-05	4.99E-05	1.98E-05	2.26E-05	4.78E-05	2.36E-05
**GQTSVDRL**	3.24E-06	2.13E-05	2.13E-05	3.36E-05	7.04E-05	2.50E-05	3.05E-05	nan	nan
**LLQL**	1.70E-06	1.54E-04	1.44E-04	4.55E-05	7.69E-05	4.29E-04	2.76E-04	3.94E-04	6.98E-04

IPTG vs TMG

Peptide	0 s	30 s	45 s	60 s	300 s	1500 s	3600 s	7200 s	14400 s
LIGVA	6.02E-06	2.78E-06	6.96E-05	2.77E-05	7.27E-07	3.33E-06	1.42E-05	3.72E-06	2.11E-05
ALHAP	5.91E-07	5.24E-05	1.22E-05	2.37E-04	5.39E-05	4.22E-04	3.23E-04	5.55E-04	4.77E-04
ALHAPSQIVA	1.50E-06	2.33E-05	nan	6.83E-04	1.07E-05	2.20E-04	3.91E-05	3.61E-04	1.82E-04
ALHAPSQIVAA	1.14E-06	4.27E-05	nan	6.73E-04	3.01E-05	1.93E-04	1.18E-05	3.64E-04	1.82E-04
ALHAPSQIVAAIK	1.13E-05	nan	1.78E-04	nan	1.84E-04	3.44E-05	1.12E-03	1.62E-04	2.05E-06
SQIVAA	1.68E-05	1.68E-05	3.28E-05	1.00E-04	nan	2.97E-05	9.50E-05	1.81E-05	1.16E-05
SQIVAAIK	3.36E-06	1.88E-05	2.60E-05	9.53E-05	5.14E-05	2.84E-05	3.78E-05	1.17E-06	5.52E-05
IVAAIK	1.82E-06	1.19E-05	5.02E-06	4.77E-05	6.78E-06	1.50E-05	1.14E-05	9.75E-06	7.57E-06
AIKSRADQLGAS	1.02E-06	2.42E-04	4.25E-05	1.07E-04	8.18E-05	2.27E-04	3.99E-04	1.16E-03	6.45E-04
ADQLGAS	2.80E-05	nan	8.81E-05	nan	1.41E-04	nan	3.24E-03	1.87E-03	1.13E-03
VVVSM	6.12E-05	4.64E-04	1.52E-04	3.52E-05	6.88E-05	3.35E-04	2.10E-05	6.96E-04	4.69E-04
MVERSGVE	1.07E-05	2.92E-03	nan	4.51E-04	5.79E-04	1.25E-03	1.12E-03	1.44E-03	8.42E-04
AAVHNL	4.20E-05	2.59E-05	3.07E-04	6.08E-05	2.40E-04	1.33E-03	1.60E-03	1.21E-03	1.73E-03
LAQRVSGL	2.04E-06	3.94E-04	1.58E-04	4.55E-05	1.59E-04	3.69E-04	2.20E-04	6.51E-04	6.34E-04
VSGLIIN	7.65E-06	1.79E-06	3.89E-05	1.09E-05	2.57E-05	1.33E-05	2.60E-05	1.19E-05	1.20E-05
IINYPLD	2.19E-05	1.89E-05	6.42E-06	nan	3.06E-05	4.14E-05	5.21E-05	3.97E-05	5.60E-05
LDDQDAIA	2.85E-05	1.34E-03	2.37E-04	3.19E-04	5.34E-04	9.22E-04	3.20E-04	9.68E-04	9.02E-04
IAVEAA	5.55E-07	2.31E-05	1.12E-04	3.60E-06	1.15E-05	6.37E-05	5.81E-05	4.68E-04	8.45E-04
ACTNVPAL	3.15E-05	2.52E-03	3.93E-06	1.03E-04	4.33E-04	1.29E-03	4.56E-04	2.43E-03	1.30E-03
ALFLD	1.47E-05	1.05E-05	1.38E-04	4.53E-05	3.55E-05	1.16E-05	1.03E-04	9.84E-05	1.89E-04
FLDVSDQTPINS	5.82E-06	2.86E-04	nan	7.13E-04	7.44E-05	5.68E-04	4.87E-04	7.88E-04	3.12E-04
DQTPIN	9.47E-06	2.88E-04	2.64E-04	4.82E-04	6.70E-05	1.53E-03	4.71E-04	9.69E-04	4.52E-04
SIIFSH	3.14E-04	1.67E-04	8.16E-05	8.25E-04	1.62E-04	1.78E-04	7.08E-05	1.24E-04	1.72E-04
SIIFSHEDGTRL	4.13E-06	2.00E-04	2.08E-06	2.49E-04	5.21E-05	6.12E-05	1.43E-05	6.38E-05	5.28E-05
EDGTRLGVEH	1.86E-05	6.77E-05	3.30E-04	nan	2.29E-04	4.68E-04	3.70E-05	5.60E-05	4.61E-04
LVAL	7.27E-08	1.28E-06	8.76E-06	1.49E-04	9.21E-06	1.82E-05	8.26E-06	1.86E-06	2.65E-05
LVALGHQQIAL	4.55E-06	6.56E-04	1.41E-04	5.01E-04	2.88E-04	5.91E-04	2.52E-04	5.03E-04	5.16E-04
LGHQQIALL	7.23E-06	5.92E-05	7.59E-05	3.62E-05	4.58E-05	6.95E-05	3.01E-05	6.27E-05	3.49E-05
IALL	2.10E-06	6.45E-06	1.06E-06	6.82E-05	4.88E-06	1.63E-05	4.33E-06	3.32E-06	3.97E-06

**LAGPLS**	2.25E-05	7.64E-04	3.15E-05	5.03E-04	6.11E-05	8.55E-04	1.33E-05	9.46E-04	2.62E-04
**LAGPLSSVSARL**	2.60E-05	1.48E-04	1.03E-04	2.78E-04	5.03E-05	6.31E-04	1.70E-04	nan	nan
**GPLSSVSAR**	3.60E-06	6.09E-04	1.90E-04	nan	2.21E-05	5.40E-04	7.85E-04	8.71E-04	nan
**LAGWHK**	1.95E-05	1.69E-04	2.46E-04	nan	2.85E-05	5.41E-04	3.17E-05	2.82E-04	3.67E-04
**YLTRNQIQP**	6.88E-06	9.01E-04	nan	1.34E-04	2.55E-04	nan	4.77E-04	nan	1.76E-03
**NQIQPIAEREGD**	1.44E-06	1.25E-03	9.05E-05	4.85E-04	nan	9.93E-04	nan	9.72E-04	9.56E-04
**IAEREGDWSAMSG**	8.41E-07	1.50E-03	4.83E-04	7.93E-04	9.48E-04	2.01E-03	1.41E-03	1.83E-03	1.70E-03
**REGDWSAMSGF**	8.56E-06	4.02E-04	3.88E-04	3.39E-04	nan	3.35E-04	nan	8.14E-04	3.64E-04
**DWSAM**	1.35E-05	5.59E-04	3.01E-04	7.27E-04	3.73E-04	9.42E-04	3.27E-04	1.09E-03	5.77E-04
**WSAM**	1.64E-05	6.22E-04	4.49E-04	3.16E-04	3.50E-04	8.71E-04	3.14E-04	nan	5.42E-04
**SAMSGFQ**	1.75E-05	1.64E-03	8.46E-04	6.77E-04	7.08E-04	1.38E-04	3.91E-04	nan	9.43E-05
**SGFQQTMQM**	5.97E-06	1.75E-05	4.08E-05	nan	2.47E-05	1.32E-05	8.10E-06	1.22E-05	2.16E-05
**QTMQML**	1.82E-06	1.44E-05	1.59E-05	nan	2.10E-06	6.22E-06	1.93E-06	1.02E-05	3.73E-05
**NEGIVPTAML**	5.68E-05	6.78E-04	1.56E-03	8.67E-06	2.22E-04	7.47E-04	3.20E-04	1.52E-03	1.53E-03
**LVANDQMALGAM**	1.83E-06	2.10E-04	nan	nan	2.39E-04	3.50E-04	1.36E-04	1.43E-04	9.36E-05
**VANDQMALGAMR**	8.82E-07	1.82E-04	7.65E-05	nan	1.59E-04	2.48E-04	8.30E-06	6.29E-05	1.96E-04
**AITESGLRVGAD**	9.06E-06	1.39E-03	1.55E-04	7.46E-04	5.85E-04	1.34E-03	3.62E-04	1.14E-03	8.92E-04
**ITESGLRVGAD**	6.34E-06	1.15E-03	1.07E-04	3.70E-04	2.95E-04	7.08E-04	3.23E-04	8.48E-04	3.94E-04
**ADISVVGY**	9.04E-06	9.78E-05	2.77E-05	nan	1.17E-04	2.16E-04	5.13E-05	2.33E-05	8.14E-05
**VVGYDD**	8.23E-07	1.07E-04	1.01E-04	nan	1.59E-04	1.46E-04	2.91E-04	4.69E-05	1.08E-04
**DDTEDSSCYIPPLTTIK**	7.14E-07	3.48E-04	1.90E-04	nan	4.67E-04	6.82E-04	7.20E-05	9.79E-04	1.36E-03
**TEDSSCYIPPLTTIK**	4.82E-06	1.10E-04	1.87E-04	nan	2.11E-04	2.08E-04	2.48E-05	4.33E-04	5.20E-04
**SSCYIPPLTTIKQD**	1.14E-06	1.12E-04	1.85E-04	3.42E-03	2.27E-04	2.98E-04	3.20E-05	1.42E-04	3.71E-04
**FRLL**	3.36E-07	7.90E-06	5.35E-05	1.58E-04	5.69E-06	3.92E-05	3.69E-05	1.96E-04	1.25E-04
**FRLLGQTSV**	7.95E-06	7.38E-05	nan	1.02E-04	5.04E-05	5.21E-05	nan	3.14E-05	4.87E-05
**LGQTSVDR**	6.77E-07	4.94E-05	3.01E-05	2.10E-04	5.09E-05	7.18E-05	2.00E-05	3.02E-05	4.40E-05
**GQTSVDRL**	3.24E-06	nan	nan	1.08E-04	nan	8.06E-05	2.81E-05	nan	nan
**LLQL**	2.23E-06	2.05E-04	3.07E-04	9.96E-05	3.30E-05	5.52E-04	1.41E-04	1.14E-03	4.22E-04

ONPF vs APO

Peptide	0 s	30 s	45 s	60 s	300 s	1500 s	3600 s	7200 s	14400 s
LIGVA	4.79E-06	2.60E-05	2.58E-05	3.69E-05	4.98E-05	1.65E-05	3.33E-06	1.27E-05	7.54E-05
ALHAP	5.91E-07	2.94E-04	3.84E-04	5.43E-04	1.19E-03	5.70E-04	4.48E-04	5.65E-04	2.02E-03
ALHAPSQIVA	8.79E-06	4.36E-05	nan	6.12E-05	2.79E-05	3.55E-05	2.05E-04	1.14E-04	2.20E-05
ALHAPSQIVAA	5.35E-06	5.18E-05	nan	1.53E-04	8.70E-05	1.01E-04	1.90E-04	4.15E-04	1.23E-03
ALHAPSQIVAAIK	nan	nan	nan	nan	nan	nan	nan	nan	nan
SQIVAA	5.52E-05	nan	nan	1.06E-04	nan	nan	7.17E-04	1.06E-03	nan
SQIVAAIK	7.68E-07	7.44E-06	nan	1.76E-04	5.20E-05	4.97E-04	1.10E-03	4.69E-04	nan
IVAAIK	2.66E-06	1.64E-06	1.78E-04	1.41E-04	1.64E-05	2.33E-04	2.53E-04	1.26E-04	1.96E-03
AIKSRADQLGAS	3.26E-06	1.13E-04	3.51E-04	1.32E-05	1.13E-04	1.94E-04	2.71E-04	4.11E-04	5.02E-04
ADQLGAS	2.43E-04	nan	5.16E-04	4.02E-05	nan	9.58E-04	8.97E-04	nan	nan
VVVSM	1.08E-05	5.01E-04	1.19E-03	1.45E-04	1.81E-03	7.05E-04	5.94E-04	9.60E-04	2.12E-03
MVERSGVE	5.56E-06	7.94E-04	nan	1.16E-03	6.62E-04	5.53E-04	6.82E-04	5.75E-04	7.04E-04
AAVHNL	2.63E-05	1.57E-05	1.29E-04	6.39E-06	5.20E-05	1.48E-04	8.49E-05	1.99E-03	nan
LAQRVSGL	5.32E-07	3.75E-05	6.49E-04	5.48E-06	2.50E-04	1.17E-04	3.64E-04	3.71E-04	4.23E-03
VSGLIIN	6.83E-05	1.89E-05	4.73E-05	9.92E-05	2.57E-05	1.03E-05	1.10E-04	9.79E-05	1.28E-05
IINYPLD	3.35E-06	2.71E-06	2.75E-06	3.07E-06	1.66E-05	2.27E-07	2.66E-05	8.06E-05	4.84E-04
LDDQDAIA	1.82E-07	7.94E-04	1.82E-03	5.38E-04	nan	4.30E-04	1.04E-03	1.05E-03	nan
IAVEAA	1.53E-06	4.28E-05	5.33E-05	2.01E-04	6.29E-05	9.52E-05	4.69E-04	4.29E-04	4.16E-03
ACTNVPAL	1.25E-05	7.17E-04	1.92E-03	2.34E-04	2.31E-03	7.46E-04	8.70E-04	1.54E-03	nan
ALFLD	1.18E-06	2.93E-05	2.30E-05	4.73E-06	3.19E-06	5.96E-06	3.08E-06	2.22E-04	nan
FLDVSDQTPINS	1.53E-05	1.44E-04	nan	2.67E-04	1.58E-04	3.89E-04	3.74E-04	6.52E-04	1.27E-03
DQTPIN	2.09E-05	2.16E-05	2.89E-04	5.27E-05	1.17E-03	6.02E-04	5.28E-04	7.20E-04	nan
SIIFSH	nan	nan	nan	nan	nan	nan	nan	nan	nan
SIIFSHEDGTRL	2.65E-06	1.77E-05	nan	6.94E-06	2.91E-05	2.33E-05	4.92E-05	6.30E-05	1.90E-04
EDGTRLGVEH	3.39E-06	2.88E-05	5.55E-05	1.55E-07	2.91E-05	3.60E-05	1.44E-04	1.46E-04	nan
LVAL	6.64E-07	1.82E-08	5.02E-06	4.25E-06	6.77E-07	1.43E-05	2.13E-06	7.86E-07	3.76E-05
LVALGHQQIAL	1.35E-06	2.82E-04	5.12E-04	8.99E-05	2.96E-04	1.14E-04	2.69E-04	6.03E-04	1.70E-03
LGHQQIALL	1.41E-05	1.54E-05	nan	6.52E-07	3.43E-05	3.51E-05	5.64E-05	6.31E-05	2.88E-05
IALL	4.48E-06	1.08E-05	9.43E-06	2.37E-06	8.01E-06	8.63E-06	4.33E-06	1.15E-05	nan

**LAGPLS**	2.08E-05	2.03E-04	2.72E-04	6.55E-05	5.52E-04	8.80E-04	1.41E-03	nan	nan
**LAGPLSSVSARL**	2.94E-05	1.56E-04	7.30E-04	1.03E-04	4.90E-04	6.06E-04	1.27E-03	7.00E-04	2.88E-03
**GPLSSVSAR**	5.13E-06	4.99E-04	1.73E-03	1.32E-04	nan	8.46E-06	2.89E-03	3.19E-03	1.57E-03
**LAGWHK**	2.14E-05	1.96E-05	6.11E-06	6.13E-06	2.00E-07	3.84E-06	3.40E-06	4.39E-05	6.09E-05
**YLTRNQIQP**	2.64E-06	6.96E-05	1.24E-03	6.69E-05	7.90E-04	1.01E-03	4.18E-04	9.71E-04	1.44E-03
**NQIQPIAEREGD**	8.77E-07	4.09E-04	1.18E-03	1.71E-04	1.71E-03	6.51E-04	6.29E-04	1.01E-03	nan
**IAEREGDWSAMSG**	5.51E-06	4.04E-04	1.51E-03	2.78E-05	2.10E-03	1.72E-03	1.53E-03	2.11E-03	9.73E-03
**REGDWSAMSGF**	4.29E-06	3.54E-05	nan	1.34E-04	1.62E-04	1.28E-04	5.79E-04	2.40E-04	2.60E-04
**DWSAM**	7.80E-06	3.80E-05	2.90E-04	8.98E-05	1.23E-03	8.48E-04	1.07E-03	1.25E-03	nan
**WSAM**	8.69E-05	1.84E-04	9.42E-04	4.13E-06	1.44E-03	1.16E-03	1.02E-03	nan	1.27E-03
**SAMSGFQ**	1.68E-05	3.39E-04	nan	3.87E-04	2.34E-03	2.67E-04	1.23E-03	8.62E-04	nan
**SGFQQTMQM**	3.76E-06	4.06E-06	2.49E-05	6.04E-05	1.66E-06	3.80E-06	6.05E-06	1.54E-05	3.88E-05
**QTMQML**	4.98E-06	8.67E-06	5.73E-06	1.38E-05	2.73E-06	2.71E-06	3.72E-06	1.95E-05	8.16E-05
**NEGIVPTAML**	1.09E-05	1.58E-04	2.44E-03	3.13E-04	7.86E-04	5.03E-04	2.07E-03	1.45E-03	5.15E-03
**LVANDQMALGAM**	2.43E-05	1.49E-04	nan	3.93E-04	2.58E-04	5.24E-04	3.20E-04	3.05E-04	2.91E-04
**VANDQMALGAMR**	7.36E-07	4.82E-05	1.05E-04	1.06E-05	7.81E-05	5.67E-05	1.72E-04	1.90E-04	1.36E-03
**AITESGLRVGAD**	1.32E-06	4.37E-04	5.86E-04	1.63E-04	7.45E-04	2.03E-04	3.80E-04	1.05E-03	2.72E-03
**ITESGLRVGAD**	8.34E-06	1.91E-04	4.21E-04	1.27E-04	2.34E-04	1.27E-04	2.15E-04	2.43E-04	4.57E-04
**ADISVVGY**	1.18E-06	2.45E-05	7.90E-06	4.99E-06	4.34E-06	1.68E-04	1.93E-05	1.19E-05	5.53E-04
**VVGYDD**	2.86E-06	3.75E-05	7.83E-05	3.97E-05	7.54E-05	1.27E-04	3.41E-04	2.23E-04	1.22E-03
**DDTEDSSCYIPPLTTIK**	1.93E-06	1.14E-04	7.17E-04	7.02E-05	3.68E-04	4.92E-04	9.25E-04	9.60E-04	nan
**TEDSSCYIPPLTTIK**	1.20E-06	3.77E-06	4.17E-04	1.79E-04	3.46E-05	3.61E-04	1.84E-04	2.65E-04	nan
**SSCYIPPLTTIKQD**	4.09E-07	6.50E-05	4.51E-04	6.44E-05	7.18E-05	3.18E-04	4.40E-04	2.98E-04	7.57E-03
**FRLL**	6.77E-07	1.48E-06	7.23E-05	2.37E-05	2.73E-06	7.48E-05	1.48E-04	6.96E-05	1.76E-03
**FRLLGQTSV**	2.20E-05	1.59E-05	nan	7.51E-05	1.96E-05	4.31E-05	3.26E-05	4.21E-05	3.26E-05
**LGQTSVDR**	3.64E-08	2.82E-05	5.30E-05	2.42E-05	3.29E-05	1.71E-05	6.02E-05	1.55E-04	7.04E-04
**GQTSVDRL**	5.68E-07	1.48E-05	2.30E-05	1.20E-05	1.89E-05	3.53E-05	1.45E-05	6.34E-05	nan
**LLQL**	9.09E-07	3.52E-05	4.83E-04	1.59E-04	3.33E-04	5.29E-04	9.07E-04	7.65E-04	4.71E-03

ONPF vs DNA

Peptide	0 s	30 s	45 s	60 s	300 s	1500 s	3600 s	7200 s	14400 s
LIGVA	1.48E-06	1.63E-05	3.89E-05	2.37E-05	4.18E-05	1.53E-05	1.82E-07	5.55E-06	4.28E-05
ALHAP	2.02E-06	9.55E-05	5.57E-04	9.96E-05	3.71E-04	2.93E-04	2.89E-04	6.29E-04	nan
ALHAPSQIVA	6.61E-06	2.92E-05	nan	1.06E-04	4.94E-05	3.19E-05	2.25E-04	6.70E-05	5.81E-05
ALHAPSQIVAA	3.24E-06	6.18E-05	nan	2.21E-04	8.11E-05	1.15E-04	2.51E-04	3.10E-04	4.32E-04
ALHAPSQIVAAIK	nan	nan	nan	nan	nan	nan	nan	nan	nan
SQIVAA	5.72E-05	nan	nan	5.95E-05	nan	nan	7.17E-04	3.59E-04	nan
SQIVAAIK	3.68E-06	1.78E-05	nan	2.08E-06	7.33E-05	4.29E-05	1.19E-03	2.76E-04	nan
IVAAIK	3.35E-06	5.56E-06	2.28E-04	8.77E-07	1.89E-05	1.56E-05	2.54E-04	5.33E-05	1.80E-03
AIKSRADQLGAS	1.26E-06	9.27E-05	4.71E-04	1.58E-04	1.59E-04	1.88E-04	2.89E-04	3.19E-04	1.25E-03
ADQLGAS	2.55E-04	nan	6.59E-04	3.31E-04	nan	1.04E-03	8.07E-04	nan	nan
VVVSM	1.10E-05	1.46E-04	1.62E-03	2.67E-04	9.31E-04	5.25E-04	4.89E-04	8.56E-04	2.18E-03
MVERSGVE	4.93E-06	3.36E-04	nan	4.95E-04	9.76E-04	5.34E-04	7.90E-04	7.65E-04	3.82E-04
AAVHNL	1.08E-05	3.34E-05	2.07E-04	6.68E-06	2.05E-04	1.44E-04	4.69E-04	2.19E-04	nan
LAQRVSGL	3.86E-07	2.17E-05	6.56E-04	7.26E-06	1.52E-04	1.16E-04	3.63E-04	1.29E-04	4.75E-03
VSGLIIN	3.28E-05	6.18E-06	1.64E-05	1.61E-05	7.14E-07	2.02E-06	2.91E-05	6.93E-05	8.74E-06
IINYPLD	8.57E-06	7.39E-06	1.40E-05	5.48E-06	3.40E-05	1.49E-05	2.00E-05	5.69E-05	8.77E-04
LDDQDAIA	2.05E-07	3.65E-04	1.63E-03	5.27E-04	8.31E-04	5.31E-04	1.34E-03	1.15E-03	nan
IAVEAA	1.39E-06	nan	9.41E-04	1.78E-06	7.73E-05	1.60E-04	3.56E-04	6.87E-05	4.08E-03
ACTNVPAL	1.77E-05	4.28E-05	1.42E-03	1.85E-05	8.17E-04	5.74E-04	1.23E-03	1.30E-03	nan
ALFLD	1.18E-06	6.07E-05	1.73E-04	1.94E-05	4.85E-05	3.72E-05	6.21E-05	2.05E-04	nan
FLDVSDQTPINS	7.02E-06	9.79E-05	nan	3.29E-04	2.27E-04	4.28E-04	5.24E-04	8.81E-04	1.47E-03
DQTPIN	2.54E-05	2.08E-05	7.36E-04	9.68E-06	5.82E-04	6.13E-04	5.96E-04	5.56E-04	nan
SIIFSH	nan	nan	nan	nan	nan	nan	nan	nan	nan
SIIFSHEDGTRL	1.03E-06	1.79E-05	nan	2.47E-05	1.94E-05	2.11E-05	4.92E-05	6.40E-05	1.93E-04
EDGTRLGVEH	7.79E-06	2.14E-05	5.35E-05	3.60E-05	3.08E-05	3.25E-05	2.42E-04	8.73E-05	nan
LVAL	5.55E-07	1.82E-06	2.02E-05	2.36E-07	1.39E-06	2.27E-08	8.67E-06	3.84E-06	9.17E-05
LVALGHQQIAL	4.02E-06	8.22E-05	3.91E-04	3.59E-05	1.55E-04	8.91E-05	3.54E-04	5.60E-04	1.97E-03
LGHQQIALL	1.03E-05	1.90E-05	2.27E-05	1.32E-05	3.96E-05	4.32E-05	5.66E-05	6.54E-05	4.37E-05
IALL	4.24E-06	5.04E-06	nan	8.23E-06	4.46E-06	1.04E-05	5.04E-06	5.30E-06	nan

**LAGPLS**	2.54E-05	1.82E-05	2.77E-04	3.60E-06	1.82E-04	nan	1.62E-03	9.28E-04	nan
**LAGPLSSVSARL**	3.18E-04	4.25E-04	9.85E-04	1.10E-04	4.30E-04	7.41E-04	8.63E-04	8.34E-04	3.60E-03
**GPLSSVSAR**	2.84E-06	1.53E-04	1.68E-03	4.11E-05	2.04E-04	1.03E-03	5.54E-04	1.82E-03	4.04E-03
**LAGWHK**	1.10E-04	4.36E-05	8.24E-05	1.43E-06	5.06E-05	1.95E-05	1.54E-05	3.24E-05	0 s.00E+00
**YLTRNQIQP**	1.62E-06	3.30E-06	1.46E-03	1.55E-06	4.02E-04	4.50E-05	4.43E-04	3.47E-04	nan
**NQIQPIAEREGD**	3.30E-06	4.58E-05	8.14E-04	9.28E-06	1.05E-03	3.57E-04	7.58E-04	7.89E-04	nan
**IAEREGDWSAMSG**	3.97E-06	2.38E-04	1.68E-03	2.75E-05	9.44E-04	8.64E-04	1.56E-03	1.05E-03	1.16E-02
**REGDWSAMSGF**	5.31E-06	1.66E-05	nan	1.83E-04	1.64E-04	5.03E-04	6.55E-04	3.84E-04	9.94E-04
**DWSAM**	1.19E-05	3.35E-05	5.87E-04	8.23E-05	7.34E-04	6.21E-04	9.00E-04	5.75E-04	nan
**WSAM**	1.50E-04	1.08E-03	1.44E-03	1.19E-05	1.04E-03	nan	1.41E-03	nan	nan
**SAMSGFQ**	1.24E-05	3.08E-04	nan	nan	1.86E-03	2.34E-04	2.14E-04	3.28E-04	nan
**SGFQQTMQM**	4.35E-06	9.86E-06	1.50E-05	1.58E-05	1.27E-05	1.01E-05	1.30E-05	2.29E-05	6.52E-05
**QTMQML**	1.55E-05	8.57E-06	nan	7.68E-06	3.36E-07	1.78E-06	2.08E-06	2.02E-06	1.48E-05
**NEGIVPTAML**	9.91E-06	1.48E-04	5.70E-03	8.57E-06	7.94E-04	5.01E-04	3.76E-03	1.12E-03	6.57E-03
**LVANDQMALGAM**	1.76E-06	8.53E-05	nan	3.56E-04	1.62E-04	4.08E-04	2.02E-04	2.03E-04	1.79E-04
**VANDQMALGAMR**	4.09E-07	4.66E-05	3.13E-04	1.74E-05	1.24E-04	7.18E-05	4.23E-04	2.11E-04	1.17E-03
**AITESGLRVGAD**	6.23E-06	1.31E-04	8.88E-04	6.46E-05	3.61E-04	2.16E-04	5.37E-04	9.80E-04	3.20E-03
**ITESGLRVGAD**	5.90E-06	1.59E-04	3.80E-04	1.44E-04	2.88E-04	1.99E-04	3.10E-04	2.96E-04	7.49E-04
**ADISVVGY**	6.15E-06	9.96E-05	1.14E-04	1.64E-04	7.78E-05	2.03E-04	7.62E-05	1.92E-05	2.09E-04
**VVGYDD**	2.46E-06	9.04E-05	8.30E-05	9.14E-05	1.38E-04	1.33E-04	4.18E-04	2.07E-04	1.14E-03
**DDTEDSSCYIPPLTTIK**	1.80E-06	5.95E-05	6.44E-04	5.22E-05	2.67E-04	3.24E-04	8.54E-04	6.26E-04	nan
**TEDSSCYIPPLTTIK**	2.64E-07	1.79E-05	4.37E-04	5.10E-05	5.82E-05	7.92E-05	2.28E-04	6.63E-05	nan
**SSCYIPPLTTIKQD**	2.01E-06	5.13E-05	4.78E-04	4.36E-05	7.24E-05	9.67E-05	5.69E-04	2.54E-04	7.54E-03
**FRLL**	4.95E-07	3.29E-06	1.27E-04	5.13E-06	5.93E-06	6.26E-06	1.96E-04	6.37E-05	1.98E-03
**FRLLGQTSV**	1.74E-06	5.61E-06	nan	4.87E-05	1.32E-05	8.77E-06	6.05E-05	2.65E-05	1.15E-04
**LGQTSVDR**	1.32E-07	2.82E-05	3.86E-05	3.76E-05	3.29E-05	2.14E-05	6.70E-05	1.30E-04	5.84E-04
**GQTSVDRL**	4.55E-07	9.94E-06	1.84E-05	9.45E-06	2.72E-05	1.46E-05	4.71E-05	3.62E-05	nan
**LLQL**	4.02E-06	3.54E-05	1.22E-03	3.46E-05	2.83E-04	4.75E-04	1.05E-03	5.49E-04	6.99E-03

ONPF vs ONPFDNA

Peptide	0 s	30 s	45 s	60 s	300 s	1500 s	3600 s	7200 s	14400 s
LIGVA	2.64E-06	2.60E-05	2.62E-05	2.10E-05	3.12E-05	1.89E-05	4.73E-07	5.82E-06	6.07E-05
ALHAP	8.41E-07	7.91E-05	3.18E-04	8.06E-05	3.80E-04	2.91E-04	1.22E-04	2.34E-04	1.90E-03
ALHAPSQIVA	8.86E-06	1.28E-04	nan	1.54E-04	5.41E-05	2.29E-04	2.51E-04	1.10E-04	3.45E-04
ALHAPSQIVAA	5.45E-07	1.17E-04	nan	2.31E-04	6.33E-05	2.15E-04	2.83E-04	2.63E-04	2.48E-04
ALHAPSQIVAAIK	nan	nan	nan	nan	nan	nan	nan	nan	nan
SQIVAA	7.92E-05	nan	nan	4.50E-05	nan	nan	7.25E-04	3.00E-04	nan
SQIVAAIK	1.86E-07	8.33E-06	nan	2.27E-06	5.17E-05	4.69E-05	1.27E-03	3.39E-04	nan
IVAAIK	1.20E-06	2.24E-06	1.83E-04	1.55E-06	1.50E-05	1.63E-05	2.44E-04	8.65E-05	1.63E-03
AIKSRADQLGAS	1.18E-06	1.22E-04	2.58E-04	1.49E-05	7.82E-05	3.28E-04	1.98E-04	1.97E-04	6.27E-04
ADQLGAS	2.73E-04	nan	4.15E-06	7.38E-05	nan	9.21E-04	7.37E-04	nan	nan
VVVSM	1.09E-05	1.70E-04	1.32E-03	2.05E-04	9.89E-04	5.92E-04	3.43E-04	4.30E-04	1.90E-03
MVERSGVE	5.35E-06	8.84E-04	nan	3.53E-04	4.64E-04	4.05E-04	3.05E-04	2.60E-04	4.67E-04
AAVHNL	3.72E-05	1.48E-05	5.84E-05	2.85E-05	1.70E-04	1.46E-04	1.35E-04	nan	nan
LAQRVSGL	5.32E-07	1.13E-05	5.34E-04	5.42E-06	1.49E-04	1.27E-04	3.22E-04	7.66E-05	4.11E-03
VSGLIIN	4.14E-05	1.16E-05	1.28E-05	9.25E-06	1.05E-04	2.79E-06	9.91E-07	3.55E-05	7.44E-06
IINYPLD	6.39E-06	1.98E-06	7.77E-06	1.73E-05	1.76E-05	3.45E-05	1.44E-05	1.08E-05	3.16E-04
LDDQDAIA	8.18E-07	3.18E-04	1.30E-03	1.57E-04	7.76E-04	5.47E-04	7.55E-04	4.38E-04	nan
IAVEAA	1.33E-06	4.22E-05	1.06E-04	1.27E-05	4.57E-05	2.19E-04	3.40E-04	5.82E-05	3.99E-03
ACTNVPAL	8.77E-07	8.91E-05	1.08E-03	3.39E-05	7.90E-04	5.80E-04	7.47E-04	1.99E-04	nan
ALFLD	2.64E-06	4.79E-05	nan	6.47E-05	4.37E-04	2.82E-05	1.08E-04	2.96E-04	nan
FLDVSDQTPINS	7.38E-06	2.09E-04	nan	1.60E-04	2.14E-04	2.90E-04	5.57E-04	5.52E-04	1.42E-03
DQTPIN	2.70E-05	3.97E-05	3.34E-04	1.38E-05	6.82E-04	6.24E-04	3.15E-04	2.37E-04	nan
SIIFSH	nan	nan	nan	nan	nan	nan	nan	nan	nan
SIIFSHEDGTRL	9.40E-07	1.68E-05	nan	1.68E-05	3.35E-05	1.64E-05	5.33E-05	7.86E-05	1.22E-04
EDGTRLGVEH	3.73E-06	3.00E-05	5.13E-05	6.95E-06	2.83E-05	4.53E-05	2.79E-04	8.30E-05	nan
LVAL	5.91E-07	2.91E-07	5.50E-06	2.77E-07	2.95E-07	3.33E-06	3.73E-06	1.14E-06	3.82E-06
LVALGHQQIAL	4.95E-07	7.78E-05	3.36E-04	2.61E-05	1.67E-04	7.68E-05	2.14E-04	4.76E-04	1.02E-03
LGHQQIALL	1.11E-05	4.01E-05	7.85E-05	5.56E-05	3.55E-05	2.67E-05	4.60E-05	4.71E-05	1.55E-05
IALL	2.64E-06	3.85E-06	1.68E-06	8.57E-06	1.02E-06	2.48E-06	3.68E-06	5.62E-06	nan

**LAGPLS**	2.22E-05	6.41E-05	1.08E-04	8.45E-06	1.75E-04	7.66E-04	1.29E-03	nan	nan
**LAGPLSSVSARL**	1.60E-05	1.92E-04	7.81E-04	8.12E-05	5.25E-04	5.95E-04	7.28E-04	9.95E-04	3.14E-03
**GPLSSVSAR**	1.89E-06	1.96E-04	1.89E-03	3.76E-05	2.02E-04	1.50E-05	4.00E-04	1.29E-03	1.28E-03
**LAGWHK**	2.50E-05	2.68E-05	6.66E-06	5.69E-06	2.77E-05	8.35E-06	nan	1.59E-05	8.73E-05
**YLTRNQIQP**	1.18E-06	4.85E-05	1.24E-03	4.97E-05	3.95E-04	1.67E-04	1.85E-04	5.02E-06	nan
**NQIQPIAEREGD**	2.95E-07	3.47E-05	7.37E-04	1.68E-06	1.07E-03	4.52E-04	4.12E-04	5.50E-04	nan
**IAEREGDWSAMSG**	4.08E-06	2.88E-04	1.28E-03	2.85E-05	1.11E-03	9.35E-04	7.53E-04	3.42E-04	7.43E-03
**REGDWSAMSGF**	2.57E-05	8.16E-05	nan	2.19E-04	1.50E-04	1.22E-04	5.63E-04	1.04E-04	1.94E-04
**DWSAM**	7.37E-06	2.69E-05	2.49E-04	3.68E-05	8.16E-04	8.08E-04	5.94E-04	2.08E-04	nan
**WSAM**	8.40E-05	1.59E-04	7.55E-04	9.04E-05	9.66E-04	8.35E-04	5.64E-04	nan	nan
**SAMSGFQ**	6.58E-05	2.78E-04	nan	2.81E-04	2.13E-03	2.56E-04	2.46E-05	2.47E-04	nan
**SGFQQTMQM**	6.89E-06	2.59E-06	3.01E-05	nan	3.09E-06	1.25E-05	6.99E-06	2.59E-05	1.66E-05
**QTMQML**	1.17E-05	8.47E-06	6.10E-06	5.72E-06	4.82E-07	1.05E-05	1.97E-06	2.37E-06	2.49E-05
**NEGIVPTAML**	3.30E-05	2.51E-04	2.94E-03	3.60E-06	7.36E-04	7.27E-04	2.04E-03	2.67E-04	1.06E-03
**LVANDQMALGAM**	1.20E-04	1.90E-03	nan	3.95E-03	2.96E-03	4.89E-03	3.97E-03	2.17E-03	5.08E-03
**VANDQMALGAMR**	4.82E-07	3.24E-05	1.55E-04	2.47E-05	8.74E-05	5.70E-05	3.30E-04	2.31E-04	6.00E-04
**AITESGLRVGAD**	2.27E-08	1.08E-04	6.61E-04	2.40E-05	3.68E-04	1.87E-04	2.79E-04	5.38E-04	1.84E-03
**ITESGLRVGAD**	9.46E-06	2.09E-04	3.06E-04	9.12E-05	1.45E-04	3.95E-05	1.59E-04	1.07E-04	4.21E-04
**ADISVVGY**	1.55E-06	5.60E-05	7.13E-05	9.60E-06	1.56E-05	1.85E-04	4.69E-05	4.05E-05	1.68E-04
**VVGYDD**	6.77E-07	5.76E-05	1.01E-04	5.08E-05	7.44E-05	1.10E-04	3.36E-04	2.15E-04	9.46E-04
**DDTEDSSCYIPPLTTIK**	1.86E-06	5.21E-05	7.24E-04	6.21E-05	3.24E-04	3.21E-04	8.25E-04	5.15E-04	nan
**TEDSSCYIPPLTTIK**	2.64E-07	8.10E-06	4.26E-04	5.05E-05	8.57E-05	8.02E-05	2.23E-04	2.72E-05	nan
**SSCYIPPLTTIKQD**	5.91E-07	6.88E-05	4.41E-04	4.52E-05	6.30E-05	9.92E-05	4.30E-04	2.16E-04	7.39E-03
**FRLL**	9.09E-07	2.50E-06	7.03E-05	3.39E-06	2.64E-06	1.87E-05	1.77E-04	4.98E-05	1.61E-03
**FRLLGQTSV**	5.61E-05	1.31E-05	nan	4.88E-05	5.22E-06	5.23E-05	2.43E-05	2.00E-05	4.36E-05
**LGQTSVDR**	1.82E-08	1.65E-05	3.46E-05	1.05E-05	1.69E-05	1.19E-05	4.56E-05	1.33E-04	5.07E-04
**GQTSVDRL**	6.18E-07	8.19E-06	1.01E-05	7.39E-06	9.66E-06	9.11E-06	1.78E-05	2.96E-05	nan
**LLQL**	1.93E-06	3.99E-05	5.79E-04	3.90E-05	3.29E-04	4.75E-04	1.11E-03	5.26E-04	4.98E-03

ONPF vs TMG

Peptide	0 s	30 s	45 s	60 s	300 s	1500 s	3600 s	7200 s	14400 s
LIGVA	1.93E-06	3.24E-06	2.66E-05	1.28E-05	1.76E-05	1.43E-05	1.38E-05	5.97E-06	3.97E-05
ALHAP	9.18E-07	1.10E-04	3.22E-04	2.59E-04	3.17E-04	4.29E-04	3.45E-04	4.51E-04	2.09E-03
ALHAPSQIVA	7.11E-06	4.28E-05	nan	7.14E-04	1.45E-05	2.26E-04	1.59E-04	3.89E-04	1.81E-04
ALHAPSQIVAA	1.37E-06	5.85E-05	nan	7.56E-04	2.60E-05	2.51E-04	9.92E-05	4.90E-04	1.71E-04
ALHAPSQIVAAIK	nan	nan	nan	nan	nan	nan	nan	nan	nan
SQIVAA	6.83E-05	nan	nan	1.21E-04	nan	nan	8.07E-04	3.16E-04	nan
SQIVAAIK	1.28E-06	8.64E-06	nan	8.92E-05	5.14E-05	7.04E-05	1.12E-03	2.19E-04	nan
IVAAIK	1.86E-06	2.95E-06	1.83E-04	4.34E-05	1.57E-05	3.01E-05	2.38E-04	5.56E-05	1.63E-03
AIKSRADQLGAS	1.88E-06	2.49E-04	2.43E-04	2.27E-05	5.81E-05	2.70E-04	3.87E-04	1.09E-03	6.36E-04
ADQLGAS	2.56E-04	nan	2.98E-05	3.17E-04	nan	1.52E-03	2.02E-03	nan	nan
VVVSM	7.16E-05	3.70E-04	1.15E-03	1.26E-04	7.87E-04	4.82E-04	3.17E-04	7.22E-04	1.63E-03
MVERSGVE	1.54E-05	2.41E-03	nan	1.93E-04	4.70E-04	1.00E-03	9.23E-04	1.01E-03	6.54E-04
AAVHNL	9.45E-06	1.51E-05	3.63E-04	4.14E-05	3.65E-05	1.85E-04	2.39E-04	2.38E-04	nan
LAQRVSGL	5.91E-07	1.74E-04	6.02E-04	4.89E-05	1.68E-04	1.65E-04	3.57E-04	4.02E-04	4.27E-03
VSGLIIN	2.48E-05	4.59E-06	3.37E-05	1.16E-05	1.64E-06	1.42E-05	2.59E-06	1.22E-05	1.20E-05
IINYPLD	2.19E-05	1.27E-05	3.67E-06	nan	1.48E-05	3.53E-05	3.15E-06	1.80E-05	3.07E-04
LDDQDAIA	6.39E-06	9.93E-04	1.20E-03	1.52E-04	6.36E-04	6.46E-04	7.54E-04	6.61E-04	nan
IAVEAA	1.17E-06	4.74E-05	1.54E-04	9.32E-07	4.92E-05	5.97E-05	3.30E-04	1.62E-04	3.98E-03
ACTNVPAL	2.44E-05	1.65E-03	1.08E-03	1.20E-04	9.28E-04	1.28E-03	1.12E-03	1.91E-03	nan
ALFLD	1.12E-05	2.67E-05	1.30E-04	2.05E-05	1.23E-05	6.55E-06	2.60E-05	2.55E-04	nan
FLDVSDQTPINS	1.23E-05	2.75E-04	7.62E-04	7.99E-04	1.28E-04	6.00E-04	5.53E-04	9.98E-04	1.37E-03
DQTPIN	2.26E-05	6.11E-05	4.45E-04	4.25E-04	5.56E-04	1.05E-03	4.94E-04	8.32E-04	nan
SIIFSH	nan	nan	nan	nan	nan	nan	nan	nan	nan
SIIFSHEDGTRL	1.09E-06	2.00E-04	nan	2.51E-04	3.68E-05	4.89E-05	2.04E-05	6.85E-05	1.38E-04
EDGTRLGVEH	1.98E-05	7.57E-05	3.63E-04	nan	1.27E-04	4.91E-04	1.29E-04	1.11E-04	nan
LVAL	6.23E-07	1.14E-07	1.15E-05	1.44E-04	2.27E-07	1.75E-05	5.60E-06	9.91E-07	2.91E-07
LVALGHQQIAL	4.55E-06	3.16E-04	2.86E-04	3.53E-04	1.24E-04	2.91E-04	1.60E-04	3.39E-04	1.09E-03
LGHQQIALL	1.71E-05	3.75E-05	2.40E-06	1.05E-05	1.62E-05	7.28E-05	4.73E-05	5.61E-05	2.81E-05
IALL	2.80E-06	9.79E-06	2.05E-06	6.97E-05	5.91E-07	1.82E-05	4.33E-06	8.57E-06	nan

**LAGPLS**	4.27E-05	4.58E-04	1.12E-04	4.23E-04	1.94E-04	9.24E-04	1.28E-03	9.46E-04	nan
**LAGPLSSVSARL**	3.99E-05	1.52E-04	6.37E-04	3.00E-04	3.59E-04	1.01E-03	6.79E-04	nan	nan
**GPLSSVSAR**	5.38E-06	5.39E-04	1.75E-03	nan	2.11E-04	4.13E-04	6.92E-04	1.19E-03	nan
**LAGWHK**	3.83E-05	1.68E-04	2.48E-04	nan	2.98E-07	5.10E-04	1.58E-05	2.84E-04	3.64E-04
**YLTRNQIQP**	2.48E-06	4.18E-04	nan	9.31E-05	3.50E-04	nan	4.35E-04	nan	7.48E-04
**NQIQPIAEREGD**	7.73E-07	9.32E-04	7.35E-04	3.55E-04	nan	8.21E-04	nan	7.30E-04	nan
**IAEREGDWSAMSG**	4.08E-06	7.77E-04	1.12E-03	7.21E-04	1.01E-03	1.79E-03	1.13E-03	1.28E-03	7.91E-03
**REGDWSAMSGF**	5.64E-06	3.69E-04	nan	4.04E-04	nan	2.89E-04	nan	8.15E-04	3.76E-04
**DWSAM**	2.02E-05	1.66E-04	3.24E-04	7.28E-04	7.40E-04	1.02E-03	8.20E-04	9.87E-04	nan
**WSAM**	7.22E-05	1.14E-04	8.00E-04	2.55E-04	7.96E-04	9.75E-04	7.99E-04	nan	5.15E-04
**SAMSGFQ**	1.94E-05	6.50E-04	nan	5.45E-04	1.88E-03	3.68E-04	9.50E-05	nan	nan
**SGFQQTMQM**	6.60E-06	3.75E-06	3.53E-05	nan	3.96E-06	1.48E-05	8.52E-06	1.63E-05	8.20E-06
**QTMQML**	5.91E-06	1.94E-05	1.83E-05	nan	5.68E-07	6.46E-06	3.13E-06	7.57E-06	1.31E-06
**NEGIVPTAML**	2.05E-05	3.91E-04	3.62E-03	1.13E-05	5.41E-04	7.35E-04	1.60E-03	1.28E-03	6.31E-04
**LVANDQMALGAM**	1.42E-06	1.42E-04	nan	nan	1.37E-04	5.12E-04	1.79E-04	1.50E-04	9.87E-05
**VANDQMALGAMR**	1.14E-06	9.91E-05	1.06E-04	nan	7.81E-05	2.53E-04	1.58E-04	1.10E-04	6.11E-04
**AITESGLRVGAD**	8.41E-06	8.58E-04	5.90E-04	5.25E-04	5.27E-04	8.52E-04	4.47E-04	1.02E-03	2.17E-03
**ITESGLRVGAD**	1.14E-05	1.09E-03	3.03E-04	2.97E-04	2.95E-04	6.42E-04	3.37E-04	8.12E-04	6.76E-04
**ADISVVGY**	1.91E-06	3.78E-05	9.98E-06	nan	3.60E-06	3.40E-04	2.42E-05	8.55E-06	2.03E-04
**VVGYDD**	8.23E-07	3.44E-05	5.91E-05	nan	7.65E-05	1.87E-04	3.05E-04	1.40E-04	9.92E-04
**DDTEDSSCYIPPLTTIK**	1.80E-06	1.91E-04	6.56E-04	nan	2.54E-04	5.81E-04	8.07E-04	1.12E-03	nan
**TEDSSCYIPPLTTIK**	2.66E-06	5.35E-05	5.37E-04	nan	2.85E-05	1.73E-04	1.76E-04	2.80E-04	nan
**SSCYIPPLTTIKQD**	4.82E-07	9.66E-05	5.53E-04	3.34E-03	5.74E-05	3.28E-04	4.37E-04	2.58E-04	7.58E-03
**FRLL**	6.77E-07	3.48E-06	1.17E-04	1.61E-04	5.69E-06	1.72E-05	1.53E-04	1.44E-04	1.66E-03
**FRLLGQTSV**	5.84E-06	5.64E-05	nan	1.48E-04	3.21E-05	5.98E-05	nan	3.69E-05	3.15E-05
**LGQTSVDR**	2.41E-07	2.02E-05	3.72E-05	1.58E-04	1.78E-05	6.39E-05	4.29E-05	1.15E-04	5.27E-04
**GQTSVDRL**	6.18E-07	nan	nan	8.16E-05	nan	6.47E-05	1.54E-05	2.37E-05	nan
**LLQL**	2.46E-06	9.12E-05	7.41E-04	9.31E-05	2.85E-04	5.98E-04	9.73E-04	1.27E-03	4.71E-03

TMG vs ONPFDNA

Peptide	0 s	30 s	45 s	60 s	300 s	1500 s	3600 s	7200 s	14400 s
LIGVA	1.62E-06	2.31E-05	5.69E-06	1.11E-05	1.49E-05	4.67E-06	1.42E-05	2.48E-06	3.15E-05
ALHAP	6.59E-07	4.07E-05	6.15E-06	2.50E-04	6.83E-05	1.47E-04	2.28E-04	3.46E-04	2.56E-04
ALHAPSQIVA	3.06E-06	1.32E-04	nan	8.01E-04	3.98E-05	4.19E-04	1.53E-04	4.24E-04	4.99E-04
ALHAPSQIVAA	9.53E-07	1.32E-04	nan	7.40E-04	6.00E-05	3.13E-04	1.90E-04	4.66E-04	4.06E-04
ALHAPSQIVAAIK	5.39E-06	1.91E-04	1.72E-04	nan	1.99E-04	4.93E-05	1.29E-03	5.19E-04	2.43E-05
SQIVAA	3.75E-05	1.61E-05	3.35E-05	8.94E-05	nan	2.26E-04	9.88E-05	2.02E-05	9.40E-06
SQIVAAIK	1.24E-06	1.08E-05	2.90E-05	9.13E-05	9.45E-07	3.17E-05	1.94E-04	1.22E-04	6.51E-05
IVAAIK	2.98E-06	2.24E-06	9.62E-06	4.37E-05	1.11E-06	1.58E-05	2.59E-05	4.94E-05	9.90E-06
AIKSRADQLGAS	8.36E-07	3.01E-04	1.84E-05	3.30E-05	9.37E-05	3.66E-04	3.31E-04	9.40E-04	6.98E-04
ADQLGAS	4.43E-05	2.22E-04	2.83E-05	3.34E-04	4.26E-04	6.28E-04	1.36E-03	4.63E-03	1.68E-03
VVVSM	6.16E-05	2.56E-04	4.00E-04	1.29E-04	2.02E-04	1.38E-04	4.56E-05	5.93E-04	4.69E-04
MVERSGVE	1.09E-05	3.08E-03	nan	2.41E-04	4.59E-04	1.08E-03	9.41E-04	1.04E-03	9.52E-04
AAVHNL	3.49E-05	2.95E-07	3.05E-04	5.75E-05	2.01E-04	4.27E-05	2.70E-04	nan	9.75E-05
LAQRVSGL	3.86E-07	1.72E-04	1.09E-04	4.38E-05	2.89E-05	6.19E-05	1.33E-04	3.37E-04	1.58E-04
VSGLIIN	2.42E-05	9.04E-06	4.64E-05	3.30E-06	1.06E-04	1.41E-05	2.04E-06	3.78E-05	1.07E-05
IINYPLD	2.17E-05	1.11E-05	7.04E-06	nan	2.84E-06	6.96E-05	1.14E-05	9.57E-06	9.21E-06
LDDQDAIA	6.88E-06	7.01E-04	1.22E-04	1.07E-04	3.24E-04	4.60E-04	1.82E-04	4.01E-04	2.84E-04
IAVEAA	1.68E-07	9.28E-06	1.62E-04	1.19E-05	7.57E-06	1.69E-04	1.63E-05	1.65E-04	3.97E-05
ACTNVPAL	2.49E-05	1.65E-03	1.43E-06	1.18E-04	1.38E-04	7.26E-04	3.87E-04	1.73E-03	6.51E-04
ALFLD	1.15E-05	2.21E-05	nan	7.81E-05	4.43E-04	2.29E-05	1.28E-04	1.43E-04	2.67E-04
FLDVSDQTPINS	5.62E-06	3.38E-04	nan	6.47E-04	9.22E-05	4.44E-04	5.19E-04	5.37E-04	2.42E-04
DQTPIN	8.76E-06	6.59E-05	2.20E-04	4.24E-04	1.31E-04	4.73E-04	1.81E-04	6.49E-04	9.56E-05
SIIFSH	5.61E-04	2.26E-04	7.31E-04	1.04E-03	4.85E-04	5.41E-04	1.03E-03	8.39E-04	nan
SIIFSHEDGTRL	2.97E-07	2.02E-04	nan	2.55E-04	5.79E-05	6.03E-05	3.30E-05	9.56E-05	4.22E-05
EDGTRLGVEH	1.78E-05	8.30E-05	3.17E-04	nan	1.01E-04	4.79E-04	1.54E-04	6.50E-05	4.25E-04
LVAL	1.14E-07	4.05E-07	7.11E-06	1.44E-04	7.73E-08	2.08E-05	8.02E-06	5.91E-07	4.11E-06
LVALGHQQIAL	4.13E-06	2.94E-04	5.87E-05	3.51E-04	5.54E-05	2.14E-04	1.02E-04	4.45E-04	7.34E-05
LGHQQIALL	7.83E-06	5.80E-05	8.10E-05	6.53E-05	2.81E-05	5.23E-05	2.42E-05	4.78E-05	1.48E-05
IALL	3.11E-06	6.51E-06	1.68E-06	7.42E-05	8.77E-07	1.59E-05	8.82E-07	3.68E-06	2.42E-05

**LAGPLS**	2.41E-05	5.20E-04	7.71E-06	4.31E-04	1.94E-05	1.99E-04	1.53E-05	nan	2.94E-04
**LAGPLSSVSARL**	2.59E-05	1.92E-04	1.46E-04	2.49E-04	1.67E-04	6.72E-04	2.43E-04	nan	nan
**GPLSSVSAR**	3.64E-06	4.43E-04	3.09E-04	nan	8.82E-06	4.20E-04	2.95E-04	1.49E-03	nan
**LAGWHK**	2.05E-05	1.94E-04	2.44E-04	nan	2.76E-05	5.12E-04	nan	2.73E-04	4.51E-04
**YLTRNQIQP**	1.33E-06	4.60E-04	nan	1.40E-04	1.31E-04	nan	2.52E-04	nan	nan
**NQIQPIAEREGD**	6.23E-07	9.03E-04	2.17E-06	3.54E-04	nan	5.85E-04	nan	8.28E-04	nan
**IAEREGDWSAMSG**	1.54E-06	7.02E-04	3.51E-04	7.05E-04	4.38E-04	1.00E-03	5.67E-04	9.37E-04	5.61E-04
**REGDWSAMSGF**	2.77E-05	4.25E-04	6.46E-04	3.97E-04	nan	2.69E-04	nan	7.44E-04	4.19E-04
**DWSAM**	1.37E-05	1.49E-04	1.64E-04	6.91E-04	1.87E-04	5.94E-04	3.56E-04	7.79E-04	3.53E-04
**WSAM**	1.18E-05	4.57E-05	2.90E-04	3.46E-04	2.12E-04	2.06E-04	2.50E-04	nan	nan
**SAMSGFQ**	7.80E-05	3.87E-04	8.60E-04	4.80E-04	2.85E-04	1.64E-04	8.80E-05	nan	1.71E-04
**SGFQQTMQM**	9.60E-06	1.17E-06	3.57E-05	nan	5.27E-06	2.23E-05	4.15E-06	1.40E-05	8.41E-06
**QTMQML**	9.46E-06	1.18E-05	1.63E-05	nan	8.23E-07	1.52E-05	2.47E-06	5.94E-06	2.62E-05
**NEGIVPTAML**	4.42E-05	3.70E-04	2.00E-03	8.31E-06	1.95E-04	4.60E-04	5.95E-04	1.04E-03	1.28E-03
**LVANDQMALGAM**	1.19E-04	2.00E-03	nan	nan	2.97E-03	4.84E-03	3.90E-03	2.08E-03	4.99E-03
**VANDQMALGAMR**	8.82E-07	1.04E-04	5.10E-05	nan	4.32E-05	1.98E-04	1.73E-04	1.63E-04	9.68E-05
**AITESGLRVGAD**	8.42E-06	7.64E-04	8.03E-05	5.01E-04	2.57E-04	7.04E-04	2.07E-04	9.76E-04	3.29E-04
**ITESGLRVGAD**	1.05E-05	1.19E-03	7.78E-05	3.76E-04	2.52E-04	6.08E-04	3.21E-04	8.08E-04	3.32E-04
**ADISVVGY**	1.68E-06	5.54E-05	6.95E-05	nan	1.26E-05	1.94E-04	3.26E-05	3.53E-05	3.90E-05
**VVGYDD**	5.91E-07	4.88E-05	4.76E-05	nan	2.85E-05	8.14E-05	3.15E-05	8.33E-05	4.82E-05
**DDTEDSSCYIPPLTTIK**	3.86E-07	1.56E-04	1.82E-04	nan	1.29E-04	2.64E-04	7.47E-05	6.80E-04	9.19E-04
**TEDSSCYIPPLTTIK**	2.84E-06	6.07E-05	1.34E-04	nan	5.89E-05	9.53E-05	6.34E-05	2.71E-04	3.36E-04
**SSCYIPPLTTIKQD**	3.36E-07	9.66E-05	1.87E-04	3.31E-03	2.40E-05	2.39E-04	1.00E-05	9.94E-05	1.92E-04
**FRLL**	6.77E-07	3.03E-06	5.18E-05	1.57E-04	3.09E-06	2.60E-05	4.20E-05	1.22E-04	4.49E-05
**FRLLGQTSV**	5.96E-05	6.28E-05	1.13E-04	1.05E-04	2.84E-05	9.46E-05	nan	1.69E-05	4.49E-05
**LGQTSVDR**	2.23E-07	1.31E-05	9.20E-06	1.57E-04	6.18E-06	5.21E-05	3.00E-06	3.22E-05	2.05E-05
**GQTSVDRL**	3.27E-07	nan	nan	8.18E-05	nan	5.85E-05	4.73E-06	2.68E-05	nan
**LLQL**	3.48E-06	6.07E-05	4.01E-04	9.25E-05	6.40E-05	1.25E-04	3.76E-04	9.28E-04	7.08E-04
