# Supplementary material for: Ligand-specific changes in conformational flexibility mediate long-range allostery in the lac repressor
Source: Nat Commun. 2023 Mar 2;14:1179. doi: 10.1038/s41467-023-36798-1 (PMC9977783; doi:10.1038/s41467-023-36798-1)
Supplement: Supplementary file 5 — Supplementary Data 3 [file 41467_2023_36798_MOESM5_ESM.pdf]

63-67 LIGVA

Charge 1

IPTG

Time	centroid	D
0	472.5075	-0.02
30	472.606	0.15
45	472.5805	0.10
60	472.566	0.08
300	472.549	0.05
1500	472.5895	0.12
3600	472.587	0.11
7200	472.6015	0.14
14400	472.6275	0.18

ONPF

Time	centroid	D
0	472.508	-0.02
30	472.537	0.03
45	472.561	0.07
60	472.582	0.11
300	472.5505	0.05
1500	472.58	0.10
3600	472.574	0.09
7200	472.578	0.10
14400	472.58	0.10

APO

Time	centroid	D
0	472.5175	0.00
30	472.5625	0.07
45	472.576	0.10
60	472.5655	0.08
300	472.5635	0.07
1500	472.579	0.10
3600	472.5965	0.13
7200	472.584	0.11
14400	472.6515	0.22

DNA

Time	centroid	D
0	472.5095	-0.02
30	472.55	0.05
45	472.545	0.04
60	472.547	0.05
300	472.543	0.04
1500	472.5265	0.01
3600	472.561	0.07
7200	472.567	0.08
14400	472.5585	0.07

ONPFDNA

Time	centroid	D
0	472.513	-0.01
30	472.5485	0.05
45	472.547	0.05
60	472.564	0.07
300	472.553	0.06
1500	472.562	0.07
3600	472.562	0.07
7200	472.573	0.09
14400	472.658	0.24

TMG

Time	centroid	D
0	472.532	0.02
30	472.589	0.12
45	472.595	0.13
60	472.628	0.18
300	472.595	0.13
1500	472.598	0.13
3600	472.6225	0.17
7200	472.5925	0.12
14400	472.616	0.16

control 472.52
infinity 474.281

$$D(t) = \frac{M_t - M_0}{M_\infty - M_0} * N$$

IPTG

Time	Replicate 1	Replicate 2	Replicate 3	Replicate 4	Replicate5	average	SD
0	472.525	472.49				472.51	0.02
30	472.618	472.594				472.61	0.02
45	472.641	472.52				472.58	0.09
60	472.604	472.528				472.57	0.05
300	472.547	472.551				472.55	0.00
1500	472.603	472.576				472.59	0.02
3600	472.592	472.582				472.59	0.01
7200	472.613	472.59				472.60	0.02
14400	472.657	472.598				472.63	0.04

ONPF

Time	Replicate 1	Replicate 2	Replicate 3	Replicate 4	average	SD
0	472.517	472.499			472.51	0.01
30	472.55	472.524			472.54	0.02
45	472.597	472.525			472.56	0.05
60	472.607	472.557			472.58	0.04
300	472.581	472.52			472.55	0.04
1500	472.608	472.552			472.58	0.04
3600	472.575	472.573			472.57	0.00
7200	472.562	472.594			472.58	0.02
14400	472.621	472.534			472.58	0.06

APO

Time	Replicate 1	Replicate 2	Replicate 3	Replicate 4	Replicate 5	average	SD
0	472.531	472.504				472.52	0.02
30	472.598	472.527				472.56	0.05
45	472.587	472.565				472.58	0.02
60	472.603	472.528				472.57	0.05
300	472.606	472.521				472.56	0.06
1500	472.568	472.59				472.58	0.02
3600	472.583	472.61				472.60	0.02
7200	472.563	472.605				472.58	0.03
14400	472.604	472.699				472.65	0.07

DNA

Time	Replicate 1	Replicate 2	Replicate 3	Replicate 4	Replicate 5	average	SD
0	472.509	472.51				472.51	0.00
30	472.577	472.523				472.55	0.04
45	472.574	472.516				472.55	0.04
60	472.573	472.521				472.55	0.04
300	472.58	472.506				472.54	0.05
1500	472.534	472.519				472.53	0.01
3600	472.558	472.564				472.56	0.00
7200	472.574	472.56				472.57	0.01
14400	472.58	472.537				472.56	0.03

ONPFDNA

Time	Replicate 1	Replicate 2	Replicate 3	Replicate 4	average	SD
0	472.521	472.505			472.51	0.01
30	472.584	472.513			472.55	0.05
45	472.559	472.535			472.55	0.02
60	472.587	472.541			472.56	0.03
300	472.581	472.525			472.55	0.04
1500	472.578	472.546			472.56	0.02
3600	472.567	472.557			472.56	0.01
7200	472.581	472.565			472.57	0.01
14400	472.696	472.62			472.66	0.05

TMG

Time	Replicate 1	Replicate 2	Replicate 3	Replicate 4	Replicate 5	average	SD
0	472.537	472.527				472.53	0.01
30	472.592	472.586				472.59	0.00
45	472.608	472.582				472.60	0.02
60	472.637	472.619				472.63	0.01
300	472.601	472.589				472.60	0.01
1500	472.599	472.597				472.60	0.00
3600	472.65	472.595				472.62	0.04
7200	472.601	472.584				472.59	0.01
14400	472.633	472.599				472.62	0.02

72-76 ALHAP

Charge 1

IPTG

Time	centroid	D
0	508.5555	0.00
30	508.7	0.28
45	508.7035	0.29
60	508.7475	0.38
300	508.893	0.66
1500	509.069	1.01
3600	509.31	1.48
7200	509.3935	1.65
14400	509.326	1.52

ONPF

Time	centroid	D
0	508.5615	0.01
30	508.938	0.75
45	509.098	1.07
60	509.1705	1.21
300	509.1495	1.17
1500	509.1875	1.24
3600	509.186	1.24
7200	509.1815	1.23
14400	509.03	0.93

APO

Time	centroid	D
0	508.5555	0.00
30	509.253	1.37
45	509.159	1.19
60	509.3405	1.54
300	509.221	1.31
1500	509.321	1.51
3600	509.3455	1.55
7200	509.2555	1.38
14400	509.3365	1.54

DNA

Time	centroid	D
0	508.563	0.01
30	509.193	1.25
45	509.274	1.41
60	509.195	1.26
300	509.24	1.35
1500	509.279	1.42
3600	509.2245	1.32
7200	509.202	1.27
14400	509.272	1.41

ONPFDNA

Time	centroid	D
0	508.555	0.00
30	509.116	1.10
45	509.247	1.36
60	509.1645	1.20
300	509.215	1.30
1500	509.0545	0.98
3600	509.2575	1.38
7200	509.1975	1.26
14400	508.971	0.82

TMG

Time	centroid	D
0	508.5645	0.02
30	508.7975	0.48
45	508.861	0.60
60	509.0495	0.97
300	509.1405	1.15
1500	509.2715	1.41
3600	509.4275	1.72
7200	509.4125	1.69
14400	509.489	1.84

control 508.5555
infinity 509.572

$$D(t) = \frac{M_t - M_0}{M_\infty - M_0} * N$$

IPTG

Time	Replicate 1	Replicate 2	Replicate 3	Replicate 4	Replicate5	average	SD
0	508.559	508.552				508.56	0.00
30	508.73	508.67				508.70	0.04
45	508.723	508.684				508.70	0.03
60	508.783	508.712				508.75	0.05
300	508.946	508.84				508.89	0.07
1500	509.193	508.945				509.07	0.18
3600	509.383	509.237				509.31	0.10
7200	509.516	509.271				509.39	0.17
14400	509.444	509.208				509.33	0.17

ONPF

Time	Replicate 1	Replicate 2	Replicate 3	Replicate 4	average	SD
0	508.567	508.556			508.56	0.01
30	509.002	508.874			508.94	0.09
45	509.23	508.966			509.10	0.19
60	509.22	509.121			509.17	0.07
300	509.281	509.018			509.15	0.19
1500	509.313	509.062			509.19	0.18
3600	509.267	509.105			509.19	0.11
7200	509.278	509.085			509.18	0.14
14400	509.347	508.706			509.03	0.45

APO

Time	Replicate 1	Replicate 2	Replicate 3	Replicate 4	Replicate 5	average	SD
0	508.554	508.557				508.56	0.00
30	509.363	509.143				509.25	0.16
45	509.22	509.098				509.16	0.09
60	509.506	509.175				509.34	0.23
300	509.441	509.001				509.22	0.31
1500	509.446	509.196				509.32	0.18
3600	509.48	509.211				509.35	0.19
7200	509.403	509.108				509.26	0.21
14400	509.428	509.245				509.34	0.13

DNA

Time	Replicate 1	Replicate 2	Replicate 3	Replicate 4	Replicate 5	average	SD
0	508.554	508.572				508.56	0.01
30	509.227	509.159				509.19	0.05
45	509.159	509.389				509.27	0.16
60	509.25	509.14				509.20	0.08
300	509.296	509.184				509.24	0.08
1500	509.298	509.26				509.28	0.03
3600	509.321	509.128				509.22	0.14
7200	509.361	509.043				509.20	0.22
14400	509.272					509.27	#DIV/0!

ONPFDNA

Time	Replicate 1	Replicate 2	Replicate 3	Replicate 4	average	SD
0	508.559	508.551			508.56	0.01
30	509.1	509.132			509.12	0.02
45	509.254	509.24			509.25	0.01
60	509.209	509.12			509.16	0.06
300	509.275	509.155			509.22	0.08
1500	509.07	509.039			509.05	0.02
3600	509.269	509.246			509.26	0.02
7200	509.257	509.138			509.20	0.08
14400	508.929	509.013			508.97	0.06

TMG

Time	Replicate 1	Replicate 2	Replicate 3	Replicate 4	Replicate 5	average	SD
0	508.569	508.56				508.56	0.01
30	508.753	508.842				508.80	0.06
45	508.878	508.844				508.86	0.02
60	508.941	509.158				509.05	0.15
300	509.128	509.153				509.14	0.02
1500	509.183	509.36				509.27	0.13
3600	509.316	509.539				509.43	0.16
7200	509.288	509.537				509.41	0.18
14400	509.378	509.6				509.49	0.16

72-81 ALHAPSQIVA

Charge 2

IPTG

Time	centroid	D
0	504.08	0.00
30	504.24	0.74
45	504.26	0.83
60	504.31	1.08
300	504.37	1.39
1500	504.47	1.87
3600	504.53	2.16
7200	504.66	2.74
14400	504.69	2.89

ONPF

Time	centroid	D
0	504.07	-0.06
30	504.36	1.34
45	504.76	3.23
60	504.63	2.61
300	504.64	2.67
1500	504.84	3.62
3600	504.98	4.29
7200	505.07	4.74
14400	505.33	5.97

APO

Time	centroid	D
0	504.09	0.05
30	504.47	1.86
45	504.52	2.09
60	504.64	2.69
300	504.72	3.04
1500	505.00	4.39
3600	505.15	5.11
7200	505.33	5.99
14400	505.39	6.27

DNA

Time	centroid	D
0	504.09	0.02
30	504.46	1.79
45		
60	504.53	2.14
300	504.51	2.03
1500	504.59	2.42
3600	504.65	2.73
7200	504.76	3.25
14400	504.85	3.69

ONPFDNA

Time	centroid	D
0	504.07	-0.05
30	504.41	1.59
45	504.55	2.25
60	504.55	2.23
300	504.54	2.21
1500	504.57	2.32
3600	504.68	2.88
7200	504.69	2.90
14400	504.73	3.12

TMG

Time	centroid	D
0	504.09	0.01
30	504.25	0.80
45	504.37	1.38
60	504.55	2.23
300	504.47	1.84
1500	504.58	2.37
3600	504.74	3.16
7200	504.82	3.55
14400	505.00	4.40

control	504.083
infinity	505.543

$$D(t) = \frac{M_t - M_0}{M_\infty - M_0} * N$$

IPTG

Time	Replicate 1	Replicate 2	Replicate 3	Replicate 4	Replicate5	average	SD
0	504.083	504.082	504.084	504.06	504.103	504.08	0.02
30	504.235	504.235	504.229		504.252	504.24	0.01
45					504.256	504.26	
60	504.301	504.319	504.282		504.335	504.31	0.02
300	504.35	504.35	504.313	504.454	504.396	504.37	0.05
1500	504.457	504.457	504.401	504.559	504.489	504.47	0.06
3600	504.511	504.511	504.476	504.595	504.57	504.53	0.05
7200	504.607	504.686	504.595	504.709	504.678	504.66	0.05
14400	504.723	504.723	504.594		504.706	504.69	0.06

ONPF

Time	Replicate 1	Replicate 2	Replicate 3	Replicate 4	average	SD
0	504.085	504.083	504.015	504.099	504.07	0.04
30	504.456	504.32	504.312	504.359	504.36	0.07
45				504.757	504.76	
60	504.644	504.539	504.74	504.589	504.63	0.09
300	504.619		504.596	504.703	504.64	0.06
1500	504.812	504.794		504.91	504.84	0.06
3600	505.122	504.82	504.843	505.123	504.98	0.17
7200	505.07	504.987	505.199	505.034	505.07	0.09
14400	505.339	505.268		505.376	505.33	0.05

APO

Time	Replicate 1	Replicate 2	Replicate 3	Replicate 4	Replicate 5	average	SD
0	504.083	504.083	504.082	504.138	504.078	504.09	0.03
30	504.417	504.417	504.407	504.587	504.523	504.47	0.08
45					504.518	504.52	
60	504.595	504.595	504.58	504.789	504.661	504.64	0.09
300	504.702	504.737	504.635	504.803	504.708	504.72	0.06
1500	504.97	504.97	504.94	505.119	504.998	505.00	0.07
3600	505.093	505.093	504.972	505.242	505.344	505.15	0.15
7200	505.311	505.311	505.112	505.481	505.443	505.33	0.14
14400	505.359	505.354	505.355	505.454	505.429	505.39	0.05

DNA

Time	Replicate 1	Replicate 2	Replicate 3	Replicate 4	Replicate 5	average	SD
0	504.085	504.097	504.085	504.089	504.079	504.09	0.01
30	504.435	504.534	504.401	504.477	504.439	504.46	0.05
45							
60	504.548	504.389	504.478	504.761	504.47	504.53	0.14
300	504.508	504.377	504.463	504.641	504.548	504.51	0.10
1500	504.602	504.553	504.509	504.676	504.602	504.59	0.06
3600	504.686	504.401	504.604	504.737	504.831	504.65	0.16
7200	504.632	504.772	504.725	504.803	504.873	504.76	0.09
14400	504.862	504.717	504.786	504.886	505.01	504.85	0.11

ONPFDNA

Time	Replicate 1	Replicate 2	Replicate 3	Replicate 4	average	SD
0	504.083	504.049	504.084		504.07	0.02
30	504.286	504.553	504.402		504.41	0.13
45			504.552		504.55	
60	504.439	504.707	504.497		504.55	0.14
300	504.473	504.632	504.526		504.54	0.08
1500	504.476	504.782	504.444		504.57	0.19
3600	504.528	504.719	504.806		504.68	0.14
7200	504.586	504.672	504.803		504.69	0.11
14400	504.668	504.993	504.539		504.73	0.23

TMG

Time	Replicate 1	Replicate 2	Replicate 3	Replicate 4	Replicate 5	average	SD
0	504.091	504.079				504.09	0.01
30	504.214	504.285				504.25	0.05
45	504.402	504.339				504.37	0.04
60	504.355	504.742				504.55	0.27
300	504.468	504.465				504.47	0.00
1500	504.47	504.684				504.58	0.15
3600	504.701	504.783				504.74	0.06
7200	504.685	504.963				504.82	0.20
14400	504.904	505.096				505.00	0.14

72-82 ALHAPSQIVAA

Charge 2

IPTG

Time	centroid	D
0	539.6288	0.00
30	539.8333	0.64
45	539.814	0.58
60	539.9164	0.90
300	539.929	0.94
1500	540.04	1.29
3600	540.1108	1.52
7200	540.2174	1.85
14400	540.2574	1.98

ONPF

Time	centroid	D
0	539.63	0.00
30	539.98	1.10
45	540.32	2.16
60	540.27	2.00
300	540.24	1.92
1500	540.50	2.74
3600	540.67	3.28
7200	540.77	3.60
14400	540.94	4.13

APO

Time	centroid	D
0	539.6333	0.01
30	540.0194	1.23
45	539.9805	1.11
60	540.2202	1.86
300	540.2282	1.89
1500	540.5934	3.04
3600	540.713	3.42
7200	540.8326	3.79
14400	540.651	3.22

DNA

Time	centroid	D
0	539.6333	0.01
30	539.974	1.09
45	540.0755	1.41
60	540.105	1.50
300	540.0598	1.36
1500	540.1285	1.57
3600	540.219	1.86
7200	540.27	2.02
14400	540.3135	2.16

ONPFDNA

Time	centroid	D
0	539.6305	0.00
30	539.9907	1.14
45	540.092	1.46
60	540.0995	1.48
300	540.0728	1.40
1500	540.0965	1.47
3600	540.2178	1.85
7200	540.2	1.80
14400	540.2478	1.95

TMG

Time	centroid	D
0	539.62	-0.02
30	539.81	0.56
45	539.93	0.96
60	540.10	1.49
300	540.01	1.21
1500	540.14	1.60
3600	540.27	2.01
7200	540.38	2.37
14400	540.56	2.93

control 539.63
infinity 542.166

$$D(t) = \frac{Mt - M0}{M\infty - M0} *N$$

IPTG

Time	Replicate 1	Replicate 2	Replicate 3	Replicate 4	Replicate5	average	SD
0	539.627	539.643	539.626	539.621	539.627	539.63	0.01
30	539.808		539.892	539.826	539.807	539.83	0.04
45	539.814					539.81	
60	539.874	539.844	540.103	539.89	539.871	539.92	0.11
300	539.956	539.846	540.031	539.934	539.878	539.93	0.07
1500	540.061	539.97	540.145	540.043	539.981	540.04	0.07
3600	540.137	540.072	540.176	540.057	540.112	540.11	0.05
7200	540.232	540.163	540.322	540.226	540.144	540.22	0.07
14400	540.278	540.205	540.333	540.306	540.165	540.26	0.07

ONPF

Time	Replicate 1	Replicate 2	Replicate 3	Replicate 4	average	SD
0	539.643	539.632	539.625	539.619	539.63	0.01
30	539.941	540.036	539.902	540.04	539.98	0.07
45	540.315				540.32	
60	540.203	540.5	540.117	540.241	540.27	0.16
300	540.305	540.204		540.21	540.24	0.06
1500	540.515	540.673	540.411	540.39	540.50	0.13
3600	540.712	540.799	540.461	540.705	540.67	0.15
7200	541.008	540.8	540.614	540.657	540.77	0.18
14400		540.911	540.981	540.921	540.94	0.04

APO

Time	Replicate 1	Replicate 2	Replicate 3	Replicate 4	Replicate 5	average	SD
0	539.595		539.683	539.63	539.625	539.63	0.04
30	540.108	539.896	540.11	540.003	539.98	540.02	0.09
45	540.069	539.892				539.98	0.13
60	540.23	540.149	540.372	540.18	540.17	540.22	0.09
300	540.324	539.99	540.329	540.286	540.212	540.23	0.14
1500	540.672	540.525	540.695	540.548	540.527	540.59	0.08
3600	540.93	540.589	540.816	540.688	540.542	540.71	0.16
7200	541.065	540.425	541.069	540.903	540.701	540.83	0.27
14400	539.625	540.756	540.962	540.951	540.961	540.65	0.58

DNA

Time	Replicate 1	Replicate 2	Replicate 3	Replicate 4	Replicate 5	average	SD
0	539.608	539.667	539.627	539.631		539.63	0.02
30	540.059	539.866	540.046	539.925		539.97	0.09
45	540.211	539.94				540.08	0.19
60	540.057	540.01	540.323	540.03		540.11	0.15
300	540.101	539.944	540.211	539.983		540.06	0.12
1500	540.153	540.052	540.249	540.06		540.13	0.09
3600	540.41	539.998	540.325	540.143		540.22	0.18
7200	540.451	540.012	540.371	540.246		540.27	0.19
14400	540.575	539.875	540.449	540.355		540.31	0.31

ONPFDNA

Time	Replicate 1	Replicate 2	Replicate 3	Replicate 4	average	SD
0	539.627	539.635	539.632	539.628	539.63	0.00
30	539.967		540.146	539.859	539.99	0.14
45	540.092				540.09	
60	540.054	540.002	540.328	540.014	540.10	0.15
300	540.079	539.952	540.204	540.056	540.07	0.10
1500	539.996	539.985	540.355	540.05	540.10	0.17
3600	540.445	540	540.323	540.103	540.22	0.20
7200	540.38	539.995	540.258	540.167	540.20	0.16
14400	540.127	540.047	540.571	540.246	540.25	0.23

TMG

Time	Replicate 1	Replicate 2	Replicate 3	Replicate 4	Replicate 5	average	SD
0	539.63	539.616				539.62	0.01
30	539.763	539.853				539.81	0.06
45	539.96	539.907				539.93	0.04
60	539.915	540.288				540.10	0.26
300	539.987	540.037				540.01	0.04
1500	540.038	540.234				540.14	0.14
3600	540.254	540.281				540.27	0.02
7200	540.242	540.518				540.38	0.20
14400	540.464	540.654				540.56	0.13

72-84 ALHAPSQIVAAIK

Charge 2

IPTG

Time	centroid	D
0	660.2685	-0.01
30	660.504	1.38
45	660.4725	1.19
60	660.6385	2.17
300	660.5255	1.51
1500	660.6485	2.23
3600	660.7385	2.76
7200	660.8125	3.20
14400	660.8715	3.54

ONPF

Time	centroid	D
0	660.278	0.05
30	660.467	1.16
45	660.952	4.02
60	660.815	3.21
300	660.926	3.87
1500	661.085	4.80
3600	661.412	6.73
7200	661.308	6.12
14400	661.64	8.07

APO

Time	centroid	D
0	660.267	-0.02
30	660.655	2.27
45	660.6225	2.08
60	660.735	2.74
300	660.698	2.52
1500	661.1165	4.99
3600	661.29	6.01
7200	661.372	6.49
14400	661.522	7.38

DNA

Time	centroid	D
0	660.258	-0.07
30	660.585	1.86
45	660.731	2.72
60	660.652	2.25
300	660.703	2.55
1500	660.7575	2.87
3600	660.8785	3.59
7200	660.8935	3.67
14400	660.834	3.32

ONPFDNA

Time	centroid	D
0	660.2625	-0.04
30	660.6295	2.12
45	660.7215	2.66
60	660.7135	2.61
300	660.678	2.40
1500	660.697	2.52
3600	660.9735	4.15
7200	660.997	4.28
14400	660.7615	2.90

TMG

Time	centroid	D
0	660.2605	-0.06
30	660.5065	1.39
45	660.708	2.58
60	660.541	1.60
300	660.706	2.57
1500	660.7045	2.56
3600	661.162	5.26
7200	661.0695	4.71
14400	661.1225	5.02

control 660.27
infinity 661.967

$$D(t) = \frac{M_t - M_0}{M_\infty - M_0} * N$$

IPTG

Time	Replicate 1	Replicate 2	Replicate 3	Replicate 4	Replicate5	average	SD
0	660.288	660.249				660.27	0.03
30	660.504					660.50	
45	660.496	660.449				660.47	0.03
60	660.588	660.689				660.64	0.07
300	660.566	660.485				660.53	0.06
1500	660.675	660.622				660.65	0.04
3600	660.748	660.729				660.74	0.01
7200	660.813	660.812				660.81	0.00
14400	660.861	660.882				660.87	0.01

ONPF

Time	Replicate 1	Replicate 2	Replicate 3	Replicate 4	average	SD
0	660.278				660.28	
30	660.467				660.47	
45	660.952				660.95	
60	660.815				660.82	
300	660.926				660.93	
1500	661.085				661.09	
3600	661.412				661.41	
7200	661.308				661.31	
14400	661.64				661.64	

APO

Time	Replicate 1	Replicate 2	Replicate 3	Replicate 4	Replicate 5	average	SD
0	660.253	660.281				660.27	0.02
30	660.655					660.66	
45	660.671	660.574				660.62	0.07
60	660.644	660.826				660.74	0.13
300	660.711	660.685				660.70	0.02
1500	661.087	661.146				661.12	0.04
3600	661.352	661.228				661.29	0.09
7200	661.538	661.206				661.37	0.23
14400	661.628	661.416				661.52	0.15

DNA

Time	Replicate 1	Replicate 2	Replicate 3	Replicate 4	Replicate 5	average	SD
0	660.267	660.249				660.26	0.01
30	660.588	660.582				660.59	0.00
45	660.841	660.621				660.73	0.16
60	660.676	660.628				660.65	0.03
300	660.744	660.662				660.70	0.06
1500	660.809	660.706				660.76	0.07
3600	661.055	660.702				660.88	0.25
7200	661.002	660.785				660.89	0.15
14400	661.169	660.499				660.83	0.47

ONPFDNA

Time	Replicate 1	Replicate 2	Replicate 3	Replicate 4	average	SD
0	660.27	660.255			660.26	0.01
30	660.681	660.578			660.63	0.07
45	660.737	660.706			660.72	0.02
60	660.68	660.747			660.71	0.05
300	660.728	660.628			660.68	0.07
1500	660.658	660.736			660.70	0.06
3600	661.072	660.875			660.97	0.14
7200	661.137	660.857			661.00	0.20
14400	660.725	660.798			660.76	0.05

TMG

Time	Replicate 1	Replicate 2	Replicate 3	Replicate 4	Replicate 5	average	SD
0	660.276	660.245				660.26	0.02
30	660.418	660.595				660.51	0.13
45	660.612	660.804				660.71	0.14
60	660.541					660.54	
300	660.614	660.798				660.71	0.13
1500	660.67	660.739				660.70	0.05
3600	660.914	661.41				661.16	0.35
7200	660.975	661.164				661.07	0.13
14400	661.121	661.124				661.12	0.00

77-82 SQIVAA

Charge 1

IPTG

Time	centroid	D
0	588.65	0.01
30	588.72	0.10
45	588.74	0.13
60	588.75	0.14
300	588.76	0.15
1500	588.73	0.12
3600	588.72	0.11
7200	588.75	0.13
14400	588.75	0.14

ONPF

Time	centroid	D
0	588.77	0.16
30	588.79	0.19
45	588.92	0.36
60	588.86	0.28
300	588.86	0.27
1500	589.11	0.59
3600	589.16	0.66
7200	589.42	0.99
14400	589.70	1.34

APO

Time	centroid	D
0	588.64	0.01
30	588.72	0.11
45	588.74	0.13
60	588.83	0.23
300	588.88	0.31
1500	589.17	0.67
3600	589.34	0.89
7200	589.50	1.09
14400	589.69	1.33

DNA

Time	centroid	D
0	588.61	-0.04
30	588.76	0.15
45	588.77	0.17
60	588.73	0.12
300	588.72	0.11
1500	588.79	0.19
3600	588.81	0.22
7200	588.82	0.23
14400	588.87	0.29

ONPFDNA

Time	centroid	D
0	588.67	0.04
30	588.67	0.03
45	588.68	0.04
60	588.72	0.10
300		
1500	588.82	0.23
3600	588.74	0.12
7200	588.74	0.13
14400	588.78	0.17

TMG

Time	centroid	D
0	588.68	0.05
30	588.76	0.15
45	588.80	0.20
60	588.89	0.32
300	588.77	0.16
1500	588.82	0.23
3600	588.83	0.24
7200	588.83	0.24
14400	588.94	0.38

control	588.64
infinity	591.793

$$D(t) = \frac{Mt - M0}{M\infty - M0} * N$$

IPTG

Time	Replicate 1	Replicate 2	Replicate 3	Replicate 4	Replicate5	average	SD
0	588.665	588.637				588.65	0.02
30	588.742	588.7				588.72	0.03
45	588.749	588.736				588.74	0.01
60	588.78	588.718				588.75	0.04
300	588.799	588.711				588.76	0.06
1500	588.738	588.726				588.73	0.01
3600	588.74	588.708				588.72	0.02
7200	588.748	588.744				588.75	0.00
14400	588.771	588.727				588.75	0.03

ONPF

Time	Replicate 1	Replicate 2	Replicate 3	Replicate 4	average	SD
0	588.713	588.823			588.77	0.08
30	588.79				588.79	
45	588.923				588.92	
60	588.904	588.812			588.86	0.07
300	588.855				588.86	
1500	589.108				589.11	
3600	589.358	588.961			589.16	0.28
7200	589.547	589.291			589.42	0.18
14400	589.697				589.70	

APO

Time	Replicate 1	Replicate 2	Replicate 3	Replicate 4	Replicate 5	average	SD
0	588.648	588.641				588.64	0.00
30	588.737	588.71				588.72	0.02
45	588.75	588.735				588.74	0.01
60	588.764	588.886				588.83	0.09
300	588.818	588.948				588.88	0.09
1500	589.053	589.288				589.17	0.17
3600	589.337	589.342				589.34	0.00
7200	589.704	589.294				589.50	0.29
14400	589.62	589.755				589.69	0.10

DNA

Time	Replicate 1	Replicate 2	Replicate 3	Replicate 4	Replicate 5	average	SD
0	588.622	588.6				588.61	0.02
30	588.788	588.732				588.76	0.04
45	588.801	588.748				588.77	0.04
60	588.767	588.699				588.73	0.05
300	588.734	588.713				588.72	0.01
1500	588.855	588.725				588.79	0.09
3600	588.821	588.806				588.81	0.01
7200	588.881	588.765				588.82	0.08
14400	589.023	588.709				588.87	0.22

ONPFDNA

Time	Replicate 1	Replicate 2	Replicate 3	Replicate 4	average	SD
0	588.707	588.634			588.67	0.05
30	588.647	588.687			588.67	0.03
45	588.666	588.684			588.68	0.01
60	588.74	588.702			588.72	0.03
300						
1500	588.923	588.715			588.82	0.15
3600	588.757	588.714			588.74	0.03
7200	588.732	588.754			588.74	0.02
14400	588.795	588.757			588.78	0.03

TMG

Time	Replicate 1	Replicate 2	Replicate 3	Replicate 4	Replicate 5	average	SD
0	588.704	588.65				588.68	0.04
30	588.78	588.736				588.76	0.03
45	588.842	588.758				588.80	0.06
60	588.825	588.96				588.89	0.10
300		588.768				588.77	
1500	588.78	588.86				588.82	0.06
3600	588.896	588.755				588.83	0.10
7200	588.859	588.796				588.83	0.04
14400	588.93	588.955				588.94	0.02

77-84 SQIVAAIK

Charge 1

IPTG

Time	centroid	D
0	829.99	0.01
30	830.084	0.12
45	830.092	0.13
60	830.1165	0.16
300	830.129	0.18
1500	830.1035	0.15
3600	830.0915	0.13
7200	830.1125	0.16
14400	830.1235	0.17

ONPF

Time	centroid	D
0	829.9955	0.02
30	830.07	0.11
45	830.111	0.16
60	830.247	0.32
300	830.191	0.25
1500	830.3705	0.47
3600	830.5665	0.70
7200	830.7195	0.88
14400	831.10	1.33

APO

Time	centroid	D
0	829.98	0.00
30	830.0455	0.08
45	830.096	0.14
60	830.2005	0.26
300	830.163	0.22
1500	830.521	0.64
3600	830.693	0.85
7200	830.9315	1.13
14400	831.1015	1.34

DNA

Time	centroid	D
0	829.963	-0.02
30	830.0655	0.10
45	830.06	0.10
60	830.1155	0.16
300	830.113	0.16
1500	830.1225	0.17
3600	830.2015	0.26
7200	830.2135	0.28
14400	830.2695	0.34

ONPFDNA

Time	centroid	D
0	829.98	0.00
30	830.074	0.11
45	830.111	0.16
60	830.105	0.15
300	830.095	0.14
1500	830.13	0.18
3600	830.166	0.22
7200	830.1975	0.26
14400	830.178	0.24

TMG

Time	centroid	D
0	829.973	-0.01
30	830.0785	0.12
45	830.1405	0.19
60	830.181	0.24
300	830.126	0.17
1500	830.146	0.20
3600	830.15	0.20
7200	830.15	0.20
14400	830.2695	0.34

control 829.98
infinity 835.018

$$D(t) = \frac{M_t - M_0}{M_\infty - M_0} \cdot N$$

IPTG

Time	Replicate 1	Replicate 2	Replicate 3	Replicate 4	Replicate5	average	SD
0	830.001	829.979				829.99	0.02
30	830.111	830.057				830.08	0.04
45	830.118	830.066				830.09	0.04
60	830.135	830.098				830.12	0.03
300	830.182	830.076				830.13	0.07
1500	830.11	830.097				830.10	0.01
3600	830.123	830.06				830.09	0.04
7200	830.112	830.113				830.11	0.00
14400	830.156	830.091				830.12	0.05

ONPF

Time	Replicate 1	Replicate 2	Replicate 3	Replicate 4	average	SD
0	829.993	829.998			830.00	0.00
30	830.083	830.057			830.07	0.02
45		830.111			830.11	
60	830.245	830.249			830.25	0.00
300	830.244	830.138			830.19	0.07
1500	830.419	830.322			830.37	0.07
3600	830.812	830.321			830.57	0.35
7200	830.829	830.61			830.72	0.15
14400	831.098				831.10	

APO

Time	Replicate 1	Replicate 2	Replicate 3	Replicate 4	Replicate 5	average	SD
0	829.974	829.986				829.98	0.01
30	830.03	830.061				830.05	0.02
45	830.109	830.083				830.10	0.02
60	830.102	830.299				830.20	0.14
300	830.156	830.17				830.16	0.01
1500	830.363	830.679				830.52	0.22
3600	830.683	830.703				830.69	0.01
7200	831.049	830.814				830.93	0.17
14400	831.067	831.136				831.10	0.05

DNA

Time	Replicate 1	Replicate 2	Replicate 3	Replicate 4	Replicate 5	average	SD
0	829.949	829.977				829.96	0.02
30	830.094	830.037				830.07	0.04
45		830.06				830.06	
60	830.126	830.105				830.12	0.01
300	830.148	830.078				830.11	0.05
1500	830.125	830.12				830.12	0.00
3600	830.274	830.129				830.20	0.10
7200	830.27	830.157				830.21	0.08
14400	830.438	830.101				830.27	0.24

ONPFDNA

Time	Replicate 1	Replicate 2	Replicate 3	Replicate 4	average	SD
0	829.982	829.978			829.98	0.00
30	830.091	830.057			830.07	0.02
45	830.14	830.082			830.11	0.04
60	830.116	830.094			830.11	0.02
300	830.101	830.089			830.10	0.01
1500	830.115	830.145			830.13	0.02
3600	830.264	830.068			830.17	0.14
7200	830.279	830.116			830.20	0.12
14400	830.218	830.138			830.18	0.06

TMG

Time	Replicate 1	Replicate 2	Replicate 3	Replicate 4	Replicate 5	average	SD
0	829.981	829.965				829.97	0.01
30	830.061	830.096				830.08	0.02
45	830.168	830.113				830.14	0.04
60	830.111	830.251				830.18	0.10
300	830.122	830.13				830.13	0.01
1500	830.107	830.185				830.15	0.06
3600	830.183	830.117				830.15	0.05
7200	830.158	830.142				830.15	0.01
14400	830.225	830.314				830.27	0.06

79-84 IVAAIK

Charge 1

IPTG

Time	centroid	D
0	614.76	0.01
30	614.84	0.10
45	614.84	0.10
60	614.84	0.10
300	614.84	0.11
1500	614.83	0.09
3600	614.83	0.09
7200	614.83	0.10
14400	614.84	0.11

ONPF

Time	centroid	D
0	614.77	0.02
30	614.83	0.09
45	614.94	0.21
60	614.90	0.18
300	614.88	0.15
1500	614.97	0.26
3600	615.07	0.37
7200	615.16	0.46
14400	615.15	0.45

APO

Time	centroid	D
0	614.75	0.00
30	614.81	0.07
45	614.83	0.10
60	614.91	0.19
300	614.86	0.13
1500	615.08	0.38
3600	615.21	0.52
7200	615.33	0.67
14400	615.39	0.73

DNA

Time	centroid	D
0	614.75	0.00
30	614.82	0.08
45	614.87	0.14
60	614.85	0.12
300	614.84	0.10
1500	614.86	0.12
3600	614.89	0.16
7200	614.91	0.18
14400	614.93	0.20

ONPFDNA

Time	centroid	D
0	614.77	0.02
30	614.82	0.08
45	614.84	0.11
60	614.84	0.10
300	614.83	0.10
1500	614.85	0.11
3600	614.86	0.13
7200	614.88	0.14
14400	614.90	0.17

TMG

Time	centroid	D
0	614.76	0.01
30	614.82	0.08
45	614.85	0.12
60	614.88	0.15
300	614.85	0.11
1500	614.86	0.13
3600	614.85	0.12
7200	614.83	0.09
14400	614.95	0.23

control 614.75
infinity 618.244

$$D(t) = \frac{M_t - M_0}{M_\infty - M_0} * N$$

IPTG

Time	Replicate 1	Replicate 2	Replicate 3	Replicate 4	Replicate5	average	SD
0	614.761	614.761				614.76	0.00
30	614.86	614.812				614.84	0.03
45	614.841	614.829				614.84	0.01
60	614.856	614.823				614.84	0.02
300	614.86	614.824				614.84	0.03
1500	614.828	614.835				614.83	0.00
3600	614.836	614.819				614.83	0.01
7200	614.828	614.839				614.83	0.01
14400	614.856	614.832				614.84	0.02

ONPF

Time	Replicate 1	Replicate 2	Replicate 3	Replicate 4	average	SD
0	614.77	614.773			614.77	0.00
30	614.836	614.818			614.83	0.01
45	615.034	614.836			614.94	0.14
60	614.91	614.898			614.90	0.01
300	614.907	614.85			614.88	0.04
1500	615.002	614.944			614.97	0.04
3600	615.186	614.962			615.07	0.16
7200	615.206	615.105			615.16	0.07
14400	615.444	614.846			615.15	0.42

APO

Time	Replicate 1	Replicate 2	Replicate 3	Replicate 4	Replicate 5	average	SD
0	614.74	614.764				614.75	0.02
30	614.805	614.811				614.81	0.00
45	614.834	614.833				614.83	0.00
60	614.825	615.001				614.91	0.12
300	614.855	614.874				614.86	0.01
1500	614.969	615.188				615.08	0.15
3600	615.17	615.244				615.21	0.05
7200	615.4	615.268				615.33	0.09
14400	615.255	615.527				615.39	0.19

DNA

Time	Replicate 1	Replicate 2	Replicate 3	Replicate 4	Replicate 5	average	SD
0	614.733	614.76				614.75	0.02
30	614.833	614.803				614.82	0.02
45	614.923	614.818				614.87	0.07
60	614.855	614.848				614.85	0.00
300	614.851	614.821				614.84	0.02
1500	614.861	614.853				614.86	0.01
3600	614.927	614.852				614.89	0.05
7200	614.928	614.889				614.91	0.03
14400	615.027	614.829				614.93	0.14

ONPFDNA

Time	Replicate 1	Replicate 2	Replicate 3	Replicate 4	average	SD
0	614.757	614.773			614.77	0.01
30	614.825	614.812			614.82	0.01
45	614.859	614.825			614.84	0.02
60	614.847	614.833			614.84	0.01
300	614.838	614.831			614.83	0.00
1500	614.84	614.855			614.85	0.01
3600	614.893	614.834			614.86	0.04
7200	614.922	614.828			614.88	0.07
14400	614.916	614.883			614.90	0.02

TMG

Time	Replicate 1	Replicate 2	Replicate 3	Replicate 4	Replicate 5	average	SD
0	614.768	614.748				614.76	0.01
30	614.809	614.827				614.82	0.01
45	614.866	614.835				614.85	0.02
60	614.834	614.931				614.88	0.07
300	614.841	614.855				614.85	0.01
1500	614.832	614.889				614.86	0.04
3600	614.875	614.828				614.85	0.03
7200	614.854	614.809				614.83	0.03
14400	614.932	614.965				614.95	0.02

82-93 AIKSRADQLGAS

Charge 2

IPTG

Time	centroid	D
0	609.1812	-0.01
30	609.5616	1.29
45	609.651	1.59
60	609.7014	1.76
300	609.6928	1.73
1500	609.936	2.56
3600	610.0612	2.99
7200	610.2114	3.50
14400	610.27	3.70

ONPF

Time	centroid	D
0	609.1915	0.03
30	609.452	0.91
45	609.6	1.42
60	609.6	1.42
300	609.6177	1.48
1500	609.825	2.18
3600	609.9018	2.44
7200	609.9843	2.72
14400	610.20	3.45

APO

Time	centroid	D
0	609.1836	0.00
30	609.5474	1.24
45	609.5615	1.29
60	609.6413	1.56
300	609.6488	1.58
1500	609.874	2.35
3600	610.0712	3.02
7200	610.2108	3.49
14400	610.4718	4.38

DNA

Time	centroid	D
0	609.1773	-0.02
30	609.5527	1.26
45	609.6475	1.58
60	609.7233	1.84
300	609.7193	1.82
1500	609.8813	2.37
3600	609.869	2.33
7200	609.9237	2.52
14400	609.949	2.60

ONPFDNA

Time	centroid	D
0	609.1755	-0.03
30	609.5128	1.12
45	609.584	1.36
60	609.6305	1.52
300	609.6445	1.57
1500	609.7778	2.02
3600	609.9385	2.57
7200	609.8933	2.41
14400	609.948	2.60

TMG

Time	centroid	D
0	609.1835	0.00
30	609.5505	1.25
45	609.7125	1.80
60	609.6545	1.60
300	609.718	1.82
1500	609.846	2.25
3600	610.1915	3.43
7200	610.2625	3.67
14400	610.4825	4.42

control 609.1836
infinity 612.123

$$D(t) = \frac{Mt - M0}{M\infty - M0} * N$$

IPTG

Time	Replicate 1	Replicate 2	Replicate 3	Replicate 4	Replicate5	average	SD
0	609.193	609.172	609.186	609.176	609.179	609.18	0.01
30	609.652	609.483	609.655	609.54	609.478	609.56	0.09
45	609.726	609.576				609.65	0.11
60	609.734	609.58	609.957	609.636	609.6	609.70	0.15
300	609.756	609.597	609.836	609.707	609.568	609.69	0.11
1500	610.139	609.828	610.013	609.907	609.793	609.94	0.14
3600	610.371	610.092	610.025	609.976	609.842	610.06	0.20
7200	610.611	610.208	610.238	610.091	609.909	610.21	0.26
14400	610.704	610.235	610.093	610.352	609.966	610.27	0.28

ONPF

Time	Replicate 1	Replicate 2	Replicate 3	Replicate 4	average	SD
0	609.205	609.205	609.179	609.177	609.19	0.02
30	609.415	609.37	609.574	609.449	609.45	0.09
45	609.763	609.437			609.60	0.23
60	609.626	609.588		609.586	609.60	0.02
300	609.664		609.65	609.539	609.62	0.07
1500	609.819	609.665	610.044	609.772	609.83	0.16
3600	610.02	609.715	610.064	609.808	609.90	0.17
7200	609.963	609.835	610.266	609.873	609.98	0.20
14400	610.378	609.842	610.363	610.203	610.20	0.25

APO

Time	Replicate 1	Replicate 2	Replicate 3	Replicate 4	Replicate 5	average	SD
0	609.167	609.175	609.226	609.181	609.169	609.18	0.02
30	609.568	609.432	609.792	609.494	609.451	609.55	0.15
45	609.684	609.439				609.56	0.17
60	609.663	609.629		609.572	609.701	609.64	0.05
300	609.639	609.494	609.912	609.632	609.567	609.65	0.16
1500	609.856	609.886	610.115	609.746	609.767	609.87	0.15
3600	610.239	609.995	610.32	609.946	609.856	610.07	0.20
7200	610.384	609.987	610.558	610.153	609.972	610.21	0.26
14400	610.841	610.342	610.607	610.289	610.28	610.47	0.25

DNA

Time	Replicate 1	Replicate 2	Replicate 3	Replicate 4	Replicate 5	average	SD
0	609.174	609.175	609.183			609.18	0.00
30	609.545	609.459	609.654			609.55	0.10
45	609.785	609.51				609.65	0.19
60	609.643	609.619	609.908			609.72	0.16
300	609.689	609.586	609.883			609.72	0.15
1500	609.813	609.824	610.007			609.88	0.11
3600	610.029	609.702	609.876			609.87	0.16
7200	610.033	609.746	609.992			609.92	0.16
14400	610.217	609.49	610.14			609.95	0.40

ONPFDNA

Time	Replicate 1	Replicate 2	Replicate 3	Replicate 4	average	SD
0	609.177	609.174	609.18	609.171	609.18	0.00
30	609.487	609.441	609.715	609.408	609.51	0.14
45	609.627	609.541			609.58	0.06
60	609.599	609.637	609.702	609.584	609.63	0.05
300	609.625	609.557	609.807	609.589	609.64	0.11
1500	609.599	609.707	610.092	609.713	609.78	0.22
3600	610.016	609.876	610.067	609.795	609.94	0.13
7200	609.973	609.831		609.876	609.89	0.07
14400	609.71	609.801	610.334	609.947	609.95	0.28

TMG

Time	Replicate 1	Replicate 2	Replicate 3	Replicate 4	Replicate 5	average	SD
0	609.19	609.177				609.18	0.01
30	609.442	609.659				609.55	0.15
45	609.722	609.703				609.71	0.01
60	609.621	609.688				609.65	0.05
300	609.673	609.763				609.72	0.06
1500	609.754	609.938				609.85	0.13
3600	610.072	610.311				610.19	0.17
7200	610.038	610.487				610.26	0.32
14400	610.343	610.622				610.48	0.20

87-93 ADQLGAS

Charge 1

IPTG

Time	centroid	D
0	661.60	-0.06
30	662.38	1.36
45	662.39	1.39
60	662.32	1.25
300	662.44	1.48
1500	662.65	1.85
3600	663.77	3.90
7200	663.43	3.28
14400	663.32	3.07

ONPF

Time	centroid	D
0	661.60	-0.06
30	662.02	0.72
45	662.08	0.83
60	662.29	1.20
300	662.17	0.99
1500	662.65	1.87
3600	662.68	1.92
7200	662.66	1.87
14400	#DIV/0!	#DIV/0!

APO

Time	centroid	D
0	661.63	0.00
30	662.15	0.94
45	662.22	1.07
60	662.38	1.38
300	662.22	1.07
1500	662.83	2.19
3600	663.00	2.51
7200	663.14	2.76
14400	663.62	3.62

DNA

Time	centroid	D
0	661.60	-0.05
30	662.16	0.96
45	662.34	1.29
60	662.46	1.51
300	662.28	1.18
1500	662.65	1.86
3600	662.62	1.80
7200	662.70	1.96
14400	662.60	1.77

ONPFDNA

Time	centroid	D
0	661.57	-0.11
30	662.04	0.75
45	662.17	0.99
60	662.35	1.31
300	662.39	1.38
1500	662.44	1.48
3600	662.70	1.95
7200	662.38	1.38
14400	662.23	1.10

TMG

Time	centroid	D
0	661.67	0.07
30	662.36	1.33
45	662.57	1.71
60	662.36	1.32
300	662.58	1.74
1500	662.84	2.21
3600	663.37	3.18
7200	663.32	3.09
14400	663.50	3.42

control	661.63
infinity	664.37

$$D(t) = \frac{M_t - M_0}{M_\infty - M_0} \, {*N}$$

IPTG

Time	Replicate 1	Replicate 2	Replicate 3	Replicate 4	Replicate5	average	SD
0	661.568	661.624				661.60	0.04
30	662.375					662.38	
45	662.451	662.335				662.39	0.08
60		662.317				662.32	
300	662.527	662.36				662.44	0.12
1500		662.646				662.65	
3600	663.441	664.091				663.77	0.46
7200	663.628	663.223				663.43	0.29
14400	663.551	663.079				663.32	0.33

ONPF

Time	Replicate 1	Replicate 2	Replicate 3	Replicate 4	average	SD
0	661.48	661.711			661.60	0.16
30		662.022			662.02	
45	662.097	662.072			662.08	0.02
60	662.247	662.326			662.29	0.06
300		662.171			662.17	
1500	662.878	662.431			662.65	0.32
3600	662.881	662.488			662.68	0.28
7200		662.656			662.66	
14400						

APO

Time	Replicate 1	Replicate 2	Replicate 3	Replicate 4	Replicate 5	average	SD
0	661.634	661.63				661.63	0.00
30	662.169	662.124				662.15	0.03
45	662.384	662.048				662.22	0.24
60	662.41	662.359				662.38	0.04
300	662.239	662.193				662.22	0.03
1500	662.881	662.776				662.83	0.07
3600	663.107	662.9				663.00	0.15
7200	663.434	662.85				663.14	0.41
14400	664.084	663.148				663.62	0.66

DNA

Time	Replicate 1	Replicate 2	Replicate 3	Replicate 4	Replicate 5	average	SD
0	661.577	661.63				661.60	0.04
30	662.118	662.199				662.16	0.06
45	662.525	662.145				662.34	0.27
60	662.585	662.327				662.46	0.18
300	662.251	662.302				662.28	0.04
1500	662.565	662.733				662.65	0.12
3600	662.694	662.542				662.62	0.11
7200		662.702				662.70	
14400	663.087	662.11				662.60	0.69

ONPFDNA

Time	Replicate 1	Replicate 2	Replicate 3	Replicate 4	average	SD
0	661.529	661.611			661.57	0.06
30	662	662.087			662.04	0.06
45	662.179	662.162			662.17	0.01
60	662.397	662.297			662.35	0.07
300	662.537	662.236			662.39	0.21
1500	662.413	662.466			662.44	0.04
3600	662.656	662.744			662.70	0.06
7200	661.945	662.823			662.38	0.62
14400	661.941	662.528			662.23	0.42

TMG

Time	Replicate 1	Replicate 2	Replicate 3	Replicate 4	Replicate 5	average	SD
0	661.697	661.642				661.67	0.04
30	662.259	662.462				662.36	0.14
45	662.527	662.604				662.57	0.05
60	662.482	662.23				662.36	0.18
300	662.555	662.61				662.58	0.04
1500	662.657	663.025				662.84	0.26
3600	663.104	663.643				663.37	0.38
7200	663.073	663.57				663.32	0.35
14400	663.422	663.582				663.50	0.11

94-98 VVVSM

Charge 1

IPTG

Time	centroid	D
0	534.609	0.00
30	535.287	0.86
45	535.322	0.90
60	535.344	0.93
300	535.337	0.92
1500	535.649	1.32
3600	535.783	1.49
7200	535.966	1.72
14400	535.998	1.76

ONPF

Time	centroid	D
0	534.773	0.21
30	535.0925	0.61
45	535.4065	1.01
60	535.5555	1.20
300	535.61	1.27
1500	535.7455	1.44
3600	535.732	1.42
7200	535.632	1.30
14400	535.67	1.35

APO

Time	centroid	D
0	534.611	0.00
30	535.2675	0.83
45	535.293	0.87
60	535.546	1.19
300	535.69	1.37
1500	535.881	1.61
3600	535.9345	1.68
7200	535.7665	1.47
14400	535.937	1.68

DNA

Time	centroid	D
0	534.6145	0.00
30	535.3825	0.98
45	535.7515	1.45
60	535.8375	1.56
300	535.879	1.61
1500	535.945	1.69
3600	535.895	1.63
7200	535.685	1.36
14400	535.825	1.54

ONPFDNA

Time	centroid	D
0	534.613	0.00
30	535.306	0.88
45	535.7015	1.39
60	535.7595	1.46
300	535.8265	1.54
1500	535.7865	1.49
3600	535.9385	1.69
7200	535.748	1.44
14400	535.6975	1.38

TMG

Time	centroid	D
0	534.665	0.07
30	535.166	0.70
45	535.4085	1.01
60	535.3375	0.92
300	535.3735	0.97
1500	535.4975	1.13
3600	535.808	1.52
7200	535.809	1.52
14400	535.975	1.73

control 534.611
infinity 536.973

$$D(t) = \frac{M_t - M_0}{M_\infty - M_0} * N$$

IPTG

Time	Replicate 1	Replicate 2	Replicate 3	Replicate 4	Replicate5	average	SD
0	534.609	534.605				534.61	0.00
30	535.287	535.059				535.17	0.16
45	535.322	535.235				535.28	0.06
60	535.344	535.298				535.32	0.03
300	535.337	535.214				535.28	0.09
1500	535.649	535.383				535.52	0.19
3600	535.783	535.733				535.76	0.04
7200	535.966	535.73				535.85	0.17
14400	535.998	535.713				535.86	0.20

ONPF

Time	Replicate 1	Replicate 2	Replicate 3	Replicate 4	average	SD
0	534.797	534.749			534.77	0.03
30	535.181	535.004			535.09	0.13
45	535.645	535.168			535.41	0.34
60	535.63	535.481			535.56	0.11
300	535.818	535.402			535.61	0.29
1500	535.906	535.585			535.75	0.23
3600	535.862	535.602			535.73	0.18
7200	535.756	535.508			535.63	0.18
14400	535.96	535.38			535.67	0.41

APO

Time	Replicate 1	Replicate 2	Replicate 3	Replicate 4	Replicate 5	average	SD
0	534.607	534.615				534.61	0.01
30	535.408	535.127				535.27	0.20
45	535.385	535.201				535.29	0.13
60	535.595	535.497				535.55	0.07
300	535.927	535.453				535.69	0.34
1500	535.995	535.767				535.88	0.16
3600	536.06	535.809				535.93	0.18
7200	535.96	535.573				535.77	0.27
14400	536.117	535.757				535.94	0.25

DNA

Time	Replicate 1	Replicate 2	Replicate 3	Replicate 4	Replicate 5	average	SD
0	534.609	534.62				534.61	0.01
30	535.396	535.369				535.38	0.02
45	535.931	535.572				535.75	0.25
60	535.933	535.742				535.84	0.14
300	535.968	535.79				535.88	0.13
1500	536.001	535.889				535.95	0.08
3600	535.995	535.795				535.90	0.14
7200	535.863	535.507				535.69	0.25
14400	536.014	535.636				535.83	0.27

ONPFDNA

Time	Replicate 1	Replicate 2	Replicate 3	Replicate 4	average	SD
0	534.618	534.608			534.61	0.01
30	535.345	535.267			535.31	0.06
45	535.826	535.577			535.70	0.18
60	535.835	535.684			535.76	0.11
300	535.932	535.721			535.83	0.15
1500	535.869	535.704			535.79	0.12
3600	535.983	535.894			535.94	0.06
7200	535.839	535.657			535.75	0.13
14400	535.84	535.555			535.70	0.20

TMG

Time	Replicate 1	Replicate 2	Replicate 3	Replicate 4	Replicate 5	average	SD
0	534.723	534.607				534.67	0.08
30	535.054	535.278				535.17	0.16
45	535.489	535.328				535.41	0.11
60	535.375	535.3				535.34	0.05
300	535.372	535.375				535.37	0.00
1500	535.47	535.525				535.50	0.04
3600	535.785	535.831				535.81	0.03
7200	535.653	535.965				535.81	0.22
14400	535.901	536.049				535.98	0.10

98-105 MVERSGVE

Charge 2

IPTG

Time	centroid	D
0	453.9893	0.00
30	455.1028	4.19
45		
60	455.2693	4.82
300	455.2928	4.91
1500	455.2773	4.85
3600	455.2728	4.83
7200	455.308	4.97
14400	455.2995	4.94

ONPF

Time	centroid	D
0	454.0058	0.06
30	454.8635	3.29
45		
60	455.15	4.37
300	455.1777	4.48
1500	455.1448	4.35
3600	455.222	4.64
7200	455.157	4.40
14400	455.27	4.83

APO

Time	centroid	D
0	453.9948	0.02
30	455.0735	4.08
45		
60	455.2315	4.68
300	455.2135	4.61
1500	455.2643	4.80
3600	455.2575	4.78
7200	455.3	4.94
14400	455.3853	5.26

DNA

Time	centroid	D
0	453.9883	-0.01
30	454.9477	3.61
45		
60	455.21	4.60
300	455.2633	4.80
1500	455.2407	4.71
3600	455.267	4.81
7200	455.2173	4.63
14400	455.2623	4.80

ONPFDNA

Time	centroid	D
0	453.9857	-0.02
30	454.944	3.60
45		
60	455.2563	4.77
300	455.274	4.84
1500	455.2013	4.57
3600	455.3243	5.03
7200	455.2117	4.60
14400	455.179	4.48

TMG

Time	centroid	D
0	454.006	0.06
30	455.288	4.89
45	455.429	5.42
60	455.486	5.64
300	455.533	5.82
1500	455.458	5.53
3600	455.511	5.73
7200	455.4815	5.62
14400	455.564	5.93

control 453.99
infinity 455.582

$$D(t) = \frac{Mt - M0}{M\infty - M0} *N$$

IPTG

Time	Replicate 1	Replicate 2	Replicate 3	Replicate 4	Replicate5	average	SD
0	453.98	453.995	453.987	453.995		453.99	0.01
30	455.645	454.923	454.844	454.999		455.10	0.37
45							
60	455.716	455.14	455.065	455.156		455.27	0.30
300	455.694	455.248	455.148	455.081		455.29	0.28
1500	455.701	455.256	455.148	455.004		455.28	0.30
3600	455.67	455.223	455.129	455.069		455.27	0.27
7200	455.701	455.185		455.038		455.31	0.35
14400	455.637	455.271	455.238	455.052		455.30	0.24

ONPF

Time	Replicate 1	Replicate 2	Replicate 3	Replicate 4	average	SD
0	454.055	453.989	453.992	453.987	454.01	0.03
30	454.941	454.81	454.675	455.028	454.86	0.15
45						
60	455.344	455.265	455.03	454.961	455.15	0.18
300	455.418	454.963		455.152	455.18	0.23
1500	455.388	455.201	455.002	454.988	455.14	0.19
3600	455.32	455.336	454.958	455.274	455.22	0.18
7200	455.287	455.282	454.951	455.108	455.16	0.16
14400	455.472	455.222	455.171	455.216	455.27	0.14

APO

Time	Replicate 1	Replicate 2	Replicate 3	Replicate 4	Replicate 5	average	SD
0	453.99	454.012	453.985	453.992		453.99	0.01
30	455.557	455.218	454.779	454.74		455.07	0.39
45							
60	455.705	455.558	454.75	454.913		455.23	0.47
300	455.573	455.34	455.062	454.879		455.21	0.31
1500	455.568	455.46	454.982	455.047		455.26	0.29
3600	455.614	455.477	455.054	454.885		455.26	0.34
7200	455.53	455.609	455.096	454.965		455.30	0.32
14400	455.661	455.746	455.063	455.071		455.39	0.37

DNA

Time	Replicate 1	Replicate 2	Replicate 3	Replicate 4	Replicate 5	average	SD
0	453.987	453.989	453.989			453.99	0.00
30	455.115	454.993	454.735			454.95	0.19
45							
60	455.378		455.042			455.21	0.24
300	455.496	455.432	454.862			455.26	0.35
1500	455.39	455.377	454.955			455.24	0.25
3600	455.464	455.447	454.89			455.27	0.33
7200	455.4	455.412	454.84			455.22	0.33
14400	455.403	455.377	455.007			455.26	0.22

ONPFDNA

Time	Replicate 1	Replicate 2	Replicate 3	Replicate 4	average	SD
0	453.99	453.976	453.991		453.99	0.01
30	455.027	455.253	454.552		454.94	0.36
45						
60	455.321	455.397	455.051		455.26	0.18
300	455.431	455.333	455.058		455.27	0.19
1500	455.093	455.432	455.079		455.20	0.20
3600	455.425	455.412	455.136		455.32	0.16
7200	455.379	455.179	455.077		455.21	0.15
14400	454.949	455.447	455.141		455.18	0.25

TMG

Time	Replicate 1	Replicate 2	Replicate 3	Replicate 4	Replicate 5	average	SD
0	454.03	453.982				454.01	0.03
30	454.932	455.644				455.29	0.50
45	455.269	455.589				455.43	0.23
60	455.439	455.533				455.49	0.07
300	455.42	455.646				455.53	0.16
1500	455.243	455.673				455.46	0.30
3600	455.304	455.718				455.51	0.29
7200	455.26	455.703				455.48	0.31
14400	455.387	455.741				455.56	0.25

109-114 AAVHNL

Charge 1

IPTG

Time	centroid	D
0	624.73	0.17
30	624.96	0.60
45	624.94	0.56
60	624.93	0.53
300	625.01	0.69
1500	625.26	1.17
3600	625.53	1.67
7200	625.83	2.25
14400	625.93	2.44

ONPF

Time	centroid	D
0	624.69	0.08
30	624.79	0.28
45	624.86	0.40
60	624.86	0.41
300	624.95	0.58
1500	625.13	0.91
3600	625.17	1.00
7200	625.15	0.96
14400	625.43	1.50

APO

Time	centroid	D
0	624.65	0.00
30	624.78	0.25
45	624.84	0.37
60	624.84	0.37
300	624.84	0.37
1500	625.07	0.80
3600	625.10	0.87
7200	625.53	1.69
14400	625.51	1.65

DNA

Time	centroid	D
0	624.67	0.04
30	624.78	0.25
45	624.85	0.40
60	624.83	0.35
300	624.95	0.59
1500	625.01	0.70
3600	625.07	0.80
7200	625.02	0.71
14400	624.99	0.66

ONPFDNA

Time	centroid	D
0	624.71	0.12
30	624.82	0.34
45	624.84	0.37
60	624.88	0.43
300	624.96	0.59
1500	624.94	0.55
3600	625.09	0.85
7200	625.03	0.73
14400	624.95	0.57

TMG

Time	centroid	D
0	624.67	0.04
30	624.86	0.40
45	624.98	0.64
60	624.92	0.51
300	625.00	0.68
1500	625.19	1.04
3600	625.44	1.51
7200	625.56	1.74
14400	625.86	2.31

control 624.6465
infinity 626.748

$$D(t) = \frac{M_t - M_0}{M_\infty - M_0} * N$$

IPTG

Time	Replicate 1	Replicate 2	Replicate 3	Replicate 4	Replicate5	average	SD
0	624.688	624.78				624.73	0.07
30	624.924	624.999				624.96	0.05
45	624.952	624.932				624.94	0.01
60	624.963	624.888				624.93	0.05
300	625.113	624.9				625.01	0.15
1500	625.526	624.993				625.26	0.38
3600	625.805	625.248				625.53	0.39
7200	626.068	625.591				625.83	0.34
14400	626.227	625.625				625.93	0.43

ONPF

Time	Replicate 1	Replicate 2	Replicate 3	Replicate 4	average	SD
0	624.671	624.707			624.69	0.03
30	624.82	624.763			624.79	0.04
45	624.915	624.802			624.86	0.08
60	624.843	624.88			624.86	0.03
300	624.941	624.966			624.95	0.02
1500	625.036	625.214			625.13	0.13
3600	625.225	625.118			625.17	0.08
7200	625.21	625.092			625.15	0.08
14400	625.432				625.43	

APO

Time	Replicate 1	Replicate 2	Replicate 3	Replicate 4	Replicate 5	average	SD
0	624.613	624.68				624.65	0.05
30	624.783	624.769				624.78	0.01
45	624.903	624.778				624.84	0.09
60	624.839	624.845				624.84	0.00
300	624.894	624.79				624.84	0.07
1500	625.08	625.052				625.07	0.02
3600	625.146	625.061				625.10	0.06
7200	625.86	625.209				625.53	0.46
14400	625.617	625.408				625.51	0.15

DNA

Time	Replicate 1	Replicate 2	Replicate 3	Replicate 4	Replicate 5	average	SD
0	624.652	624.685				624.67	0.02
30	624.808	624.744				624.78	0.05
45	624.945	624.764				624.85	0.13
60	624.837	624.827				624.83	0.01
300	625.06	624.849				624.95	0.15
1500	625.015	625.012				625.01	0.00
3600	625.218	624.915				625.07	0.21
7200	625.113	624.928				625.02	0.13
14400	625.175	624.813				624.99	0.26

ONPFDNA

Time	Replicate 1	Replicate 2	Replicate 3	Replicate 4	average	SD
0	624.667	624.75			624.71	0.06
30	624.823	624.824			624.82	0.00
45	624.834	624.843			624.84	0.01
60	624.91	624.84			624.88	0.05
300	625.051	624.859			624.96	0.14
1500	624.927	624.946			624.94	0.01
3600	625.161	625.026			625.09	0.10
7200		625.031			625.03	
14400	624.923	624.972			624.95	0.03

TMG

Time	Replicate 1	Replicate 2	Replicate 3	Replicate 4	Replicate 5	average	SD
0	624.684	624.656				624.67	0.02
30	624.851	624.859				624.86	0.01
45	625.11	624.851				624.98	0.18
60	624.96	624.872				624.92	0.06
300	625.047	624.961				625.00	0.06
1500	625.242	625.147				625.19	0.07
3600	625.539	625.336				625.44	0.14
7200	625.659	625.463				625.56	0.14
14400	625.929	625.791				625.86	0.10

115-122 LAQRVSGL

Charge 2

IPTG

Time	centroid	D
0	422.50	0.03
30	422.85	1.01
45	422.94	1.25
60	422.97	1.33
300	423.03	1.51
1500	423.19	1.94
3600	423.37	2.44
7200	423.49	2.79
14400	423.50	2.82

ONPF

Time	centroid	D
0	422.51	0.05
30	422.69	0.57
45	422.89	1.11
60	422.92	1.20
300	422.92	1.20
1500	423.04	1.54
3600	423.11	1.73
7200	423.13	1.79
14400	423.01	1.45

APO

Time	centroid	D
0	422.49	0.01
30	422.79	0.83
45	422.86	1.03
60	422.97	1.34
300	422.93	1.23
1500	423.18	1.92
3600	423.28	2.21
7200	423.34	2.36
14400	423.45	2.68

DNA

Time	centroid	D
0	422.49	0.01
30	422.60	0.29
45	422.68	0.52
60	422.67	0.49
300	422.66	0.46
1500	422.75	0.74
3600	422.79	0.84
7200	422.82	0.93
14400	422.83	0.94

ONPFDNA

Time	centroid	D
0	422.50	0.01
30	422.58	0.25
45	422.62	0.37
60	422.66	0.46
300	422.62	0.37
1500	422.69	0.57
3600	422.81	0.90
7200	422.82	0.91
14400	422.77	0.78

TMG

Time	centroid	D
0	422.50	0.02
30	422.84	0.97
45	423.01	1.44
60	422.92	1.20
300	423.10	1.70
1500	423.17	1.90
3600	423.38	2.48
7200	423.43	2.62
14400	423.59	3.07

control	422.49
infinity	424.647

$$D(t) = \frac{M_t - M_0}{M_\infty - M_0} *N$$

IPTG

Time	Replicate 1	Replicate 2	Replicate 3	Replicate 4	Replicate5	average	SD
0	422.509	422.489				422.50	0.01
30	422.966	422.743				422.85	0.16
45	423.002	422.879				422.94	0.09
60	422.979	422.959				422.97	0.01
300	423.119	422.947				423.03	0.12
1500	423.321	423.056				423.19	0.19
3600	423.454	423.281				423.37	0.12
7200	423.626	423.361				423.49	0.19
14400	423.664	423.34				423.50	0.23

ONPF

Time	Replicate 1	Replicate 2	Replicate 3	Replicate 4	average	SD
0	422.503	422.512			422.51	0.01
30	422.713	422.674			422.69	0.03
45	423.056	422.72			422.89	0.24
60	422.94	422.906			422.92	0.02
300	423.009	422.831			422.92	0.13
1500	423.122	422.963			423.04	0.11
3600	423.235	422.99			423.11	0.17
7200	423.195	423.07			423.13	0.09
14400	423.487	422.536			423.01	0.67

APO

Time	Replicate 1	Replicate 2	Replicate 3	Replicate 4	Replicate 5	average	SD
0	422.491	422.497				422.49	0.00
30	422.83	422.748				422.79	0.06
45	422.946	422.773				422.86	0.12
60	422.968	422.975				422.97	0.00
300	423.01	422.857				422.93	0.11
1500	423.189	423.17				423.18	0.01
3600	423.354	423.212				423.28	0.10
7200	423.468	423.211				423.34	0.18
14400	423.537	423.372				423.45	0.12

DNA

Time	Replicate 1	Replicate 2	Replicate 3	Replicate 4	Replicate 5	average	SD
0	422.492	422.494				422.49	0.00
30	422.624	422.567				422.60	0.04
45	422.766	422.589				422.68	0.13
60	422.657	422.678				422.67	0.01
300	422.678	422.635				422.66	0.03
1500	422.746	422.763				422.75	0.01
3600	422.863	422.722				422.79	0.10
7200	422.88	422.767				422.82	0.08
14400	423.016	422.64				422.83	0.27

ONPFDNA

Time	Replicate 1	Replicate 2	Replicate 3	Replicate 4	average	SD
0	422.498	422.492			422.50	0.00
30	422.597	422.566			422.58	0.02
45	422.655	422.588			422.62	0.05
60	422.658	422.652			422.66	0.00
300	422.64	422.608			422.62	0.02
1500	422.668	422.719			422.69	0.04
3600	422.865	422.761			422.81	0.07
7200	422.834	422.799			422.82	0.02
14400	422.776	422.767			422.77	0.01

TMG

Time	Replicate 1	Replicate 2	Replicate 3	Replicate 4	Replicate 5	average	SD
0	422.499	422.492				422.50	0.00
30	422.744	422.936				422.84	0.14
45	423.079	422.939				423.01	0.10
60	422.97	422.872				422.92	0.07
300	423.063	423.136				423.10	0.05
1500	423.119	423.224				423.17	0.07
3600	423.314	423.45				423.38	0.10
7200	423.297	423.567				423.43	0.19
14400	423.499	423.685				423.59	0.13

119-125 VSGLIIN

Charge 1

IPTG

Time	centroid	D
0	715.82	0.03
30	715.93	0.17
45	715.98	0.23
60	715.98	0.23
300	715.96	0.20
1500	715.97	0.22
3600	715.96	0.21
7200	715.98	0.22
14400	715.96	0.20

ONPF

Time	centroid	D
0	715.87	0.09
30	715.95	0.19
45	715.99	0.24
60	715.99	0.24
300	715.98	0.23
1500	715.98	0.23
3600	716.00	0.25
7200	715.95	0.20
14400	715.97	0.22

APO

Time	centroid	D
0	715.79	0.00
30	715.91	0.14
45	715.95	0.19
60	715.96	0.20
300	715.92	0.16
1500	715.92	0.15
3600	715.90	0.13
7200	715.88	0.10
14400	715.92	0.16

DNA

Time	centroid	D
0	715.85	0.06
30	715.94	0.18
45	715.92	0.16
60	715.95	0.19
300	715.95	0.19
1500	715.97	0.22
3600	715.99	0.24
7200	715.99	0.24
14400	716.00	0.25

ONPFDNA

Time	centroid	D
0	715.89	0.11
30	715.98	0.23
45	715.93	0.17
60	715.98	0.23
300	716.04	0.31
1500	715.97	0.21
3600	715.96	0.20
7200	716.02	0.27
14400	715.99	0.25

TMG

Time	centroid	D
0	715.81	0.02
30	715.95	0.19
45	715.95	0.19
60	716.01	0.26
300	716.00	0.26
1500	716.00	0.25
3600	715.96	0.21
7200	715.99	0.24
14400	716.01	0.26

control	715.79
infinity	719.867

$$D(t) = \frac{M_t - M_0}{M_\infty - M_0} * N$$

IPTG

Time	Replicate 1	Replicate 2	Replicate 3	Replicate 4	Replicate5	average	SD
0	715.803	715.832				715.82	0.02
30	715.937	715.924				715.93	0.01
45	715.996	715.962				715.98	0.02
60	715.963	716.005				715.98	0.03
300	715.997	715.924				715.96	0.05
1500	715.969	715.98				715.97	0.01
3600	716.001	715.928				715.96	0.05
7200	715.959	715.991				715.98	0.02
14400	715.974	715.943				715.96	0.02

ONPF

Time	Replicate 1	Replicate 2	Replicate 3	Replicate 4	average	SD
0	715.837	715.905			715.87	0.05
30	715.931	715.959			715.95	0.02
45	715.984	715.987			715.99	0.00
60	715.971	716.015			715.99	0.03
300	715.975	715.981			715.98	0.00
1500	715.989	715.971			715.98	0.01
3600	715.994	716.007			716.00	0.01
7200	715.937	715.97			715.95	0.02
14400	715.988	715.957			715.97	0.02

APO

Time	Replicate 1	Replicate 2	Replicate 3	Replicate 4	Replicate 5	average	SD
0	715.743	715.845				715.79	0.07
30	715.881	715.939				715.91	0.04
45	715.901	716.003				715.95	0.07
60	715.887	716.028				715.96	0.10
300	715.886	715.961				715.92	0.05
1500	715.897	715.941				715.92	0.03
3600	715.825	715.98				715.90	0.11
7200	715.807	715.95				715.88	0.10
14400	715.899	715.942				715.92	0.03

DNA

Time	Replicate 1	Replicate 2	Replicate 3	Replicate 4	Replicate 5	average	SD
0	715.82	715.871				715.85	0.04
30	715.956	715.932				715.94	0.02
45	715.951	715.891				715.92	0.04
60	715.967	715.927				715.95	0.03
300	715.94	715.951				715.95	0.01
1500	715.964	715.975				715.97	0.01
3600	716.029	715.95				715.99	0.06
7200	715.934	716.053				715.99	0.08
14400	715.984	716.015				716.00	0.02

ONPFDNA

Time	Replicate 1	Replicate 2	Replicate 3	Replicate 4	average	SD
0	715.854	715.921			715.89	0.05
30	715.962	716.004			715.98	0.03
45	715.959	715.906			715.93	0.04
60	715.975	715.985			715.98	0.01
300	715.968	716.12			716.04	0.11
1500	715.959	715.976			715.97	0.01
3600	715.956	715.963			715.96	0.00
7200	715.977	716.059			716.02	0.06
14400	716.007	715.981			715.99	0.02

TMG

Time	Replicate 1	Replicate 2	Replicate 3	Replicate 4	Replicate 5	average	SD
0	715.825	715.796				715.81	0.02
30	715.939	715.954				715.95	0.01
45	715.992	715.906				715.95	0.06
60	715.994	716.019				716.01	0.02
300	716.013	715.995				716.00	0.01
1500	716.022	715.969				716.00	0.04
3600	715.971	715.951				715.96	0.01
7200	715.967	716.007				715.99	0.03
14400	716.026	715.985				716.01	0.03

123-129 IINYPLD

Charge 1

IPTG

Time	centroid	D
0	847.95	0.00
30	848.09	0.19
45	848.09	0.21
60	848.09	0.20
300	848.11	0.23
1500	848.15	0.29
3600	848.13	0.27
7200	848.27	0.46
14400	848.37	0.62

ONPF

Time	centroid	D
0	847.95	0.00
30	848.05	0.14
45	848.10	0.21
60	848.10	0.21
300	848.15	0.29
1500	848.23	0.41
3600	848.28	0.49
7200	848.35	0.58
14400	848.30	0.52

APO

Time	centroid	D
0	847.96	0.00
30	848.06	0.16
45	848.06	0.15
60	848.09	0.21
300	848.10	0.22
1500	848.22	0.40
3600	848.36	0.61
7200	848.41	0.68
14400	848.51	0.83

DNA

Time	centroid	D
0	847.97	0.01
30	848.06	0.16
45	848.09	0.19
60	848.10	0.22
300	848.09	0.20
1500	848.20	0.37
3600	848.26	0.45
7200	848.30	0.51
14400	848.23	0.42

ONPFDNA

Time	centroid	D
0	847.95	-0.02
30	848.04	0.13
45	848.06	0.15
60	848.11	0.24
300	848.09	0.20
1500	848.18	0.34
3600	848.33	0.56
7200	848.35	0.59
14400	848.24	0.42

TMG

Time	centroid	D
0	847.98	0.03
30	848.11	0.23
45	848.11	0.23
60	848.11	0.23
300	848.13	0.26
1500	848.13	0.26
3600	848.20	0.36
7200	848.27	0.48
14400	848.39	0.66

control 847.96
infinity 850.616

$$D(t) = \frac{M_t - M_0}{M_\infty - M_0} * N$$

IPTG

Time	Replicate 1	Replicate 2	Replicate 3	Replicate 4	Replicate5	average	SD
0	847.941	847.968				847.95	0.02
30	848.106	848.064				848.09	0.03
45	848.111	848.078				848.09	0.02
60	848.117	848.066				848.09	0.04
300	848.151	848.069				848.11	0.06
1500	848.169	848.132				848.15	0.03
3600	848.08	848.187				848.13	0.08
7200	848.307	848.224				848.27	0.06
14400	848.421	848.31				848.37	0.08

ONPF

Time	Replicate 1	Replicate 2	Replicate 3	Replicate 4	average	SD
0	847.941	847.968			847.95	0.02
30	848.04	848.06			848.05	0.01
45	848.106	848.084			848.10	0.02
60	848.083	848.109			848.10	0.02
300	848.18	848.123			848.15	0.04
1500	848.229	848.224			848.23	0.00
3600	848.294	848.268			848.28	0.02
7200	848.368	848.322			848.35	0.03
14400	848.432	848.172			848.30	0.18

APO

Time	Replicate 1	Replicate 2	Replicate 3	Replicate 4	Replicate 5	average	SD
0	847.958	847.955				847.96	0.00
30	848.07	848.056				848.06	0.01
45	848.054	848.065				848.06	0.01
60	848.093	848.093				848.09	0.00
300	848.113	848.093				848.10	0.01
1500	848.221	848.226				848.22	0.00
3600	848.4	848.328				848.36	0.05
7200	848.47	848.345				848.41	0.09
14400	848.607	848.41				848.51	0.14

DNA

Time	Replicate 1	Replicate 2	Replicate 3	Replicate 4	Replicate 5	average	SD
0	847.983	847.949				847.97	0.02
30	848.081	848.046				848.06	0.02
45	848.111	848.06				848.09	0.04
60	848.089	848.112				848.10	0.02
300	848.054	848.119				848.09	0.05
1500	848.176	848.233				848.20	0.04
3600	848.286	848.225				848.26	0.04
7200	848.346	848.244				848.30	0.07
14400	848.41	848.056				848.23	0.25

ONPFDNA

Time	Replicate 1	Replicate 2	Replicate 3	Replicate 4	average	SD
0	847.932	847.958			847.95	0.02
30	848.037	848.043			848.04	0.00
45	848.04	848.075			848.06	0.02
60	848.086	848.142			848.11	0.04
300	848.074	848.099			848.09	0.02
1500	848.139	848.226			848.18	0.06
3600	848.304	848.354			848.33	0.04
7200	848.356	848.34			848.35	0.01
14400	848.216	848.261			848.24	0.03

TMG

Time	Replicate 1	Replicate 2	Replicate 3	Replicate 4	Replicate 5	average	SD
0	848.009	847.945				847.98	0.05
30	848.085	848.134				848.11	0.03
45	848.12	848.102				848.11	0.01
60	848.108					848.11	
300	848.131	848.131				848.13	0.00
1500	848.084	848.172				848.13	0.06
3600	848.2	848.196				848.20	0.00
7200	848.252	848.295				848.27	0.03
14400	848.395	848.394				848.39	0.00

128-135 LDDQDAIA

Charge 1

IPTG

Time	centroid	D
0	860.89	0.04
30	861.7285	1.66
45	861.852	1.90
60	861.8565	1.90
300	861.801	1.80
1500	861.882	1.95
3600	862.0635	2.30
7200	862.224	2.61
14400	862.3005	2.76

ONPF

Time	centroid	D
0	860.92	0.09
30	861.50	1.22
45	861.71	1.63
60	861.73	1.66
300	861.68	1.56
1500	861.79	1.77
3600	861.91	2.01
7200	861.92	2.03
14400	862.40	2.95

APO

Time	centroid	D
0	860.869	0.00
30	861.67	1.54
45	861.711	1.62
60	861.84	1.87
300	861.883	1.96
1500	861.935	2.06
3600	862.042	2.26
7200	862.133	2.44
14400	862.404	2.96

DNA

Time	centroid	D
0	860.8615	-0.01
30	861.5845	1.38
45	861.766	1.73
60	861.814	1.82
300	861.7705	1.74
1500	861.815	1.82
3600	861.872	1.93
7200	861.8635	1.92
14400	861.837	1.87

ONPFDNA

Time	centroid	D
0	860.868	0.00
30	861.566	1.34
45	861.7505	1.70
60	861.7005	1.60
300	861.725	1.65
1500	861.6295	1.47
3600	861.89	1.97
7200	861.877	1.94
14400	861.6275	1.46

TMG

Time	centroid	D
0	860.88	0.03
30	861.77	1.73
45	861.90	1.98
60	861.84	1.87
300	861.93	2.04
1500	861.92	2.03
3600	862.14	2.45
7200	862.23	2.62
14400	862.45	3.05

control 860.869
infinity 863.98

$$D(t) = \frac{Mt - M0}{M\infty - M0} *N$$

IPTG

Time	Replicate 1	Replicate 2	Replicate 3	Replicate 4	Replicate5	average	SD
0	860.925	860.855				860.89	0.05
30	861.918	861.539				861.73	0.27
45	861.963	861.741				861.85	0.16
60	861.978	861.735				861.86	0.17
300	861.957	861.645				861.80	0.22
1500	862.07	861.694				861.88	0.27
3600	862.176	861.951				862.06	0.16
7200	862.414	862.034				862.22	0.27
14400	862.516	862.085				862.30	0.30

ONPF

Time	Replicate 1	Replicate 2	Replicate 3	Replicate 4	average	SD
0	860.913	860.919			860.92	0.00
30	861.629	861.37			861.50	0.18
45	861.969	861.458			861.71	0.36
60	861.806	861.657			861.73	0.11
300	861.852	861.506			861.68	0.24
1500	861.931	861.647			861.79	0.20
3600	862.101	861.719			861.91	0.27
7200	862.058	861.781			861.92	0.20
14400	862.398				862.40	

APO

Time	Replicate 1	Replicate 2	Replicate 3	Replicate 4	Replicate 5	average	SD
0	860.868	860.87				860.87	0.00
30	861.834	861.506				861.67	0.23
45	861.897	861.525				861.71	0.26
60	861.995	861.685				861.84	0.22
300	861.883					861.88	
1500	861.994	861.876				861.94	0.08
3600	862.186	861.898				862.04	0.20
7200	862.329	861.937				862.13	0.28
14400	862.562	862.246				862.40	0.22

DNA

Time	Replicate 1	Replicate 2	Replicate 3	Replicate 4	Replicate 5	average	SD
0	860.86	860.863				860.86	0.00
30	861.642	861.527				861.58	0.08
45	861.922	861.61				861.77	0.22
60	861.967	861.661				861.81	0.22
300	861.896	861.645				861.77	0.18
1500	861.91	861.72				861.82	0.13
3600	862.065	861.679				861.87	0.27
7200	862.074	861.653				861.86	0.30
14400	862.223	861.451				861.84	0.55

ONPFDNA

Time	Replicate 1	Replicate 2	Replicate 3	Replicate 4	average	SD
0	860.874	860.862			860.87	0.01
30	861.593	861.539			861.57	0.04
45	861.828	861.673			861.75	0.11
60	861.756	861.645			861.70	0.08
300	861.838	861.612			861.73	0.16
1500	861.53	861.729			861.63	0.14
3600	861.961	861.819			861.89	0.10
7200	861.947	861.807			861.88	0.10
14400	861.516	861.739			861.63	0.16

TMG

Time	Replicate 1	Replicate 2	Replicate 3	Replicate 4	Replicate 5	average	SD
0	860.902	860.865				860.88	0.03
30	861.574	861.963				861.77	0.28
45	861.871	861.924				861.90	0.04
60	861.891	861.785				861.84	0.07
300	861.858	862				861.93	0.10
1500	861.796	862.044				861.92	0.18
3600	862.071	862.212				862.14	0.10
7200	862.099	862.361				862.23	0.19
14400	862.394	862.507				862.45	0.08

134-139 IAVEAA

Charge 1

IPTG

Time	centroid	D
0	573.63	0.01
30	573.76	0.14
45	573.77	0.15
60	573.80	0.18
300	573.88	0.26
1500	574.17	0.55
3600	574.35	0.73
7200	574.74	1.12
14400	574.70	1.09

ONPF

Time	centroid	D
0	573.64	0.02
30	573.74	0.12
45	573.95	0.33
60	573.85	0.23
300	573.84	0.22
1500	574.05	0.43
3600	574.17	0.55
7200	574.16	0.54
14400	574.16	0.54

APO

Time	centroid	D
0	573.62	0.00
30	573.71	0.09
45	573.74	0.12
60	573.86	0.24
300	573.84	0.22
1500	574.17	0.56
3600	574.29	0.68
7200	574.38	0.77
14400	574.56	0.95

DNA

Time	centroid	D
0	573.61	-0.01
30	573.77	0.16
45	573.98	0.36
60	573.83	0.22
300	573.82	0.20
1500	573.93	0.31
3600	573.97	0.35
7200	574.05	0.43
14400	574.26	0.64

ONPFDNA

Time	centroid	D
0	573.62	0.00
30	573.70	0.08
45	573.77	0.15
60	573.83	0.21
300	573.79	0.17
1500	573.93	0.31
3600	574.06	0.44
7200	574.06	0.44
14400	574.03	0.42

TMG

Time	centroid	D
0	573.63	0.01
30	573.76	0.14
45	573.88	0.26
60	573.86	0.24
300	573.89	0.27
1500	574.12	0.50
3600	574.38	0.77
7200	574.42	0.81
14400	574.69	1.08

control 573.62
infinity 577.591

$$D(t) = \frac{M_t - M_0}{M_\infty - M_0} * N$$

IPTG

Time	Replicate 1	Replicate 2	Replicate 3	Replicate 4	Replicate5	average	SD
0	573.632	573.621				573.63	0.01
30	573.786	573.727				573.76	0.04
45	573.787	573.747				573.77	0.03
60	573.785	573.813				573.80	0.02
300	573.895	573.859				573.88	0.03
1500	574.223	574.109				574.17	0.08
3600	574.404	574.294				574.35	0.08
7200	574.6	574.871				574.74	0.19
14400	574.912	574.484				574.70	0.30

ONPF

Time	Replicate 1	Replicate 2	Replicate 3	Replicate 4	average	SD
0	573.644	573.628			573.64	0.01
30	573.784	573.69			573.74	0.07
45	573.894	573.998			573.95	0.07
60	573.856	573.842			573.85	0.01
300	573.887	573.789			573.84	0.07
1500	574.102	573.992			574.05	0.08
3600	574.301	574.033			574.17	0.19
7200	574.197	574.119			574.16	0.06
14400	574.626	573.692			574.16	0.66

APO

Time	Replicate 1	Replicate 2	Replicate 3	Replicate 4	Replicate 5	average	SD
0	573.625	573.616				573.62	0.01
30	573.723	573.699				573.71	0.02
45	573.753	573.723				573.74	0.02
60	573.753	573.963				573.86	0.15
300	573.869	573.804				573.84	0.05
1500	574.125	574.219				574.17	0.07
3600	574.381	574.204				574.29	0.13
7200	574.532	574.235				574.38	0.21
14400	574.663	574.457				574.56	0.15

DNA

Time	Replicate 1	Replicate 2	Replicate 3	Replicate 4	Replicate 5	average	SD
0	573.611	573.604				573.61	0.00
30	573.774					573.77	
45	574.197	573.754				573.98	0.31
60	573.827	573.841				573.83	0.01
300	573.858	573.772				573.82	0.06
1500	573.853	574.005				573.93	0.11
3600	574.007	573.926				573.97	0.06
7200	574.096	574.001				574.05	0.07
14400	574.335	574.179				574.26	0.11

ONPFDNA

Time	Replicate 1	Replicate 2	Replicate 3	Replicate 4	average	SD
0	573.616	573.622			573.62	0.00
30	573.714	573.693			573.70	0.01
45	573.824	573.712			573.77	0.08
60	573.806	573.857			573.83	0.04
300	573.803	573.782			573.79	0.01
1500	573.833	574.023			573.93	0.13
3600	574.032	574.086			574.06	0.04
7200	574.02	574.102			574.06	0.06
14400	573.994	574.071			574.03	0.05

TMG

Time	Replicate 1	Replicate 2	Replicate 3	Replicate 4	Replicate 5	average	SD
0	573.635	573.634				573.63	0.00
30	573.739	573.779				573.76	0.03
45	573.953	573.801				573.88	0.11
60	573.861	573.864				573.86	0.00
300	573.872	573.907				573.89	0.02
1500	574.101	574.133				574.12	0.02
3600	574.394	574.368				574.38	0.02
7200	574.337	574.509				574.42	0.12
14400	574.661	574.714				574.69	0.04

139-146 ACTNVPAL

Charge 1

IPTG

Time	centroid	D
0	788.912	0.02
30	790.1225	1.79
45	790.226	1.94
60	790.327	2.09
300	790.4145	2.21
1500	790.5	2.34
3600	790.7925	2.77
7200	790.7875	2.76
14400	790.6875	2.61

ONPF

Time	centroid	D
0	788.9965	0.14
30	789.5825	1.00
45	789.971	1.57
60	790.1425	1.82
300	790.1555	1.84
1500	790.3315	2.09
3600	790.251	1.98
7200	790.255	1.98
14400	790.68	2.60

APO

Time	centroid	D
0	788.901	0.00
30	789.9475	1.53
45	790.0405	1.67
60	790.325	2.08
300	790.2835	2.02
1500	790.5545	2.42
3600	790.741	2.69
7200	790.481	2.31
14400	790.715	2.65

DNA

Time	centroid	D
0	788.907	0.01
30	789.774	1.28
45	790.1375	1.81
60	790.21	1.92
300	790.3365	2.10
1500	790.541	2.40
3600	790.5125	2.36
7200	790.3055	2.06
14400	790.337	2.10

ONPFDNA

Time	centroid	D
0	788.907	0.01
30	789.7045	1.18
45	790.0915	1.74
60	790.1665	1.85
300	790.208	1.91
1500	790.193	1.89
3600	790.629	2.53
7200	790.3325	2.09
14400	790.16	1.84

TMG

Time	centroid	D
0	788.9155	0.02
30	790.02	1.64
45	790.2085	1.91
60	790.202	1.90
300	790.573	2.45
1500	790.585	2.46
3600	790.707	2.64
7200	790.6715	2.59
14400	790.873	2.89

control 788.901
infinity 792.318

$$D(t) = \frac{M_t - M_0}{M_\infty - M_0} * N$$

IPTG

Time	Replicate 1	Replicate 2	Replicate 3	Replicate 4	Replicate5	average	SD
0	788.892	788.932				788.91	0.03
30	790.347	789.898				790.12	0.32
45	790.214	790.238				790.23	0.02
60	790.322	790.332				790.33	0.01
300	790.542	790.287				790.41	0.18
1500	790.678	790.322				790.50	0.25
3600	790.856	790.729				790.79	0.09
7200	790.985	790.59				790.79	0.28
14400	790.884	790.491				790.69	0.28

ONPF

Time	Replicate 1	Replicate 2	Replicate 3	Replicate 4	average	SD
0	788.993	789			789.00	0.00
30	789.631	789.534			789.58	0.07
45	790.215	789.727			789.97	0.35
60	790.174	790.111			790.14	0.04
300	790.364	789.947			790.16	0.29
1500	790.508	790.155			790.33	0.25
3600	790.453	790.049			790.25	0.29
7200	790.357	790.153			790.26	0.14
14400	790.675				790.68	

APO

Time	Replicate 1	Replicate 2	Replicate 3	Replicate 4	Replicate 5	average	SD
0	788.875	788.927				788.90	0.04
30	790.14	789.755				789.95	0.27
45	790.255	789.826				790.04	0.30
60	790.434	790.216				790.33	0.15
300	790.573	789.994				790.28	0.41
1500	790.654	790.455				790.55	0.14
3600	790.825	790.657				790.74	0.12
7200	790.754	790.208				790.48	0.39
14400	790.9	790.53				790.72	0.26

DNA

Time	Replicate 1	Replicate 2	Replicate 3	Replicate 4	Replicate 5	average	SD
0	788.876	788.938				788.91	0.04
30	789.775	789.773				789.77	0.00
45	790.273	790.002				790.14	0.19
60	790.215	790.205				790.21	0.01
300	790.375	790.298				790.34	0.05
1500	790.561	790.521				790.54	0.03
3600	790.676	790.349				790.51	0.23
7200	790.553	790.058				790.31	0.35
14400	790.774	789.9				790.34	0.62

ONPFDNA

Time	Replicate 1	Replicate 2	Replicate 3	Replicate 4	average	SD
0	788.901	788.913			788.91	0.01
30	789.654	789.755			789.70	0.07
45	790.089	790.094			790.09	0.00
60	790.137	790.196			790.17	0.04
300	790.208	790.208			790.21	0.00
1500	790.166	790.22			790.19	0.04
3600	790.645	790.613			790.63	0.02
7200	790.356	790.309			790.33	0.03
14400	790.215	790.105			790.16	0.08

TMG

Time	Replicate 1	Replicate 2	Replicate 3	Replicate 4	Replicate 5	average	SD
0	788.952	788.879				788.92	0.05
30	789.723	790.317				790.02	0.42
45	790.217	790.2				790.21	0.01
60	790.277	790.127				790.20	0.11
300	790.486	790.66				790.57	0.12
1500	790.387	790.783				790.59	0.28
3600	790.562	790.852				790.71	0.21
7200	790.364	790.979				790.67	0.43
14400	790.692	791.054				790.87	0.26

145-149 ALFLD

Charge 1

IPTG

Time	centroid	D
0	578.692	0.02
30	578.8005	0.20
45	578.8225	0.23
60	578.8655	0.30
300	578.813	0.22
1500	578.8455	0.27
3600	578.8535	0.28
7200	578.872	0.31
14400	578.878	0.32

ONPF

Time	centroid	D
0	578.704	0.04
30	578.807	0.21
45	578.813	0.22
60	578.819	0.23
300	578.766	0.14
1500	578.839	0.26
3600	578.824	0.23
7200	578.893	0.35
14400	579.03	0.56

APO

Time	centroid	D
0	578.675	-0.01
30	578.787	0.17
45	578.8055	0.20
60	578.797	0.19
300	578.8025	0.20
1500	578.815	0.22
3600	578.8355	0.25
7200	578.848	0.27
14400	578.9625	0.46

DNA

Time	centroid	D
0	578.667	-0.02
30	578.8005	0.20
45	578.8745	0.32
60	578.8385	0.26
300	578.838	0.26
1500	578.8715	0.31
3600	578.853	0.28
7200	578.826	0.24
14400	578.8545	0.28

ONPFDNA

Time	centroid	D
0	578.686	0.01
30	578.8195	0.23
45	578.887	0.34
60	578.841	0.26
300	578.9315	0.41
1500	578.833	0.25
3600	578.847	0.27
7200	578.852	0.28
14400	578.836	0.25

TMG

Time	centroid	D
0	578.7005	0.03
30	578.824	0.23
45	578.8945	0.35
60	578.9155	0.38
300	578.8985	0.36
1500	578.913	0.38
3600	578.8685	0.31
7200	578.924	0.40
14400	578.909	0.37

control 578.68
infinity 580.526

$$D(t) = \frac{M_t - M_0}{M_\infty - M_0} * N$$

IPTG

Time	Replicate 1	Replicate 2	Replicate 3	Replicate 4	Replicate5	average	SD
0	578.708	578.676				578.69	0.02
30	578.824	578.777				578.80	0.03
45	578.86	578.785				578.82	0.05
60	578.905	578.826				578.87	0.06
300	578.851	578.775				578.81	0.05
1500	578.87	578.821				578.85	0.03
3600	578.92	578.787				578.85	0.09
7200	578.923	578.821				578.87	0.07
14400	578.952	578.804				578.88	0.10

ONPF

Time	Replicate 1	Replicate 2	Replicate 3	Replicate 4	average	SD
0	578.712	578.696			578.70	0.01
30	578.845	578.769			578.81	0.05
45	578.844	578.782			578.81	0.04
60	578.833	578.805			578.82	0.02
300	578.779	578.753			578.77	0.02
1500	578.857	578.821			578.84	0.03
3600	578.837	578.811			578.82	0.02
7200	578.999	578.787			578.89	0.15
14400	579.025				579.03	

APO

Time	Replicate 1	Replicate 2	Replicate 3	Replicate 4	Replicate 5	average	SD
0	578.676	578.674				578.68	0.00
30	578.8	578.774				578.79	0.02
45	578.823	578.788				578.81	0.02
60	578.789	578.805				578.80	0.01
300	578.805	578.8				578.80	0.00
1500	578.817	578.813				578.82	0.00
3600	578.836	578.835				578.84	0.00
7200	578.879	578.817				578.85	0.04
14400	579.039	578.886				578.96	0.11

DNA

Time	Replicate 1	Replicate 2	Replicate 3	Replicate 4	Replicate 5	average	SD
0	578.668	578.666				578.67	0.00
30	578.844	578.757				578.80	0.06
45	578.967	578.782				578.87	0.13
60	578.868	578.809				578.84	0.04
300	578.888	578.788				578.84	0.07
1500	578.913	578.83				578.87	0.06
3600	578.91	578.796				578.85	0.08
7200	578.831	578.821				578.83	0.01
14400	578.883	578.826				578.85	0.04

ONPFDNA

Time	Replicate 1	Replicate 2	Replicate 3	Replicate 4	average	SD
0	578.695	578.677			578.69	0.01
30	578.854	578.785			578.82	0.05
45		578.887			578.89	
60	578.899	578.783			578.84	0.08
300	579.086	578.777			578.93	0.22
1500	578.868	578.798			578.83	0.05
3600	578.923	578.771			578.85	0.11
7200	578.923	578.781			578.85	0.10
14400	578.935	578.737			578.84	0.14

TMG

Time	Replicate 1	Replicate 2	Replicate 3	Replicate 4	Replicate 5	average	SD
0	578.724	578.677				578.70	0.03
30	578.829	578.819				578.82	0.01
45	578.973	578.816				578.89	0.11
60	578.885	578.946				578.92	0.04
300	578.921	578.876				578.90	0.03
1500	578.919	578.907				578.91	0.01
3600	578.904	578.833				578.87	0.05
7200	578.977	578.871				578.92	0.07
14400	578.839	578.979				578.91	0.10

147-158 FLDVSDQTPINS

Charge 2

IPTG

Time	centroid	D
0	668.7248	-0.02
30	669.2168	2.08
45	669.435	3.01
60	669.4768	3.19
300	669.593	3.69
1500	669.8428	4.76
3600	669.8855	4.94
7200	670.0165	5.50
14400	670.032	5.57

ONPF

Time	centroid	D
0	668.72	-0.04
30	669.05	1.38
45	669.27	2.29
60	669.46	3.10
300	669.46	3.12
1500	669.81	4.62
3600	669.83	4.68
7200	669.84	4.73
14400	669.94	5.18

APO

Time	centroid	D
0	668.7327	0.01
30	669.2023	2.02
45		
60	669.4883	3.24
300	669.632	3.86
1500	669.969	5.30
3600	669.9667	5.29
7200	670.0923	5.82
14400	670.1827	6.21

DNA

Time	centroid	D
0	668.72	-0.04
30	669.18	1.91
45		
60	669.61	3.76
300	669.67	4.02
1500	669.88	4.92
3600	669.93	5.11
7200	670.00	5.43
14400	670.02	5.51

ONPFDNA

Time	centroid	D
0	668.72	-0.05
30	669.11	1.61
45	669.50	3.28
60	669.51	3.33
300	669.68	4.06
1500	669.68	4.08
3600	669.96	5.25
7200	669.90	5.01
14400	669.81	4.61

TMG

Time	centroid	D
0	668.72	-0.04
30	669.24	2.17
45	669.43	3.00
60	669.62	3.78
300	669.71	4.21
1500	669.93	5.15
3600	670.10	5.84
7200	670.20	6.30
14400	670.50	7.55

control 668.73
infinity 670.835

$$D(t) = \frac{M_t - M_0}{M_\infty - M_0} * N$$

IPTG

Time	Replicate 1	Replicate 2	Replicate 3	Replicate 4	Replicate5	average	SD
0	668.74	668.726	668.716	668.717		668.72	0.01
30	669.406	669.225	669.124	669.112		669.22	0.14
45	669.435					669.44	
60	669.503	669.642	669.384	669.378		669.48	0.12
300	669.682	669.695	669.57	669.425		669.59	0.13
1500	670.133	669.815	669.77	669.653		669.84	0.21
3600	670.208	669.839	669.808	669.687		669.89	0.22
7200	670.392	669.935	669.918	669.821		670.02	0.26
14400	670.325	669.969	670.027	669.807		670.03	0.22

ONPF

Time	Replicate 1	Replicate 2	Replicate 3	Replicate 4	average	SD
0	668.767	668.671	668.723	668.717	668.72	0.04
30	669.097	668.879	669.178	669.055	669.05	0.13
45	669.544	668.988			669.27	0.39
60	669.443	669.256	669.704	669.421	669.46	0.19
300	669.662	669.259	669.485	669.435	669.46	0.17
1500	670.063	669.532	669.878	669.772	669.81	0.22
3600	670.044	669.491	670.003	669.764	669.83	0.25
7200	670.195	669.396	669.942	669.813	669.84	0.33
14400	670.434	669.19	670.066	670.074	669.94	0.53

APO

Time	Replicate 1	Replicate 2	Replicate 3	Replicate 4	Replicate 5	average	SD
0	668.775	668.707	668.716			668.73	0.04
30	669.324	669.167	669.116			669.20	0.11
45							
60	669.645	669.41	669.41			669.49	0.14
300	669.688	669.659	669.549			669.63	0.07
1500	669.948	670.144	669.815			669.97	0.17
3600	670.079	669.97	669.851			669.97	0.11
7200	670.245	670.097	669.935			670.09	0.16
14400	670.171	670.178	670.199			670.18	0.01

DNA

Time	Replicate 1	Replicate 2	Replicate 3	Replicate 4	Replicate 5	average	SD
0	668.723	668.722	668.72			668.72	0.00
30	669.244	669.17	669.115			669.18	0.06
45							
60	669.542	669.801	669.483			669.61	0.17
300	669.767	669.72	669.522			669.67	0.13
1500	670.03	669.935	669.675			669.88	0.18
3600	670.109	669.945	669.722			669.93	0.19
7200	670.15	670.135	669.712			670.00	0.25
14400	670.144	670.096	669.814			670.02	0.18

ONPFDNA

Time	Replicate 1	Replicate 2	Replicate 3	Replicate 4	average	SD
0	668.722	668.713			668.72	0.01
30	669.193	669.02			669.11	0.12
45	669.496				669.50	
60	669.525	669.494			669.51	0.02
300	669.75	669.61			669.68	0.10
1500	669.624	669.745			669.68	0.09
3600	670.078	669.838			669.96	0.17
7200	669.951	669.851			669.90	0.07
14400	669.719	669.896			669.81	0.13

TMG

Time	Replicate 1	Replicate 2	Replicate 3	Replicate 4	Replicate 5	average	SD
0	668.738	668.704				668.72	0.02
30	669.131	669.342				669.24	0.15
45	669.489	669.375				669.43	0.08
60	669.427	669.803				669.62	0.27
300	669.701	669.727				669.71	0.02
1500	669.79	670.078				669.93	0.20
3600	669.976	670.214				670.10	0.17
7200	670.04	670.369				670.20	0.23
14400	670.423	670.571				670.50	0.10

152-157 DQTPIN

Charge 1

IPTG

Time	centroid	D
0	687.698	-0.01
30	688.054	0.46
45	688.1125	0.54
60	688.1975	0.65
300	688.6055	1.19
1500	688.8225	1.47
3600	689.0975	1.83
7200	688.942	1.63
14400	688.8225	1.47

ONPF

Time	centroid	D
0	687.7565	0.07
30	687.955	0.33
45	688.125	0.55
60	688.272	0.75
300	688.5025	1.05
1500	688.6785	1.28
3600	688.6785	1.28
7200	688.6445	1.24
14400	689.00	1.70

APO

Time	centroid	D
0	687.705	0.00
30	688.026	0.42
45	688.073	0.48
60	688.317	0.81
300	688.5865	1.16
1500	688.884	1.55
3600	688.9345	1.62
7200	688.8825	1.55
14400	689.054	1.78

DNA

Time	centroid	D
0	687.7015	0.00
30	688.0265	0.42
45	688.2465	0.71
60	688.3415	0.84
300	688.6115	1.19
1500	688.762	1.39
3600	688.7375	1.36
7200	688.6	1.18
14400	688.546	1.11

ONPFDNA

Time	centroid	D
0	687.712	0.01
30	687.969	0.35
45	688.1585	0.60
60	688.276	0.75
300	688.546	1.11
1500	688.5	1.05
3600	688.81	1.45
7200	688.6845	1.29
14400	688.4395	0.97

TMG

Time	centroid	D
0	687.709	0.01
30	688.068	0.48
45	688.1525	0.59
60	688.3755	0.88
300	688.69	1.30
1500	688.884	1.55
3600	688.9775	1.68
7200	688.899	1.57
14400	689.0735	1.80

control	687.705
infinity	689.984

$$D(t) = \frac{M_t - M_0}{M_\infty - M_0} *N$$

IPTG

Time	Replicate 1	Replicate 2	Replicate 3	Replicate 4	Replicate5	average	SD
0	687.718	687.678				687.70	0.03
30	688.17	687.938				688.05	0.16
45	688.186	688.039				688.11	0.10
60	688.257	688.138				688.20	0.08
300	688.665	688.546				688.61	0.08
1500	689.066	688.579				688.82	0.34
3600	689.224	688.971				689.10	0.18
7200	689.08	688.804				688.94	0.20
14400	688.965	688.68				688.82	0.20

ONPF

Time	Replicate 1	Replicate 2	Replicate 3	Replicate 4	average	SD
0	687.723	687.79			687.76	0.05
30	687.986	687.924			687.96	0.04
45	688.249	688.001			688.13	0.18
60	688.252	688.292			688.27	0.03
300	688.677	688.328			688.50	0.25
1500	688.86	688.497			688.68	0.26
3600	688.81	688.547			688.68	0.19
7200	688.752	688.537			688.64	0.15
14400	688.996				689.00	

APO

Time	Replicate 1	Replicate 2	Replicate 3	Replicate 4	Replicate 5	average	SD
0	687.7	687.71				687.71	0.01
30	688.041	688.011				688.03	0.02
45	688.096	688.05				688.07	0.03
60	688.267	688.367				688.32	0.07
300	688.77	688.403				688.59	0.26
1500	688.896	688.872				688.88	0.02
3600	689.043	688.826				688.93	0.15
7200	689.05	688.715				688.88	0.24
14400	689.185	688.923				689.05	0.19

DNA

Time	Replicate 1	Replicate 2	Replicate 3	Replicate 4	Replicate 5	average	SD
0	687.685	687.718				687.70	0.02
30	688.04	688.013				688.03	0.02
45	688.405	688.088				688.25	0.22
60	688.33	688.353				688.34	0.02
300	688.651	688.572				688.61	0.06
1500	688.734	688.79				688.76	0.04
3600	688.862	688.613				688.74	0.18
7200	688.738	688.462				688.60	0.20
14400	688.879	688.213				688.55	0.47

ONPFDNA

Time	Replicate 1	Replicate 2	Replicate 3	Replicate 4	average	SD
0	687.693	687.731			687.71	0.03
30	688.004	687.934			687.97	0.05
45	688.213	688.104			688.16	0.08
60	688.257	688.295			688.28	0.03
300	688.63	688.462			688.55	0.12
1500	688.463	688.537			688.50	0.05
3600	688.803	688.817			688.81	0.01
7200	688.723	688.646			688.68	0.05
14400	688.466	688.413			688.44	0.04

TMG

Time	Replicate 1	Replicate 2	Replicate 3	Replicate 4	Replicate 5	average	SD
0	687.72	687.698				687.71	0.02
30	688.019	688.117				688.07	0.07
45	688.248	688.057				688.15	0.14
60	688.224	688.527				688.38	0.21
300	688.678	688.702				688.69	0.02
1500	688.727	689.041				688.88	0.22
3600	688.878	689.077				688.98	0.14
7200	688.714	689.084				688.90	0.26
14400	689.006	689.141				689.07	0.10

158-163 SIIFSH

Charge 1

IPTG

Time	centroid	D
0	703.80	-0.10
30	703.98	0.21
45	704.00	0.24
60	704.03	0.30
300	704.00	0.24
1500	704.02	0.28
3600	704.01	0.25
7200	704.02	0.28
14400	704.09	0.39

ONPF

Time	centroid	D
0	703.83	-0.05
30	703.88	0.04
45	704.13	0.45
60	704.10	0.40
300	704.09	0.40
1500	704.04	0.31
3600	704.33	0.78
7200	704.19	0.55
14400	704.63	1.28

APO

Time	centroid	D
0	703.85	0.00
30	703.97	0.19
45	704.00	0.23
60	704.05	0.33
300	704.06	0.35
1500	704.13	0.46
3600	704.21	0.59
7200	704.33	0.78
14400	704.68	1.36

DNA

Time	centroid	D
0	703.93	0.12
30	703.97	0.19
45	704.00	0.24
60	704.05	0.32
300	704.07	0.36
1500	704.11	0.42
3600	704.18	0.54
7200	704.15	0.49
14400	704.21	0.60

ONPFDNA

Time	centroid	D
0	704.02	0.27
30	704.14	0.47
45	704.12	0.44
60	704.11	0.43
300	704.21	0.59
1500	704.21	0.58
3600	704.19	0.55
7200	704.30	0.73
14400	704.30	0.73

TMG

Time	centroid	D
0	703.97	0.20
30	704.13	0.45
45	704.11	0.42
60	704.25	0.65
300	704.10	0.41
1500	704.15	0.49
3600	704.13	0.46
7200	704.17	0.52
14400	704.30	0.74

control	703.854
infinity	706.272

$$D(t) = \frac{M_t - M_0}{M_\infty - M_0} \cdot N$$

IPTG

Time	Replicate 1	Replicate 2	Replicate 3	Replicate 4	Replicate5	average	SD
0	703.794	703.798				703.80	0.00
30	704.076	703.889				703.98	0.13
45	704.064	703.93				704.00	0.09
60	704.136	703.932				704.03	0.14
300	704.082	703.922				704.00	0.11
1500	704.078	703.963				704.02	0.08
3600	704.062	703.952				704.01	0.08
7200	704.103	703.94				704.02	0.12
14400	704.179	704.005				704.09	0.12

ONPF

Time	Replicate 1	Replicate 2	Replicate 3	Replicate 4	average	SD
0	703.826				703.83	
30	703.88				703.88	
45	704.125				704.13	
60	704.095				704.10	
300	704.094				704.09	
1500	704.039				704.04	
3600	704.328				704.33	
7200	704.188				704.19	
14400	704.626				704.63	

APO

Time	Replicate 1	Replicate 2	Replicate 3	Replicate 4	Replicate 5	average	SD
0	703.815	703.893				703.85	0.06
30	704.009	703.923				703.97	0.06
45	704.067	703.925				704.00	0.10
60	704.105	704.003				704.05	0.07
300	704.063					704.06	
1500	704.127	704.135				704.13	0.01
3600	704.284	704.135				704.21	0.11
7200	704.467	704.184				704.33	0.20
14400	704.784	704.569				704.68	0.15

DNA

Time	Replicate 1	Replicate 2	Replicate 3	Replicate 4	Replicate 5	average	SD
0	703.953	703.906				703.93	0.03
30	704.049	703.886				703.97	0.12
45	704.115	703.887				704.00	0.16
60	704.136	703.962				704.05	0.12
300	704.072	704.075				704.07	0.00
1500	704.217	703.997				704.11	0.16
3600	704.294	704.061				704.18	0.16
7200	704.181	704.123				704.15	0.04
14400	704.42	704.009				704.21	0.29

ONPFDNA

Time	Replicate 1	Replicate 2	Replicate 3	Replicate 4	average	SD
0	704.135	703.902			704.02	0.16
30	704.247	704.028			704.14	0.15
45	704.323	703.922			704.12	0.28
60	704.263	703.966			704.11	0.21
300	704.369	704.058			704.21	0.22
1500	704.36	704.055			704.21	0.22
3600	704.423	703.95			704.19	0.33
7200	704.511	704.082			704.30	0.30
14400	704.295				704.30	

TMG

Time	Replicate 1	Replicate 2	Replicate 3	Replicate 4	Replicate 5	average	SD
0	704.104	703.841				703.97	0.19
30	704.104	704.146				704.13	0.03
45	704.106	704.106				704.11	0.00
60	704.061	704.435				704.25	0.26
300	704.049	704.149				704.10	0.07
1500	704.067	704.228				704.15	0.11
3600	704.1	704.159				704.13	0.04
7200	704.157	704.182				704.17	0.02
14400	704.258	704.345				704.30	0.06

158-169 SIIFSHEDGTRL

Charge 3

IPTG

Time	centroid	D
0	459.1918	0.26
30	459.2418	0.67
45	459.269	0.89
60	459.263	0.84
300	459.277	0.95
1500	459.2662	0.87
3600	459.2762	0.95
7200	459.297	1.12
14400	459.3056	1.19

ONPF

Time	centroid	D
0	459.16	-0.02
30	459.23	0.61
45	459.23	0.55
60	459.24	0.65
300	459.24	0.67
1500	459.28	0.95
3600	459.31	1.22
7200	459.33	1.39
14400	459.39	1.89

APO

Time	centroid	D
0	459.1638	0.03
30	459.216	0.46
45	459.252	0.75
60	459.2683	0.88
300	459.285	1.02
1500	459.3224	1.32
3600	459.35	1.55
7200	459.4662	2.50
14400	459.5616	3.28

DNA

Time	centroid	D
0	459.158	-0.02
30	459.2117	0.42
45	459.2435	0.68
60	459.234	0.60
300	459.2263	0.54
1500	459.2443	0.69
3600	459.256	0.78
7200	459.2593	0.81
14400	459.2773	0.96

ONPFDNA

Time	centroid	D
0	459.1607	0.01
30	459.204	0.36
45	459.229	0.56
60	459.2337	0.60
300	459.2323	0.59
1500	459.224	0.52
3600	459.252	0.75
7200	459.2647	0.85
14400	459.2373	0.63

TMG

Time	centroid	D
0	459.16	0.04
30	459.18	0.13
45	459.29	1.04
60	459.44	2.24
300	459.26	0.84
1500	459.30	1.13
3600	459.31	1.22
7200	459.27	0.91
14400	459.36	1.62

control 459.16
infinity 460.386

$$D(t) = \frac{M_t - M_0}{M_\infty - M_0} * N$$

IPTG

Time	Replicate 1	Replicate 2	Replicate 3	Replicate 4	Replicate5	average	SD
0	459.241	459.209	459.168	459.163	459.178	459.19	0.03
30	459.213	459.3	459.215	459.203	459.278	459.24	0.04
45		459.253			459.285	459.27	0.02
60		459.268	459.243	459.23	459.311	459.26	0.04
300	459.394	459.21	459.233	459.232	459.316	459.28	0.08
1500	459.369	459.217	459.23	459.227	459.288	459.27	0.06
3600	459.386	459.263	459.233	459.243	459.256	459.28	0.06
7200	459.43	459.259	459.258	459.248	459.29	459.30	0.08
14400	459.439	459.248	459.26	459.256	459.325	459.31	0.08

ONPF

Time	Replicate 1	Replicate 2	Replicate 3	Replicate 4	average	SD
0	459.169	459.165	459.138	459.159	459.16	0.01
30	459.222	459.185	459.28	459.251	459.23	0.04
45	459.227				459.23	
60	459.203	459.211	459.268	459.278	459.24	0.04
300	459.249	459.213	459.215	459.292	459.24	0.04
1500	459.274	459.258	459.265	459.311	459.28	0.02
3600	459.252	459.261	459.331	459.395	459.31	0.07
7200	459.278	459.305	459.297	459.442	459.33	0.08
14400	459.207	459.355	459.426	459.579	459.39	0.15

APO

Time	Replicate 1	Replicate 2	Replicate 3	Replicate 4	Replicate 5	average	SD
0	459.201	459.145	459.163	459.149	459.161	459.16	0.02
30		459.234	459.212	459.146	459.272	459.22	0.05
45		459.225			459.279	459.25	0.04
60		459.266	459.261	459.267	459.279	459.27	0.01
300	459.403	459.24	459.237		459.26	459.29	0.08
1500	459.438	459.351	459.283	459.298	459.242	459.32	0.08
3600	459.491	459.345	459.312	459.248	459.354	459.35	0.09
7200	459.608	459.393	459.449	459.357	459.524	459.47	0.10
14400	459.67	459.599	459.477	459.347	459.715	459.56	0.15

DNA

Time	Replicate 1	Replicate 2	Replicate 3	Replicate 4	Replicate 5	average	SD
0	459.164	459.154	459.156			459.16	0.01
30	459.222	459.166	459.247			459.21	0.04
45	459.222		459.265			459.24	0.03
60	459.242	459.176	459.284			459.23	0.05
300	459.23	459.178	459.271			459.23	0.05
1500	459.269	459.181	459.283			459.24	0.06
3600	459.258	459.186	459.324			459.26	0.07
7200	459.282	459.171	459.325			459.26	0.08
14400	459.241	459.182	459.409			459.28	0.12

ONPFDNA

Time	Replicate 1	Replicate 2	Replicate 3	Replicate 4	average	SD
0	459.161	459.157	459.164		459.16	0.00
30	459.217	459.16	459.235		459.20	0.04
45	459.229				459.23	
60	459.24	459.19	459.271		459.23	0.04
300	459.222	459.171	459.304		459.23	0.07
1500	459.231	459.173	459.268		459.22	0.05
3600	459.245	459.182	459.329		459.25	0.07
7200	459.243	459.184	459.367		459.26	0.09
14400	459.237	459.191	459.284		459.24	0.05

TMG

Time	Replicate 1	Replicate 2	Replicate 3	Replicate 4	Replicate 5	average	SD
0	459.161	459.168				459.16	0.00
30	459.073	459.279				459.18	0.15
45	459.291	459.284				459.29	0.00
60	459.319	459.551				459.44	0.16
300	459.222	459.304				459.26	0.06
1500	459.248	459.349				459.30	0.07
3600	459.307	459.311				459.31	0.00
7200	459.223	459.32				459.27	0.07
14400	459.318	459.398				459.36	0.06

164-173 EDGTRLGVEH

Charge 2

IPTG

Time	centroid	D
0	557.0895	0.04
30	557.2945	0.82
45	557.2205	0.54
60	557.29	0.81
300	557.2795	0.77
1500	557.231	0.58
3600	557.223	0.55
7200	557.236	0.60
14400	557.291	0.81

ONPF

Time	centroid	D
0	557.09	0.02
30	557.19	0.43
45	557.26	0.67
60	557.24	0.62
300	557.24	0.62
1500	557.25	0.65
3600	557.31	0.88
7200	557.33	0.97
14400	557.51	1.64

APO

Time	centroid	D
0	557.0765	-0.01
30	557.182	0.39
45	557.198	0.45
60	557.2055	0.48
300	557.197	0.45
1500	557.2845	0.79
3600	557.3105	0.89
7200	557.433	1.36
14400	557.6805	2.31

DNA

Time	centroid	D
0	557.0715	-0.03
30	557.1965	0.45
45	557.203	0.47
60	557.2325	0.59
300	557.2215	0.54
1500	557.212	0.51
3600	557.2665	0.72
7200	557.2305	0.58
14400	557.251	0.66

ONPFDNA

Time	centroid	D
0	557.077	-0.01
30	557.216	0.52
45	557.206	0.48
60	557.2435	0.63
300	557.2085	0.49
1500	557.2095	0.50
3600	557.2635	0.71
7200	557.246	0.64
14400	557.256	0.68

TMG

Time	centroid	D
0	557.10	0.09
30	557.19	0.44
45	557.29	0.81
60	557.23	0.59
300	557.24	0.60
1500	557.33	0.96
3600	557.26	0.68
7200	557.23	0.57
14400	557.37	1.11

control 557.08
infinity 559.161

$$D(t) = \frac{M_t - M_0}{M_\infty - M_0} * N$$

IPTG

Time	Replicate 1	Replicate 2	Replicate 3	Replicate 4	Replicate5	average	SD
0	557.08	557.099				557.09	0.01
30	557.281	557.308				557.29	0.02
45	557.25	557.191				557.22	0.04
60	557.331	557.249				557.29	0.06
300	557.364	557.195				557.28	0.12
1500	557.248	557.214				557.23	0.02
3600	557.267	557.179				557.22	0.06
7200	557.259	557.213				557.24	0.03
14400	557.337	557.245				557.29	0.07

ONPF

Time	Replicate 1	Replicate 2	Replicate 3	Replicate 4	average	SD
0	557.074	557.099			557.09	0.02
30	557.218	557.168			557.19	0.04
45	557.307	557.204			557.26	0.07
60	557.242	557.239			557.24	0.00
300	557.279	557.202			557.24	0.05
1500	557.289	557.21			557.25	0.06
3600	557.393	557.226			557.31	0.12
7200	557.391	557.272			557.33	0.08
14400	557.507				557.51	

APO

Time	Replicate 1	Replicate 2	Replicate 3	Replicate 4	Replicate 5	average	SD
0	557.071	557.082				557.08	0.01
30	557.213	557.151				557.18	0.04
45	557.218	557.178				557.20	0.03
60	557.208	557.203				557.21	0.00
300	557.208	557.186				557.20	0.02
1500	557.305	557.264				557.28	0.03
3600	557.341	557.28				557.31	0.04
7200	557.5	557.366				557.43	0.09
14400	557.858	557.503				557.68	0.25

DNA

Time	Replicate 1	Replicate 2	Replicate 3	Replicate 4	Replicate 5	average	SD
0	557.055	557.088				557.07	0.02
30	557.22	557.173				557.20	0.03
45	557.22	557.186				557.20	0.02
60	557.277	557.188				557.23	0.06
300	557.236	557.207				557.22	0.02
1500	557.227	557.197				557.21	0.02
3600	557.346	557.187				557.27	0.11
7200	557.266	557.195				557.23	0.05
14400	557.337	557.165				557.25	0.12

ONPFDNA

Time	Replicate 1	Replicate 2	Replicate 3	Replicate 4	average	SD
0	557.07	557.084			557.08	0.01
30	557.248	557.184			557.22	0.05
45	557.219	557.193			557.21	0.02
60	557.224	557.263			557.24	0.03
300	557.217	557.2			557.21	0.01
1500	557.179	557.24			557.21	0.04
3600	557.355	557.172			557.26	0.13
7200	557.278	557.214			557.25	0.05
14400	557.246	557.266			557.26	0.01

TMG

Time	Replicate 1	Replicate 2	Replicate 3	Replicate 4	Replicate 5	average	SD
0	557.133	557.072				557.10	0.04
30	557.135	557.254				557.19	0.08
45	557.158	557.421				557.29	0.19
60	557.233					557.23	
300	557.163	557.311				557.24	0.10
1500	557.17	557.489				557.33	0.23
3600	557.247	557.267				557.26	0.01
7200	557.179	557.28				557.23	0.07
14400	557.215	557.52				557.37	0.22

174-177 LVAL

Charge 1

IPTG

Time	centroid	D
0	415.52	0.00
30	415.56	0.05
45	415.56	0.05
60	415.57	0.07
300	415.57	0.07
1500	415.58	0.07
3600	415.60	0.10
7200	415.64	0.16
14400	415.70	0.23

ONPF

Time	centroid	D
0	415.52	0.00
30	415.55	0.04
45	415.58	0.08
60	415.57	0.06
300	415.57	0.06
1500	415.57	0.07
3600	415.59	0.09
7200	415.61	0.12
14400	415.69	0.22

APO

Time	centroid	D
0	415.52	0.00
30	415.55	0.04
45	415.55	0.04
60	415.57	0.06
300	415.56	0.05
1500	415.59	0.09
3600	415.60	0.10
7200	415.64	0.16
14400	415.82	0.38

DNA

Time	centroid	D
0	415.52	0.00
30	415.55	0.04
45	415.58	0.07
60	415.56	0.05
300	415.56	0.05
1500	415.58	0.07
3600	415.60	0.10
7200	415.61	0.11
14400	415.63	0.14

ONPFDNA

Time	centroid	D
0	415.52	0.00
30	415.55	0.03
45	415.56	0.05
60	415.56	0.06
300	415.56	0.05
1500	415.57	0.06
3600	415.59	0.09
7200	415.60	0.11
14400	415.61	0.11

TMG

Time	centroid	D
0	415.52	0.00
30	415.56	0.05
45	415.59	0.09
60	415.66	0.18
300	415.58	0.07
1500	415.61	0.11
3600	415.61	0.12
7200	415.63	0.14
14400	415.71	0.24

control 415.52
infinity 417.087

$$D(t) = \frac{M_t - M_0}{M_\infty - M_0} *N$$

IPTG

Time	Replicate 1	Replicate 2	Replicate 3	Replicate 4	Replicate5	average	SD
0	415.52	415.52				415.52	0.00
30	415.564	415.548				415.56	0.01
45	415.57	415.548				415.56	0.02
60	415.588	415.556				415.57	0.02
300	415.595	415.55				415.57	0.03
1500	415.583	415.57				415.58	0.01
3600	415.615	415.588				415.60	0.02
7200	415.654	415.635				415.64	0.01
14400	415.738	415.662				415.70	0.05

ONPF

Time	Replicate 1	Replicate 2	Replicate 3	Replicate 4	average	SD
0	415.516	415.527			415.52	0.01
30	415.55	415.55			415.55	0.00
45	415.598	415.565			415.58	0.02
60	415.567	415.573			415.57	0.00
300	415.569	415.562			415.57	0.00
1500	415.575	415.573			415.57	0.00
3600	415.596	415.584			415.59	0.01
7200	415.62	415.607			415.61	0.01
14400	415.689	415.689			415.69	0.00

APO

Time	Replicate 1	Replicate 2	Replicate 3	Replicate 4	Replicate 5	average	SD
0	415.519	415.524				415.52	0.00
30	415.548	415.55				415.55	0.00
45	415.552	415.556				415.55	0.00
60	415.55	415.58				415.57	0.02
300	415.551	415.561				415.56	0.01
1500	415.564	415.62				415.59	0.04
3600	415.593	415.611				415.60	0.01
7200	415.642	415.644				415.64	0.00
14400	415.862	415.771				415.82	0.06

DNA

Time	Replicate 1	Replicate 2	Replicate 3	Replicate 4	Replicate 5	average	SD
0	415.519	415.52				415.52	0.00
30	415.561	415.541				415.55	0.01
45	415.607	415.549				415.58	0.04
60	415.56	415.564				415.56	0.00
300	415.57	415.554				415.56	0.01
1500	415.577	415.578				415.58	0.00
3600	415.619	415.577				415.60	0.03
7200	415.62	415.594				415.61	0.02
14400	415.7	415.558				415.63	0.10

ONPFDNA

Time	Replicate 1	Replicate 2	Replicate 3	Replicate 4	average	SD
0	415.516	415.519			415.52	0.00
30	415.551	415.543			415.55	0.01
45	415.563	415.552			415.56	0.01
60	415.566	415.561			415.56	0.00
300	415.558	415.554			415.56	0.00
1500	415.556	415.583			415.57	0.02
3600	415.603	415.577			415.59	0.02
7200	415.607	415.598			415.60	0.01
14400	415.592	415.621			415.61	0.02

TMG

Time	Replicate 1	Replicate 2	Replicate 3	Replicate 4	Replicate 5	average	SD
0	415.522	415.518				415.52	0.00
30	415.558	415.563				415.56	0.00
45	415.608	415.57				415.59	0.03
60	415.57	415.748				415.66	0.13
300	415.577	415.578				415.58	0.00
1500	415.577	415.639				415.61	0.04
3600	415.629	415.596				415.61	0.02
7200	415.625	415.632				415.63	0.00
14400	415.703	415.711				415.71	0.01

174-184 LVALGHQQIAL

Charge 2

IPTG

Time	centroid	D
0	582.21	0.02
30	582.67	1.41
45	582.77	1.71
60	582.80	1.79
300	582.74	1.63
1500	582.79	1.78
3600	582.86	1.99
7200	582.95	2.26
14400	583.00	2.41

ONPF

Time	centroid	D
0	582.24	0.12
30	582.49	0.87
45	582.69	1.48
60	582.70	1.50
300	582.68	1.44
1500	582.72	1.55
3600	582.75	1.64
7200	582.75	1.66
14400	582.80	1.80

APO

Time	centroid	D
0	582.2	0.00
30	582.632	1.30
45	582.6825	1.45
60	582.7415	1.63
300	582.653	1.36
1500	582.7725	1.72
3600	582.8405	1.92
7200	582.8485	1.95
14400	583.1055	2.72

DNA

Time	centroid	D
0	582.21	0.03
30	582.61	1.22
45	582.73	1.58
60	582.73	1.59
300	582.74	1.62
1500	582.78	1.74
3600	582.82	1.85
7200	582.78	1.74
14400	582.83	1.88

ONPFDNA

Time	centroid	D
0	582.20	0.00
30	582.57	1.12
45	582.71	1.53
60	582.72	1.56
300	582.72	1.56
1500	582.68	1.44
3600	582.83	1.90
7200	582.78	1.74
14400	582.69	1.48

TMG

Time	centroid	D
0	582.21	0.03
30	582.69	1.46
45	582.78	1.75
60	582.90	2.09
300	582.82	1.86
1500	582.84	1.91
3600	582.91	2.13
7200	582.94	2.23
14400	583.07	2.61

control 582.2
infinity 585.199

$$D(t) = \frac{M_t - M_0}{M_\infty - M_0} * N$$

IPTG

Time	Replicate 1	Replicate 2	Replicate 3	Replicate 4	Replicate5	average	SD
0	582.21	582.2				582.21	0.01
30	582.818	582.525				582.67	0.21
45	582.855	582.682				582.77	0.12
60	582.892	582.703				582.80	0.13
300	582.868	582.619				582.74	0.18
1500	582.938	582.65				582.79	0.20
3600	582.976	582.752				582.86	0.16
7200	583.093	582.816				582.95	0.20
14400	583.16	582.848				583.00	0.22

ONPF

Time	Replicate 1	Replicate 2	Replicate 3	Replicate 4	average	SD
0	582.235	582.245			582.24	0.01
30	582.544	582.439			582.49	0.07
45	582.817	582.568			582.69	0.18
60	582.727	582.671			582.70	0.04
300	582.761	582.6			582.68	0.11
1500	582.783	582.653			582.72	0.09
3600	582.832	582.659			582.75	0.12
7200	582.854	582.652			582.75	0.14
14400	583.036	582.562			582.80	0.34

APO

Time	Replicate 1	Replicate 2	Replicate 3	Replicate 4	Replicate 5	average	SD
0	582.193	582.207				582.20	0.01
30	582.745	582.519				582.63	0.16
45	582.795	582.57				582.68	0.16
60	582.806	582.677				582.74	0.09
300	582.752	582.554				582.65	0.14
1500	582.818	582.727				582.77	0.06
3600	582.926	582.755				582.84	0.12
7200	583	582.697				582.85	0.21
14400	583.298	582.913				583.11	0.27

DNA

Time	Replicate 1	Replicate 2	Replicate 3	Replicate 4	Replicate 5	average	SD
0	582.195	582.223				582.21	0.02
30	582.647	582.563				582.61	0.06
45	582.805	582.65				582.73	0.11
60	582.764	582.695				582.73	0.05
300	582.786	582.695				582.74	0.06
1500	582.805	582.753				582.78	0.04
3600	582.926	582.707				582.82	0.15
7200	582.923	582.636				582.78	0.20
14400	583.054	582.596				582.83	0.32

ONPFDNA

Time	Replicate 1	Replicate 2	Replicate 3	Replicate 4	average	SD
0	582.202	582.199			582.20	0.00
30	582.613	582.535			582.57	0.06
45	582.764	582.655			582.71	0.08
60	582.745	582.694			582.72	0.04
300	582.771	582.667			582.72	0.07
1500	582.68	582.681			582.68	0.00
3600	582.9	582.769			582.83	0.09
7200	582.905	582.652			582.78	0.18
14400	582.692	582.697			582.69	0.00

TMG

Time	Replicate 1	Replicate 2	Replicate 3	Replicate 4	Replicate 5	average	SD
0	582.225	582.195				582.21	0.02
30	582.566	582.808				582.69	0.17
45	582.768	582.8				582.78	0.02
60	582.761	583.034				582.90	0.19
300	582.8	582.837				582.82	0.03
1500	582.729	582.946				582.84	0.15
3600	582.874	582.946				582.91	0.05
7200	582.85	583.034				582.94	0.13
14400	583.006	583.133				583.07	0.09

177-185 LGHQQIALL

Charge 2

IPTG

Time	centroid	D
0	497.09	0.05
30	497.35	0.95
45	497.46	1.34
60	497.42	1.21
300	497.41	1.19
1500	497.41	1.16
3600	497.42	1.22
7200	497.44	1.26
14400	497.46	1.33

ONPF

Time	centroid	D
0	497.12	0.17
30	497.30	0.79
45	497.34	0.94
60	497.38	1.06
300	497.38	1.07
1500	497.40	1.12
3600	497.41	1.19
7200	497.42	1.20
14400	497.45	1.30

APO

Time	centroid	D
0	497.10	0.09
30	497.32	0.86
45	497.33	0.90
60	497.38	1.06
300	497.39	1.10
1500	497.40	1.15
3600	497.43	1.23
7200	497.41	1.16
14400	497.44	1.26

DNA

Time	centroid	D
0	497.08	0.04
30	497.33	0.89
45	497.43	1.24
60	497.42	1.21
300	497.44	1.28
1500	497.44	1.29
3600	497.44	1.29
7200	497.45	1.32
14400	497.44	1.29

ONPFDNA

Time	centroid	D
0	497.08	0.02
30	497.30	0.78
45	497.42	1.21
60	497.45	1.31
300	497.42	1.22
1500	497.39	1.12
3600	497.44	1.28
7200	497.44	1.29
14400	497.42	1.19

TMG

Time	centroid	D
0	497.09	0.06
30	497.38	1.07
45	497.49	1.47
60	497.54	1.63
300	497.53	1.60
1500	497.50	1.48
3600	497.49	1.44
7200	497.51	1.53
14400	497.55	1.66

control 497.072
infinity 499.088

$$D(t) = \frac{M_t - M_0}{M_\infty - M_0} * N$$

IPTG

Time	Replicate 1	Replicate 2	Replicate 3	Replicate 4	Replicate5	average	SD
0	497.083	497.095	497.092	497.087	497.071	497.09	0.01
30	497.271	497.358	497.313	497.289	497.503	497.35	0.09
45	497.358				497.559	497.46	0.14
60	497.385		497.378	497.37	497.547	497.42	0.08
300	497.296	497.485	497.384	497.348	497.56	497.41	0.11
1500	497.315	497.468	497.407	497.354	497.493	497.41	0.07
3600	497.364	497.493	497.382	497.37	497.502	497.42	0.07
7200	497.318	497.537	497.411	497.378	497.534	497.44	0.10
14400	497.398	497.528	497.428	497.377	497.544	497.46	0.08

ONPF

Time	Replicate 1	Replicate 2	Replicate 3	Replicate 4	average	SD
0	497.111	497.189	497.086	497.093	497.12	0.05
30	497.26	497.338	497.257	497.34	497.30	0.05
45	497.343				497.34	0.00
60	497.386		497.378	497.367	497.38	0.01
300	497.339	497.429	497.334	497.42	497.38	0.05
1500	497.356	497.502	497.373	497.349	497.40	0.07
3600	497.336	497.53	497.359	497.43	497.41	0.09
7200		497.506	497.355	497.396	497.42	0.08
14400		497.512	497.41	497.42	497.45	0.06

APO

Time	Replicate 1	Replicate 2	Replicate 3	Replicate 4	Replicate 5	average	SD
0	497.072	497.139	497.101	497.084		497.10	0.03
30	497.311		497.357	497.288		497.32	0.04
45	497.331					497.33	
60	497.386		497.373	497.374		497.38	0.01
300	497.317	497.482	497.394	497.358		497.39	0.07
1500	497.386	497.477	497.383	497.365		497.40	0.05
3600	497.401	497.526	497.405	497.368		497.43	0.07
7200	497.316	497.52	497.428	497.364		497.41	0.09
14400	497.378	497.511	497.439	497.413		497.44	0.06

DNA

Time	Replicate 1	Replicate 2	Replicate 3	Replicate 4	Replicate 5	average	SD
0	497.083	497.088	497.09	497.078		497.08	0.01
30	497.326	497.343	497.271	497.379		497.33	0.04
45	497.38			497.48		497.43	0.07
60	497.393		497.386	497.481		497.42	0.05
300	497.399	497.519	497.351	497.49		497.44	0.08
1500	497.42	497.506	497.363	497.488		497.44	0.07
3600	497.384	497.511	497.382	497.495		497.44	0.07
7200		497.535	497.355	497.469		497.45	0.09
14400	497.347	497.512	497.407	497.507		497.44	0.08

ONPFDNA

Time	Replicate 1	Replicate 2	Replicate 3	Replicate 4	average	SD
0	497.063	497.086	497.082		497.08	0.01
30	497.281	497.234	497.373		497.30	0.07
45	497.339		497.5		497.42	0.11
60	497.381		497.516		497.45	0.10
300	497.375	497.401	497.494		497.42	0.06
1500	497.368	497.409	497.405		497.39	0.02
3600	497.41	497.421	497.49		497.44	0.04
7200		497.404	497.484		497.44	0.06
14400		497.406	497.425		497.42	0.01

TMG

Time	Replicate 1	Replicate 2	Replicate 3	Replicate 4	Replicate 5	average	SD
0	497.109	497.07				497.09	0.03
30	497.341	497.419				497.38	0.06
45	497.483	497.506				497.49	0.02
60	497.519	497.566				497.54	0.03
300	497.516	497.547				497.53	0.02
1500	497.445	497.549				497.50	0.07
3600	497.46	497.513				497.49	0.04
7200	497.473	497.552				497.51	0.06
14400	497.523	497.578				497.55	0.04

182-185 IALL

Charge 1

IPTG

Time	centroid	D
0	429.512	-0.02
30	429.5425	0.01
45	429.5445	0.02
60	429.5615	0.04
300	429.555	0.03
1500	429.554	0.03
3600	429.546	0.02
7200	429.5515	0.02
14400	429.5425	0.01

ONPF

Time	centroid	D
0	429.502	-0.03
30	429.543	0.01
45	429.5385	0.01
60	429.5355	0.01
300	429.5375	0.01
1500	429.5305	0.00
3600	429.524	-0.01
7200	429.525	-0.01
14400	429.56	0.03

APO

Time	centroid	D
0	429.53	-0.01
30	429.55	0.02
45	429.55	0.02
60	429.56	0.04
300	429.56	0.04
1500	429.54	0.01
3600	429.55	0.02
7200	429.53	0.01
14400	429.60	0.08

DNA

Time	centroid	D
0	429.511	-0.02
30	429.535	0.01
45	429.576	0.05
60	429.5405	0.01
300	429.547	0.02
1500	429.535	0.01
3600	429.544	0.02
7200	429.5435	0.02
14400	429.579	0.06

ONPFDNA

Time	centroid	D
0	429.484	-0.05
30	429.526	0.00
45	429.523	-0.01
60	429.541	0.01
300	429.504	-0.03
1500	429.52	-0.01
3600	429.5295	0.00
7200	429.5195	-0.01
14400	429.514	-0.02

TMG

Time	centroid	D
0	429.50	-0.03
30	429.54	0.02
45	429.54	0.01
60	429.59	0.07
300	429.55	0.03
1500	429.56	0.04
3600	429.55	0.02
7200	429.54	0.01
14400	429.55	0.02

control 429.53
infinity 431.3

$$D(t) = \frac{M_t - M_0}{M_\infty - M_0} * N$$

IPTG

Time	Replicate 1	Replicate 2	Replicate 3	Replicate 4	Replicate5	average	SD
0	429.507	429.517				429.51	0.01
30	429.546	429.539				429.54	0.00
45	429.543	429.546				429.54	0.00
60	429.567	429.556				429.56	0.01
300	429.571	429.539				429.56	0.02
1500	429.549	429.559				429.55	0.01
3600	429.532	429.56				429.55	0.02
7200	429.552	429.551				429.55	0.00
14400	429.529	429.556				429.54	0.02

ONPF

Time	Replicate 1	Replicate 2	Replicate 3	Replicate 4	average	SD
0	429.51	429.494			429.50	0.01
30	429.557	429.529			429.54	0.02
45	429.546	429.531			429.54	0.01
60	429.546	429.525			429.54	0.01
300	429.542	429.533			429.54	0.01
1500	429.542	429.519			429.53	0.02
3600	429.538	429.51			429.52	0.02
7200	429.542	429.508			429.53	0.02
14400	429.56				429.56	

APO

Time	Replicate 1	Replicate 2	Replicate 3	Replicate 4	Replicate 5	average	SD
0	429.512	429.539				429.53	0.02
30	429.527	429.567				429.55	0.03
45	429.526	429.569				429.55	0.03
60	429.56	429.569				429.56	0.01
300	429.542	429.583				429.56	0.03
1500	429.524	429.561				429.54	0.03
3600	429.543	429.556				429.55	0.01
7200	429.516	429.553				429.53	0.03
14400	429.626	429.575				429.60	0.04

DNA

Time	Replicate 1	Replicate 2	Replicate 3	Replicate 4	Replicate 5	average	SD
0	429.498	429.524				429.51	0.02
30	429.544	429.526				429.54	0.01
45		429.576				429.58	
60	429.522	429.559				429.54	0.03
300	429.532	429.562				429.55	0.02
1500	429.514	429.556				429.54	0.03
3600	429.535	429.553				429.54	0.01
7200	429.542	429.545				429.54	0.00
14400	429.57	429.588				429.58	0.01

ONPFDNA

Time	Replicate 1	Replicate 2	Replicate 3	Replicate 4	average	SD
0	429.493	429.475			429.48	0.01
30	429.53	429.522			429.53	0.01
45	429.529	429.517			429.52	0.01
60	429.56	429.522			429.54	0.03
300	429.498	429.51			429.50	0.01
1500	429.518	429.522			429.52	0.00
3600	429.532	429.527			429.53	0.00
7200	429.524	429.515			429.52	0.01
14400	429.55	429.478			429.51	0.05

TMG

Time	Replicate 1	Replicate 2	Replicate 3	Replicate 4	Replicate 5	average	SD
0	429.495	429.514				429.50	0.01
30	429.525	429.562				429.54	0.03
45	429.532	429.547				429.54	0.01
60	429.528	429.65				429.59	0.09
300	429.551	429.558				429.55	0.00
1500	429.533	429.592				429.56	0.04
3600	429.543	429.556				429.55	0.01
7200	429.525	429.552				429.54	0.02
14400	429.543	429.555				429.55	0.01

185-190 LAGPLS

Charge 1

IPTG

Time	centroid	D
0	557.62	0.00
30	558.26	0.93
45	558.35	1.07
60	558.41	1.15
300	558.41	1.16
1500	558.67	1.53
3600	558.90	1.88
7200	558.89	1.86
14400	558.78	1.70

ONPF

Time	centroid	D
0	557.60	-0.03
30	558.03	0.60
45	558.21	0.87
60	558.35	1.07
300	558.39	1.13
1500	558.64	1.49
3600	558.70	1.57
7200	558.55	1.37
14400	558.84	1.79

APO

Time	centroid	D
0	557.62	0.00
30	558.23	0.89
45	558.25	0.92
60	558.39	1.13
300	558.42	1.17
1500	558.74	1.64
3600	558.82	1.75
7200	558.86	1.81
14400	558.83	1.76

DNA

Time	centroid	D
0	557.61	-0.02
30	558.16	0.79
45	558.30	0.99
60	558.29	0.97
300	558.47	1.25
1500	558.57	1.39
3600	558.62	1.46
7200	558.50	1.29
14400	558.74	1.63

ONPFDNA

Time	centroid	D
0	557.60	-0.03
30	558.15	0.78
45	558.29	0.99
60	558.29	0.98
300	558.44	1.19
1500	558.32	1.02
3600	558.66	1.52
7200		
14400	558.24	0.90

TMG

Time	centroid	D
0	557.60	-0.03
30	558.24	0.90
45	558.34	1.05
60	558.47	1.24
300	558.51	1.31
1500	558.65	1.51
3600	558.93	1.91
7200	558.88	1.84
14400	559.02	2.05

control 557.62
infinity 559.671

$$D(t) = \frac{M_t - M_0}{M_\infty - M_0} \cdot N$$

IPTG

Time	Replicate 1	Replicate 2	Replicate 3	Replicate 4	Replicate5	average	SD
0	557.618	557.625				557.62	0.00
30	558.389	558.129				558.26	0.18
45	558.391	558.316				558.35	0.05
60	558.476	558.343				558.41	0.09
300	558.461	558.365				558.41	0.07
1500	558.861	558.475				558.67	0.27
3600	558.923	558.881				558.90	0.03
7200	559.05	558.729				558.89	0.23
14400	558.844	558.722				558.78	0.09

ONPF

Time	Replicate 1	Replicate 2	Replicate 3	Replicate 4	average	SD
0	557.566	557.633			557.60	0.05
30	558.036	558.023			558.03	0.01
45	558.289	558.136			558.21	0.11
60	558.351	558.348			558.35	0.00
300	558.49	558.294			558.39	0.14
1500	558.841	558.436			558.64	0.29
3600	558.961	558.431			558.70	0.37
7200	558.714	558.393			558.55	0.23
14400	558.841				558.84	

APO

Time	Replicate 1	Replicate 2	Replicate 3	Replicate 4	Replicate 5	average	SD
0	557.614	557.623				557.62	0.01
30	558.336	558.125				558.23	0.15
45	558.347	558.156				558.25	0.14
60	558.454	558.334				558.39	0.08
300	558.563	558.275				558.42	0.20
1500	558.828	558.656				558.74	0.12
3600	558.906	558.733				558.82	0.12
7200	558.859					558.86	
14400	558.946	558.705				558.83	0.17

DNA

Time	Replicate 1	Replicate 2	Replicate 3	Replicate 4	Replicate 5	average	SD
0	557.593	557.626				557.61	0.02
30	558.13	558.192				558.16	0.04
45	558.395	558.201				558.30	0.14
60	558.299	558.271				558.29	0.02
300	558.494	558.455				558.47	0.03
1500		558.569				558.57	
3600	558.755	558.48				558.62	0.19
7200	558.659	558.341				558.50	0.22
14400	558.736					558.74	

ONPFDNA

Time	Replicate 1	Replicate 2	Replicate 3	Replicate 4	average	SD
0	557.587	557.607			557.60	0.01
30	558.093	558.211			558.15	0.08
45	558.284	558.304			558.29	0.01
60	558.27	558.313			558.29	0.03
300	558.433	558.44			558.44	0.00
1500	558.284	558.351			558.32	0.05
3600	558.637	558.684			558.66	0.03
7200						
14400	558.161	558.309			558.24	0.10

TMG

Time	Replicate 1	Replicate 2	Replicate 3	Replicate 4	Replicate 5	average	SD
0	557.633	557.563				557.60	0.05
30	558.08	558.397				558.24	0.22
45	558.317	558.353				558.34	0.03
60	558.317	558.622				558.47	0.22
300	558.481	558.546				558.51	0.05
1500	558.555	558.753				558.65	0.14
3600	558.909	558.943				558.93	0.02
7200	558.716	559.04				558.88	0.23
14400	558.916	559.123				559.02	0.15

185-196 LAGPLSSVSARL

Charge 2

IPTG

Time	centroid	D
0	586.1762	-0.14
30	586.4296	0.92
45	586.6877	1.99
60	586.8808	2.80
300	586.9417	3.06
1500	587.2398	4.30
3600	587.3935	4.94
7200	587.6755	6.12
14400	587.6406	5.97

ONPF

Time	centroid	D
0	586.20	-0.03
30	586.65	1.83
45	586.95	3.09
60	587.18	4.05
300	587.28	4.46
1500	587.61	5.85
3600	587.59	5.78
7200	587.45	5.18
14400	587.57	5.67

APO

Time	centroid	D
0	586.208	-0.01
30	586.7106	2.09
45	586.958	3.12
60	587.279	4.46
300	587.2793	4.47
1500	587.4508	5.18
3600	587.5124	5.44
7200	587.6995	6.22
14400	587.742	6.40

DNA

Time	centroid	D
0	586.32	0.44
30	586.95	3.11
45	587.16	3.98
60	587.35	4.76
300	587.50	5.39
1500	587.62	5.89
3600	587.67	6.12
7200	587.52	5.49
14400	587.56	5.65

ONPFDNA

Time	centroid	D
0	586.17	-0.18
30	586.73	2.16
45	587.10	3.73
60	587.21	4.18
300	587.36	4.79
1500	587.46	5.24
3600	587.74	6.40
7200	587.48	5.32
14400	587.50	5.37

TMG

Time	centroid	D
0	586.19	-0.07
30	586.60	1.62
45	586.95	3.08
60	587.20	4.13
300	587.07	3.57
1500	587.30	4.57
3600	587.79	6.61
7200	588.02	7.58
14400	588.02	7.57

control 586.21
infinity 588.365

$$D(t) = \frac{M_t - M_0}{M_\infty - M_0} *N$$

IPTG

Time	Replicate 1	Replicate 2	Replicate 3	Replicate 4	Replicate5	average	SD
0	586.148	586.171	586.187	586.187	586.188	586.18	0.02
30	586.328	586.396	586.346	586.403	586.675	586.43	0.14
45		586.599	586.583		586.881	586.69	0.17
60		586.735	586.993	586.924	586.871	586.88	0.11
300			587.076	586.86	586.889	586.94	0.12
1500		587.122	587.44	587.115	587.282	587.24	0.15
3600	587.255	587.513	587.519	587.287		587.39	0.14
7200	587.551	587.569	587.674		587.908	587.68	0.16
14400	587.655	587.571	587.79	587.585	587.602	587.64	0.09

ONPF

Time	Replicate 1	Replicate 2	Replicate 3	Replicate 4	average	SD
0	586.129	586.258	586.187	586.237	586.20	0.06
30	586.702	586.502		586.744	586.65	0.13
45		586.685		587.214	586.95	0.37
60		587.052	587.192	587.292	587.18	0.12
300	587.193	586.999	587.249	587.666	587.28	0.28
1500	587.746	587.225	587.499	587.97	587.61	0.32
3600	587.853	587.186		587.745	587.59	0.36
7200		587.062	587.592	587.695	587.45	0.34
14400	587.878	586.437	587.684	588.27	587.57	0.79

APO

Time	Replicate 1	Replicate 2	Replicate 3	Replicate 4	Replicate 5	average	SD
0	586.288	586.253	586.186	586.185	586.128	586.21	0.06
30	586.864	586.778	586.478	586.772	586.661	586.71	0.15
45		586.884	586.848		587.142	586.96	0.16
60	587.433	587.289	587.261	587.26	587.152	587.28	0.10
300	587.344		587.493	587.241	587.039	587.28	0.19
1500		587.49	587.59	587.558	587.165	587.45	0.20
3600	587.81	587.595	587.668	587.74	586.749	587.51	0.43
7200	587.938		587.813	587.605	587.442	587.70	0.22
14400	587.696	587.661	587.85	587.84	587.663	587.74	0.10

DNA

Time	Replicate 1	Replicate 2	Replicate 3	Replicate 4	Replicate 5	average	SD
0	586.192	586.192	586.702	586.174		586.32	0.26
30	586.713	586.798	587.338	586.967		586.95	0.28
45		586.968		587.36		587.16	0.28
60	587.473	587.241	587.373	587.31		587.35	0.10
300	587.397	587.401	587.658	587.544		587.50	0.13
1500	587.649	587.733	587.271	587.832		587.62	0.25
3600	587.703	587.389	587.616	587.99		587.67	0.25
7200	587.497	587.179	587.625	587.797		587.52	0.26
14400	587.625	586.991	587.692	587.942		587.56	0.40

ONPFDNA

Time	Replicate 1	Replicate 2	Replicate 3	Replicate 4	average	SD
0	586.149	586.163	586.185	586.171	586.17	0.02
30	586.842	586.66	586.532	586.871	586.73	0.16
45		586.976		587.229	587.10	0.18
60		587.147	587.258	587.23	587.21	0.06
300	587.303	587.161	587.342	587.619	587.36	0.19
1500	587.687	587.407	587.476	587.288	587.46	0.17
3600	587.628		587.651	587.949	587.74	0.18
7200		587.131	587.555	587.762	587.48	0.32
14400	587.774	587.225	587.635	587.346	587.50	0.25

TMG

Time	Replicate 1	Replicate 2	Replicate 3	Replicate 4	Replicate 5	average	SD
0	586.23	586.156				586.19	0.05
30	586.533	586.662				586.60	0.09
45	586.955	586.942				586.95	0.01
60	587.085	587.312				587.20	0.16
300	587.061	587.069				587.07	0.01
1500	587.131	587.477				587.30	0.24
3600	587.72	587.866				587.79	0.10
7200		588.024				588.02	
14400		588.023				588.02	

187-195 GPLSSVSAR

Charge 2

IPTG

Time	centroid	D
0	437.51	0.03
30	437.96	1.26
45	438.08	1.57
60	438.18	1.85
300	438.26	2.06
1500	438.67	3.15
3600	438.81	3.55
7200	439.13	4.41
14400	438.87	3.71

ONPF

Time	centroid	D
0	437.54	0.11
30	438.03	1.43
45	438.40	2.43
60	438.54	2.80
300	438.68	3.18
1500	438.60	2.97
3600	438.87	3.70
7200	438.82	3.57
14400	438.38	2.37

APO

Time	centroid	D
0	437.50	0.01
30	438.31	2.19
45	438.31	2.18
60	438.66	3.13
300	438.49	2.68
1500	438.60	2.98
3600	438.94	3.89
7200	438.94	3.90
14400	439.18	4.54

DNA

Time	centroid	D
0	437.51	0.02
30	438.19	1.85
45	438.58	2.92
60	438.58	2.91
300	438.77	3.42
1500	438.86	3.67
3600	438.74	3.34
7200	438.82	3.57
14400	438.67	3.15

ONPFDNA

Time	centroid	D
0	437.51	0.02
30	438.11	1.66
45	438.37	2.35
60	438.43	2.52
300	438.59	2.94
1500	438.59	2.95
3600	439.11	4.36
7200	438.77	3.43
14400	438.54	2.80

TMG

Time	centroid	D
0	437.52	0.04
30	437.97	1.27
45	438.19	1.86
60	438.13	1.71
300	438.37	2.36
1500	438.65	3.10
3600	439.07	4.25
7200	439.23	4.68
14400	439.51	5.45

control 437.5
infinity 440.088

$$D(t) = \frac{M_t - M_0}{M_\infty - M_0} * N$$

IPTG

Time	Replicate 1	Replicate 2	Replicate 3	Replicate 4	Replicate5	average	SD
0	437.512	437.509				437.51	0.00
30	438.073	437.855				437.96	0.15
45	438.156	438.004				438.08	0.11
60	438.255	438.11				438.18	0.10
300	438.287	438.233				438.26	0.04
1500	438.75	438.58				438.67	0.12
3600	438.649	438.978				438.81	0.23
7200	439.228	439.031				439.13	0.14
14400	439.179	438.562				438.87	0.44

ONPF

Time	Replicate 1	Replicate 2	Replicate 3	Replicate 4	average	SD
0	437.55	437.53			437.54	0.01
30	438.117	437.938			438.03	0.13
45	438.702	438.096			438.40	0.43
60	438.575	438.496			438.54	0.06
300	438.781	438.57			438.68	0.15
1500	438.613	438.583			438.60	0.02
3600	439.017	438.721			438.87	0.21
7200	438.983	438.654			438.82	0.23
14400	438.642	438.112			438.38	0.37

APO

Time	Replicate 1	Replicate 2	Replicate 3	Replicate 4	Replicate 5	average	SD
0	437.489	437.516				437.50	0.02
30	438.448	438.169				438.31	0.20
45	438.363	438.251				438.31	0.08
60	438.732	438.581				438.66	0.11
300		438.489				438.49	
1500	438.618	438.587				438.60	0.02
3600	439.308	438.567				438.94	0.52
7200	439.328	438.557				438.94	0.55
14400	439.306	439.053				439.18	0.18

DNA

Time	Replicate 1	Replicate 2	Replicate 3	Replicate 4	Replicate 5	average	SD
0	437.501	437.516				437.51	0.01
30	438.166	438.205				438.19	0.03
45	438.6	438.556				438.58	0.03
60	438.604	438.551				438.58	0.04
300	438.755	438.775				438.77	0.01
1500	438.619	439.095				438.86	0.34
3600	438.644	438.829				438.74	0.13
7200	439.092	438.551				438.82	0.38
14400	439.056	438.276				438.67	0.55

ONPFDNA

Time	Replicate 1	Replicate 2	Replicate 3	Replicate 4	average	SD
0	437.505	437.509			437.51	0.00
30	438.166	438.061			438.11	0.07
45	438.48	438.258			438.37	0.16
60	438.411	438.456			438.43	0.03
300	438.587	438.585			438.59	0.00
1500	438.614	438.565			438.59	0.03
3600	439.122	439.1			439.11	0.02
7200	438.976	438.558			438.77	0.30
14400	438.54	438.533			438.54	0.00

TMG

Time	Replicate 1	Replicate 2	Replicate 3	Replicate 4	Replicate 5	average	SD
0	437.529	437.501				437.52	0.02
30	437.824	438.118				437.97	0.21
45	438.258	438.121				438.19	0.10
60	438.133					438.13	
300	438.35	438.394				438.37	0.03
1500	438.495	438.795				438.65	0.21
3600	438.944	439.198				439.07	0.18
7200	439.035	439.426				439.23	0.28
14400		439.514				439.51	

198-203 LAGWHK

Charge 1

IPTG

Time	centroid	D
0	711.87	0.04
30	712.05	0.41
45	712.03	0.38
60	712.10	0.52
300	712.03	0.37
1500	712.04	0.40
3600	711.97	0.25
7200	711.98	0.26
14400	712.03	0.38

ONPF

Time	centroid	D
0	711.87	0.04
30	711.95	0.20
45	711.99	0.29
60	711.99	0.28
300	711.99	0.29
1500	711.97	0.25
3600	711.96	0.24
7200	712.03	0.36
14400	712.03	0.37

APO

Time	centroid	D
0	711.85	0.01
30	711.95	0.21
45	712.02	0.35
60	711.96	0.22
300	712.02	0.35
1500	711.98	0.26
3600	711.98	0.26
7200	711.98	0.27
14400	712.13	0.58

DNA

Time	centroid	D
0	711.92	0.15
30	712.05	0.42
45	712.06	0.42
60	711.97	0.25
300	712.04	0.40
1500	712.03	0.37
3600	712.07	0.45
7200	712.02	0.36
14400	712.01	0.32

ONPFDNA

Time	centroid	D
0	711.81	-0.08
30	712.01	0.33
45	711.97	0.25
60	711.97	0.24
300	712.03	0.37
1500	711.95	0.22
3600	711.97	0.24
7200	711.96	0.23
14400	712.02	0.35

TMG

Time	centroid	D
0	711.85	-0.01
30	711.98	0.26
45	712.01	0.32
60	711.99	0.30
300	712.11	0.53
1500	712.03	0.36
3600	712.06	0.44
7200	711.99	0.29
14400	712.06	0.44

control 711.85
infinity 713.783

$$D(t) = \frac{M_t - M_0}{M_\infty - M_0} * N$$

IPTG

Time	Replicate 1	Replicate 2	Replicate 3	Replicate 4	Replicate5	average	SD
0	711.849	711.887				711.87	0.03
30	712.058	712.034				712.05	0.02
45	712.053	712.01				712.03	0.03
60	712.092	712.113				712.10	0.01
300	712.093	711.968				712.03	0.09
1500	711.974	712.111				712.04	0.10
3600	712.019	711.921				711.97	0.07
7200	712.016	711.937				711.98	0.06
14400	712.052	712.015				712.03	0.03

ONPF

Time	Replicate 1	Replicate 2	Replicate 3	Replicate 4	average	SD
0	711.822	711.919			711.87	0.07
30	711.954	711.942			711.95	0.01
45	712.017	711.968			711.99	0.03
60	711.985	711.987			711.99	0.00
300	711.988	711.997			711.99	0.01
1500	711.99	711.953			711.97	0.03
3600	711.977	711.951			711.96	0.02
7200	711.988	712.064			712.03	0.05
14400	712.027				712.03	0.00

APO

Time	Replicate 1	Replicate 2	Replicate 3	Replicate 4	Replicate 5	average	SD
0	711.851	711.857				711.85	0.00
30	712.001	711.898				711.95	0.07
45	712.028	712.009				712.02	0.01
60	711.985	711.927				711.96	0.04
300	712.019	712.016				712.02	0.00
1500	711.966	711.986				711.98	0.01
3600	711.991	711.959				711.98	0.02
7200	712.047	711.917				711.98	0.09
14400	712.222	712.039				712.13	0.13

DNA

Time	Replicate 1	Replicate 2	Replicate 3	Replicate 4	Replicate 5	average	SD
0	711.824	712.022				711.92	0.14
30	711.984	712.122				712.05	0.10
45	711.963	712.147				712.06	0.13
60	711.959	711.984				711.97	0.02
300	711.97	712.119				712.04	0.11
1500	712.07	711.985				712.03	0.06
3600	712.027	712.105				712.07	0.06
7200	711.978	712.07				712.02	0.07
14400	712.006	712.006				712.01	0.00

ONPFDNA

Time	Replicate 1	Replicate 2	Replicate 3	Replicate 4	average	SD
0	711.832	711.792			711.81	0.03
30	711.957	712.065			712.01	0.08
45	711.984	711.961			711.97	0.02
60	711.993	711.943			711.97	0.04
300	711.973	712.083			712.03	0.08
1500	711.93	711.978			711.95	0.03
3600	711.967				711.97	
7200	711.979	711.944			711.96	0.02
14400	711.922	712.118			712.02	0.14

TMG

Time	Replicate 1	Replicate 2	Replicate 3	Replicate 4	Replicate 5	average	SD
0	711.815	711.876				711.85	0.04
30	711.881	712.073				711.98	0.14
45	711.89	712.121				712.01	0.16
60	711.994					711.99	
300	712.106	712.111				712.11	0.00
1500	711.859	712.193				712.03	0.24
3600	712.034	712.09				712.06	0.04
7200	711.868	712.112				711.99	0.17
14400	711.922	712.205				712.06	0.20

204-212 YLTRNQIQP

Charge 2

IPTG

Time	centroid	D
0	567.1345	0.01
30	567.5975	1.15
45	567.7285	1.48
60	567.768	1.58
300	567.845	1.77
1500	568.062	2.30
3600	568.3575	3.03
7200	568.5455	3.50
14400	568.4985	3.38

ONPF

Time	centroid	D
0	567.142	0.03
30	567.382	0.62
45	567.6465	1.28
60	567.732	1.49
300	567.76	1.56
1500	567.9385	2.00
3600	568.0275	2.22
7200	568.108	2.41
14400	568.32	2.93

APO

Time	centroid	D
0	567.131	0.00
30	567.5225	0.97
45	567.592	1.14
60	567.775	1.59
300	567.724	1.47
1500	568.243	2.75
3600	568.2395	2.74
7200	568.434	3.22
14400	568.5435	3.49

DNA

Time	centroid	D
0	567.125	-0.01
30	567.5345	1.00
45	567.6395	1.26
60	567.668	1.33
300	567.773	1.59
1500	568.024	2.21
3600	568.0925	2.38
7200	568.301	2.89
14400	568.557	3.52

ONPFDNA

Time	centroid	D
0	567.121	-0.02
30	567.538	1.01
45	567.6065	1.18
60	567.6745	1.34
300	567.7655	1.57
1500	567.796	1.64
3600	568.141	2.50
7200	568.3155	2.93
14400		

TMG

Time	centroid	D
0	567.1315	0.00
30	567.59	1.14
45	567.713	1.44
60	567.794	1.64
300	567.8815	1.86
1500	568.155	2.53
3600	568.4125	3.17
7200	568.568	3.55
14400	568.7	3.88

control 567.13
infinity 569.56

$$D(t) = \frac{M_t - M_0}{M_\infty - M_0} * N$$

IPTG

Time	Replicate 1	Replicate 2	Replicate 3	Replicate 4	Replicate5	average	SD
0	567.152	567.117				567.13	0.02
30	567.761	567.434				567.60	0.23
45	567.803	567.654				567.73	0.11
60	567.816	567.72				567.77	0.07
300	567.953	567.737				567.85	0.15
1500	568.265	567.859				568.06	0.29
3600	568.469	568.246				568.36	0.16
7200	568.735	568.356				568.55	0.27
14400	568.752	568.245				568.50	0.36

ONPF

Time	Replicate 1	Replicate 2	Replicate 3	Replicate 4	average	SD
0	567.134	567.15			567.14	0.01
30	567.395	567.369			567.38	0.02
45	567.904	567.389			567.65	0.36
60	567.741	567.723			567.73	0.01
300	567.89	567.63			567.76	0.18
1500	567.988	567.889			567.94	0.07
3600	568.128	567.927			568.03	0.14
7200	568.114	568.102			568.11	0.01
14400	568.412	568.225			568.32	0.13

APO

Time	Replicate 1	Replicate 2	Replicate 3	Replicate 4	Replicate 5	average	SD
0	567.14	567.122				567.13	0.01
30	567.583	567.462				567.52	0.09
45	567.633	567.551				567.59	0.06
60	567.835	567.715				567.78	0.08
300	567.887	567.561				567.72	0.23
1500	568.474	568.012				568.24	0.33
3600	568.353	568.126				568.24	0.16
7200	568.665	568.203				568.43	0.33
14400	568.809	568.278				568.54	0.38

DNA

Time	Replicate 1	Replicate 2	Replicate 3	Replicate 4	Replicate 5	average	SD
0	567.13	567.12				567.13	0.01
30	567.538	567.531				567.53	0.00
45	567.757	567.522				567.64	0.17
60	567.666	567.67				567.67	0.00
300	567.845	567.701				567.77	0.10
1500	568.019	568.029				568.02	0.01
3600	568.212	567.973				568.09	0.17
7200	568.439	568.163				568.30	0.20
14400	568.557					568.56	

ONPFDNA

Time	Replicate 1	Replicate 2	Replicate 3	Replicate 4	average	SD
0	567.122	567.12			567.12	0.00
30	567.488	567.588			567.54	0.07
45	567.649	567.564			567.61	0.06
60	567.726	567.623			567.67	0.07
300	567.835	567.696			567.77	0.10
1500	567.714	567.878			567.80	0.12
3600	568.149	568.133			568.14	0.01
7200	568.3	568.331			568.32	0.02
14400					#DIV/0!	

TMG

Time	Replicate 1	Replicate 2	Replicate 3	Replicate 4	Replicate 5	average	SD
0	567.14	567.123				567.13	0.01
30	567.439	567.741				567.59	0.21
45		567.713				567.71	
60	567.723	567.865				567.79	0.10
300	567.833	567.93				567.88	0.07
1500		568.155				568.16	
3600	568.295	568.53				568.41	0.17
7200		568.568				568.57	
14400	568.52	568.88				568.70	0.25

208-219 NQIQPIAEREGD

Charge 2

IPTG

Time	centroid	D
0	685.732	0.04
30	686.5615	2.47
45	686.7235	2.94
60	686.768	3.07
300	686.8305	3.25
1500	686.7885	3.13
3600	686.993	3.73
7200	687.1135	4.08
14400	687.2465	4.47

ONPF

Time	centroid	D
0	685.74	0.06
30	686.29	1.66
45	686.58	2.52
60	686.59	2.56
300	686.66	2.75
1500	686.67	2.78
3600	686.72	2.93
7200	686.78	3.10
14400	687.23	4.43

APO

Time	centroid	D
0	685.723	0.01
30	686.516	2.33
45	686.563	2.47
60	686.7005	2.87
300	686.6115	2.61
1500	686.872	3.38
3600	686.9435	3.59
7200	687.029	3.84
14400	687.334	4.73

DNA

Time	centroid	D
0	685.72	0.00
30	686.44	2.12
45	686.53	2.37
60	686.53	2.38
300	686.60	2.57
1500	686.70	2.86
3600	686.73	2.95
7200	686.77	3.08
14400	686.66	2.76

ONPFDNA

Time	centroid	D
0	685.73	0.02
30	686.41	2.03
45	686.58	2.52
60	686.51	2.32
300	686.57	2.49
1500	686.46	2.17
3600	686.84	3.28
7200	686.91	3.47
14400	686.67	2.77

TMG

Time	centroid	D
0	685.73	0.02
30	686.53	2.37
45	686.71	2.89
60	686.89	3.42
300	686.82	3.23
1500	686.85	3.31
3600	687.06	3.93
7200	687.04	3.88
14400	687.27	4.55

control	685.72
infinity	688.791

$$D(t) = \frac{Mt - M0}{M\infty - M0} * N$$

IPTG

Time	Replicate 1	Replicate 2	Replicate 3	Replicate 4	Replicate5	average	SD
0	685.739	685.725				685.73	0.01
30	686.701	686.422				686.56	0.20
45	686.794	686.653				686.72	0.10
60	686.853	686.683				686.77	0.12
300	687.069	686.592				686.83	0.34
1500	686.957	686.62				686.79	0.24
3600	687.086	686.9				686.99	0.13
7200	687.274	686.953				687.11	0.23
14400	687.454	687.039				687.25	0.29

ONPF

Time	Replicate 1	Replicate 2	Replicate 3	Replicate 4	average	SD
0	685.737	685.744			685.74	0.00
30	686.329	686.245			686.29	0.06
45	686.781	686.379			686.58	0.28
60	686.603	686.584			686.59	0.01
300	686.894	686.421			686.66	0.33
1500	686.807	686.532			686.67	0.19
3600	686.86	686.582			686.72	0.20
7200	686.889	686.666			686.78	0.16
14400	687.231				687.23	

APO

Time	Replicate 1	Replicate 2	Replicate 3	Replicate 4	Replicate 5	average	SD
0	685.717	685.729				685.72	0.01
30	686.66	686.372				686.52	0.20
45	686.719	686.407				686.56	0.22
60	686.797	686.604				686.70	0.14
300	686.807	686.416				686.61	0.28
1500	687.002	686.742				686.87	0.18
3600	687.067	686.82				686.94	0.17
7200	687.237	686.821				687.03	0.29
14400	687.594	687.074				687.33	0.37

DNA

Time	Replicate 1	Replicate 2	Replicate 3	Replicate 4	Replicate 5	average	SD
0	685.706	685.732				685.72	0.02
30	686.472	686.417				686.44	0.04
45	686.596	686.464				686.53	0.09
60	686.552	686.511				686.53	0.03
300	686.64	686.553				686.60	0.06
1500	686.669	686.723				686.70	0.04
3600	686.876	686.577				686.73	0.21
7200	686.947	686.595				686.77	0.25
14400	687.122	686.199				686.66	0.65

ONPFDNA

Time	Replicate 1	Replicate 2	Replicate 3	Replicate 4	average	SD
0	685.725	685.729			685.73	0.00
30	686.4	686.424			686.41	0.02
45	686.592	686.571			686.58	0.01
60	686.51	686.513			686.51	0.00
300	686.623	686.515			686.57	0.08
1500	686.382	686.536			686.46	0.11
3600	686.897	686.781			686.84	0.08
7200	687.039	686.772			686.91	0.19
14400		686.666			686.67	

TMG

Time	Replicate 1	Replicate 2	Replicate 3	Replicate 4	Replicate 5	average	SD
0	685.734	685.723				685.73	0.01
30	686.306	686.751				686.53	0.31
45	686.702	686.708				686.71	0.00
60	686.747	687.026				686.89	0.20
300		686.821				686.82	
1500	686.686	687.01				686.85	0.23
3600		687.062				687.06	
7200	686.877	687.21				687.04	0.24
14400	687.174	687.369				687.27	0.14

213-225 IAEREGDWSAMSG

Charge 2

IPTG

Time	centroid	D
0	705.219	-0.04
30	705.9905	2.67
45	706.129	3.15
60	706.229	3.50
300	706.506	4.48
1500	706.5925	4.78
3600	706.63	4.91
7200	706.6635	5.03
14400	706.619	4.87

ONPF

Time	centroid	D
0	705.2605	0.11
30	705.61	1.33
45	705.9085	2.38
60	706.047	2.87
300	706.136	3.18
1500	706.367	3.99
3600	706.4005	4.11
7200	706.298	3.75
14400	706.23	3.51

APO

Time	centroid	D
0	705.229	0.00
30	705.7945	1.98
45	705.906	2.37
60	706.0675	2.94
300	706.233	3.52
1500	706.5695	4.70
3600	706.6575	5.01
7200	706.6065	4.83
14400	706.862	5.72

DNA

Time	centroid	D
0	705.23	0.00
30	705.7125	1.69
45	705.916	2.41
60	705.983	2.64
300	706.1335	3.17
1500	706.442	4.25
3600	706.378	4.03
7200	706.319	3.82
14400	706.2385	3.54

ONPFDNA

Time	centroid	D
0	705.2265	-0.01
30	705.6585	1.50
45	705.8425	2.15
60	705.9445	2.51
300	706.0315	2.81
1500	706.0985	3.05
3600	706.4995	4.45
7200	706.462	4.32
14400	706.0775	2.97

TMG

Time	centroid	D
0	705.2365	0.02
30	705.962	2.57
45	706.0965	3.04
60	706.407	4.13
300	706.5195	4.52
1500	706.633	4.92
3600	706.6935	5.13
7200	706.645	4.96
14400	706.793	5.48

control 705.23
infinity 708.366

$$D(t) = \frac{M_t - M_0}{M_\infty - M_0} * N$$

IPTG

Time	Replicate 1	Replicate 2	Replicate 3	Replicate 4	Replicate5	average	SD
0	705.221	705.217				705.22	0.00
30	706.213	705.768				705.99	0.31
45	706.275	705.983				706.13	0.21
60	706.301	706.157				706.23	0.10
300	706.713	706.299				706.51	0.29
1500	706.836	706.349				706.59	0.34
3600	706.857	706.403				706.63	0.32
7200	706.885	706.442				706.66	0.31
14400	706.873	706.365				706.62	0.36

ONPF

Time	Replicate 1	Replicate 2	Replicate 3	Replicate 4	average	SD
0	705.247	705.274			705.26	0.02
30	705.71	705.51			705.61	0.14
45	706.146	705.671			705.91	0.34
60	706.082	706.012			706.05	0.05
300	706.351	705.921			706.14	0.30
1500	706.585	706.149			706.37	0.31
3600	706.591	706.21			706.40	0.27
7200	706.435	706.161			706.30	0.19
14400	706.867	705.592			706.23	0.90

APO

Time	Replicate 1	Replicate 2	Replicate 3	Replicate 4	Replicate 5	average	SD
0	705.218	705.24				705.23	0.02
30	705.905	705.684				705.79	0.16
45	706.069	705.743				705.91	0.23
60	706.085	706.05				706.07	0.02
300	706.496	705.97				706.23	0.37
1500	706.786	706.353				706.57	0.31
3600	706.876	706.439				706.66	0.31
7200	706.918	706.295				706.61	0.44
14400	707.221	706.503				706.86	0.51

DNA

Time	Replicate 1	Replicate 2	Replicate 3	Replicate 4	Replicate 5	average	SD
0	705.224	705.236				705.23	0.01
30	705.768	705.657				705.71	0.08
45	706.105	705.727				705.92	0.27
60	706	705.966				705.98	0.02
300	706.209	706.058				706.13	0.11
1500	706.438	706.446				706.44	0.01
3600	706.601	706.155				706.38	0.32
7200	706.516	706.122				706.32	0.28
14400	706.719	705.758				706.24	0.68

ONPFDNA

Time	Replicate 1	Replicate 2	Replicate 3	Replicate 4	average	SD
0	705.233	705.22			705.23	0.01
30	705.735	705.582			705.66	0.11
45	705.961	705.724			705.84	0.17
60	705.963	705.926			705.94	0.03
300	706.153	705.91			706.03	0.17
1500	706.036	706.161			706.10	0.09
3600	706.571	706.428			706.50	0.10
7200	706.466	706.458			706.46	0.01
14400	706.122	706.033			706.08	0.06

TMG

Time	Replicate 1	Replicate 2	Replicate 3	Replicate 4	Replicate 5	average	SD
0	705.243	705.23				705.24	0.01
30	705.781	706.143				705.96	0.26
45	706.169	706.024				706.10	0.10
60	706.211	706.603				706.41	0.28
300	706.423	706.616				706.52	0.14
1500	706.407	706.859				706.63	0.32
3600	706.532	706.855				706.69	0.23
7200	706.418	706.872				706.65	0.32
14400	706.623	706.963				706.79	0.24

216-226 REGDWSAMSGF

Charge 2

IPTG

Time	centroid	D
0	622.13	-0.05
30	622.40	0.91
45	622.52	1.32
60	622.69	1.93
300	622.82	2.36
1500	622.98	2.93
3600	622.98	2.93
7200	622.99	2.98
14400	623.01	3.04

ONPF

Time	centroid	D
0	622.16	0.06
30	622.33	0.65
45	622.63	1.73
60	622.62	1.69
300	622.63	1.72
1500	622.92	2.73
3600	623.20	3.71
7200	622.85	2.50
14400	623.07	3.27

APO

Time	centroid	D
0	622.1428	0.01
30	622.3478	0.73
45	622.415	0.96
60	622.5756	1.53
300	622.7176	2.02
1500	622.925	2.75
3600	622.9746	2.92
7200	622.971	2.91
14400	623.1148	3.41

DNA

Time	centroid	D
0	622.13	-0.04
30	622.30	0.56
45	622.45	1.07
60	622.55	1.43
300	622.60	1.62
1500	622.80	2.30
3600	622.84	2.45
7200	622.74	2.11
14400	622.76	2.17

ONPFDNA

Time	centroid	D
0	622.06	-0.28
30	622.24	0.36
45	622.35	0.72
60	622.49	1.23
300	622.55	1.44
1500	622.68	1.89
3600	622.91	2.70
7200	622.90	2.65
14400	622.77	2.21

TMG

Time	centroid	D
0	622.09	-0.16
30	622.45	1.08
45	622.60	1.59
60	622.80	2.30
300	622.95	2.82
1500	623.09	3.34
3600	623.20	3.72
7200	623.01	3.04
14400	623.24	3.85

control 622.14
infinity 624.71

$$D(t) = \frac{Mt - M0}{M\infty - M0} * N$$

IPTG

Time	Replicate 1	Replicate 2	Replicate 3	Replicate 4	Replicate5	average	SD
0	622.101	622.1	622.188	622.119	622.122	622.13	0.04
30	622.597	622.324	622.35	622.39	622.342	622.40	0.11
45	622.641	622.392				622.52	0.18
60	622.679	622.566	622.879	622.665	622.661	622.69	0.11
300	623.032	622.648	622.949	622.819	622.628	622.82	0.18
1500	623.233	622.828	623.094	622.903	622.831	622.98	0.18
3600	623.17	623.014		622.883	622.842	622.98	0.15
7200	623.215		622.967	622.923	622.864	622.99	0.15
14400	623.198	622.936	623.084	622.952	622.865	623.01	0.13

ONPF

Time	Replicate 1	Replicate 2	Replicate 3	Replicate 4	average	SD
0	622.128	622.168	622.168	622.168	622.16	0.02
30	622.285	622.369	622.374	622.275	622.33	0.05
45	622.633				622.63	
60	622.574	622.614	622.837	622.46	622.62	0.16
300	622.775	622.748	622.537	622.466	622.63	0.15
1500	623.018	622.862	623.026	622.769	622.92	0.13
3600	623.01	623.034	623.072	623.677	623.20	0.32
7200	622.909	622.897	622.958	622.649	622.85	0.14
14400	623.209	623.09	623.094	622.898	623.07	0.13

APO

Time	Replicate 1	Replicate 2	Replicate 3	Replicate 4	Replicate 5	average	SD
0	622.116	622.116	622.146	622.168	622.168	622.14	0.03
30	622.283	622.314	622.462	622.332		622.35	0.08
45	622.471	622.359				622.42	0.08
60	622.464	622.573	622.674	622.593	622.574	622.58	0.07
300	622.792	622.605	622.871	622.737	622.583	622.72	0.12
1500	623.105	622.903	622.988	622.837	622.792	622.93	0.12
3600	623.211	622.931	623.089	622.878	622.764	622.97	0.18
7200	623.237	622.812		623.027	622.808	622.97	0.20
14400	623.45	622.999		623.039	622.971	623.11	0.23

DNA

Time	Replicate 1	Replicate 2	Replicate 3	Replicate 4	Replicate 5	average	SD
0	622.115	622.105	622.125	622.168		622.13	0.03
30	622.311	622.275	622.334	622.275		622.30	0.03
45	622.611	622.28				622.45	0.23
60	622.538	622.499	622.721	622.433		622.55	0.12
300	622.675	622.564	622.71	622.463		622.60	0.11
1500	622.935	622.912	623.005	622.339		622.80	0.31
3600		622.697	622.985			622.84	0.20
7200	622.952	622.74	622.893	622.383		622.74	0.26
14400	623.222	622.427	623.061	622.323		622.76	0.45

ONPFDNA

Time	Replicate 1	Replicate 2	Replicate 3	Replicate 4	average	SD
0	622.123	621.96	622.054	622.102	622.06	0.07
30	622.308	622.091	622.372	622.205	622.24	0.12
45	622.547	622.143			622.35	0.29
60	622.489	622.298	622.671	622.509	622.49	0.15
300	622.627	622.419	622.623	622.532	622.55	0.10
1500	622.615	622.585	622.821	622.698	622.68	0.11
3600	623.065		622.891	622.775	622.91	0.15
7200	622.941			622.855	622.90	0.06
14400	622.74	622.625		622.944	622.77	0.16

TMG

Time	Replicate 1	Replicate 2	Replicate 3	Replicate 4	Replicate 5	average	SD
0	622.079	622.108				622.09	0.02
30	622.309	622.589				622.45	0.20
45	622.718	622.472				622.60	0.17
60	622.67	622.923				622.80	0.18
300		622.945				622.95	
1500	622.983	623.202				623.09	0.15
3600		623.201				623.20	
7200	622.808	623.208				623.01	0.28
14400	623.111	623.368				623.24	0.18

219-223 DWSAM

Charge 1

IPTG

Time	centroid	D
0	609.56	-0.03
30	610.01	0.63
45	610.09	0.75
60	610.17	0.87
300	610.60	1.49
1500	610.67	1.60
3600	610.79	1.77
7200	610.75	1.72
14400	610.75	1.71

ONPF

Time	centroid	D
0	609.56	-0.02
30	609.71	0.20
45	609.88	0.44
60	610.01	0.63
300	610.24	0.97
1500	610.51	1.37
3600	610.55	1.42
7200	610.50	1.35
14400	610.88	1.90

APO

Time	centroid	D
0	609.57	0.00
30	609.82	0.35
45	609.91	0.49
60	610.04	0.68
300	610.37	1.16
1500	610.72	1.66
3600	610.78	1.75
7200	610.75	1.72
14400	610.94	1.99

DNA

Time	centroid	D
0	609.5455	-0.04
30	609.7255	0.22
45	609.883	0.45
60	609.955	0.56
300	610.2135	0.93
1500	610.5075	1.36
3600	610.507	1.36
7200	610.458	1.29
14400	610.812	1.80

ONPFDNA

Time	centroid	D
0	609.55	-0.04
30	609.68	0.15
45	609.76	0.27
60	609.92	0.50
300	610.08	0.74
1500	610.22	0.95
3600	610.60	1.50
7200	610.51	1.37
14400	610.23	0.95

TMG

Time	centroid	D
0	609.55	-0.03
30	609.97	0.57
45	610.04	0.68
60	610.33	1.10
300	610.58	1.46
1500	610.71	1.66
3600	610.75	1.72
7200	610.74	1.70
14400	610.87	1.89

control 609.574
infinity 611.632

$$D(t) = \frac{M_t - M_0}{M_\infty - M_0} \cdot N$$

IPTG

Time	Replicate 1	Replicate 2	Replicate 3	Replicate 4	Replicate5	average	SD
0	609.551	609.559				609.56	0.01
30	610.16	609.858				610.01	0.21
45	610.186	609.986				610.09	0.14
60	610.216	610.127				610.17	0.06
300	610.728	610.464				610.60	0.19
1500	610.846	610.501				610.67	0.24
3600	610.832	610.743				610.79	0.06
7200	610.883	610.623				610.75	0.18
14400	610.878	610.622				610.75	0.18

ONPF

Time	Replicate 1	Replicate 2	Replicate 3	Replicate 4	average	SD
0	609.54	609.579			609.56	0.03
30	609.746	609.677			609.71	0.05
45	609.984	609.772			609.88	0.15
60	609.963	610.053			610.01	0.06
300	610.43	610.042			610.24	0.27
1500	610.698	610.33			610.51	0.26
3600	610.718	610.377			610.55	0.24
7200	610.607	610.393			610.50	0.15
14400	610.879				610.88	

APO

Time	Replicate 1	Replicate 2	Replicate 3	Replicate 4	Replicate 5	average	SD
0	609.567	609.581				609.57	0.01
30	609.847	609.787				609.82	0.04
45	609.979	609.842				609.91	0.10
60	609.984	610.092				610.04	0.08
300	610.546	610.199				610.37	0.25
1500	610.828	610.602				610.72	0.16
3600	610.949	610.605				610.78	0.24
7200	610.991	610.513				610.75	0.34
14400	611.171	610.703				610.94	0.33

DNA

Time	Replicate 1	Replicate 2	Replicate 3	Replicate 4	Replicate 5	average	SD
0	609.529	609.562				609.55	0.02
30	609.751	609.7				609.73	0.04
45	610.028	609.738				609.88	0.21
60	609.905	610.005				609.96	0.07
300	610.266	610.161				610.21	0.07
1500	610.491	610.524				610.51	0.02
3600	610.65	610.364				610.51	0.20
7200	610.6	610.316				610.46	0.20
14400	610.812					610.81	

ONPFDNA

Time	Replicate 1	Replicate 2	Replicate 3	Replicate 4	average	SD
0	609.541	609.551			609.55	0.01
30	609.695	609.661			609.68	0.02
45	609.812	609.713			609.76	0.07
60	609.915	609.917			609.92	0.00
300	610.168	609.998			610.08	0.12
1500	610.12	610.326			610.22	0.15
3600	610.66	610.54			610.60	0.08
7200	610.509	610.517			610.51	0.01
14400	610.162	610.289			610.23	0.09

TMG

Time	Replicate 1	Replicate 2	Replicate 3	Replicate 4	Replicate 5	average	SD
0	609.524	609.578				609.55	0.04
30	609.877	610.055				609.97	0.13
45	610.123	609.961				610.04	0.11
60	610.136	610.526				610.33	0.28
300	610.523	610.634				610.58	0.08
1500	610.564	610.861				610.71	0.21
3600	610.626	610.879				610.75	0.18
7200	610.536	610.95				610.74	0.29
14400	610.744	610.992				610.87	0.18

220-223 WSAM

Charge 1

IPTG

Time	centroid	D
0	494.48	0.00
30	494.90	0.56
45	494.98	0.67
60	495.06	0.77
300	495.47	1.31
1500	495.54	1.39
3600	495.66	1.55
7200	495.61	1.49
14400	495.59	1.46

ONPF

Time	centroid	D
0	494.52	0.05
30	494.63	0.20
45	494.82	0.45
60	494.93	0.59
300	495.12	0.84
1500	495.38	1.19
3600	495.47	1.30
7200	495.48	1.32
14400	495.52	1.37

APO

Time	centroid	D
0	494.48	0.00
30	494.72	0.32
45	494.82	0.45
60	494.95	0.62
300	495.27	1.05
1500	495.62	1.51
3600	495.63	1.51
7200	495.63	1.51
14400	495.60	1.48

DNA

Time	centroid	D
0	494.51	0.04
30	494.81	0.43
45	494.82	0.45
60	494.89	0.54
300	495.14	0.87
1500	495.49	1.33
3600	495.33	1.13
7200	495.35	1.14
14400	495.75	1.68

ONPFDNA

Time	centroid	D
0	494.47	-0.01
30	494.58	0.14
45	494.67	0.26
60	494.87	0.52
300	494.94	0.61
1500	495.13	0.86
3600	495.43	1.25
7200	495.38	1.19
14400	495.25	1.01

TMG

Time	centroid	D
0	494.50	0.03
30	494.95	0.62
45	495.00	0.68
60	495.18	0.92
300	495.47	1.30
1500	495.60	1.48
3600	495.65	1.54
7200	495.68	1.58
14400	495.72	1.64

control 494.4775
infinity 495.999

$$D(t) = \frac{M_t - M_0}{M_\infty - M_0} * N$$

IPTG

Time	Replicate 1	Replicate 2	Replicate 3	Replicate 4	Replicate5	average	SD
0	494.508	494.448				494.48	0.04
30	495.085	494.715				494.90	0.26
45	495.108	494.859				494.98	0.18
60	495.122	495.006				495.06	0.08
300	495.607	495.338				495.47	0.19
1500	495.732	495.34				495.54	0.28
3600	495.72	495.595				495.66	0.09
7200	495.76	495.461				495.61	0.21
14400	495.712	495.459				495.59	0.18

ONPF

Time	Replicate 1	Replicate 2	Replicate 3	Replicate 4	average	SD
0	494.578	494.452			494.52	0.09
30	494.711	494.553			494.63	0.11
45	495.003	494.63			494.82	0.26
60	494.925	494.928			494.93	0.00
300	495.324	494.911			495.12	0.29
1500	495.594	495.174			495.38	0.30
3600	495.644	495.294			495.47	0.25
7200	495.479				495.48	
14400	495.638	495.397			495.52	0.17

APO

Time	Replicate 1	Replicate 2	Replicate 3	Replicate 4	Replicate 5	average	SD
0	494.506	494.449				494.48	0.04
30	494.784	494.659				494.72	0.09
45	494.954	494.693				494.82	0.18
60	494.934	494.964				494.95	0.02
300	495.464	495.083				495.27	0.27
1500	495.763	495.484				495.62	0.20
3600	495.787	495.468				495.63	0.23
7200	495.625					495.63	
14400	495.838	495.367				495.60	0.33

DNA

Time	Replicate 1	Replicate 2	Replicate 3	Replicate 4	Replicate 5	average	SD
0	494.573	494.442				494.51	0.09
30	495.039	494.577				494.81	0.33
45	495.03	494.608				494.82	0.30
60	494.913	494.862				494.89	0.04
300	495.261	495.019				495.14	0.17
1500	495.487					495.49	
3600	495.55	495.117				495.33	0.31
7200	495.55	495.14				495.35	0.29
14400	495.754					495.75	

ONPFDNA

Time	Replicate 1	Replicate 2	Replicate 3	Replicate 4	average	SD
0	494.492	494.441			494.47	0.04
30	494.634	494.534			494.58	0.07
45	494.756	494.592			494.67	0.12
60	494.941	494.8			494.87	0.10
300	495.045	494.84			494.94	0.14
1500	495.088	495.173			495.13	0.06
3600	495.449	495.408			495.43	0.03
7200	495.404	495.365			495.38	0.03
14400	495.247				495.25	

TMG

Time	Replicate 1	Replicate 2	Replicate 3	Replicate 4	Replicate 5	average	SD
0	494.502	494.5				494.50	0.00
30	494.946	494.953				494.95	0.00
45	495.092	494.9				495.00	0.14
60	495.06	495.297				495.18	0.17
300	495.432	495.5				495.47	0.05
1500	495.503	495.698				495.60	0.14
3600	495.535	495.766				495.65	0.16
7200		495.678				495.68	
14400	495.604	495.839				495.72	0.17

221-227 SAMSGFQ

Charge 1

IPTG

Time	centroid	D
0	727.74	-0.06
30	728.45	0.88
45	728.65	1.15
60	728.95	1.55
300	729.18	1.85
1500	729.55	2.35
3600	729.41	2.17
7200	729.47	2.24
14400	729.42	2.18

ONPF

Time	centroid	D
0	727.83	0.05
30	727.94	0.20
45	#DIV/0!	#DIV/0!
60	728.65	1.15
300	728.66	1.17
1500	729.22	1.91
3600	729.14	1.80
7200	729.04	1.66
14400	729.41	2.17

APO

Time	centroid	D
0	727.79	-0.01
30	728.23	0.59
45	728.39	0.80
60	728.43	0.86
300	728.93	1.52
1500	729.32	2.04
3600	729.41	2.16
7200	729.36	2.09
14400	729.54	2.33

DNA

Time	centroid	D
0	727.80	0.01
30	728.10	0.41
45	728.38	0.78
60	728.70	1.21
300	728.60	1.08
1500	729.12	1.77
3600	729.21	1.90
7200	729.01	1.63
14400	729.30	2.01

ONPFDNA

Time	centroid	D
0	727.79	0.00
30	728.10	0.41
45	728.28	0.65
60	728.48	0.92
300	728.54	1.00
1500	728.85	1.41
3600	729.22	1.91
7200	729.20	1.88
14400	729.00	1.61

TMG

Time	centroid	D
0	727.85	0.09
30	728.49	0.94
45	728.73	1.26
60	729.13	1.79
300	729.21	1.89
1500	729.38	2.13
3600	729.43	2.19
7200	729.49	2.27
14400	729.51	2.29

control 727.79
infinity 731.537

$D(t) = \frac{M_t - M_0}{M_\infty - M_0} * N$

IPTG

Time	Replicate 1	Replicate 2	Replicate 3	Replicate 4	Replicate5	average	SD
0	727.734	727.753				727.74	0.01
30	728.715	728.188				728.45	0.37
45	728.723	728.583				728.65	0.10
60	728.824	729.083				728.95	0.18
300	729.372	728.982				729.18	0.28
1500	729.555	729.549				729.55	0.00
3600	729.544	729.282				729.41	0.19
7200	729.467					729.47	
14400	729.429	729.412				729.42	0.01

ONPF

Time	Replicate 1	Replicate 2	Replicate 3	Replicate 4	average	SD
0	727.815	727.843			727.83	0.02
30	728.06	727.816			727.94	0.17
45						
60	728.557	728.752			728.65	0.14
300	728.984	728.344			728.66	0.45
1500	729.332	729.107			729.22	0.16
3600	729.167	729.108			729.14	0.04
7200	729.152	728.919			729.04	0.16
14400	729.414				729.41	

APO

Time	Replicate 1	Replicate 2	Replicate 3	Replicate 4	Replicate 5	average	SD
0	727.759	727.813				727.79	0.04
30	728.168	728.291				728.23	0.09
45	728.473	728.313				728.39	0.11
60	728.324	728.541				728.43	0.15
300	729.091	728.765				728.93	0.23
1500	729.362	729.272				729.32	0.06
3600	729.667	729.151				729.41	0.36
7200	729.54	729.172				729.36	0.26
14400	729.916	729.162				729.54	0.53

DNA

Time	Replicate 1	Replicate 2	Replicate 3	Replicate 4	Replicate 5	average	SD
0	727.817	727.773				727.80	0.03
30	728.055	728.146				728.10	0.06
45	728.61	728.145				728.38	0.33
60		728.695				728.70	
300	728.594	728.6				728.60	0.00
1500	729.103	729.131				729.12	0.02
3600	729.318	729.109				729.21	0.15
7200	729.075	728.941				729.01	0.09
14400	729.299					729.30	

ONPFDNA

Time	Replicate 1	Replicate 2	Replicate 3	Replicate 4	average	SD
0	727.846	727.729			727.79	0.08
30	728.079	728.118			728.10	0.03
45	728.355	728.204			728.28	0.11
60	728.555	728.401			728.48	0.11
300	728.658	728.415			728.54	0.17
1500	728.807	728.883			728.85	0.05
3600	729.24	729.196			729.22	0.03
7200	729.202	729.196			729.20	0.00
14400	728.934	729.065			729.00	0.09

TMG

Time	Replicate 1	Replicate 2	Replicate 3	Replicate 4	Replicate 5	average	SD
0	727.884	727.825				727.85	0.04
30	728.347	728.636				728.49	0.20
45	728.936	728.528				728.73	0.29
60	728.987	729.273				729.13	0.20
300	729.236	729.176				729.21	0.04
1500	729.296	729.47				729.38	0.12
3600	729.367	729.499				729.43	0.09
7200		729.492				729.49	
14400	729.436	729.579				729.51	0.10

224-232 SGFQQTMQM

Charge 2

IPTG

Time	centroid	D
0	529.54	0.00
30	529.60	0.19
45	529.61	0.22
60	529.61	0.23
300	529.61	0.21
1500	529.61	0.22
3600	529.62	0.26
7200	529.65	0.34
14400	529.71	0.54

ONPF

Time	centroid	D
0	529.56	0.06
30	529.61	0.23
45	529.65	0.34
60	529.65	0.36
300	529.62	0.25
1500	529.64	0.30
3600	529.66	0.39
7200	529.66	0.38
14400	529.71	0.54

APO

Time	centroid	D
0	529.54	-0.01
30	529.58	0.12
45	529.61	0.21
60	529.63	0.29
300	529.60	0.18
1500	529.62	0.24
3600	529.62	0.26
7200	529.63	0.29
14400	529.75	0.67

DNA

Time	centroid	D
0	529.55	0.04
30	529.60	0.19
45	529.58	0.13
60	529.60	0.19
300	529.61	0.21
1500	529.63	0.27
3600	529.61	0.22
7200	529.63	0.29
14400	529.68	0.44

ONPFDNA

Time	centroid	D
0	529.55	0.02
30	529.59	0.15
45	529.61	0.22
60	529.63	0.29
300	529.58	0.13
1500	529.61	0.23
3600	529.61	0.23
7200	529.64	0.33
14400	529.62	0.25

TMG

Time	centroid	D
0	529.55	0.04
30	529.63	0.27
45	529.64	0.31
60	529.65	0.35
300	529.66	0.37
1500	529.65	0.35
3600	529.67	0.42
7200	529.68	0.44
14400	529.75	0.65

control	529.54
infinity	531.777

$$D(t) = \frac{M_t - M_0}{M_\infty - M_0} * N$$

IPTG

Time	Replicate 1	Replicate 2	Replicate 3	Replicate 4	Replicate5	average	SD
0	529.532	529.549				529.54	0.01
30	529.632	529.572				529.60	0.04
45	529.644	529.577				529.61	0.05
60	529.631	529.598				529.61	0.02
300	529.643	529.574				529.61	0.05
1500	529.617	529.603				529.61	0.01
3600	529.64	529.606				529.62	0.02
7200	529.671	529.624				529.65	0.03
14400	529.746	529.677				529.71	0.05

ONPF

Time	Replicate 1	Replicate 2	Replicate 3	Replicate 4	average	SD
0	529.565	529.576	529.541		529.56	0.02
30	529.632	529.591	529.616		529.61	0.02
45	529.683	529.613			529.65	0.05
60	529.626	529.623	529.713		529.65	0.05
300	529.614	529.609	529.632		529.62	0.01
1500	529.629	529.619	529.658		529.64	0.02
3600	529.687	529.629	529.675		529.66	0.03
7200	529.666	529.61	529.706		529.66	0.05
14400	529.685		529.737		529.71	0.04

APO

Time	Replicate 1	Replicate 2	Replicate 3	Replicate 4	Replicate 5	average	SD
0	529.528	529.548				529.54	0.01
30	529.57	529.588				529.58	0.01
45	529.583	529.63				529.61	0.03
60	529.583	529.682				529.63	0.07
300	529.592	529.605				529.60	0.01
1500	529.607	529.624				529.62	0.01
3600	529.628	529.619				529.62	0.01
7200	529.642	529.625				529.63	0.01
14400	529.794	529.712				529.75	0.06

DNA

Time	Replicate 1	Replicate 2	Replicate 3	Replicate 4	Replicate 5	average	SD
0	529.541	529.564				529.55	0.02
30	529.622	529.582				529.60	0.03
45	529.58	529.585				529.58	0.00
60	529.6	529.6				529.60	0.00
300	529.633	529.582				529.61	0.04
1500	529.606	529.647				529.63	0.03
3600	529.591	529.631				529.61	0.03
7200	529.656	529.612				529.63	0.03
14400	529.737	529.625				529.68	0.08

ONPFDNA

Time	Replicate 1	Replicate 2	Replicate 3	Replicate 4	average	SD
0	529.564	529.531			529.55	0.02
30	529.587	529.586			529.59	0.00
45	529.639	529.581			529.61	0.04
60	529.634				529.63	
300	529.592	529.57			529.58	0.02
1500	529.591	529.638			529.61	0.03
3600	529.623	529.606			529.61	0.01
7200	529.67	529.619			529.64	0.04
14400	529.597	529.64			529.62	0.03

TMG

Time	Replicate 1	Replicate 2	Replicate 3	Replicate 4	Replicate 5	average	SD
0	529.569	529.537				529.55	0.02
30	529.617	529.633				529.63	0.01
45	529.674	529.607				529.64	0.05
60	529.652					529.65	
300	529.67	529.644				529.66	0.02
1500	529.627	529.679				529.65	0.04
3600	529.686	529.661				529.67	0.02
7200	529.691	529.669				529.68	0.02
14400	529.749	529.748				529.75	0.00

228-233 QTMQML

Charge 1

IPTG

Time	centroid	D
0	751.85	-0.01
30	751.90	0.05
45	751.91	0.06
60	751.92	0.07
300	751.90	0.06
1500	751.93	0.09
3600	751.95	0.11
7200	752.01	0.19
14400	752.09	0.29

ONPF

Time	centroid	D
0	751.86	0.00
30	751.88	0.02
45	751.94	0.10
60	751.92	0.07
300	751.91	0.07
1500	751.92	0.07
3600	751.94	0.11
7200	751.97	0.14
14400	752.05	0.24

APO

Time	centroid	D
0	751.86	-0.01
30	751.90	0.05
45	751.89	0.04
60	751.91	0.06
300	751.91	0.06
1500	751.96	0.13
3600	751.95	0.11
7200	752.02	0.20
14400	752.15	0.37

DNA

Time	centroid	D
0	751.83	-0.04
30	751.89	0.04
45	751.91	0.06
60	751.90	0.06
300	751.91	0.06
1500	751.93	0.08
3600	751.91	0.06
7200	751.96	0.12
14400	751.94	0.11

ONPFDNA

Time	centroid	D
0	751.84	-0.03
30	751.89	0.03
45	751.89	0.04
60	751.92	0.08
300	751.90	0.05
1500	751.93	0.09
3600	751.93	0.09
7200	751.95	0.11
14400	751.95	0.11

TMG

Time	centroid	D
0	751.85	-0.02
30	751.90	0.06
45	751.94	0.10
60	751.91	0.06
300	751.92	0.08
1500	751.95	0.11
3600	751.95	0.12
7200	752.00	0.18
14400	752.08	0.28

control 751.86
infinity 755.01

$$D(t) = \frac{M_t - M_0}{M_\infty - M_0} * N$$

IPTG

Time	Replicate 1	Replicate 2	Replicate 3	Replicate 4	Replicate5	average	SD
0	751.854	751.854				751.85	0.00
30	751.913	751.887				751.90	0.02
45	751.916	751.897				751.91	0.01
60	751.93	751.901				751.92	0.02
300	751.914	751.895				751.90	0.01
1500	751.928	751.94				751.93	0.01
3600	751.944	751.949				751.95	0.00
7200	752.027	751.995				752.01	0.02
14400	752.134	752.045				752.09	0.06

ONPF

Time	Replicate 1	Replicate 2	Replicate 3	Replicate 4	average	SD
0	751.845	751.875			751.86	0.02
30	751.857	751.899			751.88	0.03
45	751.951	751.921			751.94	0.02
60	751.904	751.931			751.92	0.02
300	751.916	751.911			751.91	0.00
1500	751.911	751.925			751.92	0.01
3600	751.952	751.935			751.94	0.01
7200	751.978	751.957			751.97	0.01
14400	752.047				752.05	0.00

APO

Time	Replicate 1	Replicate 2	Replicate 3	Replicate 4	Replicate 5	average	SD
0	751.849	751.863				751.86	0.01
30	751.892	751.904				751.90	0.01
45	751.881	751.9				751.89	0.01
60	751.883	751.931				751.91	0.03
300	751.897	751.921				751.91	0.02
1500	751.953	751.973				751.96	0.01
3600	751.934	751.957				751.95	0.02
7200	752.05	751.988				752.02	0.04
14400	752.217	752.083				752.15	0.09

DNA

Time	Replicate 1	Replicate 2	Replicate 3	Replicate 4	Replicate 5	average	SD
0	751.806	751.856				751.83	0.04
30	751.887	751.898				751.89	0.01
45		751.909				751.91	
60	751.888	751.919				751.90	0.02
300	751.904	751.911				751.91	0.00
1500	751.918	751.932				751.93	0.01
3600	751.904	751.917				751.91	0.01
7200	751.954	751.956				751.96	0.00
14400	751.973	751.916				751.94	0.04

ONPFDNA

Time	Replicate 1	Replicate 2	Replicate 3	Replicate 4	average	SD
0	751.819	751.86			751.84	0.03
30	751.88	751.89			751.89	0.01
45	751.88	751.901			751.89	0.01
60	751.934	751.911			751.92	0.02
300	751.907	751.898			751.90	0.01
1500	751.905	751.951			751.93	0.03
3600	751.937	751.925			751.93	0.01
7200	751.945	751.954			751.95	0.01
14400	751.913	751.987			751.95	0.05

TMG

Time	Replicate 1	Replicate 2	Replicate 3	Replicate 4	Replicate 5	average	SD
0	751.836	751.856				751.85	0.01
30	751.879	751.929				751.90	0.04
45	751.964	751.908				751.94	0.04
60	751.905					751.91	
300	751.927	751.917				751.92	0.01
1500	751.933	751.968				751.95	0.02
3600	751.963	751.943				751.95	0.01
7200	752.017	751.982				752.00	0.02
14400	752.089	752.072				752.08	0.01

234-243 NEGIVPTAML

Charge 1

IPTG

Time	centroid	D
0	1045.24	0.03
30	1045.75	0.67
45	1045.89	0.84
60	1046.00	0.98
300	1046.22	1.26
1500	1046.82	2.01
3600	1047.17	2.45
7200	1047.22	2.52
14400	1047.20	2.49

ONPF

Time	centroid	D
0	1045.35	0.16
30	1045.56	0.43
45	1045.94	0.90
60	1046.12	1.13
300	1046.08	1.08
1500	1046.57	1.71
3600	1046.76	1.94
7200	1046.74	1.92
14400	1047.20	2.49

APO

Time	centroid	D
0	1045.22	0.00
30	1045.64	0.53
45	1045.80	0.73
60	1046.03	1.03
300	1046.15	1.17
1500	1046.87	2.08
3600	1047.07	2.33
7200	1047.08	2.34
14400	1047.47	2.83

DNA

Time	centroid	D
0	1045.27	0.07
30	1045.62	0.51
45	1046.12	1.13
60	1046.19	1.23
300	1046.21	1.25
1500	1046.66	1.82
3600	1046.81	2.01
7200	1046.71	1.88
14400	1046.68	1.84

ONPFDNA

Time	centroid	D
0	1045.23	0.01
30	1045.59	0.47
45	1045.87	0.81
60	1046.11	1.12
300	1046.09	1.09
1500	1046.41	1.50
3600	1046.96	2.19
7200	1046.85	2.06
14400	1046.62	1.76

TMG

Time	centroid	D
0	1045.25	0.04
30	1045.74	0.66
45	1046.12	1.13
60	1046.17	1.20
300	1046.30	1.36
1500	1046.75	1.93
3600	1047.18	2.47
7200	1047.12	2.40
14400	1047.43	2.79

control 1045.22
infinity 1050.776

$$D(t) = \frac{M_t - M_0}{M_\infty - M_0} * N$$

IPTG

Time	Replicate 1	Replicate 2	Replicate 3	Replicate 4	Replicate5	average	SD
0	1045.292	1045.197				1045.24	0.07
30	1045.906	1045.601				1045.75	0.22
45	1046.001	1045.78				1045.89	0.16
60	1045.992	1046.004				1046.00	0.01
300	1046.334	1046.113				1046.22	0.16
1500	1046.987	1046.651				1046.82	0.24
3600	1047.28	1047.05				1047.17	0.16
7200	1047.384	1047.055				1047.22	0.23
14400	1047.442	1046.949				1047.20	0.35

ONPF

Time	Replicate 1	Replicate 2	Replicate 3	Replicate 4	average	SD
0	1045.361	1045.329			1045.35	0.02
30	1045.647	1045.474			1045.56	0.12
45	1046.29	1045.582			1045.94	0.50
60	1046.105	1046.132			1046.12	0.02
300	1046.248	1045.903			1046.08	0.24
1500	1046.74	1046.408			1046.57	0.23
3600	1047.053	1046.474			1046.76	0.41
7200	1046.862	1046.627			1046.74	0.17
14400	1047.305	1047.091			1047.20	0.15

APO

Time	Replicate 1	Replicate 2	Replicate 3	Replicate 4	Replicate 5	average	SD
0	1045.198	1045.235				1045.22	0.03
30	1045.678	1045.608				1045.64	0.05
45	1045.893	1045.706				1045.80	0.13
60	1045.904	1046.165				1046.03	0.18
300	1046.268	1046.036				1046.15	0.16
1500	1046.878	1046.858				1046.87	0.01
3600	1047.244	1046.896				1047.07	0.25
7200	1047.334	1046.821				1047.08	0.36
14400	1047.99	1046.947				1047.47	0.74

DNA

Time	Replicate 1	Replicate 2	Replicate 3	Replicate 4	Replicate 5	average	SD
0	1045.29	1045.256				1045.27	0.02
30	1045.65	1045.599				1045.62	0.04
45	1046.551	1045.684				1046.12	0.61
60	1046.211	1046.177				1046.19	0.02
300	1046.328	1046.092				1046.21	0.17
1500	1046.665	1046.657				1046.66	0.01
3600	1047.165	1046.463				1046.81	0.50
7200	1046.929	1046.493				1046.71	0.31
14400	1047.272	1046.089				1046.68	0.84

ONPFDNA

Time	Replicate 1	Replicate 2	Replicate 3	Replicate 4	average	SD
0	1045.27	1045.191			1045.23	0.06
30	1045.672	1045.513			1045.59	0.11
45	1046.056	1045.674			1045.87	0.27
60	1046.116	1046.108			1046.11	0.01
300	1046.191	1045.984			1046.09	0.15
1500	1046.303	1046.526			1046.41	0.16
3600	1047.13	1046.793			1046.96	0.24
7200	1046.881	1046.822			1046.85	0.04
14400	1046.832	1046.398			1046.62	0.31

TMG

Time	Replicate 1	Replicate 2	Replicate 3	Replicate 4	Replicate 5	average	SD
0	1045.284	1045.225				1045.25	0.04
30	1045.625	1045.862				1045.74	0.17
45	1046.387	1045.844				1046.12	0.38
60	1046.153	1046.195				1046.17	0.03
300	1046.3	1046.297				1046.30	0.00
1500	1046.638	1046.865				1046.75	0.16
3600	1047.111	1047.243				1047.18	0.09
7200	1046.887	1047.362				1047.12	0.34
14400	1047.281	1047.586				1047.43	0.22

243-254 LVANDQMALGAM

Charge 2

IPTG

Time	centroid	D
0	617.6978	-0.01
30	617.928	1.00
45	617.828	0.56
60	618.0675	1.61
300	617.9755	1.21
1500	618.0183	1.40
3600	618.0295	1.44
7200	618.1115	1.80
14400	618.1148	1.82

ONPF

Time	centroid	D
0	617.727	0.12
30	617.973	1.20
45	617.879	0.78
60	618.1175	1.83
300	618.0243	1.42
1500	618.0948	1.73
3600	618.1773	2.09
7200	618.1995	2.19
14400	618.26	2.45

APO

Time	centroid	D
0	617.7045	0.02
30	617.9255	0.99
45	617.882	0.80
60	618.037	1.48
300	617.9868	1.26
1500	618.084	1.68
3600	618.1265	1.87
7200	618.2123	2.25
14400	618.2335	2.34

DNA

Time	centroid	D
0	617.7147	0.06
30	617.9227	0.98
45	618.06	1.58
60	618.1287	1.88
300	618.018	1.39
1500	618.129	1.88
3600	618.1537	1.99
7200	618.2137	2.25
14400	618.2507	2.41

ONPFDNA

Time	centroid	D
0	617.80	0.44
30	618.21	2.22
45	617.95	1.11
60	618.43	3.22
300	618.34	2.79
1500	618.47	3.38
3600	618.51	3.57
7200	618.43	3.22
14400	618.55	3.73

TMG

Time	centroid	D
0	617.682	-0.08
30	617.823	0.54
45	617.8655	0.73
60	617.819	0.52
300	617.8135	0.50
1500	617.8	0.44
3600	617.836	0.60
7200	617.896	0.86
14400	617.8235	0.54

control 617.7
infinity 619.981

$$D(t) = \frac{Mt - M0}{M\infty - M0} *N$$

IPTG

Time	Replicate 1	Replicate 2	Replicate 3	Replicate 4	Replicate5	average	SD
0	617.687	617.675	617.712	617.717		617.70	0.02
30	617.75	618.096	617.936	617.93		617.93	0.14
45	617.828					617.83	
60	617.877	618.292	618.066	618.035		618.07	0.17
300	617.726	618.19	618.006	617.98		617.98	0.19
1500	617.811	618.21	618.045	618.007		618.02	0.16
3600	617.867	618.194	618.036	618.021		618.03	0.13
7200	617.923	618.306	618.126	618.091		618.11	0.16
14400	617.946	618.287	618.14	618.086		618.11	0.14

ONPF

Time	Replicate 1	Replicate 2	Replicate 3	Replicate 4	average	SD
0	617.752	617.715	617.727	617.714	617.73	0.02
30	617.959	618.064	617.891	617.978	617.97	0.07
45	617.879				617.88	
60	617.865	618.33	618.087	618.188	618.12	0.20
300	617.914	618.181	617.954	618.048	618.02	0.12
1500	617.759	618.362	618.107	618.151	618.09	0.25
3600	618.004	618.382	618.092	618.231	618.18	0.17
7200	617.99	618.381	618.184	618.243	618.20	0.16
14400	618.074	618.424	618.241	618.293	618.26	0.14

APO

Time	Replicate 1	Replicate 2	Replicate 3	Replicate 4	Replicate 5	average	SD
0	617.61	617.782	617.711	617.715		617.70	0.07
30	617.743	618.146	617.893	617.92		617.93	0.17
45	617.882					617.88	0.00
60	617.716	618.21	618.125	618.097		618.04	0.22
300	617.718	618.222	618.013	617.994		617.99	0.21
1500	617.756	618.293	618.149	618.138		618.08	0.23
3600	617.856	618.362	618.151	618.137		618.13	0.21
7200	617.945	618.432	618.265	618.207		618.21	0.20
14400	617.96	618.464	618.256	618.254		618.23	0.21

DNA

Time	Replicate 1	Replicate 2	Replicate 3	Replicate 4	Replicate 5	average	SD
0	617.707	617.722	617.715			617.71	0.01
30	617.854	618.039	617.875			617.92	0.10
45	618.06					618.06	
60	617.997	618.325	618.064			618.13	0.17
300	617.95	618.165	617.939			618.02	0.13
1500	618.01	618.288	618.089			618.13	0.14
3600	618.088	618.284	618.089			618.15	0.11
7200	618.104	618.337	618.2			618.21	0.12
14400	618.166	618.385	618.201			618.25	0.12

ONPFDNA

Time	Replicate 1	Replicate 2	Replicate 3	Replicate 4	average	SD
0	617.727	617.963	617.714		617.80	0.14
30	617.897	618.849	617.873		618.21	0.56
45	617.954				617.95	
60	617.948	619.345	618.01		618.43	0.79
300	617.866	619.131	618.014		618.34	0.69
1500	617.926	619.477	618.01		618.47	0.87
3600	618.097	619.433	618.012		618.51	0.80
7200	618.103	619.106	618.095		618.43	0.58
14400	617.964	619.596	618.093		618.55	0.91

TMG

Time	Replicate 1	Replicate 2	Replicate 3	Replicate 4	Replicate 5	average	SD
0	617.682	617.682				617.68	0.00
30	617.742	617.904				617.82	0.11
45	617.847	617.884				617.87	0.03
60	617.819					617.82	
300	617.75	617.877				617.81	0.09
1500	617.688	617.912				617.80	0.16
3600	617.781	617.891				617.84	0.08
7200	617.855	617.937				617.90	0.06
14400	617.81	617.837				617.82	0.02

244-255 VANDQMALGAMR

Charge 2

IPTG

Time	centroid	D
0	639.2315	-0.01
30	639.488	0.73
45	639.5035	0.77
60	639.521	0.82
300	639.5005	0.76
1500	639.513	0.80
3600	639.6055	1.07
7200	639.562	0.94
14400	639.5935	1.03

ONPF

Time	centroid	D
0	639.2575	0.07
30	639.4205	0.54
45	639.507	0.78
60	639.518	0.81
300	639.502	0.77
1500	639.5615	0.94
3600	639.602	1.06
7200	639.614	1.09
14400	639.62	1.10

APO

Time	centroid	D
0	639.2295	-0.01
30	639.4315	0.57
45	639.4505	0.62
60	639.4895	0.73
300	639.4635	0.66
1500	639.59	1.02
3600	639.6215	1.11
7200	639.6595	1.22
14400	639.8585	1.79

DNA

Time	centroid	D
0	639.2305	-0.01
30	639.4375	0.58
45	639.516	0.81
60	639.5035	0.77
300	639.492	0.74
1500	639.5335	0.86
3600	639.606	1.07
7200	639.603	1.06
14400	639.623	1.12

ONPFDNA

Time	centroid	D
0	639.23	-0.01
30	639.42	0.53
45	639.48	0.70
60	639.48	0.71
300	639.46	0.65
1500	639.47	0.67
3600	639.58	0.98
7200	639.60	1.04
14400	639.57	0.96

TMG

Time	centroid	D
0	639.2375	0.01
30	639.4845	0.72
45	639.541	0.88
60	639.493	0.74
300	639.5345	0.86
1500	639.613	1.09
3600	639.563	0.94
7200	639.576	0.98
14400	639.6835	1.29

control 639.234
infinity 642.719

$$D(t) = \frac{Mt - M0}{M\infty - M0} *N$$

IPTG

Time	Replicate 1	Replicate 2	Replicate 3	Replicate 4	Replicate5	average	SD
0	639.234	639.229				639.23	0.00
30	639.561	639.415				639.49	0.10
45	639.568	639.439				639.50	0.09
60	639.604	639.438				639.52	0.12
300	639.589	639.412				639.50	0.13
1500	639.566	639.46				639.51	0.07
3600	639.585	639.626				639.61	0.03
7200	639.61	639.514				639.56	0.07
14400	639.682	639.505				639.59	0.13

ONPF

Time	Replicate 1	Replicate 2	Replicate 3	Replicate 4	average	SD
0	639.253	639.262			639.26	0.01
30	639.448	639.393			639.42	0.04
45	639.583	639.431			639.51	0.11
60	639.542	639.494			639.52	0.03
300	639.56	639.444			639.50	0.08
1500	639.617	639.506			639.56	0.08
3600	639.695	639.509			639.60	0.13
7200	639.684	639.544			639.61	0.10
14400	639.792	639.442			639.62	0.25

APO

Time	Replicate 1	Replicate 2	Replicate 3	Replicate 4	Replicate 5	average	SD
0	639.225	639.234				639.23	0.01
30	639.475	639.388				639.43	0.06
45	639.498	639.403				639.45	0.00
60	639.492	639.487				639.49	0.00
300	639.494	639.433				639.46	0.04
1500	639.596	639.584				639.59	0.01
3600	639.65	639.593				639.62	0.04
7200	639.734	639.585				639.66	0.11
14400	640.069	639.648				639.86	0.30

DNA

Time	Replicate 1	Replicate 2	Replicate 3	Replicate 4	Replicate 5	average	SD
0	639.229	639.232				639.23	0.00
30	639.48	639.395				639.44	0.06
45	639.623	639.409				639.52	0.15
60	639.523	639.484				639.50	0.03
300	639.551	639.433				639.49	0.08
1500	639.563	639.504				639.53	0.04
3600	639.727	639.485				639.61	0.17
7200	639.685	639.521				639.60	0.12
14400	639.807	639.439				639.62	0.26

ONPFDNA

Time	Replicate 1	Replicate 2	Replicate 3	Replicate 4	average	SD
0	639.234	639.229			639.23	0.00
30	639.452	639.388			639.42	0.05
45	639.531	639.426			639.48	0.07
60	639.511	639.455			639.48	0.04
300	639.498	639.422			639.46	0.05
1500	639.476	639.461			639.47	0.01
3600	639.673	639.478			639.58	0.14
7200	639.686	639.509			639.60	0.13
14400	639.616	639.519			639.57	0.07

TMG

Time	Replicate 1	Replicate 2	Replicate 3	Replicate 4	Replicate 5	average	SD
0	639.244	639.231				639.24	0.01
30	639.416	639.553				639.48	0.10
45	639.534	639.548				639.54	0.01
60	639.493					639.49	
300	639.504	639.565				639.53	0.04
1500	639.509	639.717				639.61	0.15
3600	639.569	639.557				639.56	0.01
7200	639.542	639.61				639.58	0.05
14400	639.629	639.738				639.68	0.08

256-267 AITESGLRVGAD

Charge 2

IPTG

Time	centroid	D
0	595.162	0.01
30	595.9025	2.26
45	596.053	2.72
60	596.132	2.96
300	596.073	2.78
1500	596.1225	2.94
3600	596.257	3.35
7200	596.3055	3.49
14400	596.409	3.81

ONPF

Time	centroid	D
0	595.2155	0.17
30	595.5995	1.34
45	595.8665	2.15
60	595.949	2.41
300	595.9175	2.31
1500	596.006	2.58
3600	596.0065	2.58
7200	595.992	2.54
14400	596.07	2.76

APO

Time	centroid	D
0	595.1605	0.00
30	595.809	1.98
45	595.9	2.26
60	596.0435	2.69
300	595.94	2.38
1500	596.088	2.83
3600	596.1715	3.08
7200	596.164	3.06
14400	596.518	4.14

DNA

Time	centroid	D
0	595.1715	0.04
30	595.7555	1.82
45	595.934	2.36
60	595.9285	2.34
300	595.968	2.46
1500	596.0335	2.66
3600	596.0405	2.69
7200	595.998	2.56
14400	596.022	2.63

ONPFDNA

Time	centroid	D
0	595.16	0.01
30	595.71	1.68
45	595.92	2.32
60	595.89	2.24
300	595.95	2.41
1500	595.87	2.15
3600	596.08	2.81
7200	596.05	2.72
14400	595.91	2.29

TMG

Time	centroid	D
0	595.1755	0.05
30	595.865	2.15
45	596.017	2.61
60	596.14	2.99
300	596.141	2.99
1500	596.172	3.09
3600	596.2425	3.30
7200	596.269	3.38
14400	596.4835	4.04

control	595.16
infinity	598.439

$$D(t) = \frac{M_t - M_0}{M_\infty - M_0} * N$$

IPTG

Time	Replicate 1	Replicate 2	Replicate 3	Replicate 4	Replicate5	average	SD
0	595.168	595.156				595.16	0.01
30	596.089	595.716				595.90	0.26
45	596.144	595.962				596.05	0.13
60	596.248	596.016				596.13	0.16
300	596.217	595.929				596.07	0.20
1500	596.312	595.933				596.12	0.27
3600	596.355	596.159				596.26	0.14
7200	596.456	596.155				596.31	0.21
14400	596.585	596.233				596.41	0.25

ONPF

Time	Replicate 1	Replicate 2	Replicate 3	Replicate 4	average	SD
0	595.216	595.215			595.22	0.00
30	595.674	595.525			595.60	0.11
45	596.046	595.687			595.87	0.25
60	595.985	595.913			595.95	0.05
300	596.05	595.785			595.92	0.19
1500	596.102	595.91			596.01	0.14
3600	596.126	595.887			596.01	0.17
7200	596.118	595.866			595.99	0.18
14400	596.385	595.748			596.07	0.45

APO

Time	Replicate 1	Replicate 2	Replicate 3	Replicate 4	Replicate 5	average	SD
0	595.152	595.169				595.16	0.01
30	595.945	595.673				595.81	0.19
45	596.034	595.766				595.90	0.00
60	596.131	595.956				596.04	0.12
300	596.093	595.787				595.94	0.22
1500	596.132	596.044				596.09	0.06
3600	596.253	596.09				596.17	0.12
7200	596.369	595.959				596.16	0.29
14400	596.737	596.299				596.52	0.31

DNA

Time	Replicate 1	Replicate 2	Replicate 3	Replicate 4	Replicate 5	average	SD
0	595.153	595.19				595.17	0.03
30	595.796	595.715				595.76	0.06
45	596.063	595.805				595.93	0.18
60	595.976	595.881				595.93	0.07
300	596.016	595.92				595.97	0.07
1500	596.085	595.982				596.03	0.07
3600	596.164	595.917				596.04	0.17
7200	596.193	595.803				596.00	0.28
14400	596.295	595.749				596.02	0.39

ONPFDNA

Time	Replicate 1	Replicate 2	Replicate 3	Replicate 4	average	SD
0	595.161	595.163			595.16	0.00
30	595.73	595.691			595.71	0.03
45	595.984	595.855			595.92	0.09
60	595.898	595.888			595.89	0.01
300	596.003	595.899			595.95	0.07
1500	595.833	595.899			595.87	0.05
3600	596.116	596.05			596.08	0.05
7200	596.17	595.936			596.05	0.17
14400	595.908	595.917			595.91	0.01

TMG

Time	Replicate 1	Replicate 2	Replicate 3	Replicate 4	Replicate 5	average	SD
0	595.197	595.154				595.18	0.03
30	595.661	596.069				595.87	0.29
45	596.001	596.033				596.02	0.02
60	595.974	596.306				596.14	0.23
300	596.034	596.248				596.14	0.15
1500	595.978	596.366				596.17	0.27
3600	596.141	596.344				596.24	0.14
7200	596.069	596.469				596.27	0.28
14400	596.349	596.618				596.48	0.19

257-267 ITESGLRVGAD

Charge 2

IPTG

Time	centroid	D
0	559.62	-0.04
30	560.23	1.91
45	560.49	2.73
60	560.51	2.78
300	560.45	2.59
1500	560.51	2.78
3600	560.57	2.99
7200	560.68	3.33
14400	560.75	3.56

ONPF

Time	centroid	D
0	559.65	0.06
30	560.09	1.44
45	560.30	2.13
60	560.37	2.33
300	560.32	2.18
1500	560.40	2.43
3600	560.45	2.59
7200	560.45	2.59
14400	560.59	3.05

APO

Time	centroid	D
0	559.6262	-0.01
30	560.2032	1.81
45	560.349	2.28
60	560.4542	2.61
300	560.371	2.34
1500	560.4782	2.68
3600	560.5496	2.91
7200	560.6272	3.16
14400	560.7978	3.70

DNA

Time	centroid	D
0	559.62	-0.03
30	560.14	1.63
45	560.35	2.28
60	560.39	2.41
300	560.39	2.41
1500	560.44	2.57
3600	560.49	2.71
7200	560.41	2.48
14400	560.49	2.72

ONPFDNA

Time	centroid	D
0	559.60	-0.09
30	560.15	1.63
45	560.36	2.32
60	560.38	2.36
300	560.40	2.43
1500	560.29	2.08
3600	560.50	2.76
7200	560.42	2.49
14400	560.39	2.42

TMG

Time	centroid	D
0	559.64	0.04
30	560.33	2.23
45	560.47	2.66
60	560.56	2.95
300	560.57	2.97
1500	560.60	3.08
3600	560.67	3.28
7200	560.74	3.51
14400	560.88	3.97

control 559.63
infinity 562.474

$$D(t) = \frac{M_t - M_0}{M_\infty - M_0} * N$$

IPTG

Time	Replicate 1	Replicate 2	Replicate 3	Replicate 4	Replicate5	average	SD
0	559.622	559.61	559.623	559.613	559.614	559.62	0.01
30	560.531	560.138	560.262	560.136	560.094	560.23	0.18
45	560.589	560.394				560.49	0.14
60	560.637	560.448	560.693	560.4	560.362	560.51	0.15
300	560.666	560.367	560.563	560.373	560.273	560.45	0.16
1500	560.727	560.375	560.646	560.429	560.359	560.51	0.17
3600	560.769	560.602	560.629	560.461	560.41	560.57	0.14
7200	560.915	560.627	560.769	560.587	560.52	560.68	0.16
14400	561.021	560.662	560.753	560.753	560.578	560.75	0.17

ONPF

Time	Replicate 1	Replicate 2	Replicate 3	Replicate 4	average	SD
0	559.683	559.671	559.619	559.619	559.65	0.03
30	560.112	559.981	560.23	560.017	560.09	0.11
45	560.474	560.132			560.30	0.24
60	560.407	560.332	560.386	560.342	560.37	0.04
300	560.496	560.205	560.379	560.2	560.32	0.14
1500	560.526	560.317	560.391	560.363	560.40	0.09
3600	560.537	560.313	560.596	560.349	560.45	0.14
7200	560.535	560.299	560.529	560.435	560.45	0.11
14400	560.811	560.167	560.716	560.684	560.59	0.29

APO

Time	Replicate 1	Replicate 2	Replicate 3	Replicate 4	Replicate 5	average	SD
0	559.605	559.624	559.677	559.617	559.608	559.63	0.03
30	560.364	560.117	560.453	560.03	560.052	560.20	0.19
45	560.495	560.203				560.35	0.21
60	560.551	560.382	560.727	560.306	560.305	560.45	0.18
300	560.532	560.204	560.626	560.289	560.204	560.37	0.20
1500	560.568	560.466	560.696	560.33	560.331	560.48	0.16
3600	560.667	560.484	560.816	560.403	560.378	560.55	0.19
7200	560.799	560.381	560.916	560.589	560.451	560.63	0.23
14400	561.002	560.737	560.891	560.685	560.674	560.80	0.14

DNA

Time	Replicate 1	Replicate 2	Replicate 3	Replicate 4	Replicate 5	average	SD
0	559.613	559.638	559.618	559.611		559.62	0.01
30	560.282	560.15	560.212	559.934		560.14	0.15
45	560.463	560.239				560.35	0.16
60	560.415	560.311	560.624	560.218		560.39	0.17
300	560.5	560.353	560.597	560.122		560.39	0.21
1500	560.526	560.427	560.632	560.189		560.44	0.19
3600	560.68	560.343	560.67	560.252		560.49	0.22
7200	560.601	560.189	560.625	560.244		560.41	0.23
14400	560.742	560.154	560.708	560.357		560.49	0.28

ONPFDNA

Time	Replicate 1	Replicate 2	Replicate 3	Replicate 4	average	SD
0	559.619	559.618	559.556	559.614	559.60	0.03
30	560.178	560.103	560.371	559.929	560.15	0.18
45	560.429	560.296			560.36	0.09
60	560.33	560.319	560.58	560.279	560.38	0.14
300	560.446	560.327	560.521	560.292	560.40	0.11
1500	560.265	560.321	560.273	560.293	560.29	0.02
3600	560.562	560.486	560.627	560.335	560.50	0.13
7200	560.527	560.315		560.409	560.42	0.11
14400	560.294	560.34	560.479	560.466	560.39	0.09

TMG

Time	Replicate 1	Replicate 2	Replicate 3	Replicate 4	Replicate 5	average	SD
0	559.661	559.624				559.64	0.03
30	560.095	560.573				560.33	0.34
45	560.425	560.516				560.47	0.06
60	560.437	560.69				560.56	0.18
300	560.462	560.672				560.57	0.15
1500	560.42	560.785				560.60	0.26
3600	560.55	560.784				560.67	0.17
7200	560.535	560.943				560.74	0.29
14400	560.757	561.011				560.88	0.18

266-273 ADISVVGY

Charge 1

IPTG

Time	centroid	D
0	823.917	0.02
30	824.122	0.27
45	824.093	0.23
60	824.1735	0.33
300	824.152	0.31
1500	824.0955	0.24
3600	824.1005	0.24
7200	824.1275	0.28
14400	824.1135	0.26

ONPF

Time	centroid	D
0	823.92	0.03
30	824.08	0.22
45	824.13	0.28
60	824.14	0.29
300	824.12	0.27
1500	824.21	0.37
3600	824.13	0.28
7200	824.08	0.22
14400	824.08	0.22

APO

Time	centroid	D
0	823.904	0.00
30	824.062	0.20
45	824.0585	0.19
60	824.0815	0.22
300	824.0875	0.23
1500	824.0905	0.23
3600	824.0915	0.23
7200	824.0835	0.22
14400	824.314	0.50

DNA

Time	centroid	D
0	823.909	0.01
30	824.0855	0.23
45	824.127	0.28
60	824.1505	0.30
300	824.112	0.26
1500	824.0855	0.23
3600	824.094	0.24
7200	824.07	0.21
14400	824.093	0.23

ONPFDNA

Time	centroid	D
0	823.897	0.00
30	824.091	0.23
45	824.123	0.27
60	824.115	0.26
300	824.075	0.21
1500	824.1015	0.24
3600	824.076	0.21
7200	824.078	0.22
14400	824.099	0.24

TMG

Time	centroid	D
0	823.91	0.02
30	824.11	0.26
45	824.15	0.30
60	824.18	0.34
300	824.17	0.32
1500	824.24	0.41
3600	824.16	0.32
7200	824.14	0.29
14400	824.20	0.37

control 823.9
infinity 828.844

$$D(t) = \frac{M_t - M_0}{M_\infty - M_0} * N$$

IPTG

Time	Replicate 1	Replicate 2	Replicate 3	Replicate 4	Replicate5	average	SD
0	823.938	823.896				823.92	0.03
30	824.188	824.056				824.12	0.09
45	824.129	824.057				824.09	0.05
60	824.281	824.066				824.17	0.15
300	824.232	824.072				824.15	0.11
1500	824.143	824.048				824.10	0.07
3600	824.151	824.05				824.10	0.07
7200	824.162	824.093				824.13	0.05
14400	824.163	824.064				824.11	0.07

ONPF

Time	Replicate 1	Replicate 2	Replicate 3	Replicate 4	average	SD
0	823.928	823.914			823.92	0.01
30	824.112	824.047			824.08	0.05
45	824.146	824.11			824.13	0.03
60	824.152	824.119			824.14	0.02
300	824.133	824.106			824.12	0.02
1500	824.303	824.112			824.21	0.14
3600	824.161	824.096			824.13	0.05
7200	824.1	824.061			824.08	0.03
14400	824.174	823.983			824.08	0.14

APO

Time	Replicate 1	Replicate 2	Replicate 3	Replicate 4	Replicate 5	average	SD
0	823.908	823.9				823.90	0.01
30	824.079	824.045				824.06	0.02
45	824.048	824.069				824.06	0.01
60	824.083	824.08				824.08	0.00
300	824.08	824.095				824.09	0.01
1500	824.08	824.101				824.09	0.01
3600	824.089	824.094				824.09	0.00
7200	824.1	824.067				824.08	0.02
14400	824.46	824.168				824.31	0.21

DNA

Time	Replicate 1	Replicate 2	Replicate 3	Replicate 4	Replicate 5	average	SD
0	823.926	823.892				823.91	0.02
30	824.152	824.019				824.09	0.09
45	824.204	824.05				824.13	0.11
60	824.244	824.057				824.15	0.13
300	824.176	824.048				824.11	0.09
1500	824.131	824.04				824.09	0.06
3600	824.15	824.038				824.09	0.08
7200	824.096	824.044				824.07	0.04
14400	824.142	824.044				824.09	0.07

ONPFDNA

Time	Replicate 1	Replicate 2	Replicate 3	Replicate 4	average	SD
0	823.903	823.891			823.90	0.01
30	824.136	824.046			824.09	0.06
45	824.183	824.063			824.12	0.08
60	824.131	824.099			824.12	0.02
300	824.101	824.049			824.08	0.04
1500	824.134	824.069			824.10	0.05
3600	824.115	824.037			824.08	0.06
7200	824.121	824.035			824.08	0.06
14400	824.11	824.088			824.10	0.02

TMG

Time	Replicate 1	Replicate 2	Replicate 3	Replicate 4	Replicate 5	average	SD
0	823.906	823.921				823.91	0.01
30	824.082	824.146				824.11	0.05
45	824.166	824.136				824.15	0.02
60	824.181					824.18	
300	824.162	824.17				824.17	0.01
1500	824.137	824.333				824.24	0.14
3600	824.177	824.144				824.16	0.02
7200	824.131	824.15				824.14	0.01
14400	824.159	824.249				824.20	0.06

270-275 VVGYDD

Charge 1

IPTG

Time	centroid	D
0	667.66	0.01
30	667.89	0.52
45	667.87	0.47
60	667.91	0.57
300	667.88	0.51
1500	667.86	0.47
3600	667.94	0.63
7200	667.87	0.48
14400	667.87	0.48

ONPF

Time	centroid	D
0	667.65	0.00
30	667.82	0.38
45	667.87	0.48
60	667.86	0.45
300	667.84	0.42
1500	667.87	0.48
3600	667.87	0.49
7200	667.90	0.55
14400	667.84	0.42

APO

Time	centroid	D
0	667.65	0.01
30	667.83	0.38
45	667.82	0.36
60	667.83	0.39
300	667.80	0.33
1500	667.86	0.47
3600	667.90	0.55
7200	667.93	0.62
14400	668.10	0.99

DNA

Time	centroid	D
0	667.64	-0.01
30	667.83	0.40
45	667.82	0.38
60	667.82	0.36
300	667.83	0.39
1500	667.84	0.42
3600	667.84	0.42
7200	667.85	0.43
14400	667.83	0.40

ONPFDNA

Time	centroid	D
0	667.64	-0.01
30	667.83	0.40
45	667.86	0.45
60	667.83	0.39
300	667.80	0.33
1500	667.78	0.28
3600	667.83	0.39
7200	667.82	0.38
14400	667.80	0.32

TMG

Time	centroid	D
0	667.65	0.00
30	667.89	0.52
45	667.92	0.59
60	667.91	0.57
300	667.94	0.63
1500	667.97	0.69
3600	667.91	0.58
7200	667.90	0.56
14400	667.98	0.73

control	667.65
infinity	669.477

$$D(t) = \frac{M_t - M_0}{M_\infty - M_0} \cdot N$$

IPTG

Time	Replicate 1	Replicate 2	Replicate 3	Replicate 4	Replicate5	average	SD
0	667.66	667.65				667.66	0.01
30	667.959	667.815				667.89	0.10
45	667.94	667.793				667.87	0.10
60	668.011	667.813				667.91	0.14
300	667.971	667.793				667.88	0.13
1500	667.925	667.804				667.86	0.09
3600	668.065	667.812				667.94	0.18
7200	667.92	667.823				667.87	0.07
14400	667.925	667.809				667.87	0.08

ONPF

Time	Replicate 1	Replicate 2	Replicate 3	Replicate 4	average	SD
0	667.647	667.657			667.65	0.01
30	667.858	667.789			667.82	0.05
45	667.923	667.812			667.87	0.08
60	667.9	667.813			667.86	0.06
300	667.898	667.782			667.84	0.08
1500	667.948	667.794			667.87	0.11
3600	668.002	667.743			667.87	0.18
7200	667.987	667.814			667.90	0.12
14400	668.068	667.612			667.84	0.32

APO

Time	Replicate 1	Replicate 2	Replicate 3	Replicate 4	Replicate 5	average	SD
0	667.664	667.641				667.65	0.02
30	667.855	667.796				667.83	0.04
45	667.851	667.781				667.82	0.05
60	667.843	667.809				667.83	0.02
300	667.831	667.775				667.80	0.04
1500	667.895	667.83				667.86	0.05
3600	667.947	667.858				667.90	0.06
7200	668.002	667.864				667.93	0.10
14400	668.226	667.979				668.10	0.17

DNA

Time	Replicate 1	Replicate 2	Replicate 3	Replicate 4	Replicate 5	average	SD
0	667.654	667.633				667.64	0.01
30	667.893	667.77				667.83	0.09
45	667.86	667.783				667.82	0.05
60	667.871	667.759				667.82	0.08
300	667.893	667.763				667.83	0.09
1500	667.878	667.804				667.84	0.05
3600	667.921	667.763				667.84	0.11
7200	667.909	667.784				667.85	0.09
14400	667.936	667.727				667.83	0.15

ONPFDNA

Time	Replicate 1	Replicate 2	Replicate 3	Replicate 4	average	SD
0	667.641	667.648			667.64	0.00
30	667.875	667.786			667.83	0.06
45	667.905	667.806			667.86	0.07
60	667.858	667.798			667.83	0.04
300	667.828	667.774			667.80	0.04
1500	667.787	667.765			667.78	0.02
3600	667.871	667.788			667.83	0.06
7200	667.89	667.758			667.82	0.09
14400	667.79	667.804			667.80	0.01

TMG

Time	Replicate 1	Replicate 2	Replicate 3	Replicate 4	Replicate 5	average	SD
0	667.645	667.654				667.65	0.01
30	667.863	667.916				667.89	0.04
45	667.907	667.933				667.92	0.02
60	667.91					667.91	
300	667.909	667.967				667.94	0.04
1500	667.9	668.032				667.97	0.09
3600	667.917	667.91				667.91	0.00
7200	667.889	667.919				667.90	0.02
14400	667.931	668.033				667.98	0.07

274-290 DDTEDSSCYIPPLTTIK

Charge 2

IPTG

Time	centroid	D
0	950.0085	-0.03
30	950.424	1.02
45	950.4475	1.08
60	950.567	1.39
300	950.804	1.99
1500	951.166	2.90
3600	951.407	3.51
7200	951.574	3.94
14400	951.6605	4.16

ONPF

Time	centroid	D
0	950.0145	-0.01
30	950.335	0.80
45	950.5905	1.45
60	950.598	1.46
300	950.61	1.49
1500	950.9635	2.39
3600	951.247	3.11
7200	951.6	4.00
14400	952.08	5.22

APO

Time	centroid	D
0	950.02	0.00
30	950.381	0.91
45	950.4145	1.00
60	950.5705	1.39
300	950.613	1.50
1500	951.1745	2.92
3600	951.4885	3.72
7200	951.784	4.47
14400	952.248	5.64

DNA

Time	centroid	D
0	950.001	-0.05
30	950.3295	0.78
45	950.3615	0.87
60	950.4085	0.98
300	950.4155	1.00
1500	950.5445	1.33
3600	950.519	1.26
7200	950.4885	1.19
14400	950.4555	1.10

ONPFDNA

Time	centroid	D
0	949.9975	-0.06
30	950.3205	0.76
45	950.398	0.96
60	950.3985	0.96
300	950.381	0.91
1500	950.417	1.01
3600	950.5495	1.34
7200	950.5345	1.30
14400	950.416	1.00

TMG

Time	centroid	D
0	950.007	-0.03
30	950.444	1.07
45	950.554	1.35
60	950.636	1.56
300	950.7855	1.94
1500	951.149	2.86
3600	951.4065	3.51
7200	951.494	3.73
14400	951.763	4.42

control	950.02
infinity	955.152

$$D(t) = \frac{M_t - M_0}{M_\infty - M_0} * N$$

IPTG

Time	Replicate 1	Replicate 2	Replicate 3	Replicate 4	Replicate5	average	SD
0	950.003	950.014				950.01	0.01
30	950.319	950.529				950.42	0.15
45	950.362	950.533				950.45	0.12
60	950.466	950.668				950.57	0.14
300	950.649	950.959				950.80	0.22
1500	951.014	951.318				951.17	0.21
3600	951.358	951.456				951.41	0.07
7200	951.438	951.71				951.57	0.19
14400	951.49	951.831				951.66	0.24

ONPF

Time	Replicate 1	Replicate 2	Replicate 3	Replicate 4	average	SD
0	950.024	950.005			950.01	0.01
30	950.286	950.384			950.34	0.07
45	950.409	950.772			950.59	0.26
60	950.649	950.547			950.60	0.07
300	950.499	950.721			950.61	0.16
1500	950.831	951.096			950.96	0.19
3600	951.04	951.454			951.25	0.29
7200	951.438	951.762			951.60	0.23
14400		952.082			952.08	

APO

Time	Replicate 1	Replicate 2	Replicate 3	Replicate 4	Replicate 5	average	SD
0	950.016	950.024				950.02	0.01
30	950.319	950.443				950.38	0.09
45	950.334	950.495				950.41	0.11
60	950.606	950.535				950.57	0.05
300	950.524	950.702				950.61	0.13
1500	951.272	951.077				951.17	0.14
3600	951.399	951.578				951.49	0.13
7200	951.621	951.947				951.78	0.23
14400	952.081	952.415				952.25	0.24

DNA

Time	Replicate 1	Replicate 2	Replicate 3	Replicate 4	Replicate 5	average	SD
0	949.998	950.004				950.00	0.00
30	950.3	950.359				950.33	0.04
45	950.312	950.411				950.36	0.07
60	950.392	950.425				950.41	0.02
300	950.367	950.464				950.42	0.07
1500	950.529	950.56				950.54	0.02
3600	950.455	950.583				950.52	0.09
7200	950.398	950.579				950.49	0.13
14400	950.264	950.647				950.46	0.27

ONPFDNA

Time	Replicate 1	Replicate 2	Replicate 3	Replicate 4	average	SD
0	949.994	950.001			950.00	0.00
30	950.299	950.342			950.32	0.03
45	950.315	950.481			950.40	0.12
60	950.37	950.427			950.40	0.04
300	950.307	950.455			950.38	0.10
1500	950.428	950.406			950.42	0.02
3600	950.499	950.6			950.55	0.07
7200	950.489	950.58			950.53	0.06
14400	950.485	950.347			950.42	0.10

TMG

Time	Replicate 1	Replicate 2	Replicate 3	Replicate 4	Replicate 5	average	SD
0	950.004	950.01				950.01	0.00
30	950.354	950.534				950.44	0.13
45	950.61	950.498				950.55	0.08
60	950.636					950.64	
300	950.745	950.826				950.79	0.06
1500	951.029	951.269				951.15	0.17
3600	951.367	951.446				951.41	0.06
7200	951.306	951.682				951.49	0.27
14400	951.549	951.977				951.76	0.30

276-290 TEDSSCYIPPLTTIK

Charge 2

IPTG

Time	centroid	D
0	834.895	-0.07
30	835.118	1.00
45	835.1525	1.16
60	835.243	1.60
300	835.3665	2.19
1500	835.758	4.07
3600	835.968	5.07
7200	836.202	6.20
14400	836.175	6.07

ONPF

Time	centroid	D
0	834.9085	-0.01
30	835.056	0.70
45	835.23	1.53
60	835.2755	1.75
300	835.259	1.67
1500	835.538	3.01
3600	835.81	4.32
7200	835.9305	4.89
14400	836.35	6.92

APO

Time	centroid	D
0	834.911	0.00
30	835.0665	0.75
45	835.1105	0.96
60	835.2145	1.46
300	835.2545	1.65
1500	835.7565	4.06
3600	836.0035	5.24
7200	836.2905	6.62
14400	836.5885	8.05

DNA

Time	centroid	D
0	834.8885	-0.10
30	835.039	0.62
45	835.067	0.75
60	835.096	0.89
300	835.116	0.99
1500	835.2025	1.40
3600	835.1715	1.25
7200	835.1755	1.27
14400	835.167	1.23

ONPFDNA

Time	centroid	D
0	834.9055	-0.02
30	835.0335	0.59
45	835.0745	0.79
60	835.092	0.87
300	835.0595	0.72
1500	835.1375	1.09
3600	835.212	1.45
7200	835.1955	1.37
14400	835.1395	1.10

TMG

Time	centroid	D
0	834.906	-0.02
30	835.155	1.17
45	835.355	2.13
60	835.277	1.76
300	835.39	2.30
1500	835.685	3.72
3600	835.9305	4.89
7200	836.083	5.62
14400	836.317	6.75

control 834.91
infinity 837.204

$$D(t) = \frac{Mt - M0}{M\infty - M0} *N$$

IPTG

Time	Replicate 1	Replicate 2	Replicate 3	Replicate 4	Replicate5	average	SD
0	834.906	834.884				834.90	0.02
30	835.174	835.062				835.12	0.08
45	835.212	835.093				835.15	0.08
60	835.3	835.186				835.24	0.08
300	835.474	835.259				835.37	0.15
1500	835.837	835.679				835.76	0.11
3600	835.998	835.938				835.97	0.04
7200	836.299	836.105				836.20	0.14
14400	836.282	836.068				836.18	0.15

ONPF

Time	Replicate 1	Replicate 2	Replicate 3	Replicate 4	average	SD
0	834.907	834.91			834.91	0.00
30	835.061	835.051			835.06	0.01
45	835.381	835.079			835.23	0.21
60	835.225	835.326			835.28	0.07
300	835.298	835.22			835.26	0.06
1500	835.604	835.472			835.54	0.09
3600	835.906	835.714			835.81	0.14
7200	835.899	835.962			835.93	0.04
14400	836.354				836.35	

APO

Time	Replicate 1	Replicate 2	Replicate 3	Replicate 4	Replicate 5	average	SD
0	834.903	834.919				834.91	0.01
30	835.08	835.053				835.07	0.02
45	835.123	835.098				835.11	0.02
60	835.129	835.3				835.21	0.12
300	835.274	835.235				835.25	0.03
1500	835.632	835.881				835.76	0.18
3600	836.034	835.973				836.00	0.04
7200	836.407	836.174				836.29	0.16
14400	836.679	836.498				836.59	0.13

DNA

Time	Replicate 1	Replicate 2	Replicate 3	Replicate 4	Replicate 5	average	SD
0	834.892	834.885				834.89	0.00
30	835.07	835.008				835.04	0.04
45	835.102	835.032				835.07	0.05
60	835.112	835.08				835.10	0.02
300	835.157	835.075				835.12	0.06
1500	835.204	835.201				835.20	0.00
3600	835.229	835.114				835.17	0.08
7200	835.227	835.124				835.18	0.07
14400	835.293	835.041				835.17	0.18

ONPFDNA

Time	Replicate 1	Replicate 2	Replicate 3	Replicate 4	average	SD
0	834.902	834.909			834.91	0.00
30	835.054	835.013			835.03	0.03
45	835.1	835.049			835.07	0.04
60	835.107	835.077			835.09	0.02
300	835.116	835.003			835.06	0.08
1500	835.13	835.145			835.14	0.01
3600	835.267	835.157			835.21	0.08
7200	835.218	835.173			835.20	0.03
14400	835.103	835.176			835.14	0.05

TMG

Time	Replicate 1	Replicate 2	Replicate 3	Replicate 4	Replicate 5	average	SD
0	834.918	834.894				834.91	0.02
30	835.101	835.209				835.16	0.08
45	835.437	835.273				835.36	0.12
60	835.277					835.28	
300	835.383	835.397				835.39	0.01
1500	835.613	835.757				835.69	0.10
3600	835.909	835.952				835.93	0.03
7200	835.963	836.203				836.08	0.17
14400	836.186	836.448				836.32	0.19

279-292 SSCYIPPLTTIKQD

Charge 2

IPTG

Time	centroid	D
0	783.9025	0.01
30	784.2165	0.84
45	784.2365	0.90
60	784.3165	1.11
300	784.3625	1.23
1500	784.568	1.77
3600	784.726	2.19
7200	784.8975	2.65
14400	784.9435	2.77

ONPF

Time	centroid	D
0	783.9145	0.04
30	784.1165	0.58
45	784.317	1.11
60	784.3105	1.09
300	784.2805	1.01
1500	784.473	1.52
3600	784.6905	2.10
7200	784.8995	2.65
14400	784.74	2.24

APO

Time	centroid	D
0	783.8985	0.00
30	784.169	0.72
45	784.195	0.79
60	784.2895	1.04
300	784.262	0.96
1500	784.658	2.01
3600	784.8805	2.60
7200	785.204	3.46
14400	785.599	4.51

DNA

Time	centroid	D
0	783.9085	0.03
30	784.1215	0.59
45	784.188	0.77
60	784.1835	0.76
300	784.1945	0.78
1500	784.2055	0.81
3600	784.257	0.95
7200	784.1985	0.80
14400	784.1935	0.78

ONPFDNA

Time	centroid	D
0	783.9045	0.02
30	784.1065	0.55
45	784.1455	0.65
60	784.1535	0.68
300	784.1705	0.72
1500	784.1485	0.66
3600	784.2355	0.89
7200	784.2195	0.85
14400	784.167	0.71

TMG

Time	centroid	D
0	783.8995	0.00
30	784.2005	0.80
45	784.3605	1.22
60	784.716	2.17
300	784.3595	1.22
1500	784.5615	1.76
3600	784.6805	2.07
7200	784.8465	2.51
14400	785.0945	3.17

control 783.8985
infinity 787.671

$$D(t) = \frac{Mt - M0}{M\infty - M0} *N$$

IPTG

Time	Replicate 1	Replicate 2	Replicate 3	Replicate 4	Replicate5	average	SD
0	783.91	783.895				783.90	0.01
30	784.269	784.164				784.22	0.07
45	784.281	784.192				784.24	0.06
60	784.398	784.235				784.32	0.12
300	784.472	784.253				784.36	0.15
1500	784.627	784.509				784.57	0.08
3600	784.762	784.69				784.73	0.05
7200	784.96	784.835				784.90	0.09
14400	785.043	784.844				784.94	0.14

ONPF

Time	Replicate 1	Replicate 2	Replicate 3	Replicate 4	average	SD
0	783.919	783.91			783.91	0.01
30	784.16	784.073			784.12	0.06
45	784.466	784.168			784.32	0.21
60	784.266	784.355			784.31	0.06
300	784.332	784.229			784.28	0.07
1500	784.545	784.401			784.47	0.10
3600	784.844	784.537			784.69	0.22
7200	785.001	784.798			784.90	0.14
14400	785.381	784.106			784.74	0.90

APO

Time	Replicate 1	Replicate 2	Replicate 3	Replicate 4	Replicate 5	average	SD
0	783.897	783.9				783.90	0.00
30	784.21	784.128				784.17	0.06
45	784.246	784.144				784.20	0.07
60	784.25	784.329				784.29	0.06
300	784.298	784.226				784.26	0.05
1500	784.547	784.769				784.66	0.16
3600	784.906	784.855				784.88	0.04
7200	785.282	785.126				785.20	0.11
14400	785.699	785.499				785.60	0.14

DNA

Time	Replicate 1	Replicate 2	Replicate 3	Replicate 4	Replicate 5	average	SD
0	783.918	783.899				783.91	0.01
30	784.152	784.091				784.12	0.04
45	784.252	784.124				784.19	0.09
60	784.204	784.163				784.18	0.03
300	784.231	784.158				784.19	0.05
1500	784.217	784.194				784.21	0.02
3600	784.345	784.169				784.26	0.12
7200	784.259	784.138				784.20	0.09
14400	784.285	784.102				784.19	0.13

ONPFDNA

Time	Replicate 1	Replicate 2	Replicate 3	Replicate 4	average	SD
0	783.908	783.901			783.90	0.00
30	784.15	784.063			784.11	0.06
45	784.191	784.1			784.15	0.06
60	784.176	784.131			784.15	0.03
300	784.199	784.142			784.17	0.04
1500	784.132	784.165			784.15	0.02
3600	784.245	784.226			784.24	0.01
7200	784.259	784.18			784.22	0.06
14400	784.158	784.176			784.17	0.01

TMG

Time	Replicate 1	Replicate 2	Replicate 3	Replicate 4	Replicate 5	average	SD
0	783.902	783.897				783.90	0.00
30	784.142	784.259				784.20	0.08
45	784.451	784.27				784.36	0.13
60	784.29	785.142				784.72	0.60
300	784.337	784.382				784.36	0.03
1500	784.448	784.675				784.56	0.16
3600	784.659	784.702				784.68	0.03
7200	784.784	784.909				784.85	0.09
14400	784.992	785.197				785.09	0.14

293-296 FRLL

Charge 1

IPTG

Time	centroid	D
0	548.6745	0.01
30	548.722	0.07
45	548.7295	0.08
60	548.767	0.12
300	548.774	0.13
1500	548.9555	0.36
3600	549.154	0.62
7200	549.2785	0.77
14400	549.2585	0.75

ONPF

Time	centroid	D
0	548.676	0.01
30	548.712	0.05
45	548.793	0.16
60	548.7725	0.13
300	548.753	0.11
1500	548.8265	0.20
3600	548.921	0.32
7200	548.9855	0.40
14400	548.94	0.34

APO

Time	centroid	D
0	548.6695	0.00
30	548.7085	0.05
45	548.735	0.08
60	548.7745	0.13
300	548.7455	0.10
1500	548.928	0.33
3600	549.03	0.46
7200	549.112	0.56
14400	549.3015	0.80

DNA

Time	centroid	D
0	548.6675	0.00
30	548.704	0.04
45	548.764	0.12
60	548.739	0.09
300	548.7295	0.08
1500	548.7685	0.13
3600	548.8355	0.21
7200	548.871	0.26
14400	548.8855	0.27

ONPFDNA

Time	centroid	D
0	548.668	0.00
30	548.7055	0.05
45	548.722	0.07
60	548.731	0.08
300	548.721	0.07
1500	548.7665	0.12
3600	548.8195	0.19
7200	548.8335	0.21
14400	548.8385	0.21

TMG

Time	centroid	D
0	548.6645	-0.01
30	548.7195	0.06
45	548.799	0.16
60	548.851	0.23
300	548.788	0.15
1500	548.864	0.25
3600	549.0725	0.51
7200	549.139	0.60
14400	549.3005	0.80

control 548.6695
infinity 550.244

$$D(t) = \frac{M_t - M_0}{M_\infty - M_0} *N$$

IPTG

Time	Replicate 1	Replicate 2	Replicate 3	Replicate 4	Replicate5	average	SD
0	548.677	548.672				548.67	0.00
30	548.74	548.704				548.72	0.03
45	548.745	548.714				548.73	0.02
60	548.772	548.762				548.77	0.01
300	548.786	548.762				548.77	0.02
1500	548.994	548.917				548.96	0.05
3600	549.193	549.115				549.15	0.06
7200	549.348	549.209				549.28	0.10
14400	549.325	549.192				549.26	0.09

ONPF

Time	Replicate 1	Replicate 2	Replicate 3	Replicate 4	average	SD
0	548.671	548.681			548.68	0.01
30	548.721	548.703			548.71	0.01
45	548.854	548.732			548.79	0.09
60	548.759	548.786			548.77	0.02
300	548.765	548.741			548.75	0.02
1500	548.843	548.81			548.83	0.02
3600	549.01	548.832			548.92	0.13
7200	549.03	548.941			548.99	0.06
14400	549.239	548.643			548.94	0.42

APO

Time	Replicate 1	Replicate 2	Replicate 3	Replicate 4	Replicate 5	average	SD
0	548.666	548.673				548.67	0.00
30	548.709	548.708				548.71	0.00
45	548.751	548.719				548.74	0.02
60	548.741	548.808				548.77	0.05
300	548.743	548.748				548.75	0.00
1500	548.866	548.99				548.93	0.09
3600	549.045	549.015				549.03	0.02
7200	549.155	549.069				549.11	0.06
14400	549.39	549.213				549.30	0.13

DNA

Time	Replicate 1	Replicate 2	Replicate 3	Replicate 4	Replicate 5	average	SD
0	548.666	548.669				548.67	0.00
30	548.714	548.694				548.70	0.01
45	548.821	548.707				548.76	0.08
60	548.729	548.749				548.74	0.01
300	548.743	548.716				548.73	0.02
1500	548.777	548.76				548.77	0.01
3600	548.889	548.782				548.84	0.08
7200	548.91	548.832				548.87	0.06
14400	549.027	548.744				548.89	0.20

ONPFDNA

Time	Replicate 1	Replicate 2	Replicate 3	Replicate 4	average	SD
0	548.663	548.673			548.67	0.01
30	548.713	548.698			548.71	0.01
45	548.734	548.71			548.72	0.02
60	548.729	548.733			548.73	0.00
300	548.72	548.722			548.72	0.00
1500	548.739	548.794			548.77	0.04
3600	548.862	548.777			548.82	0.06
7200	548.861	548.806			548.83	0.04
14400	548.834	548.843			548.84	0.01

TMG

Time	Replicate 1	Replicate 2	Replicate 3	Replicate 4	Replicate 5	average	SD
0	548.661	548.668				548.66	0.00
30	548.709	548.73				548.72	0.01
45	548.851	548.747				548.80	0.07
60	548.758	548.944				548.85	0.13
300	548.801	548.775				548.79	0.02
1500	548.838	548.89				548.86	0.04
3600	549.095	549.05				549.07	0.03
7200	549.062	549.216				549.14	0.11
14400	549.251	549.35				549.30	0.07

293-301 FRLLGQTSV

Charge 1

IPTG

Time	centroid	D
0	511.09	-0.03
30	511.23	0.33
45	511.22	0.31
60	511.27	0.43
300	511.28	0.47
1500	511.35	0.63
3600	511.43	0.83
7200	511.51	1.03
14400	511.52	1.07

ONPF

Time	centroid	D
0	511.09	-0.03
30	511.18	0.21
45		
60	511.25	0.39
300	511.24	0.35
1500	511.28	0.45
3600	511.37	0.68
7200	511.42	0.81
14400	511.60	1.28

APO

Time	centroid	D
0	511.10	0.00
30	511.19	0.24
45	511.27	0.42
60	511.29	0.48
300	511.27	0.43
1500	511.33	0.58
3600	511.42	0.82
7200	511.51	1.05
14400	511.69	1.50

DNA

Time	centroid	D
0	511.07	-0.09
30	511.16	0.16
45		
60	511.19	0.24
300	511.19	0.24
1500	511.21	0.28
3600	511.26	0.40
7200	511.32	0.56
14400	511.38	0.72

ONPFDNA

Time	centroid	D
0	511.15	0.12
30	511.18	0.20
45	511.23	0.33
60	511.19	0.22
300	511.18	0.21
1500	511.21	0.29
3600	511.26	0.40
7200	511.25	0.38
14400	511.26	0.42

TMG

Time	centroid	D
0	511.07	-0.07
30	511.21	0.27
45	511.25	0.37
60	511.29	0.47
300	511.27	0.42
1500	511.23	0.33
3600	511.39	0.74
7200	511.40	0.77
14400	511.56	1.17

control 511.1
infinity 513.855

$$D(t) = \frac{M_t - M_0}{M_\infty - M_0} * N$$

IPTG

Time	Replicate 1	Replicate 2	Replicate 3	Replicate 4	Replicate5	average	SD
0	511.114	511.069	511.081			511.09	0.02
30	511.296	511.201	511.189			511.23	0.06
45	511.222					511.22	
60	511.27	511.27	511.266			511.27	0.00
300	511.355	511.246	511.252			511.28	0.06
1500	511.353	511.359	511.334			511.35	0.01
3600	511.518	511.382	511.38			511.43	0.08
7200	511.56	511.493	511.465			511.51	0.05
14400	511.594	511.527	511.448			511.52	0.07

ONPF

Time	Replicate 1	Replicate 2	Replicate 3	Replicate 4	average	SD
0	511.102	511.082	511.075		511.09	0.01
30	511.162	511.175	511.208		511.18	0.02
45						
60	511.174	511.237	511.346		511.25	0.09
300	511.255	511.205	511.248		511.24	0.03
1500	511.242	511.277	511.318		511.28	0.04
3600	511.371	511.307	511.425		511.37	0.06
7200	511.355	511.429	511.468		511.42	0.06
14400	511.626	511.545	511.636		511.60	0.05

APO

Time	Replicate 1	Replicate 2	Replicate 3	Replicate 4	Replicate 5	average	SD
0	511.029	511.228	511.094	511.066	511.085	511.10	0.08
30	511.165		511.282	511.174	511.158	511.19	0.06
45	511.27	511.261				511.27	0.01
60	511.186	511.326	511.423	511.247	511.267	511.29	0.09
300	511.354	511.241	511.323	511.223	511.209	511.27	0.06
1500	511.167	511.421	511.381	511.348	511.328	511.33	0.10
3600		511.434	511.499	511.389	511.374	511.42	0.06
7200		511.4	511.563	511.566	511.525	511.51	0.08
14400	511.705	511.651	511.805	511.639	511.649	511.69	0.07

DNA

Time	Replicate 1	Replicate 2	Replicate 3	Replicate 4	Replicate 5	average	SD
0	511.061	511.072				511.07	0.01
30	511.174	511.152				511.16	0.02
45							
60	511.207	511.182				511.19	0.02
300	511.216	511.172				511.19	0.03
1500	511.212	511.212				511.21	0.00
3600	511.305	511.212				511.26	0.07
7200	511.341	511.303				511.32	0.03
14400	511.457	511.309				511.38	0.10

ONPFDNA

Time	Replicate 1	Replicate 2	Replicate 3	Replicate 4	average	SD
0	511.104	511.255	511.079		511.15	0.10
30	511.169	511.224	511.146		511.18	0.04
45	511.194	511.264			511.23	0.05
60	511.172		511.203		511.19	0.02
300	511.176		511.192		511.18	0.01
1500	511.143	511.308	511.192		511.21	0.08
3600	511.273	511.268	511.231		511.26	0.02
7200	511.251	511.248	511.252		511.25	0.00
14400	511.193	511.268	511.33		511.26	0.07

TMG

Time	Replicate 1	Replicate 2	Replicate 3	Replicate 4	Replicate 5	average	SD
0	511.09	511.058				511.07	0.02
30	511.151	511.259				511.21	0.08
45	511.32	511.173				511.25	0.10
60	511.21	511.36				511.29	0.11
300	511.228	511.306				511.27	0.06
1500	511.282	511.176				511.23	0.07
3600	511.39					511.39	
7200	511.371	511.432				511.40	0.04
14400	511.529	511.589				511.56	0.04

296-303 LGQTSVDR

Charge 2

IPTG

Time	centroid	D
0	438.474	0.02
30	438.6195	0.48
45	438.6285	0.51
60	438.6435	0.56
300	438.634	0.53
1500	438.625	0.50
3600	438.615	0.47
7200	438.6485	0.58
14400	438.636	0.54

ONPF

Time	centroid	D
0	438.476	0.03
30	438.5825	0.37
45	438.6215	0.49
60	438.6125	0.46
300	438.612	0.46
1500	438.6185	0.48
3600	438.6265	0.51
7200	438.671	0.65
14400	438.63	0.50

APO

Time	centroid	D
0	438.467	0.00
30	438.59	0.39
45	438.5975	0.41
60	438.611	0.46
300	438.586	0.38
1500	438.626	0.50
3600	438.638	0.54
7200	438.669	0.64
14400	438.803	1.07

DNA

Time	centroid	D
0	438.4635	-0.01
30	438.587	0.38
45	438.584	0.37
60	438.605	0.44
300	438.6	0.42
1500	438.597	0.41
3600	438.6025	0.43
7200	438.612	0.46
14400	438.605	0.44

ONPFDNA

Time	centroid	D
0	438.465	-0.01
30	438.581	0.36
45	438.5985	0.42
60	438.5955	0.41
300	438.581	0.36
1500	438.5805	0.36
3600	438.5995	0.42
7200	438.618	0.48
14400	438.589	0.39

TMG

Time	centroid	D
0	438.4695	0.01
30	438.6425	0.56
45	438.646	0.57
60	438.7505	0.90
300	438.662	0.62
1500	438.6745	0.66
3600	438.662	0.62
7200	438.657	0.60
14400	438.7105	0.77

control	438.467
infinity	440.358

$$D(t) = \frac{Mt - M0}{M\infty - M0} *N$$

IPTG

Time	Replicate 1	Replicate 2	Replicate 3	Replicate 4	Replicate5	average	SD
0	438.479	438.469				438.47	0.01
30	438.667	438.572				438.62	0.07
45	438.665	438.592				438.63	0.05
60	438.7	438.587				438.64	0.08
300	438.685	438.583				438.63	0.07
1500	438.658	438.592				438.63	0.05
3600	438.648	438.582				438.62	0.05
7200	438.684	438.613				438.65	0.05
14400	438.672	438.6				438.64	0.05

ONPF

Time	Replicate 1	Replicate 2	Replicate 3	Replicate 4	average	SD
0	438.475	438.477			438.48	0.00
30	438.608	438.557			438.58	0.04
45	438.663	438.58			438.62	0.06
60	438.63	438.595			438.61	0.02
300	438.64	438.584			438.61	0.04
1500	438.644	438.593			438.62	0.04
3600	438.675	438.578			438.63	0.07
7200	438.748	438.594			438.67	0.11
14400	438.793	438.459			438.63	0.24

APO

Time	Replicate 1	Replicate 2	Replicate 3	Replicate 4	Replicate 5	average	SD
0	438.466	438.468				438.47	0.00
30	438.62	438.56				438.59	0.04
45	438.632	438.563				438.60	0.05
60	438.643	438.579				438.61	0.05
300	438.618	438.554				438.59	0.05
1500	438.643	438.609				438.63	0.02
3600	438.669	438.607				438.64	0.04
7200	438.72	438.618				438.67	0.07
14400	438.907	438.699				438.80	0.15

DNA

Time	Replicate 1	Replicate 2	Replicate 3	Replicate 4	Replicate 5	average	SD
0	438.461	438.466				438.46	0.00
30	438.617	438.557				438.59	0.04
45	438.604	438.564				438.58	0.03
60	438.647	438.563				438.61	0.06
300	438.632	438.568				438.60	0.05
1500	438.62	438.574				438.60	0.03
3600	438.639	438.566				438.60	0.05
7200	438.647	438.577				438.61	0.05
14400	438.67	438.54				438.61	0.09

ONPFDNA

Time	Replicate 1	Replicate 2	Replicate 3	Replicate 4	average	SD
0	438.465	438.465			438.47	0.00
30	438.597	438.565			438.58	0.02
45	438.612	438.585			438.60	0.02
60	438.612	438.579			438.60	0.02
300	438.593	438.569			438.58	0.02
1500	438.582	438.579			438.58	0.00
3600	438.612	438.587			438.60	0.02
7200	438.655	438.581			438.62	0.05
14400	438.591	438.587			438.59	0.00

TMG

Time	Replicate 1	Replicate 2	Replicate 3	Replicate 4	Replicate 5	average	SD
0	438.473	438.466				438.47	0.00
30	438.621	438.664				438.64	0.03
45	438.628	438.664				438.65	0.03
60	438.659	438.842				438.75	0.13
300	438.648	438.676				438.66	0.02
1500	438.621	438.728				438.67	0.08
3600	438.665	438.659				438.66	0.00
7200	438.637	438.677				438.66	0.03
14400	438.677	438.744				438.71	0.05

297-304 GQTSVDRL

Charge 1

IPTG

Time	centroid	D
0	438.474	0.04
30	438.574	0.34
45	438.5835	0.37
60	438.5875	0.38
300	438.6045	0.43
1500	438.586	0.38
3600	438.5955	0.40
7200	438.636	0.52
14400	438.556	0.29

ONPF

Time	centroid	D
0	438.47	0.04
30	438.55	0.27
45	438.57	0.34
60	438.58	0.35
300	438.58	0.34
1500	438.59	0.39
3600	438.58	0.35
7200	438.58	0.36
14400		

APO

Time	centroid	D
0	438.4625	0.01
30	438.568	0.32
45	438.5625	0.31
60	438.5735	0.34
300	438.5585	0.29
1500	438.614	0.46
3600	438.579	0.35
7200	438.6085	0.44
14400	438.685	0.67

DNA

Time	centroid	D
0	438.458	-0.01
30	438.5475	0.26
45	438.5675	0.32
60	438.563	0.31
300	438.5745	0.34
1500	438.5855	0.37
3600	438.5885	0.38
7200	438.5655	0.31
14400	438.5725	0.33

ONPFDNA

Time	centroid	D
0	438.462	0.01
30	438.5545	0.28
45	438.561	0.30
60	438.5595	0.30
300	438.5555	0.28
1500	438.549	0.27
3600	438.564	0.31
7200	438.563	0.31
14400	438.5505	0.27

TMG

Time	centroid	D
0	438.46	-0.01
30		
45	438.58	0.35
60	438.65	0.56
300	438.59	0.38
1500	438.62	0.46
3600	438.61	0.43
7200	438.60	0.42
14400		

control	438.46
infinity	440.475

$$D(t) = \frac{M_t - M_0}{M_\infty - M_0} * N$$

IPTG

Time	Replicate 1	Replicate 2	Replicate 3	Replicate 4	Replicate5	average	SD
0	438.487	438.461				438.47	0.02
30	438.605	438.543				438.57	0.04
45	438.617	438.55				438.58	0.05
60	438.628	438.547				438.59	0.06
300	438.666	438.543				438.60	0.09
1500	438.622	438.55				438.59	0.05
3600	438.634	438.557				438.60	0.05
7200	438.636					438.64	
14400		438.556				438.56	

ONPF

Time	Replicate 1	Replicate 2	Replicate 3	Replicate 4	average	SD
0	438.467	438.477			438.47	0.01
30	438.565	438.534			438.55	0.02
45	438.596	438.551			438.57	0.03
60	438.59	438.562			438.58	0.02
300	438.596	438.554			438.58	0.03
1500	438.611	438.57			438.59	0.03
3600	438.607	438.551			438.58	0.04
7200	438.608	438.554			438.58	0.04
14400					#DIV/0!	

APO

Time	Replicate 1	Replicate 2	Replicate 3	Replicate 4	Replicate 5	average	SD
0	438.465	438.46				438.46	0.00
30	438.592	438.544				438.57	0.03
45	438.59	438.535				438.56	0.04
60	438.595	438.552				438.57	0.03
300	438.583	438.534				438.56	0.03
1500	438.653	438.575				438.61	0.06
3600	438.583	438.575				438.58	0.01
7200	438.661	438.556				438.61	0.07
14400	438.745	438.625				438.69	0.08

DNA

Time	Replicate 1	Replicate 2	Replicate 3	Replicate 4	Replicate 5	average	SD
0	438.458	438.458				438.46	0.00
30	438.565	438.53				438.55	0.02
45	438.59	438.545				438.57	0.03
60	438.581	438.545				438.56	0.03
300	438.607	438.542				438.57	0.05
1500	438.605	438.566				438.59	0.03
3600	438.631	438.546				438.59	0.06
7200	438.601	438.53				438.57	0.05
14400	438.619	438.526				438.57	0.07

ONPFDNA

Time	Replicate 1	Replicate 2	Replicate 3	Replicate 4	average	SD
0	438.465	438.459			438.46	0.00
30	438.569	438.54			438.55	0.02
45	438.568	438.554			438.56	0.01
60	438.574	438.545			438.56	0.02
300	438.565	438.546			438.56	0.01
1500	438.54	438.558			438.55	0.01
3600	438.578	438.55			438.56	0.02
7200	438.593	438.533			438.56	0.04
14400	438.559	438.542			438.55	0.01

TMG

Time	Replicate 1	Replicate 2	Replicate 3	Replicate 4	Replicate 5	average	SD
0	438.459	438.453				438.46	0.00
30							
45	438.576					438.58	
60	438.584	438.715				438.65	0.09
300	438.586					438.59	
1500	438.559	438.671				438.62	0.08
3600	438.597	438.613				438.61	0.01
7200	438.578	438.626				438.60	0.03
14400							

304-307 LLQL

Charge 1

IPTG

Time	centroid	D
0	486.6065	0.00
30	486.8875	0.36
45	486.963	0.46
60	487.026	0.54
300	487.2025	0.77
1500	487.4555	1.10
3600	487.7605	1.50
7200	487.803	1.56
14400	487.7145	1.44

ONPF

Time	centroid	D
0	486.629	0.02
30	486.722	0.15
45	486.867	0.34
60	486.951	0.44
300	487.041	0.56
1500	487.2275	0.81
3600	487.3195	0.93
7200	487.3185	0.92
14400	487.24	0.82

APO

Time	centroid	D
0	486.607	0.00
30	486.7885	0.23
45	486.8695	0.34
60	487.0185	0.53
300	487.1405	0.69
1500	487.443	1.09
3600	487.639	1.34
7200	487.605	1.30
14400	487.73	1.46

DNA

Time	centroid	D
0	486.607	0.00
30	486.726	0.15
45	486.964	0.46
60	487.0085	0.52
300	487.094	0.63
1500	487.373	0.99
3600	487.423	1.06
7200	487.4135	1.05
14400	487.3465	0.96

ONPFDNA

Time	centroid	D
0	486.594	-0.02
30	486.704	0.12
45	486.803	0.25
60	486.9125	0.39
300	486.9915	0.50
1500	487.1525	0.71
3600	487.4065	1.04
7200	487.31	0.91
14400	487.2065	0.78

TMG

Time	centroid	D
0	486.6125	0.00
30	486.9065	0.39
45	487.0195	0.53
60	486.9685	0.47
300	487.3065	0.91
1500	487.4165	1.05
3600	487.6965	1.42
7200	487.6925	1.41
14400	487.879	1.65

control 486.61
infinity 488.144

$$D(t) = \frac{M_t - M_0}{M_\infty - M_0} * N$$

IPTG

Time	Replicate 1	Replicate 2	Replicate 3	Replicate 4	Replicate5	average	SD
0	486.61	486.603				486.61	0.00
30	486.978	486.797				486.89	0.13
45	487	486.926				486.96	0.05
60	486.988	487.064				487.03	0.05
300	487.238	487.167				487.20	0.05
1500	487.609	487.302				487.46	0.22
3600	487.794	487.727				487.76	0.05
7200	487.932	487.674				487.80	0.18
14400	487.821	487.608				487.71	0.15

ONPF

Time	Replicate 1	Replicate 2	Replicate 3	Replicate 4	average	SD
0	486.634	486.624			486.63	0.01
30	486.766	486.678			486.72	0.06
45	487.026	486.708			486.87	0.22
60	486.984	486.918			486.95	0.05
300	487.164	486.918			487.04	0.17
1500	487.389	487.066			487.23	0.23
3600	487.536	487.103			487.32	0.31
7200	487.473	487.164			487.32	0.22
14400	487.733	486.739			487.24	0.70

APO

Time	Replicate 1	Replicate 2	Replicate 3	Replicate 4	Replicate 5	average	SD
0	486.602	486.612				486.61	0.01
30	486.787	486.79				486.79	0.00
45	486.905	486.834				486.87	0.05
60	486.931	487.106				487.02	0.12
300	487.197	487.084				487.14	0.08
1500	487.388	487.498				487.44	0.08
3600	487.694	487.584				487.64	0.08
7200	487.74	487.47				487.61	0.19
14400	487.839	487.621				487.73	0.15

DNA

Time	Replicate 1	Replicate 2	Replicate 3	Replicate 4	Replicate 5	average	SD
0	486.593	486.621				486.61	0.02
30	486.729	486.723				486.73	0.00
45	487.169	486.759				486.96	0.29
60	486.98	487.037				487.01	0.04
300	487.115	487.073				487.09	0.03
1500	487.369	487.377				487.37	0.01
3600	487.527	487.319				487.42	0.15
7200	487.493	487.334				487.41	0.11
14400	487.717	486.976				487.35	0.52

ONPFDNA

Time	Replicate 1	Replicate 2	Replicate 3	Replicate 4	average	SD
0	486.603	486.585			486.59	0.01
30	486.72	486.688			486.70	0.02
45	486.884	486.722			486.80	0.11
60	486.945	486.88			486.91	0.05
300	487.046	486.937			486.99	0.08
1500	487.146	487.159			487.15	0.01
3600	487.525	487.288			487.41	0.17
7200	487.381	487.239			487.31	0.10
14400	487.371	487.042			487.21	0.23

TMG

Time	Replicate 1	Replicate 2	Replicate 3	Replicate 4	Replicate 5	average	SD
0	486.623	486.602				486.61	0.01
30	486.851	486.962				486.91	0.08
45	487.144	486.895				487.02	0.18
60	487.032	486.905				486.97	0.09
300	487.283	487.33				487.31	0.03
1500	487.334	487.499				487.42	0.12
3600	487.615	487.778				487.70	0.12
7200	487.478	487.907				487.69	0.30
14400	487.77	487.988				487.88	0.15
