# Supplementary material for: Ligand-specific changes in conformational flexibility mediate long-range allostery in the lac repressor
Source: Nat Commun. 2023 Mar 2;14:1179. doi: 10.1038/s41467-023-36798-1 (PMC9977783; doi:10.1038/s41467-023-36798-1)
Supplement: Supplementary file 6 — Reporting Summary [file 41467_2023_36798_MOESM6_ESM.pdf]

nature portfolio

Corresponding author(s): Anum Glasgow

Last updated by author(s): Jan 27, 2023

Reporting Summary

Nature Portfolio wishes to improve the reproducibility of the work that we publish. This form provides structure for consistency and transparency in reporting. For further information on Nature Portfolio policies, see our Editorial Policies and the Editorial Policy Checklist.

Statistics

For all statistical analyses, confirm that the following items are present in the figure legend, table legend, main text, or Methods section.

n/a	Confirmed	
☐	☒	The exact sample size (*n*) for each experimental group/condition, given as a discrete number and unit of measurement
☐	☒	A statement on whether measurements were taken from distinct samples or whether the same sample was measured repeatedly
☒	☐	The statistical test(s) used AND whether they are one- or two-sided<br>*Only common tests should be described solely by name; describe more complex techniques in the Methods section.*
☒	☐	A description of all covariates tested
☐	☒	A description of any assumptions or corrections, such as tests of normality and adjustment for multiple comparisons
☐	☒	A full description of the statistical parameters including central tendency (e.g. means) or other basic estimates (e.g. regression coefficient) AND variation (e.g. standard deviation) or associated estimates of uncertainty (e.g. confidence intervals)
☒	☐	For null hypothesis testing, the test statistic (e.g. *F*, *t*, *r*) with confidence intervals, effect sizes, degrees of freedom and *P* value noted<br>*Give P values as exact values whenever suitable.*
☒	☐	For Bayesian analysis, information on the choice of priors and Markov chain Monte Carlo settings
☒	☐	For hierarchical and complex designs, identification of the appropriate level for tests and full reporting of outcomes
☒	☐	Estimates of effect sizes (e.g. Cohen's *d*, Pearson's *r*), indicating how they were calculated

Our web collection on statistics for biologists contains articles on many of the points above.

Software and code

Policy information about availability of computer code

Data collection	HDX/MS data acquisition was controlled using Xcalibur software (version 4.1, Thermo Fisher Scientific), or Compass HyStar software (version 5.1.8.1, Bruker Daltonik GmbH) for the ternary state experiment. Computational modeling was performed using Rosetta 3.9 (version 2019.19). The biolayer interferometry experiment was performed using an Octet RED96 system (Pall ForteBio LLC) and analyzed using Octet Data Analysis HT software (version 9.0, Pall ForteBio LLC).
Data analysis	For each HDX/MS sample, peptides were identified using combined MS/MS raw data by searching against the LacI amino acid sequence (UniProtKB, www.uniprot.org, accessed 6/19/2019) and the pepsin amino acid sequence using Proteome Discoverer software (version 1.3, SEQUEST algorithm, Thermo Fisher Scientific), Byonic software (Protein Metrics, Cupertino, CA), or BioTools software (Bruker Daltonics). The exported peptide lists (sequence, charge state, and retention time) from separate samples were imported into HD Examiner 2.0 (Sierra Analytics, Modesto, CA) and combined. Custom scripts to analyze computational water placement are available at https://github.com/anumazam/watermaps_analysis_scripts. The associated DOI is 10.5281/zenodo.7577312.

For manuscripts utilizing custom algorithms or software that are central to the research but not yet described in published literature, software must be made available to editors and reviewers. We strongly encourage code deposition in a community repository (e.g. GitHub). See the Nature Portfolio guidelines for submitting code & software for further information.

Data

Policy information about availability of data

All manuscripts must include a data availability statement. This statement should provide the following information, where applicable:

- Accession codes, unique identifiers, or web links for publicly available datasets
- A description of any restrictions on data availability
- For clinical datasets or third party data, please ensure that the statement adheres to our policy

Data availability statement: All data generated in this study are provided in the Supplementary information, Supplementary files, and Source File. The raw MS data have been deposited in the Zenodo database under accession code 10.5281/zenodo.7585854 [http://doi.org/10.2210/pdb6YNV/pdb] (raw MS files on Zenodo). PDB files used for protein modeling include: 2PAF [https://www.wwpdb.org/pdb?id=pdb_00002paf] (ONPF-bound LacI core domain), 2P9H [https://www.wwpdb.org/pdb?id=pdb_00002p9h] (IPTG-bound LacI core domain), and 1LBI [https://www.wwpdb.org/pdb?id=pdb_00001lbi] (apo LacI core domain with tetramerization helix). All Python scripts for structural water modeling and WaterMap generation available on Github (https://github.com/anumazam/watermaps_analysis_scripts) with DOI: 10.5281/zenodo.7577312.

Human research participants

Policy information about studies involving human research participants and Sex and Gender in Research.

Reporting on sex and gender	n/a
Population characteristics	n/a
Recruitment	n/a
Ethics oversight	n/a

Note that full information on the approval of the study protocol must also be provided in the manuscript.

Field-specific reporting

Please select the one below that is the best fit for your research. If you are not sure, read the appropriate sections before making your selection.

☒ Life sciences ☐ Behavioural & social sciences ☐ Ecological, evolutionary & environmental sciences

For a reference copy of the document with all sections, see nature.com/documents/nr-reporting-summary-flat.pdf

Life sciences study design

All studies must disclose on these points even when the disclosure is negative.

Sample size	We ran at least three biological replicates for HDX/MS experiments for each functional state of LacI except for TMG-LacI and the ternary state. The TMG experiments were performed in duplicate and the ternary state experiment in singlicate. Both TMG and ternary state experiments were performed as validation experiments: TMG as an additional inducer molecule to compare with IPTG, and the ternary state to explore how both inducer and DNA binding would affect the conformational ensemble of LacI. In the ternary state experiment, the IPTG-LacI and DNA-LacI conditions were repeated and found to be in agreement with the original set of experiments. These sample sizes were chosen in accordance with accepted community standards for performing HDX/MS experiments as described in Masson et al.
Data exclusions	We excluded data from peptides for which HDX/MS for at least one state was missing, or for which more than one replicate was missing, or for which the fully-deuterated peptide data were missing. We also excluded data for peptides that were determined to be low-quality by the HD Examiner software. For peptides with HDX/MS corresponding to more than one charge state, we used the most complete and high-quality dataset as determined by HD Examiner, except for the ternary state experiment (where all the high quality charge states were used if multiple were found).
Replication	Experiments were all performed independently and repeated 3-5 times as described in the manuscript, except as detailed above in "sample size" for the TMG and ternary complex experiments.
Randomization	We ran HDX/MS such that biological replicates were performed on different days and experiments for different functional states were also performed in random combinations on different days. We also scheduled sample injections for different timepoints in a non-sequential order (except for the ternary state experiment, where injections were in timepoint order).
Blinding	Blinding was not performed in this study because we had to set up the experiments, so it was not possible not to know which experiment was being run at any time. However, all HDX/MS data were collected by a robotic system. Also, the computational water placement simulations were conducted at the same time as the HDX/MS data were collected.

Reporting for specific materials, systems and methods

We require information from authors about some types of materials, experimental systems and methods used in many studies. Here, indicate whether each material, system or method listed is relevant to your study. If you are not sure if a list item applies to your research, read the appropriate section before selecting a response.

Materials & experimental systems

n/a	Involved in the study
☒	☐ Antibodies
☒	☐ Eukaryotic cell lines
☒	☐ Palaeontology and archaeology
☒	☐ Animals and other organisms
☒	☐ Clinical data
☒	☐ Dual use research of concern

Methods

n/a	Involved in the study
☒	☐ ChIP-seq
☒	☐ Flow cytometry
☒	☐ MRI-based neuroimaging
